# Proteome Profiling of the Mutagen-Induced Morphological and Yield Macro-Mutant Lines of *Nigella sativa* L.

**DOI:** 10.3390/plants8090321

**Published:** 2019-09-02

**Authors:** Ambreen Asif, Mohammad Yunus K. Ansari, Abeer Hashem, Baby Tabassum, Elsayed Fathi Abd_Allah, Altaf Ahmad

**Affiliations:** 1 Department of Botany, Aligarh Muslim University, Aligarh 202002, India; 2 Botany and Microbiology Department, College of Science, King Saud University, P.O. Box. 2460, Riyadh 11451, Saudi Arabia; 3 Mycology and Plant Disease Survey Department, Plant Pathology Research Institute, ARC, Gaza 12511, Egypt; 4 Toxicology Laboratory, Department of Zoology, Govt. Raza PG College, Rampur 244901, UP, India; 5 Plant Production Department, College of Food and Agricultural Sciences, King Saud University, P.O. Box 2460, Riyadh 11451, Saudi Arabia

**Keywords:** black cumin, medicinal plant, mutagens, mutant lines, *Nigella sativa*, proteomics

## Abstract

In the present investigation, the leaf proteome profile of the macro-mutant lines of *Nigella sativa* L. was analyzed to identify the key proteins involved in the expression of traits associated with the morphology, seed yield, and content of thymoquinone. In our earlier study, the macro-mutants were generated with contrasting morphological traits and seed yields through induced mutagenesis, using ethyl methyl sulfonate, gamma rays, and combinations of both. Analysis of the leaf proteome of the control and macro-mutant lines of *N. sativa* showed that twenty-three proteins were differentially expressed. These differentially expressed proteins were sequenced through mass spectrometry and identified using the MASCOT software. On the basis of their function, these proteins were categorized into several groups. Most proteins were found in the categories of signal transduction (18%) and carbon metabolism (18%). A total of 13% of proteins belonged to the categories of energy and metabolism. Proteins in the categories of secondary plant metabolism, stress defense, cytoskeleton, and protein synthesis were also found. The polycomb group protein (FIE1), transcription factor (PRE1), and geranyl diphosphate synthase were notable proteins, in addition to some proteins of signal transduction and carbon metabolism. Expression patterns of the differentially expressed proteins were also studied at the transcript level by using qRT-PCR. Transcriptomics analysis was consistent with the proteomics data. This study shows the changes that take place at the proteomic level through induced mutagenesis, as well as the involvement of some proteins in the expression traits associated with plant height, seed yield, and the thymoquinone content of *N. sativa*. The identified proteins might help elucidate the metabolic pathways involved in the expression of traits, including seed yield, and the active compounds of medicinal plants.

## 1. Introduction

Medicinal and aromatic plants are one of the most important sources of life saving drugs for the majority of the world’s population. A large population of the world chiefly relies on herbal medicine. An estimate of the World Health Organization showed that approximately 80% of the population of less developed countries depend almost exclusively on traditional medicine for their primary healthcare requirements. Thus, medicinal and aromatic plants are utilized regularly by more than three billion people in these countries [1]. These plants also provide a good source of income. With an increase in the economic growth of herbal products during the last decade, it is estimated that the market for herbal medicines will cross USD 107 billion by the end of 2020 [2]. As the demand for herbal medicines is increasing globally, the supply of medicinal plants is declining because most of this harvest is derived from wild and naturally growing resources. This over-exploitation of medicinal plants from natural resource has led these plants to be under the threat of extinction. The genetic improvement of medicinal plants to produce higher yields and more active ingredients might help fulfil the increasing demand for medicinal plants. These improved high-yielding medicinal plants can be used for commercial cultivation purposes. However, this strategy of genetic improvement can only be possible when there is a large genetic variability in the population. Induced mutagenesis is an important approach for enhancing the genetic variation in medicinal plants because medicinal plants have narrow genetic variability, and conventional breeding approaches for the development of genetically improved varieties to produce higher yields and active principles takes a long time [3]. The role of mutation breeding and plant mutagenesis in increasing the genetic variability for desired traits in various food crops has been proven beyond doubt [4,5,6]. Mutagen-induced mutation is a potential tool for improving different traits in crops. However, reports on the improvement of medicinal plants for cultivation purposes, using induced mutagenesis, are meager.

*Nigella sativa* L. is a potential medicinal and aromatic plant with a historical and religious background. It belongs to the botanical family of Ranunculaceae and is commonly known as black cumin. The seeds of *N. sativa* are used in traditional medicine for the treatment of a large number of ailments [7,8,9,10]. Due to its excellent therapeutic properties, *N. sativa* is considered to be among the top-ranked evidence-based herbal medicines. Thymoquinone is a major bioactive molecule in *N. sativa* seeds [11]. Effective utilization of *N. sativa* for therapeutic and trade uses will largely depend on its seed yield and quality in terms of thymoquinone content. Present germplasms of this plant might not meet future needs. There is an increasing demand for *Nigella* seed oil in various industries world-wide [12]. Since the production of black cumin is limited and the demand for seeds and fixed oil is increasing, the development of genetically improved *N. sativa* is needed so that future demand for this plant can be fulfilled. Since a high genetic similarity has been reported among populations of *N. sativa* [13,14], we have developed genetically variable mutant lines of this plant with contrasting morphological traits, seed yields, and thymoquinone content through induced mutagenesis [15]. Because our horizons of research are expanding through genomic, proteomic, and metabolomic approaches, thereby offering opportunities to explore significant information on medicinal plants, identification of the proteins involved in the expression of traits in these mutant lines might help elucidate the metabolic pathways involved in the expression of characters, including seed yield and thymoquinone content. These results will lay the foundation for the improvement of medicinal plants through a mutation breeding approach at the molecular level, to produce better yields and quality. In this way, these plants could be cultivated for commercial purposes, and over-exploitation of wild resources could be stopped to save these precious medicinal plants from extinction.

## 2. Results

### 2.1. Standardization of Protein Isolation Methods

Three different methods for the isolation of the leaf protein were used in this study to standardize the best protein isolation method for *N. sativa*. The isolated protein was further separated by 2-Dimensional Electrophoresis (DE). The maximum quantity of the protein was found by a phenol-based method (3.86 mg/g leaf fresh weight), followed by the trichloroacetic acid (TCA)-acetone method (3.38 mg/g leaf fresh weight). The minimum amount of the protein was reported by the Tris-buffer-based isolation method (2.94 mg/g leaf fresh weight). Analysis of the isolated protein by 2-DE revealed that there was also a variation in the number of protein spots among the isolated proteins obtained via these three methods. The number of protein spots was 259, 211, and 214 by the phenol-based, Tris-buffer-based, and the TCA-acetone based methods, respectively (Table 1). 

Optimization of the pH range of immobilized pH gradient (IPG) strips was also carried out to achieve the maximum resolved protein spots. An IPG strip with pH values of 3–10 resulted in an uneven resolution of protein spots. Due to the wide range of pH values for this IPG strip, the proteins were retained in the middle of the gel. Within the narrow pH range of the IPG strips, a lesser number of protein spots were resolved in the gels with a pH of 3–6 and a pH of 7–10 for the IPG strips. The maximum resolved protein spots were observed with the pH 4–7 IPG strip (Supplementary Figure S1). Hence, the IPG strip with a pH of 4–7 was used to separate the protein spots of all mutant lines in the 2-DE gels.

### 2.2. Proteome Profiling of Macro-Mutant Lines

#### 2.2.1. Number of Differentially Expressed Proteins in the Leaves of the Mutant Lines of *Nigella sativa*

**Figure 1:**
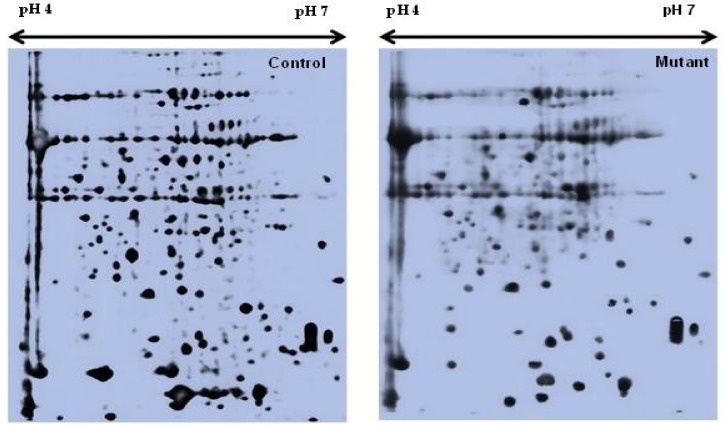
Representative 2-DE profiles of the leaf proteins of *Nigella sativa* L.

Two-dimensional gel electrophoresis was used to analyze the differentially expressed proteins of the mutant lines. Fully matured leaves from the control and all nine mutant lines were sampled for the leaf proteome analysis. Quantitative image analysis revealed that the Coomassie Brilliant Blue (CBB)-stained protein spots changed their intensities in the leaves of these mutant lines. The intensity of the protein spots was quantified, normalized, and relatively expressed. A representative 2D-gel image of the control and mutant plants is shown in Figure 1.

The intensity was quantified for each spot in each gel, taking *p* < 0.05 as significant, as per Dunnet’s test. This analysis revealed the change in the patterns of the expression of the proteins in the leaves. A total number of 219 to 260 protein spots were observed in the 2D profiles of the leaves of these mutant lines and the control (Table 2). A gel pattern was created between the 2D gels of all the plants, and the software detected that twenty-three spots showed differential expression among the mutant lines compared to the control. When a comparison was made between the expression patterns of the proteins in the control and those in the mutant plants, it was found that the number of down-regulated proteins in the mutant plants was in the range of 8–14 and the up-regulated proteins were 7–14 in the mutant plants. Interestingly, there was an appearance of new proteins in some mutant lines: three proteins in the HY1 mutant line, two proteins in the MHY4 mutant line, and one protein in the LY3 mutant line, compared to the control (Table 3).

#### 2.2.2. Identification and Expression Patterns of Differentially Expressed Proteins of Mutant lines 

The differentially expressed protein spots were identified, as shown in Table 4. The up-regulation and down-regulation of these proteins (compared with the control) are depicted in Table 5. Spot #1011 was identified as a large subunit of ribulose bis-phosphate carboxylase/oxygenase. The nominal mass and isoelectric point of this spot were 50,841 Da and 6.22, respectively. This protein is chloroplastic and is involved in the photosynthetic process. The expression of this protein was upregulated in mutant HY1, MHY2, and LY1, when compared to the control. In LY3, VLY2, and VLY3, the expression of this protein in the leaves was downregulated compared to the control plants. Expression of this protein in HY3 and NY3 was equal to the control. Spot #3401 was identified as ribulose bisphosphate carboxylase/oxygenase activase. The molecular weight (MW) and isoelectric point (pI) of this spot were found to be 48,079 Da and 6.20, respectively. This is a chloroplastic protein, found in the stroma. 

The rubisco activase is involved in the process of photosynthesis, through the activation of rubisco. The pattern of the expression of this protein in the leaves of different mutants of *N. sativa* was similar to that of spot #1011, except for NY3, where the expression of this protein was downregulated. Spot #4201 was the ATP synthase gamma chain. The MW and pI of this spot were found to be 41,706 Da and 8.1, respectively. It is also an ATP synthase subunit, involved in the synthesis of ATP. Compared to the control, the expression of this protein is upregulated in the leaves of the HY1, HY3, MHY4, NY3, LY3, and VLY2 mutants. In mutants MHY2, LY1, and VLY3, the expression of this protein is equal to that of the control plant (Table 4 and Table 5). 

Spot #1201 has been identified as oxygen evolving enhancer protein 1. The MW and pI of this spot were found to be 35,377 Da and 5.5, respectively. This protein is also a part of the photosynthetic pathway. It is located in the thylakoid membrane of the chloroplast. This protein stabilizes the manganese cluster, which is the primary site of water splitting. The expression of this protein in the leaves of the HY1, MHY2, and LY1 mutant lines was higher than that of the control plants, but for LY3, VLY2, and VLY3, its expression was downregulated. There was no difference in the expression of this protein in HY3, MHY4, and NY3, when compared to the control. Spot #4301 has been identified as the PSII stability/assembly factor, HCF 136. The molecular weight and pI of this protein spot were found to be 45,498 Da and 9.0, respectively. This is also a chloroplastic protein that is involved in the photosynthetic process of plants. This is an essential protein for PS II biogenesis and is required for the assembly of an early intermediate in PS II. The expression of this protein was higher in the leaves of the HY1, MHY2, and LY3 mutants than in the leaves of the control plants. In all other mutants (HY3, MHY2, NY3, LY1, VLY2, and VLY3), the expression of this protein was equal to that of the control plants. Spot #2101 has been identified as imidazole glycerol-phosphate dehydratase 1. The MW and pI of this spot were found to be 29,264 Da and 7.2, respectively. The cellular location of this protein is in the chloroplast. It is involved in the biosynthesis of amino acids. The expression of this protein was down regulated in the HY3, MHY2, NY3, LY1, VLY2, and VLY3 mutants compared to the control plants. In all other mutants (HY1, MHY4, and LY3), the expression of this protein was equal to that of the control plant. Spot #4401 has been identified as malate dehydrogenase-1. The MW and pI of this spot were found to be 35,570 Da and 6.11, respectively. This protein was present in the cytoplasm and was involved in the carbohydrate metabolic process. The expression pattern of this protein was equal in the HY1, MHY4, and LY3 mutant plants compared to the control. There was also an up regulation of this protein in HY3, MHY2, NY3, LY1, VLY2, and VLY3 mutant lines compared to the control.

Spot #4601 was identified as sucrose phosphate synthase 1. The MW and pI of this spot were found to be 119,743 Da and 6.0, respectively. It was located in the chloroplast membrane and was involved in sucrose biosynthesis by catalyzing the rate-limiting step. In comparison to the control, this protein was upregulated in mutants HY1, MHY4, and LY3 but was downregulated in LY1 and VLY3. The expression of this protein in HY3, MHY2, NY3, and VLY2 was equal to that of the control. Spot #6301 was identified as the 50 S ribosomal protein subunit L23. The MW and pI of this spot were found to be 10,046 Da and 10.2, respectively. It was located in the chloroplast and was involved in the translation process through binding to the 23S rRNA. The expression of this protein was upregulated in the mutants HY1, MHY4, LY3, and VLY2 compared to the control. In the mutants MHY2, LY1, and VLY3, there was a down-regulation of this protein compared to the control. There was no change in the expression pattern of this protein in mutants HY3 and NY3 over the control. Spot #-6801 was identified as an auxin-repressed 12.5 kDa protein. The MW and pI of this spot were found to be 12,408 Da and 9.1, respectively. This protein is involved in the auxin-activated signalling pathway. This protein was upregulated in HY1, MHY4, and LY3. Expression of this protein was equal to that of the control in the mutant lines HY3 and NY3. However, down regulation of this protein was found in the MHY2, LY1, VLY2, and VLY3 mutants of *Nigella sativa*. Spot #6901 has been identified as DNA-(apurinic or apyrimidinic site) lyase. The MW and pI of this spot were found to be 60,622 Da and 9.1, respectively. It is a chloroplastic protein that is involved in base-excision repair. Expression of this protein was equal to that of the control in the mutants, except for MHY2, LY1, and VLY3, where it was down regulated. Spot #7704 has been identified as 1-aminocyclopropane-1-carboxylate oxidase 2. The MW and pI of this spot were found to be 36,183 Da and 4.98, respectively. This protein is present in the cell wall, endoplasmic reticulum (ER), and cytosol, and involved in ethylene biosynthesis. This protein is upregulated in all the mutants except MHY2, LY1, and VLY3, where its expression is equal to that of the control. 

Spot #2908 has been identified as L-ascorbate peroxidase (APX1). The MW and pI of this spot were found to be 27,800 Da and 5.72, respectively. This protein is present in the cytoplasm and plays a role in the antioxidant defense system. Expression of this protein is upregulated in all the mutants over the control, except HY1, MHY4, and LY3, where its expression is equal to that of the control. Spot #3658 has been identified as manganese peroxide dismutase (MSD1). The MW and pI of this spot were found to be 25,443 Da and 8.47, respectively. This protein is present in the mitochondria and is involved in the defense response through the removal of superoxide anion radicals. The expression of this protein is similar to that of spot #2908. Spot #8054 is glyceraldehyde 3-phosphate dehydrogenase A (GAPDH). The MW and pI of this spot were found to be 36,914 Da and 6.62, respectively. This protein is found in the cytoplasm. Compared to the control, this protein was up-regulated in the leaves of the HY1, HY3, MHY4, NY3, LY3, and VLY2 mutants, and down-regulated in MHY2, LY1, and VLY3 (Table 5). Spot #1268 has been identified as a Tubulin alpha-6 chain (TUA6). The MW and pI of this spot were found to be 49,537 Da and 4.93, respectively. This protein is present in the cytoskeleton and involved in microtubule cytoskeleton organization. The expression of this protein was upregulated in the leaves of the HY1 and MHY2 mutant lines of *N. sativa* when compared with the control. In the mutants HY3, MHY4, and NY3, the expression of this protein was equal to that of the control. There was, however, down-regulation in the expression of this protein in the leaves of LY1, as well as VLY2 and VLY3. Spot #7315 has been identified as eukaryotic translation initiation factor 3, subunit I. The MW and pI of this spot were found to be 36,388 Da and 6.50, respectively. This spot is present in the cytoplasm and takes part in the synthesis of protein. This protein was upregulated in the mutants HY1, MHY2, and LY3 when compared to the control. In other mutant lines, down-regulation in the expression of this protein was observed in comparison to the control. Spot #2401 has been identified as allene oxide cyclase 3. This protein is present in the chloroplast and is involved in jasmonic acid biosynthesis. This protein was upregulated in the MHY2, LY1, and VLY3 mutants when compared with the control. In other mutants, the expression of this protein was equal to that of the control. Spot #3001 has been identified as deoxyuridine 5′-triphosphate nucleotidol hydrolase. This protein is involved in nucleotide metabolism and present in the cytoplasm. This protein was upregulated in HY1, MHY4, and LY3 mutants when compared with the control. In all the other mutants, the expression of this protein was equal to that of the control. Spot #3909 has been identified as the GTP-binding nuclear protein, Ran-1. The MW and pI of this spot were found to be 25,275 Da and 6.38, respectively. The expression pattern of this protein in the leaves of the mutants was similar to that of spot #6301. Spot #4006 is the polycomb group protein, FIE1. The MW and PI of this protein are 52,100 Da and 7.55, respectively. Compared to the control, the expression of this protein was upregulated in the HY1 and MHY2 mutant lines, when compared to the control. In all the other mutant lines, this protein was down-regulated, except in HY3 and MHY3, where its expression was equal to that of the control plant. Spot #4518 has been identified as the transcription factor, PRE1. This protein was up-regulated in the HY1 and MHY2 mutant lines, but down regulated in the NY3, LY1, LY3, VLY2, and VLY3 mutant lines when compared with the control. In the HY3 and MHY4 mutant lines, the expression of this protein is equal to that of the control. Spot #4900 has been identified as geranyl diphosphate synthase. Expression of this protein was up-regulated in the NY3, LY3, and VLY3 mutant lines, but remained equal to the control (Table 4 and Table 5).

#### 2.2.3. Functional Categorization of Differentially Expressed Proteins

**Figure 2:**
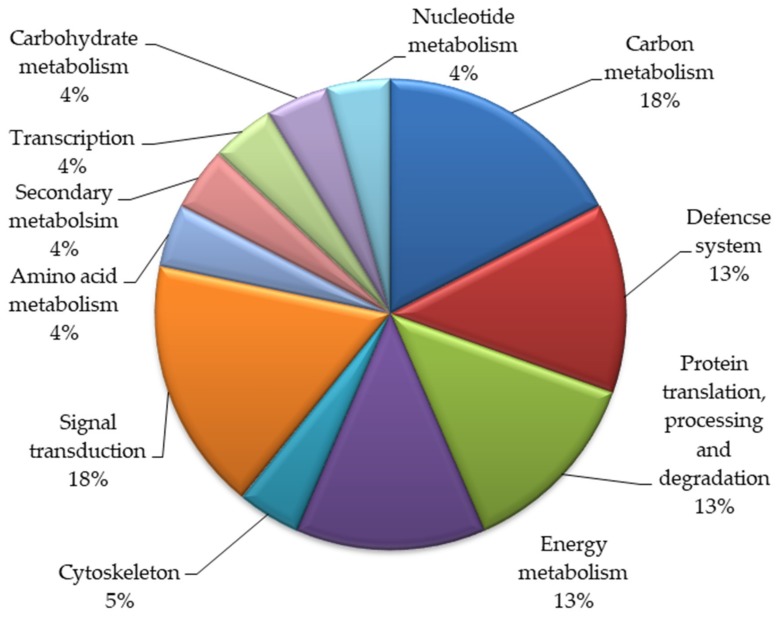
Functional categorization of the identified proteins in the mutant lines of *Nigella sativa* L.

Based on biological functions, the identified twenty-three proteins were classified into various functional groups (Figure 2). These proteins take part in various metabolic pathways. Most proteins belonged to the signal transduction (18%) and carbon metabolism (18%) categories. Thirteen percent of the proteins each belonged to the categories of energy and metabolism, the defense system and protein translation, processing, and degradation. Five percent of the total proteins were involved in the cytoskeleton, and 4% of these proteins were involved in amino acid metabolism, transcription, carbohydrate metabolism, and nucleotide metabolism.

#### 2.2.4. Expression of the Genes Encoding Differentially Expressed Proteins

**Figure 3:**
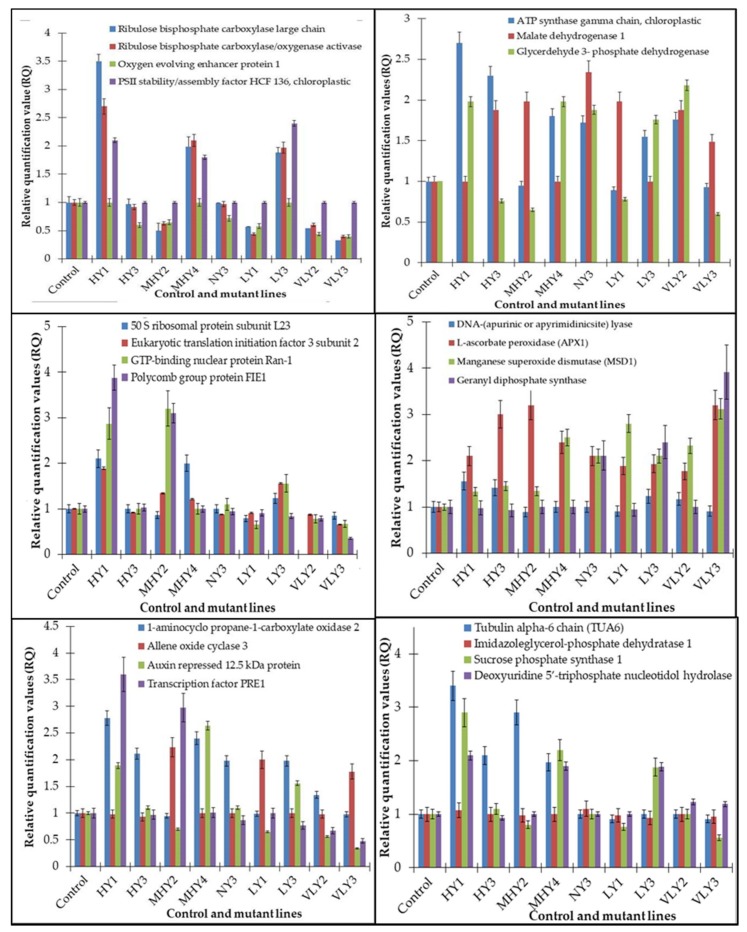
Expression analyses of the genes encoding differentially expressed proteins of the control and mutant lines of *Nigella sativa* L., as determined by quantitative RT-PCR. Values are the mean of three replicates (n = 3). Bars show the standard error. The normalization of the gene expression of mutant plants was done by setting the gene expression of the control as 1.

Correlation of the expression pattern of the proteins was made with the expression of the corresponding gene at the transcript level (Figure 3). Expression of most of the genes was similar to the protein expression. The expression pattern of five genes, however, was not correlated with the expression pattern of the protein and mRNA levels.

## 3. Discussion

Presently, improvement in the yield and quality of medicinal plants is a more important feature than the yield of the crop because of the pressing demand for medicinal plants in various industrial sectors. Since the genetic improvement program of any organism requires wide genetic variability in the population, the approach of induced mutagenesis is used to widen the genetic variation and diversity of medicinal plants so that medicinal plant with an improved yield and active principle can be developed. Improvement of medicinal plants to give them higher yields and more active ingredients through induced mutagenesis has been reported over the last several years, though these represent only 0.2% of the total crop varieties developed through mutation breeding [3]. In our earlier study, *Nigella sativa* L. was treated with ethyl methane sulfonate, γ-rays, and a combination of treatments to explore mutagen-induced genetic variability using chemical and physical mutagens; this study reported nine lines of mutants [15,16]. These mutant plants showed significant variability in their growth habits, shapes, and the colors of their leaves; the characteristics of their flowers and seeds; their seed thymoquinone content; and their seed yield [15]. Induced mutagenesis has many advantages because of its easy processing and requirement of little genetic information from the plant. Induced mutagenesis by EMS and γ-rays is currently the most suitable and prevalent method to induce point mutations. This approach has successfully generated mutants of several medicinal plants. Narcotic ‘opium poppy’ has been genetically converted into non-narcotic ‘seed poppy’ using EMS and γ-rays [17]. A distinct *Catharanthus roseus* mutant with unique inflorescence (monogenic recessive with leafless inflorescence architecture and increased flower frequency) and salt tolerance features has been developed through chemical mutagenesis [18]. EMS-induced macro-mutants of *C. roseus* were developed with high root and leaf alkaloids [19]. Mutagenesis has played a crucial role in improving some medicinal plants, such as *Atropa, Crocus*, *Papaver*, and *Psoralea* [20]. Eleven mutant lines of *Trigonella foenum-graecum* L. were identified to have better yield potential and higher diosgenin content by Floria and Ichim [21] using EMS and gamma rays. These reports confirm that induced mutations could be used for the genetic improvement of *Nigella sativa.* However, the use of random chemical mutagenesis as a genetic approach has some limitations because of difficulty in identifying the genes involved in the expression of the phenotypic traits of mutants [22].

To understand the phenotypic changes in the morphological and yield mutant lines of *N. sativa* at the molecular level, a proteomics approach was applied using 2-DE to determine the proteins involved in the expressing phenotypic traits. *Nigella sativa* contains a large number of interfering metabolites. These metabolites cause streaking of the gel and change the plant’s heterogeneity. Analysis of differentially expressed proteins through 2D gels requires that proteins be well separated with no streaking, background staining, or smearing. The phenol-based method was found to be better than other methods in the present study in terms of the quantity of the isolated protein. The quality of the isolated protein from the three methods of protein isolation was measured by separating the protein in a 2D gel system. The greatest numbers of protein spots have been observed in proteins isolated using the phenol-based method. Therefore, the phenol-based method of protein isolation was also used in this study. This method of protein isolation is preferred also over other protein isolation methods because of its high clean-up capacity. This method also hinders the molecular interaction between proteins and other interfering compounds [23]. The IPG strips with pH ranges of 3–6, 3–10, 4-7, and 7–10 were chosen to optimize the maximum resolution of the protein spots. For different plant tissues, IPG strips of different pH ranges were used to ensure that the pIs of the most proteins were covered and to prevent the clustering of proteins with similar pIs [24]. In general, for the separation of proteins from plant tissues, IPG strips of pH 4–7 were used because such strips can resolve more than 80% of the protein spots in a gel [25].

The Rubisco large subunit (Spot #1011) catalyzes the first step of CO_2_ assimilation and photorespiration [26]. Rubisco activity limits photosynthesis in C3 plants [27,28]. This enzyme was up-regulated in tall mutants, indicating that tall mutants can maintain a high efficiency of photosynthesis compared to the control. Increased photosynthesis enhances crop yields [29,30]. There was down-regulation of this enzyme in dwarf mutants when compared to the control. Ribulose bisphosphate carboxylase/oxygenase activase (Spot #3401) mediates the activation of Rubisco by facilitating the ATP-dependent removal of various inhibitory sugar phosphates from the Rubisco’s active site [31]. Reduced expression of Rubisco activase has been reported to decrease photosynthesis and growth rates [31]. This spot was down regulated in dwarf mutants and up-regulated in tall mutants. The down-regulation of Rubisco activase revealed that the photosynthetic apparatus undergoes extensive degradation [32], leading to a low photosynthetic rate. Overexpression of Rubisco and Rubisco activase in tall mutants indicated that these mutants increased the capacity of photosynthetic flux and biomass production compared to the other mutants and the control, thereby presenting good growth and seed yields. 

The Oxygen evolving enhancer protein (spot #1201) is a subunit of the oxygen-evolving complex of photosystem II in the chloroplast [33]. It stabilizes the PSII and, thus, has an important role in the primary reaction of photosynthesis. Up-regulation of this protein was observed in tall mutants. No effect on the intensity of this protein was found in the semi-dwarf mutant. It is suggested that oxygen-evolving complex proteins help the tall mutant plants adapt to adverse conditions, thereby releasing the oxygen-evolving enhancer protein. The gamma chain of ATP synthase (spot #4201) is involved in ATP biosynthetic pathway, producing ATP from ADP, and takes part in photophosphorylation [34]. In our study, this protein was up-regulated in both tall and semi-dwarf mutants. The expression of this protein was equal to that of the control in dwarf mutants. An earlier study at the transcript level showed the decreased expression of ATP synthase gamma chain under stress condition [35]. 

The Chloroplastic PSII stability/assembly factor HCF 136 (spot #4301) stabilizes and activates the PSII complex [36,37]. Overexpression of this protein in tall mutants, therefore, indicates activated PSII and efficient photosynthesis compared to the control and the other mutant lines. The GAPDH takes part in glycolysis, an important metabolic pathway providing energy and maintaining production of both primary and secondary metabolites [38]. It was up-regulated in tall and semi-dwarf mutants. The TCA cycle is a fundamental metabolic pathway, the intermediates of which form important metabolites and substrates for amino-acid synthesis. Malate dehydrogenase (spot #2101), an important enzyme of the TCA cycle, did not show any change in the expression of tall mutants compared to the control and was up-regulated in other mutant plants. Sucrose phosphate synthase (spot #4601) regulates the balance between starch and sucrose. It is involved in sucrose degradation, as well as sucrose synthesis [39]. This enzyme was up-regulated in tall mutants, possibly to meet energy demands. 

Deoxyuridine 5’-triphosphate nucleotide hydrolase (spot #3001) decreases the level of dUTP so that uracil cannot be incorporated into DNA. Down-regulation of this gene under stress conditions resulted in damaging the DNA [40]. Ascorbate peroxidase (spot #2908) and manganese peroxide dismutase (spot #3658) are the main enzymes of the antioxidant defense system of plants. Many plants cope with oxidative stress via the over-expression of ascorbate peroxidase and superoxide dismutase genes [41]. Expressions of these enzymes did not change in tall mutant plants in comparison to the control, indicating that tall mutants can also fight against adverse environmental conditions. 

Allene oxide cyclase (spot # 2401) is involved in the jasmonate biosynthetic pathway, catalyzing the most critical step [42]. Jasmonates (JAs) are involved in the regulation of a wide range of metabolic activities, from stress responses to development [43]. The JA acts as a signaling molecule in response to wounds and pathogenesis [44]. It also works as a stress hormone and promotes the synthesis of various primary and secondary metabolites in plants [45,46,47]. In our study, the expression of this protein was higher in the dwarf mutant line than in the tall and semi-dwarf mutant lines and the control. The up-regulation of this protein in dwarf mutants could be correlated with the higher level of thymoquinone content in the seeds of these mutants (Supplementary Table S2). It has been reported that the overexpression of the allene oxide cyclase gene (*HnAOC*) in tobacco led to the up regulation of genes from the nicotine biosynthetic pathway. This resulted in a 4.8-fold enhancement in the nicotine level over the control [46]. Transgenic *Artemisia annua*, overexpressing allene oxide cyclase (*AaAOC*), synthesized higher levels of endogenous JA and resulted in an increased biosynthesis of artemisinin [42]. 

Microtubules are the principal component of the cytoskeleton; microtubules continuously assemble and disassemble within the cell. The microtubules determine the cell’s shape. Tubulin is the major constituent of microtubules. The tubulin alpha-6 chain (spot #1268) in our study is up-regulated in tall mutants and down-regulated in the dwarf mutant. Reduced expression of the α-tubulin gene in *A. thaliana* caused cellular defects that resulted in poor growth and abnormal morphology of the root. A low level of a-tubulin proteins resulted in microtubule disassembly, causing a reduction in growth [48]. The down-regulation of this protein in dwarf mutants in our study could also be correlated with the reduced growth of these plants. In this study, the auxin-repressed protein (spot #6801) was up-regulated in dwarf mutant lines but did not show any change in other mutants compared to the control. Salicylic acid signaling is mediated by an auxin-repressed protein, which is induced by indole acetic acid [49]. Reports demonstrate the induction of auxin-repressed proteins by abiotic stresses, causing an arrest in growth, possibly by inhibiting cell elongation [50]. Transcription factor PRE1 has been found to regulate extensive developmental processes in plants. In this study, spot #4518 was up-regulated in tall mutant lines but down-regulated in the dwarf mutant lines of *N. sativa*. Plant height and seed size are directly affected by cell length. PRE1 has been reported to work in association with the ACE protein (cell elongation regulator) and AtIBH1 protein (cell elongation inhibitor) [51]. While the ACE induces cell elongation, AtIBH1 inhibits the activity of ACE. PRE1 has been found to promote elongation by indirectly interfering with the activity of AtIBH1. Final cell length is determined by the balance between the three proteins. These proteins are the transcription factors controlling the functions of several genes [51]. It is suggested that mutagens affect the expression of the PRE1 protein. Up-regulation of this protein produced tall mutant lines and down-regulation generated dwarf mutant lines. Polycomb proteins have been found to regulate extensive developmental processes in plants [52]. 

In the present study, spot #4006 was identified as the polycomb group protein FIE1. This protein was up-regulated in tall and semi-dwarf mutant lines. Liu et al. [52] explained that mutations in this protein resulted in the dwarfism and smaller grain size of rice. In the present study, spot #4900 is geranyl diphosphate synthase (GPPS), which is involved in the isoprenoid biosynthetic process and is the key enzyme of the monoterpene pathway. Analysis of its expression and thymoquinone content in the mutant lines of *N. sativa* demonstrated the correlation between the expression pattern of this protein and its thymoquinone content. The mutant lines (NY3, LY3, and VLY3) showed up-regulation of this protein compared to the control. The seed thymoquinone content of these plants was higher compared to the other mutant lines and the control (Supplementary Table S2). Kahila et al. [53] have shown that the application of TiO_2_ and SiO_2_ nanoparticles to *N. sativa* enhanced the expression of the GPPS gene, resulting in higher thymoquinone synthesis compared to the control. In the present experiment, the expressions of differentially expressed proteins were also validated at the transcript level. However, the transcript level expressions of some proteins were different from the translation level. This could be because of post-transcriptional or post-translational modifications.

## 4. Materials and Methods

### 4.1. The Macro-Mutant Lines of Nigella sativa L.

Asif and Ansari [15] generated nine mutants of the *N. sativa* L. var. Azad Kalaunji-1 by using chemical and physical mutagens (EMS, γ-rays, and their combinations). The morphological characteristics, seed yield, and seed thymoquinone content of these mutants are given in Supplementary Table S2. These mutant lines were used to study the proteome profile of leaves. Normal (control) plants were erect, with a moderate height of 50–60 cm, with normal branches featuring wispy and green leaves, white flowers with five petaloid sepals, numerous yellow anthers, a whorl of nectaries, a pentalocular capsule, black triquetrous seeds, and a normal seed yield. The thymoquinone content in the seeds of the control plant was 1002 µg/g.

### 4.2. Analysis of the Proteome Profile of the Macro-Mutant Lines

#### 4.2.1. Standardization of Protocols for Protein Isolation

The protocols for protein isolation from the leaves of *N. sativa* were standardized because many interfering compounds are present in the leaves of this plant. Methods involving TCA-actone, phenol, and Tris-buffer were used to standardize the protocol for protein isolation.

Granier [54] described the protein isolation method using Tris-buffer. One hundred milligrams of leaf tissue were crushed into a fine powder in Liq. N_2_. The powdered form of the leaves was placed in 1 mL extraction buffer (pre-chilled) containing 50 mM Tris (pH 7.5), 0.5% Triton X-100, and 15 μL 2 mM dithiothreitol (DTT). This was followed by incubation on ice for 1 h. After incubation, the centrifugation of the resultant solution was carried out for 30 min at 14,000 rpm at 4 °C. Ten percent of TCA-acetone solution was added to the supernatant and incubated at −20 °C overnight to precipitate the protein. Thereafter, centrifugation of the solution was carried out for 10 min at 12,000 rpm to pellet down the proteins. The pellet was washed using cold acetone. The washing process was repeated 2–3 times. The pellet was then dried.

Isaacson et al. [55] described a phenol-based method of protein isolation. One gram of leaf tissue was crushed into a fine powder in Liq. N_2_. The powdered form of the leaves was placed in an extraction buffer containing HEPES [4-(2-hydroxyethyl)-1-piperazine ethanesulfonic acid, 50 mM], sucrose (700 mM), PMSF (Phenylmethanesulfonyl fluoride, 1 mM), β-mercaptoethanol (2%), KCl (100 mM), and EDTA (Ethylenediaminetetraacetic acid, 50 mM) (pH 7.5). Fifteen milliliters of phenol was added to the extraction buffer and mixed for 30 min. The solution was centrifuged at 5000 rpm for 10 min at 4 °C to recover the upper phenolic layer. The upper phenolic layer was mixed with ice-cold 0.1 M ammonium acetate (15 mL). The resultant solution was incubated at −20 °C overnight to precipitate the protein. Centrifugation was carried out at 8000 rpm for 15 min at 4 °C to obtain the protein. Washing of the pellet was done by methanol followed by acetone. The pellet was then dried.

The TCA–acetone based method of protein isolation was given by Damerval et al. [56]. One gram of leaf tissue was crushed into a fine powder in Liq. N_2_. The powdered form of the leaves was placed in 2 mL of precipitation solution (10% TCA-Acetone and 0.07% β-mercaptoethanol). This was followed by incubation at −20°C for overnight. Centrifugation of the solution was carried out at 14,000 rpm for 10 min. Washing of the pellet was done with acetone and 0.07% β-mercaptoethanol. The pellet was thereafter air-dried.

#### 4.2.2. Quantification of Isolated Protein

The isolated proteins were solubilized in a solubilization buffer (2M thiourea, 4% CHAPS, 7M urea, and 50 mM DTT). Bradford’s method was used to quantify the protein [57].

#### 4.2.3. Two-Dimensional Gel Electrophoresis

Two-dimensional electrophoresis was carried out via the method of O’Farrel [58]. Initially, the pH range of IPG strips was optimized. For this purpose, 11 cm of IPG strips (pH 3–6, pH 3–10, pH 4–7, and pH 7–10) (Bio-Rad Laboratories, Inc., Hercules, CA, USA) was taken. Rehydration of the strips was carried out at 20 °C for 14 h in 250 μL of the protein sample (400 μg). Isoelectric focusing was carried out with the PROTEAN^®^IEF system (Bio-Rad Laboratories, Inc., Hercules, CA, USA) using the following programme: 200 volt (1 h), 600 volt (1 h), 1100 volt (2 h), 2000 volt (2 h), a linear increase of 7500 volt for 18 h and 500 volt (1 h). Thereafter, the IPG strips were placed in a buffer (50 mM Tris, 8.5 M urea, 25% glycerol, 2.0% SDS, and 150 mM DTT, pH 8.8) for reduction. Thereafter, the IPG strips were placed in an alkylation buffer (pH 8.8, Tris, 8.5 M urea, 25% glycerol, 2.0% SDS, and 145 mM iodoacetamide) for 15 min. The IPG strips were separated via second dimensional SDS-PAGE using 12% polyacrylamide with vertical gel electrophoresis. A constant voltage of 250 V was maintained. Staining of the gels was carried out in 0.1% (*w/v*) Coomassie brilliant blue G-250. Destaining was done, thereafter, by washing the gels several times with water. The maximum number of protein spots was obtained from the 11 cm IPG strip of pH 4–7 (Supplementary Figure S1). Therefore, the 11 cm IPG strip (pH 4–7) was used for proteome analysis. 

#### 4.2.4. Gel Imaging and Statistical Analysis

Gel imaging was carried out using a densitometer (GS-800, Bio-Rad, USA). PDQuest software (version 8.0, Bio-Rad, USA) was used for the detection and intensity quantification of protein spots. A reference gel that contained the highest number of spots was taken. The value of each protein spot was normalized in terms of the percentage of the total volume of all gel spots. A significant expression of the spots was detected at a 5% significance level (*p*-value < 0.05). The final refinement of these spots was carried out using a *p*-value <0.05 to discard false positive, a power of >0.8 to ensure reproducibility among gels of the same conditions, and a fold number >2 for the biological significance. Assistat version 7.7 beta software was used for the statistical analysis. 

#### 4.2.5. In-Gel Reduction

The in-gel reduction and alkylation of selected protein spots were carried out according to the method of Webster and David [59]. The selected spots of the differentially expressed proteins were incubated with 10 mM DTT for 1 h at 50 °C for reduction. After cooling to room temperature, the DTT solution was removed, and 50 mM iodoacetamide solution (100 μL) was added. For alkylation, incubation was performed for 1 h with occasional mixing. Washing of the gel pieces was done with 100 μL aqueous buffer (50 mM NH_4_HCO_3_) for 5 min and then with 100 μL organic buffer (Acetonitrile/50 mM NH_4_HCO_3_, 1:1). Drying of the gel was carried out in a centrifugal vacuum concentrator. A total of 10–20 μL trypsin (12.5 ng/μL) was added to digest the proteins. The resultant peptide mixture was acidified by adding 1 μL aqueous solution of formic acid (1% *v/v*). The digested proteins were stored at −20 °C until analysis.

#### 4.2.6. Identification and Functional Categorization of Differentially Expressed Proteins

The digested protein samples were analyzed by a mass spectrometer (MALDI-MS SCIEX TOF/TOF™ 5800 system, Applied Biosciences, USA). The mass spectrometer data were recorded in the positive ionization mode. Identification of the protein was carried out by searching the acquired mass spectrometer data in Swissprot (a non-redundant protein sequence database) using the MASCOT search engine version 3.5 (Matrix Science, UK). All the parameters for the identification of proteins were set according to Yousuf et al. [60]. The functional categorization of proteins was analyzed with websites, including UniProt (http://www.uniprot.org/).

### 4.3. Gene-Specific qRT-PCR

A Trizol reagent was used to isolate the total RNA from the leaves. DNA contamination from the isolated RNA was removed by treating the samples with DNase (RNase free). The RNA samples were quantified using NanoDrop (Thermo Scientific, USA). Synthesis of cDNA was then carried out using a cDNA synthesis kit (Bio-Rad, USA). A list of primers for the qRT-PCR analysis of the selected genes is given in Supplementary Table S3. Real-time PCR and calculations of the expression levels of the genes were carried out according to Yousuf et al. [61]. 

### 4.4. Statistical Analyses

Three biological replicates for each treatment and the control were used for statistical analyses. A two-tailed Student’s t-test with a significance of 95% was performed on the normalized value of the protein spots with the help of SPSS software.

## 5. Conclusions

Characterization of the molecular basis of mutagenesis has transformed mutation induction from chance events into science-based techniques. The present study provides a comparative proteomic analysis of mutant lines generated by induced mutagenesis. The results highlight the proteins that were differentially expressed between the control and mutant lines of *N. sativa*. A set of proteins was found to be potentially involved (directly or indirectly) in the expression of the plant height, seed yield, and thymoquinone content in the mutant lines. This group of proteins notably includes the proteins involved in carbon metabolism, signaling mechanisms, the differentiation of cells, carbohydrate catabolism, and secondary metabolism.

## Figures and Tables

**Table 1:** Comparison of the quantity of the protein and number of spots obtained by the three isolation methods.

Protein Isolation Method	Quantity of Protein (mg g^−1^ FW)	Number of Protein Spots
Phenol-based method	3.86 ± 0.113^a^	259 ± 10^a^
Tris-buffer-based method	2.94 ± 0.216^c^	211 ± 13^b^
TCA-acetone based method	3.38 ± 0.142^b^	214 ± 17^b^

* Values are Mean ± SD. The values followed by a similar letter are not significantly different (ANOVA test, *p* < 0.05). FW = Fresh Weight.

**Table 2:** Total number of the leaf protein spots of *Nigella sativa* L.

S.N.	Control/Mutants *	Number of Protein Spots
1.	Control	241
2.	HY1	258
3.	HY3	238
4.	MHY2	222
5.	MHY4	260
6.	NY3	249
7.	LY1	219
8.	LY3	255
9.	VLY2	251
10.	VLY3	220

* Abbreviations of mutant lines are as per Supplementary Table S2.

**Table 3:** Number of differentially expressed proteins in the leaves of the mutant lines of *Nigella sativa* L. The expression patterns of the proteins were compared with those of the control plant.

Patterns of Expression	Mutants Lines of *Nigella sativa* L.								
	HY1	HY3	MHY2	MHY4	NY3	LY1	LY3	VLY2	VLY3
Down-regulated	8	12	14	10	12	12	12	11	12
Up-regulated	13	8	7	14	8	9	13	8	9
Appearance of a new protein	3			2			1		

**Table 4:** Molecular functions, metabolic pathways, and accession numbers of the identified proteins.

Spot No.	Protein View	MASCOT Score	Nominal Mass (Da)	pI	Sequence Match (%)	Protein Name	Protein Function	Sub-Cellular Location	Pathway/Biological Process	Accession No.
1011	RBL_ERYCG	148	50841	6.22	65	Ribulose bisphosphate carboxylase large chain	Carboxylation of D-ribulose 1,5-bisphosphate	Chloroplast	Photosynthesis	Q33438
3401	RCA_MAIZE	119	48079	6.20	53	Ribulose bisphosphate carboxylase/oxygenase activase	Activation of Rubisco	Chloroplast stroma	Photosynthesis	Q9ZT00
4201	ATPG_TOBAC	90	41706	8.1	38	ATP synthase gamma chain, chloroplastic	Produces ATP from ADP in the presence of a proton gradient across the membrane.	Chloroplast thylakoid membrane	ATP synthesis, hydrogen ion transport	P29790
1201	PSBO_SPIOL	50	35377	5.5	30	Oxygen evolving enhancer protein 1, chloroplastic	It stabilizes the manganese cluster, which is the primary site of water splitting.	Chloroplast thylakoid membrane	Photosynthesis	P12359
4301	P2SAF_ORYSJ	50	45498	9.0	24	PSII stability/assembly factor HCF 136, chloroplastic	Essential for PS II biogenesis, required for assembly of an early intermediate in PSII assembly	Chloroplast thylakoid membrane, peripheral membrane protein	Photosynthesis	Q5Z5A8
2101	HIS7A_ARATH	74	29264	7.2	51	Imidazole glycerol- phosphate dehydratase 1	It catalyzes D-erythro-1-(imidazole-4-yl) glycerol 3 phosphate to 3-(imidazole-4-yl)-2-oxopropylphosphate releasing water.	Chloroplast	Amino acid biosynthesis, L-Histidine biosynthesis	Isoform 1: P34047-1 Isoform 2: P34047-2
4401	MDHC1_ARATH	37	35570	6.11	24	Malate dehydrogenase 1	It catalyzes a reversible NAD-dependent dehydrogenase reaction involved in central metabolism and redox homeostasis	Cytoplasm	Carbohydrate metabolic process	P93819
4601	SPSA1_CRAPL	312	119743	6.0	87	Sucrose phosphate synthase 1	It has a role in photosynthetic sucrose synthesis by catalyzing the rate limiting step of sucrose biosynthesis from UDP glucose and fructose-6- phosphate.	Chloroplast thylakoid membrane	Glycan biosynthesis, sucrose biosynthesis	O04932
6301	RK23_CHLUV	82	10046	10.2	63	50S ribosomal protein subunit L23	Binds to 23S rRNA	Chloroplast	Translation	P56368
6801	12KD_FRAAN	77	12408	9.1	64	Auxin repressed 12.5 kDa protein	Auxin-activated signaling pathway		Auxin signaling pathway	Q05349
6901	ARP_ARATH	92	60622	9.1	28	DNA-(apurinic or apyrimidinic) lyase	It repairs oxidative DNA damages.	Chloroplast	Base-excision repair	P45951
7704	ACCO2_ARATH	89	36183	4.98	49	1-aminocyclo propane-1-carboxylate oxidase 2	It is involved in ethylene biosynthesis.	Cell wall, ER, cytosol	Ethylene biosynthesis	Q41931
2908	APX1_ARATH	123	27800	5.72	68	L-ascorbate peroxidase (APX1)	It plays a key role in hydrogen peroxide removal.	Cytoplasm	Antioxidant defense system	Q05431
3658	SODM1_ARATH	89	25443	8.47	58	Manganese superoxide dismutase (MSD1)	It destroys superoxide anion radicals	Mitochondria	Defense response	O81235
8054	G3PC1_ARATH	100	36914	6.62	45	Glyceraldehyde 3- phosphate dehydrogenase	It catalyzes the first step of the pathway by converting D-glyceraldehyde 3-phosphate into 3-phospho D-glyceroyl phosphate	Cytoplasm	Glycolytic process	P25858
1268	TBA6_ARATH	115	49537	4.93	56	Tubulin alpha-6 chain (TUA6)	Structural constituent of cytoskeleton	Cytoskeleton	Microtubule cytoskeleton organization	P29511
7315	EIF3I_ARATH	125	36388	6.50	58	Eukaryotic translation initiation factor 3 subunit 2(eIF3I1/TRIP-1)	Component of eukaryotic initiation factor 3 (eIF-3)	Cytoplasm	Protein synthesis	Q38884
2401	AOC3_ARATH	66	28015	9.19	43	Allene oxide cyclase 3	It is involved in the production of 12-oxo-phytodienoic acid, a precursor of jasmonic acid.	Chloroplast	Jasmonic acid biosynthesis process	Q9LS01
3001	DUT_ARATH	70	17603	5.3	51	Deoxyuridine 5′-triphosphate nucleotidol hydrolase	It produces dUMP, the immediate precursor of thymidine nucleotides and decreases the intracellular concentration of Dutp	Cytoplasm	Nucleotide metabolism (pyrimidine metabolism)	Q9STG6
3909	RAN1_ORYS1	120	25275	6.38	56	GTP-binding nuclear protein Ran-1	It is involved in nucleocyto- plasmic transport.	Nucleus	Protein import into nucleus	P41916
4006	FIE1_ORYSJ	99	52,100	7.55	44	Polycomb group protein FIE1	It is involved in cell differentiation, endosperm development, and seed development.	Nucleus	Differentiation, Transcription, Transcription regulation	Q6ZJW8
4518	PRE1_ARATH	120	10,560	9.00	79	Transcription factor PRE1	It integrates multiple signaling pathways to regulate cell elongation and plant development.	Nucleus	Gibberellic acid mediated signaling pathway	Q9FLE9
4900	V5REB1_NIGSA	51	23010	5.67	72	Geranyl diphosphate synthase	It is involved in the isoprenoid biosynthetic process	Cytoplasm	Key gene of monotrepene pathway	V5REB1

**Table 5:** Fold-change abundance of differentially expressed leaf proteins of the control and the mutants of *Nigella sativa* L.

Protein Name	Expression Pattern of Proteins in Leaves of Control and Mutant Lines of *Nigella sativa* L.									
	Control	HY1	HY3	MHY2	MHY4	NY3	LY1	LY3	VLY2	VLY3
Ribulose bisphosphate carboxylase large chain	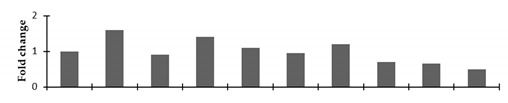									
Ribulose bisphosphate carboxylase/oxygenase activase	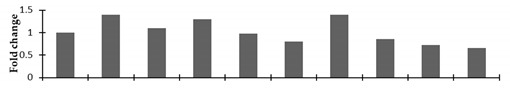									
ATP synthase gamma chain	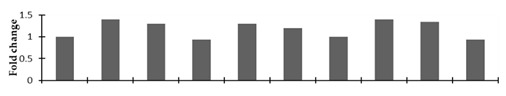									
Oxygen evolving enhancer protein 1	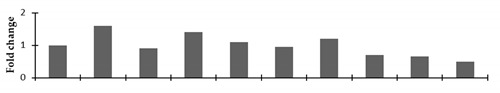									
PSII stability/assembly factor HCF 136, chloroplastic	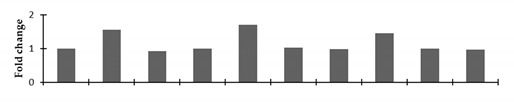									
Imidazole glycerol-phosphate dehydratase 1	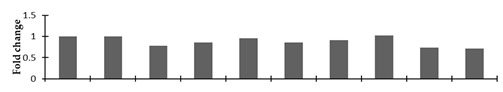									
Malate dehydrogenase 1	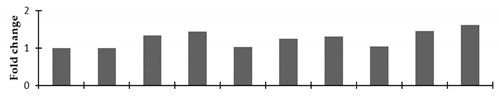									
Sucrose phosphate synthase 1	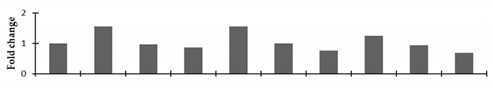									
50S ribosomal subunit L23	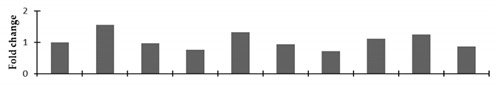									
Auxin repressed 12.5 kDa protein	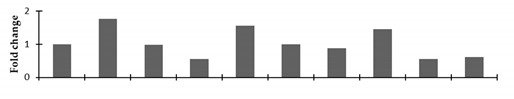									
DNA-(apurinic or apyrimidinic site) lyase	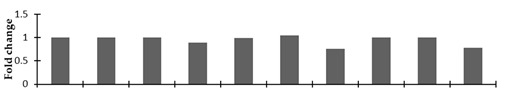									
1-aminocyclopropane-1-carboxylate oxidase 2	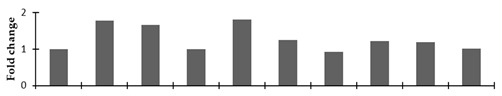									
L-ascorbate peroxidase (APX1)	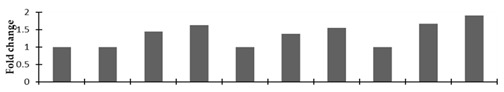									
Manganese superoxide dismutase (MSD1)	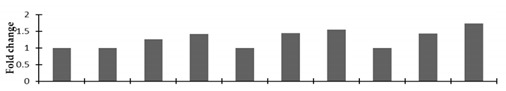									
Glyceraldehyde 3- phosphate dehydrogenase A	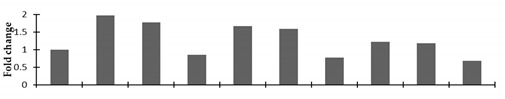									
Tubulin alpha-6 chain (TUA6)	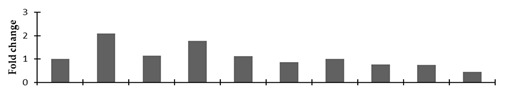									
Eukaryotic translation initiation factor 3 subunit 2 (eIF3I1/TRIP-1)	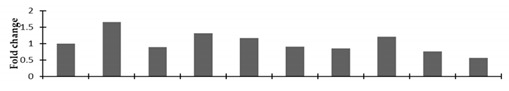									
Allene oxide cyclase 3	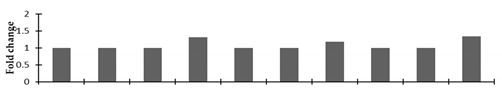									
Deoxyuridine 5′-triphosphate nucleotidol hydrolase	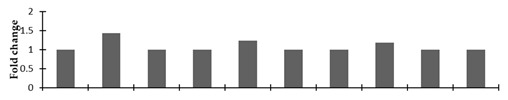									
GTP-binding nuclear protein Ran-1	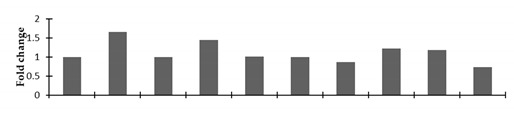									
Polycomb group protein FIE1	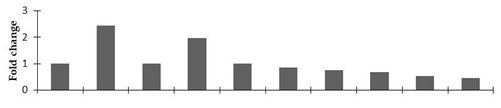									
Transcription factor PRE1	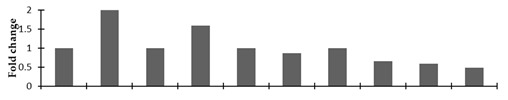									
Geranyl diphosphate synthase	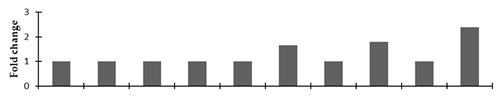									

The protein-expression level in the mutant lines is expressed against control value that is considered as 1.

## Supplementary Materials

The following are available online at https://www.mdpi.com/2223-7747/8/9/321/s1, Table S1: List of phenotypic categories and mutant characteristics, Table S2: Macro-mutant lines of *Nigella sativa* L., Table S3: List of primers for real time PCR, Figure S1: 2-D gel patterns of leaf proteins of *N. sativa* using IPG strips with different pH ranges, Figure S2: MASCOT search results.

## Abbreviations

EMS	Ethyl methyl sulfonate
2DE	Two dimensional gel electrophoresis
MALDI-TOF	Matrix assisted laser desorption/ionization-time of flight
CBB	Coomassie Brilliant Blue
RT-PCR	Real-time PCR
