# Supplementary material for: Proteome Profiling of the Mutagen-Induced Morphological and Yield Macro-Mutant Lines of Nigella sativa L
Source: Plants (Basel). 2019 Sep 2;8(9):321. doi: 10.3390/plants8090321 (PMC6784210; doi:10.3390/plants8090321)
Supplement: Supplementary file 1 [file plants-08-00321-s001.pdf]

Table S1. List of phenotypic categories and mutant characteristics (M_2 generation)

Class	Sub-class		Mutant code	No. of mutant plants
**Plant growth (186)**	Plant height (114)	Tall plants (60-70 cm)	A	51
		Semi-dwarf (40-50 cm)	B	40
		Dwarf (<40 cm)	C	23
	Branching pattern (72)	Unbranched	D	12
		Profused branches	E	18
		Few branches	F	26
		Branches from bottom	G	9
		One-sided branches	H	7
**Leaf (115)**	Colour (86)	Dark green	I	16
		Pale green	J	30
		Yellow	K	32
		Ivory	L	8
	Shape (29)	Bi and tri lobed pinnae	M	10
		Feathery	N	5
		Thin small hairy	O	8
		Upward folded	P	4
		Twisted with short petiole	Q	2
**Flower (161)**		No flower	R	5
	Colour (25)	Yellow-coloured	S	15
		Pink-coloured	T	10
	Petaloid sepal (19)	Narrow-long twisted	U	6
		Ovate with mucronate-like apex	V	5
		Fused	W	8
	Stamen (64)	Black anther type	X	18
		Multi-colour anther type	Y	16
		Brown colour anther type	Z	10
		Long filament type	AB	20
	Gynoecium (48)	Bicarpellary	AC	6
		Tricarpellary	AD	10
		Tetracarpellary	AE	4
		Multicarpellary	AF	19
		Long-twisted style type	AG	4

		Short style type	AH	5
**Capsules (54)**	Locular number	2	AI	8
		3	AJ	20
		4	AK	10
		more than 5	AL	16
**Seed (114)**	Size (42)	Large	AM	16
		Minute	AN	26
	Colour (46)	White	AO	24
		Bicolour	AP	11
		Rust	AQ	8
		Yellow	AR	3
	Shape (26)	Round	AS	12
		Thin elongated	AT	7
		Boat	AU	3
		Beaked	AV	4
	**Total**			**630**

Table S2. Macro-mutant lines of *Nigella sativa* L.

**S.N.**	**Mutant lines**	**Characteristic features** (codes of characters are as per table S1)
1	HY1	High yielding (HY), tall (A), profused branches (E), more than 5 locules in capsule (AL), large-sized seeds (AM), 1011 µg/g thymoquinone content
2	HY3	High yielding (HY), Semi-dwarf (B), branches from bottom (G), pale green leaves (J), round seeds (AS), 1072 µg/g thymoquinone content
3	MHY2	Moderately high yielding (MHY), tall (A), yellow leaves (K), beaked seeds (AV), 1022 µg/g thymoquinone content
4	MHY4	Moderately high yielding (MHY), Semi-dwarf (B), bi-tri-lobed pinnae (M), narrow-long twisted petaloid sepals (U), thin-elongated seeds (AT), 1089 µg/g thymoquinone content
5	NY3	Normal yield (NY), dwarf (C), few branches (F), white coloured seeds (AO), 1040 µg/g thymoquinone content
6	LY1	Low yield (LY), semi-dwarf (B), feathery leaves (N), multicoloured anthers (Y), minute (AN), white seeds (AO), 1028 µg/g thymoquinone content
7	LY3	Low yield (LY), dwarf (C), tricarpellary (AD), trilocular (AJ), yellow coloured seeds (AR), 1123 µg/g thymoquinone content
8	VLY2	Very low yield (VLY), dwarf (C), bilocular capsules (AI), minute seeds (AN), 1093 µg/g thymoquinone content
9	VLY3	Very low yield (VLY), dwarf (C), thin-small hairy (O), brown anthers (Z), boat shaped seed (AU), 1218 µg/g thymoquinone content

Table S3. List of primers for real time PCR

Gene name	Forward primer	Reverse primer
Ribulose bisphosphate carboxylase large chain	CTGCAGGTACATGCGAAGAA	TTGCTAATACCCGGAAGTGG
Ribulose bisphosphate carboxylase/ oxygenase activase	TCGTTGAGAGCCTTGGAGTT	CTGAGGTAGGTCTCGGCAAG
ATP synthase gamma chain, chloroplastic	CCCCTGTTCACTGTGGTCTT	TGAGGGGAACATCAATGTCA
Oxygen evolving enhancer protein 1, chloroplastic	CAACAGCCTCATGAAGACGA	CTTTGGCTTCCCAAACACAT
PSII stability/assembly factor HCF 136, chloroplastic	CCGTGACAAAGCTGCTGATA	GTTGCCAAGCACAAATCCTT
Imidazoleglycerol-phosphate dehydratase 1	CGTTTTGCCAGCTTCTTTTC	ACGCTCACCAAGAGCCTTTA
Malate dehydrogenase 1	CAAGGACGATGCATGGTATG	GTCACGGATGTGGTCACAAG
Sucrose phosphate synthase 1	TGTGGATGTTTCATGCAGGT	GTTTGCCACCAATAGCAGGT
50S ribosomal protein subunit L23	CGGTTATTGGGGAAAAATCA	AATAACCCGGTTGAAGCGTA
Auxin repressed 12.5 kDa protein	TAGCAAGACGATGGGAAACC	AACCAACAACGTGGCATACA
DNA-(apurinic or apyrimidinic) lyase	ACAATGAGACCTCCCCCTCT	TCCTGCAAGCACAAGATGTC
1-aminocyclo propane-1-carboxylate oxidase 2	AAGGTCTTAGGGCCCACACT	CAACCGACATCCTGTTTCCT
L-ascorbate peroxidase (APX1)	GCATGGACATCAAACCCTCT	AGCAAACCCAAGCTCAGAAA
Manganese superoxide dismutase (MSD1)	CCTTACGATTATGGCGCATT	CTTCACTGGAAGGAGCAAGG
Glycerdehyde 3- phosphate dehydrogenase	CGGTCAATGGAAACACAATG	AACAACCTTCTTGGCACCAC
Tubulin alpha-6 chain (TUA6)	AATGCTTGCTGGGAGCTTTA	CGATCACAGTTGGCTCAAGA
Eukaryotic translation initiation factor 3 subunit 2(eIF3I1/TRIP-1)	TGGAACTTACCGTGGTCACA	CACTGCAAGACGATCTCCAA
Allene oxide cyclase 3	GACCGAAAACTCCAGACCAA	TTCAGGAACGTGTTGGATCA
Deoxyuridine 5′-triphosphate nucleotidol hydrolase	CTGGTTTGGCTTGGAAACAT	CACCATCACCACGAACAGTC
GTP-binding nuclear protein Ran-1	TTACTGCTGGGACACTGCTG	CACACAGAACAATCGGGATG
Polycomb group protein FIE1	AAGGTGAACCACAGGAAACG	TTCCCTCAGTGTGCTTGTTG
Transcription factor PRE1	TGCTCCAAGGATCTCCGATA	AGACAAACGCTCGCTCAGAT
Geranyl diphosphate synthase	GAATTGCGTACGAGACAGCA	GCAAGCAAGCTAACGACCTC

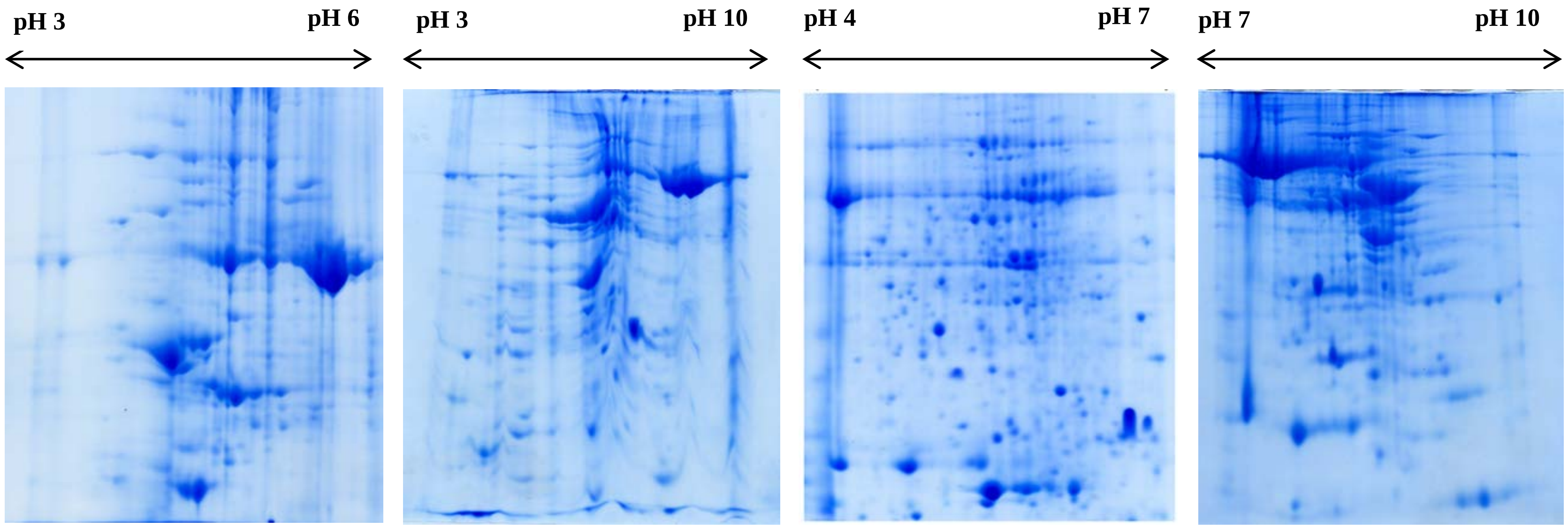

Supplementary figure 1. 2-D gel patterns of leaf proteins of *N. sativa* using IPG strips with different pH ranges.

MATRIX SCIENCE MASCOT Search Results

Protein View: RBL_ERYCG

Ribulose bisphosphate carboxylase large chain (Fragment) OS=Erythrina crista-galli OX=49817 GN=rbcL PE=3 SV=1

**Database:**	SwissProt
**Score:**	148
**Expect:**	6.3e-11
**Monoisotopic mass (M_r):**	50384
**Calculated pI:**	6.22
**Taxonomy:**	Erythrina crista-galli

Sequence similarity is available as an NCBI BLAST search of RBL_ERYCG against nr.

Search parameters

**Enzyme:**	Trypsin: cuts C-term side of KR unless next residue is P.
**Mass values searched:**	25
**Mass values matched:**	15

Protein sequence coverage: 65%

Matched peptides shown in ***bold red***.

```
  1 SVGFKAGVKD YKLTYYTPEY ETKDTDILAA FRVTPQPGVP PEEAGAAVAA
 51 ESSTGTWTTV WTDGLTSLDR YKGRCYHIEP VAGEENQYIA YVAYPLDLFE
101 EGSVTNMFTS IVGNVFGFKA LRALRLEDFR IPTAYVKTFQ GPPHGIQVER
151 DKLNKYGRPL LGCTIKPKLG LSAKNYGRAV YECLRGGLDF TKDDENVNSQ
201 PFMRWRDRFL FCAEALYKAQ AETGEIKGHY LNATAGTCEE MIKRAVFARE
251 LGVPIIMHDY LTGGFTANTS LAHYCRDNGL LLHIHRAMHA VIDRQKNHGM
301 HFRVLAKALR LSGGDHVHSG TVVGKLEGER EITLGFVDLI RDDLIEKDRS
351 RGIYFTQDWV SLPGVLPVAS GGIHVWHMPA LTEIFGDDSV LQFGGGTLGH
401 PWGNAPGAVA NRVALEACVQ ARNEGRDLAR EGNEIIREAS KWSPELAAAC
451 EVWKE
```

Unformatted sequence string: 455 residues (for pasting into other applications).

Sort by ◉ residue number ○ increasing mass ○ decreasing mass
Show ◉ matched peptides only ○ predicted peptides also

Start – End	Observed	Mr(expt)	Mr(calc)	Delta	M	Peptide
13 – 23	1407.6678	1406.6605	1406.6605	-0.0000	0	K.LTYYTPEYETK.D
33 – 70	3854.8718	3853.8645	3853.8647	-0.0002	0	R.VTPQPGVPPEEAGAAVAAESSTGTWTTVWTDGLTSLDR.Y
71 – 72	311.0000	309.9927	309.1688	0.8239	0	R.YK.G
75 – 119	5061.4001	5060.3928	5060.3929	-0.0000	0	R.CYHIEPVAGEENQYIAYVAYPLDLFEEGSVTNMFTSIVGNVFGFK.A
138 – 150	1465.7546	1464.7473	1464.7474	-0.0001	0	K.TFQGPPHGIQVER.D
156 – 168	1445.8297	1444.8224	1444.8224	0.0000	0	K.YGRPLLGCTIKPK.L
193 – 204	1451.6219	1450.6146	1450.6147	-0.0001	0	K.DDENVNSQPFMR.W
209 – 218	1204.6071	1203.5998	1203.5998	0.0000	0	R.FLFCAEALYK.A
228 – 243	1737.7934	1736.7861	1736.7862	-0.0000	0	K.GHYLNATAGTCEEMIK.R
250 – 276	2979.4491	2978.4418	2978.4419	-0.0001	0	R.ELGVPIIMHDYLTGGFTANTSLAHYCR.D
277 – 286	1187.6643	1186.6570	1186.6571	-0.0001	0	R.DNGLLLHIHR.A
311 – 325	1449.7444	1448.7371	1448.7372	-0.0001	0	R.LSGGDHVHSGTVVGK.L
331 – 341	1275.7307	1274.7234	1274.7234	-0.0000	0	R.EITLGFVDLIR.D
352 – 412	6369.1766	6368.1693	6368.1695	-0.0002	0	R.GIYFTQDWVSLPGVLPVASGGIHVWHMPALTEIFGDDSVLQFGGGTLGHPWGNAPGAVANR.V
442 – 454	1489.7144	1488.7071	1488.7071	0.0000	0	K.WSPELAAACEVWK.E

No match to: 138.0000, 156.0000, 193.0000, 209.0000, 228.0000, 250.0000, 277.0000, 331.0000, 352.0000, 442.0000

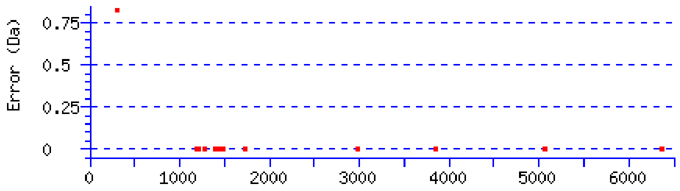

RMS error 686 ppm

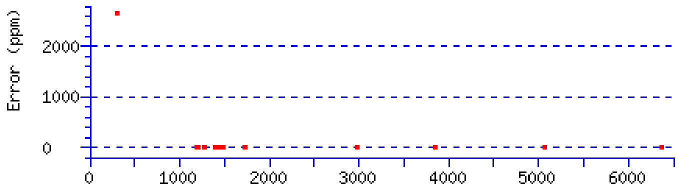

RMS error 686 ppm

```
ID   RBL_ERYCG               Reviewed;         455 AA.
AC   Q33438;
DT   02-NOV-2001, integrated into UniProtKB/Swiss-Prot.
DT   01-NOV-1996, sequence version 1.
DT   31-JUL-2019, entry version 92.
DE   RecName: Full=Ribulose bisphosphate carboxylase large chain {ECO:0000255|HAMAP-Rule:MF_01338};
DE            Short=RuBisCO large subunit {ECO:0000255|HAMAP-Rule:MF_01338};
DE            EC=4.1.1.39 {ECO:0000255|HAMAP-Rule:MF_01338};
DE   Flags: Fragment;
```

```
GN   Name=rbcL {ECO:0000255|HAMAP-Rule:MF_01338};
OS   Erythrina crista-galli (Cockspur coral tree) (Micropteryx
OS   crista-galli).
OG   Plastid; Chloroplast.
OC   Eukaryota; Viridiplantae; Streptophyta; Embryophyta; Tracheophyta;
OC   Spermatophyta; Magnoliopsida; eudicotyledons; Gunneridae;
OC   Pentapetalae; rosids; fabids; Fabales; Fabaceae; Papilionoideae;
OC   50 kb inversion clade; NPAAA clade; indigoferoid/millettioid clade;
OC   Phaseoleae; Erythrina.
OX   NCBI_TaxID=49817;
RN   [1]
RP   NUCLEOTIDE SEQUENCE [GENOMIC DNA].
RC   TISSUE=Leaf;
RA   Kaess E., Wink M.;
RT   "Molecular phylogeny of the Papilionoideae (family Leguminosae): rbcL
RT   sequences versus chemical taxonomy.";
RL   Bot. Acta 108:149-162(1995).
CC   -!- FUNCTION: RuBisCO catalyzes two reactions: the carboxylation of D-
CC       ribulose 1,5-bisphosphate, the primary event in carbon dioxide
CC       fixation, as well as the oxidative fragmentation of the pentose
CC       substrate in the photorespiration process. Both reactions occur
CC       simultaneously and in competition at the same active site.
CC       {ECO:0000255|HAMAP-Rule:MF_01338}.
CC   -!- CATALYTIC ACTIVITY:
CC       Reaction=2 3-phospho-D-glycerate + 2 H(+) = CO2 + D-ribulose 1,5-
CC         bisphosphate + H2O; Xref=Rhea:RHEA:23124, ChEBI:CHEBI:15377,
CC         ChEBI:CHEBI:15378, ChEBI:CHEBI:16526, ChEBI:CHEBI:57870,
CC         ChEBI:CHEBI:58272; EC=4.1.1.39; Evidence={ECO:0000255|HAMAP-
CC         Rule:MF_01338};
CC   -!- CATALYTIC ACTIVITY:
CC       Reaction=D-ribulose 1,5-bisphosphate + O2 = 2-phosphoglycolate +
CC         3-phospho-D-glycerate + 2 H(+); Xref=Rhea:RHEA:36631,
CC         ChEBI:CHEBI:15378, ChEBI:CHEBI:15379, ChEBI:CHEBI:57870,
CC         ChEBI:CHEBI:58033, ChEBI:CHEBI:58272;
CC         Evidence={ECO:0000255|HAMAP-Rule:MF_01338};
CC   -!- COFACTOR:
CC       Name=Mg(2+); Xref=ChEBI:CHEBI:18420;
CC         Evidence={ECO:0000255|HAMAP-Rule:MF_01338};
CC       Note=Binds 1 Mg(2+) ion per subunit. {ECO:0000255|HAMAP-
CC       Rule:MF_01338};
CC   -!- SUBUNIT: Heterohexadecamer of 8 large chains and 8 small chains;
CC       disulfide-linked. The disulfide link is formed within the large
CC       subunit homodimers. {ECO:0000255|HAMAP-Rule:MF_01338}.
CC   -!- SUBCELLULAR LOCATION: Plastid, chloroplast.
CC   -!- PTM: The disulfide bond which can form in the large chain dimeric
CC       partners within the hexadecamer appears to be associated with
CC       oxidative stress and protein turnover. {ECO:0000255|HAMAP-
CC       Rule:MF_01338}.
CC   -!- MISCELLANEOUS: The basic functional RuBisCO is composed of a large
CC       chain homodimer in a "head-to-tail" conformation. In form I
CC       RuBisCO this homodimer is arranged in a barrel-like tetramer with
CC       the small subunits forming a tetrameric "cap" on each end of the
CC       "barrel". {ECO:0000255|HAMAP-Rule:MF_01338}.
CC   -!- SIMILARITY: Belongs to the RuBisCO large chain family. Type I
CC       subfamily. {ECO:0000255|HAMAP-Rule:MF_01338}.
DR   EMBL; Z70170; CAA94028.1; -; Genomic_DNA.
DR   SMR; Q33438; -.
DR   PRIDE; Q33438; -.
DR   GO; GO:0009507; C:chloroplast; IEA:UniProtKB-SubCell.
DR   GO; GO:0000287; F:magnesium ion binding; IEA:InterPro.
DR   GO; GO:0004497; F:monooxygenase activity; IEA:UniProtKB-KW.
DR   GO; GO:0016984; F:ribulose-bisphosphate carboxylase activity; IEA:UniProtKB-EC.
DR   GO; GO:0009853; P:photorespiration; IEA:UniProtKB-KW.
DR   GO; GO:0019253; P:reductive pentose-phosphate cycle; IEA:UniProtKB-KW.
DR   CDD; cd08212; RuBisCO_large_I; 1.
DR   Gene3D; 3.20.20.110; -; 1.
DR   Gene3D; 3.30.70.150; -; 1.
DR   HAMAP; MF_01338; RuBisCO_L_type1; 1.
DR   InterPro; IPR033966; RuBisCO.
DR   InterPro; IPR020878; RuBisCo_large_chain_AS.
DR   InterPro; IPR000685; RuBisCO_lsu_C.
DR   InterPro; IPR036376; RuBisCO_lsu_C_sf.
DR   InterPro; IPR017443; RuBisCO_lsu_fd_N.
DR   InterPro; IPR036422; RuBisCO_lsu_N_sf.
DR   InterPro; IPR020888; RuBisCO_lsuI.
DR   PANTHER; PTHR42704; PTHR42704; 1.
DR   Pfam; PF00016; RuBisCO_large; 1.
DR   Pfam; PF02788; RuBisCO_large_N; 1.
DR   SFLD; SFLDS00014; RuBisCO; 1.
DR   SUPFAM; SSF51649; SSF51649; 1.
DR   SUPFAM; SSF54966; SSF54966; 1.
DR   PROSITE; PS00157; RUBISCO_LARGE; 1.
PE   3: Inferred from homology;
KW   Calvin cycle; Carbon dioxide fixation; Chloroplast; Disulfide bond;
KW   Lyase; Magnesium; Metal-binding; Methylation; Monooxygenase;
KW   Oxidoreductase; Photorespiration; Photosynthesis; Plastid.
FT   CHAIN        <1   >455       Ribulose bisphosphate carboxylase large
FT                                chain.
FT                                /FTId=PRO_0000062462.
FT   ACT_SITE    166    166       Proton acceptor. {ECO:0000255|HAMAP-
FT                                Rule:MF_01338}.
FT   ACT_SITE    285    285       Proton acceptor. {ECO:0000255|HAMAP-
FT                                Rule:MF_01338}.
FT   METAL       192    192       Magnesium; via carbamate group.
FT                                {ECO:0000255|HAMAP-Rule:MF_01338}.
FT   METAL       194    194       Magnesium. {ECO:0000255|HAMAP-
```

```
FT                                   Rule:MF_01338}.
FT   METAL       195    195       Magnesium. {ECO:0000255|HAMAP-
FT                                   Rule:MF_01338}.
FT   BINDING     114    114       Substrate; in homodimeric partner.
FT                                   {ECO:0000255|HAMAP-Rule:MF_01338}.
FT   BINDING     164    164       Substrate. {ECO:0000255|HAMAP-
FT                                   Rule:MF_01338}.
FT   BINDING     168    168       Substrate. {ECO:0000255|HAMAP-
FT                                   Rule:MF_01338}.
FT   BINDING     286    286       Substrate. {ECO:0000255|HAMAP-
FT                                   Rule:MF_01338}.
FT   BINDING     318    318       Substrate. {ECO:0000255|HAMAP-
FT                                   Rule:MF_01338}.
FT   BINDING     370    370       Substrate. {ECO:0000255|HAMAP-
FT                                   Rule:MF_01338}.
FT   SITE        325    325       Transition state stabilizer.
FT                                   {ECO:0000255|HAMAP-Rule:MF_01338}.
FT   MOD_RES       5      5       N6,N6,N6-trimethyllysine.
FT                                   {ECO:0000255|HAMAP-Rule:MF_01338}.
FT   MOD_RES     192    192       N6-carboxylysine. {ECO:0000255|HAMAP-
FT                                   Rule:MF_01338}.
FT   DISULFID    238    238       Interchain; in linked form.
FT                                   {ECO:0000255|HAMAP-Rule:MF_01338}.
FT   NON_TER       1      1
FT   NON_TER     455    455
SQ   SEQUENCE   455 AA;  50416 MW;  6C240343BCDA4029 CRC64;
     SVGFKAGVKD YKLTYYTPEY ETKDTDILAA FRVTPQPGVP PEEAGAAVAA ESSTGTWTTV
     WTDGLTSLDR YKGRCYHIEP VAGEENQYIA YVAYPLDLFE EGSVTNMFTS IVGNVFGFKA
     LRALRLEDFR IPTAYVKTFQ GPPHGIQVER DKLNKYGRPL LGCTIKPKLG LSAKNYGRAV
     YECLRGGLDF TKDDENVNSQ PFMRWRDRFL FCAEALYKAQ AETGEIKGHY LNATAGTCEE
     MIKRAVFARE LGVPIIMHDY LTGGFTANTS LAHYCRDNGL LLHIHRAMHA VIDRQKNHGM
     HFRVLAKALR LSGGDHVHSG TVVGKLEGER EITLGFVDLI RDDLIEKDRS RGIYFTQDWV
     SLPGVLPVAS GGIHVWHMPA LTEIFGDDSV LQFGGGTLGH PWGNAPGAVA NRVALEACVQ
     ARNEGRDLAR EGNEIIREAS KWSPELAAAC EVWKE
```

Mascot: http://www.matrixscience.com/

MATRIX SCIENCE

MASCOT Search Results

Protein View: RCA_MAIZE

Ribulose bisphosphate carboxylase/oxygenase activase, chloroplastic OS=Zea mays OX=4577 GN=RCA1 PE=2 SV=3

**Database:**	SwissProt
**Score:**	119
**Expect:**	5e-08
**Monoisotopic mass (M_r):**	47908
**Calculated pI:**	6.29
**Taxonomy:**	Zea mays

Sequence similarity is available as an NCBI BLAST search of RCA_MAIZE against nr.

Search parameters

**Enzyme:**	Trypsin: cuts C-term side of KR unless next residue is P.
**Mass values searched:**	20
**Mass values matched:**	12

Protein sequence coverage: 53%

Matched peptides shown in ***bold red***.

1 MAAAFSSTVG APASTPTRSS FLGKKLNKPQ VSAAVTYHGK SSSSNSRFKA
51 MAAKEVDETK QTDEDRWK**GL** **AYDISDDQQD** **ITR**GK**GLVDN** **LFQAPMGDGT**
101 **HVAVLSSYDY** **ISQGQKSYNF** **DNMMDGFYIA** **K**GFMDKLVVH LSKNFMTLPN
151 IKVPLILGIW GGK**GQGK**SFQ CELVFAK**MGI** **TPIMMSAGEL** **ESGNAGEPAK**
201 LIRQRYREAS DLIKKGK**MSC** **LFINDLDAGA** **GRMGGTTQYT** **VNNQMVNATL**
251 **MNIADNPTNV** **QLPGMYNK**ED NPR**VPIIVTG** **NDFSTLYAPL** **IR**DGRMEKFY
301 WAPTREDRIG VCKGIFR**TDG** **VDEEHVVQLV** **DTFPGQSIDF** **FGALR**ARVYD
351 DEVRR**WVSET** **GVENIAR**KLV NSKEGPPTFE QPKITIEK**LL** **EYGHMLVAEQ**
401 **ENVK**RVQLAD K**YLNEAALGE** **ANEDAMK**TGS FFK

Unformatted sequence string: 433 residues (for pasting into other applications).

Sort by ◉ residue number ○ increasing mass ○ decreasing mass
Show ◉ matched peptides only ○ predicted peptides also

Start – End	Observed	Mr(expt)	Mr(calc)	Delta	M	Peptide
69 – 83	1709.7976	1708.7903	1708.7904	-0.0001	0	K.GLAYDISDDQQDITR.G
86 – 116	3310.6048	3309.5975	3309.5976	-0.0001	0	K.GLVDNLFQAPMGDGTHVAVLSSYDYISQGQK.S
117 – 131	1815.7716	1814.7643	1814.7644	-0.0001	0	K.SYNFDNMMDGFYIAK.G
164 – 167	389.0000	387.9927	388.2070	-0.2143	0	K.GQGK.S
178 – 200	2291.0716	2290.0643	2290.0643	0.0000	0	K.MGITPIMMSAGELESGNAGEPAK.L
218 – 232	1582.7352	1581.7279	1581.7279	-0.0000	0	K.MSCLFINDLDAGAGR.M
233 – 268	3943.8445	3942.8372	3942.8373	-0.0000	0	R.MGGTTQYTVNNQMVNATLMNIADNPTNVQLPGMYNK.E
274 – 292	2089.1691	2088.1618	2088.1619	-0.0001	0	R.VPIIVTGNDFSTLYAPLIR.D
318 – 345	3091.5006	3090.4933	3090.4935	-0.0002	0	R.TDGVDEEHVVQLVDTFPGQSIDFFGALR.A
356 – 367	1360.6855	1359.6782	1359.6783	-0.0000	0	R.WVSETGVENIAR.K
389 – 404	1872.9524	1871.9451	1871.9451	0.0000	0	K.LLEYGHMLVAEQENVK.R
412 – 427	1738.7952	1737.7879	1737.7879	0.0000	0	K.YLNEAALGEANEDAMK.T

No match to: 117.0000, 178.0000, 218.0000, 233.0000, 274.0000, 318.0000, 356.0000, 412.0000

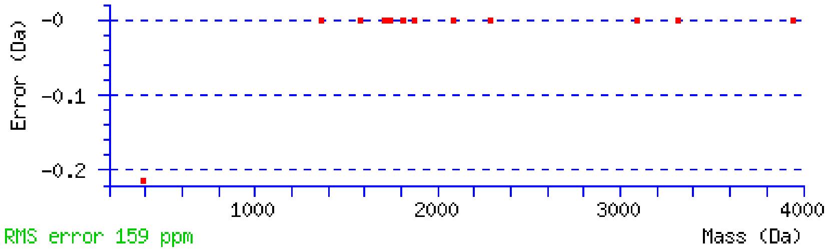

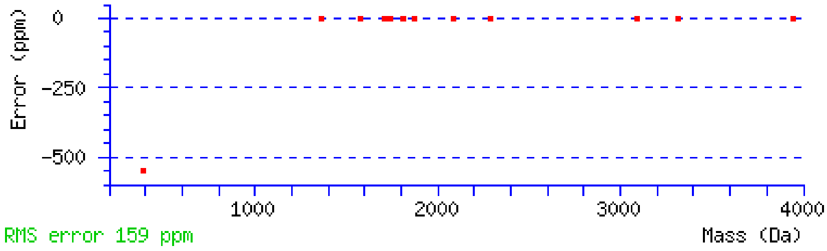

```
ID   RCA_MAIZE               Reviewed;         433 AA.
AC   Q9ZT00;
DT   01-DEC-2000, integrated into UniProtKB/Swiss-Prot.
DT   28-MAR-2003, sequence version 3.
DT   31-JUL-2019, entry version 99.
DE   RecName: Full=Ribulose bisphosphate carboxylase/oxygenase activase, chloroplastic;
DE            Short=RA;
DE            Short=RuBisCO activase;
DE   Flags: Precursor;
GN   Name=RCA1; Synonyms=RCA;
OS   Zea mays (Maize).
OC   Eukaryota; Viridiplantae; Streptophyta; Embryophyta; Tracheophyta;
OC   Spermatophyta; Magnoliopsida; Liliopsida; Poales; Poaceae;
OC   PACMAD clade; Panicoideae; Andropogonodae; Andropogoneae; Tripsacinae;
OC   Zea.
OX   NCBI_TaxID=4577;
RN   [1]
RP   NUCLEOTIDE SEQUENCE [MRNA].
RC   STRAIN=cv. Chalqueno; TISSUE=Leaf;
RA   Ayala-Ochoa A., Loza-Tavera H., Sanchez de Jimenez E.;
RT   "A cDNA from maize (Zea mays L) encoding ribulose-1,5-bisphosphate
RT   carboxylase/oxygenase activase.";
RL   (er) Plant Gene Register PGR98-207(1998).
RN   [2]
RP   SEQUENCE REVISION TO 57-68; 160; 219 AND 347.
RA   Ayala-Ochoa A., Loza-Tavera H., Sanchez de Jimenez E.;
RL   Submitted (APR-2002) to the EMBL/GenBank/DDBJ databases.
CC   -!- FUNCTION: Activation of RuBisCO (ribulose-1,5-bisphosphate
CC       carboxylase/oxygenase; EC 4.1.1.39) involves the ATP-dependent
CC       carboxylation of the epsilon-amino group of lysine leading to a
CC       carbamate structure.
CC   -!- SUBCELLULAR LOCATION: Plastid, chloroplast stroma.
CC   -!- SIMILARITY: Belongs to the RuBisCO activase family. {ECO:0000305}.
DR   EMBL; AF084478; AAC97932.3; -; mRNA.
DR   RefSeq; NP_001104921.1; NM_001111451.2.
DR   SMR; Q9ZT00; -.
DR   STRING; 4577.GRMZM2G162200_P01; -.
DR   PaxDb; Q9ZT00; -.
DR   PRIDE; Q9ZT00; -.
DR   EnsemblPlants; Zm00001d048593_T001; Zm00001d048593_P001; Zm00001d048593.
DR   GeneID; 541712; -.
DR   Gramene; Zm00001d048593_T001; Zm00001d048593_P001; Zm00001d048593.
DR   KEGG; zma:541712; -.
DR   MaizeGDB; 114858; -.
DR   eggNOG; KOG0651; Eukaryota.
DR   eggNOG; COG1222; LUCA.
DR   HOGENOM; HOG000243931; -.
DR   OMA; RFAQNNK; -.
DR   OrthoDB; 655049at2759; -.
DR   Proteomes; UP000007305; Chromosome 4.
DR   ExpressionAtlas; Q9ZT00; baseline and differential.
DR   Genevisible; Q9ZT00; ZM.
DR   GO; GO:0009570; C:chloroplast stroma; IBA:GO_Central.
DR   GO; GO:0009579; C:thylakoid; IBA:GO_Central.
DR   GO; GO:0005524; F:ATP binding; IEA:UniProtKB-KW.
DR   GO; GO:0046863; F:ribulose-1,5-bisphosphate carboxylase/oxygenase activator activity; IBA:GO_Central.
DR   InterPro; IPR003959; ATPase_AAA_core.
DR   InterPro; IPR027417; P-loop_NTPase.
```

```
DR   Pfam; PF00004; AAA; 1.
DR   SUPFAM; SSF52540; SSF52540; 1.
PE   2: Evidence at transcript level;
KW   ATP-binding; Chloroplast; Complete proteome; Nucleotide-binding;
KW   Plastid; Reference proteome; Transit peptide.
FT   TRANSIT       1     53       Chloroplast. {ECO:0000255}.
FT   CHAIN        54    433       Ribulose bisphosphate
FT                                carboxylase/oxygenase activase,
FT                                chloroplastic.
FT                                /FTId=PRO_0000030236.
FT   NP_BIND     161    168       ATP. {ECO:0000255}.
SQ   SEQUENCE   433 AA;  47938 MW;  5730A336E6D378D2 CRC64;
     MAAAFSSTVG APASTPTRSS FLGKKLNKPQ VSAAVTYHGK SSSSNSRFKA MAAKEVDETK
     QTDEDRWKGL AYDISDDQQD ITRGKGLVDN LFQAPMGDGT HVAVLSSYDY ISQGQKSYNF
     DNMMDGFYIA KGFMDKLVVH LSKNFMTLPN IKVPLILGIW GGKGQGKSFQ CELVFAKMGI
     TPIMMSAGEL ESGNAGEPAK LIRQRYREAS DLIKKGKMSC LFINDLDAGA GRMGGTTQYT
     VNNQMVNATL MNIADNPTNV QLPGMYNKED NPRVPIIVTG NDFSTLYAPL IRDGRMEKFY
     WAPTREDRIG VCKGIFRTDG VDEEHVVQLV DTFPGQSIDF FGALRARVYD DEVRRWVSET
     GVENIARKLV NSKEGPPTFE QPKITIEKLL EYGHMLVAEQ ENVKRVQLAD KYLNEAALGE
     ANEDAMKTGS FFK
```

Mascot: http://www.matrixscience.com/

MATRIX SCIENCE MASCOT Search Results

Protein View: ATPG_TOBAC

ATP synthase gamma chain, chloroplastic OS=Nicotiana tabacum OX=4097 GN=ATPC PE=1 SV=1

**Database:**	SwissProt
**Score:**	90
**Expect:**	3.6e-05
**Monoisotopic mass (M_r):**	41421
**Calculated pI:**	8.16
**Taxonomy:**	Nicotiana tabacum

Sequence similarity is available as an NCBI BLAST search of ATPG_TOBAC against nr.

Search parameters

**Enzyme:**	Trypsin: cuts C-term side of KR unless next residue is P.
**Mass values searched:**	11
**Mass values matched:**	8

Protein sequence coverage: 38%

Matched peptides shown in ***bold red***.

```
  1 MSCSNLTMLV SSKPSLSDSS ALSFRSSVSP FQLPNHNTSG PSNPSRSSSV
 51 TPVHCGLRDL RDRIESVKNT QKITEAMKLV AAAKVRRAQE AVVGARPFSE
101 TLVEVLYNIN EQLQTDDIDV PLTKVRPVKK VALVVVTGDR GLCGGFNNYL
151 IKKAEARIRD LKALGIDYTI ISVGKKGNSY FIRRPYIPVD KFLEGSNLPT
201 AKDAQAIADD VFSLFVSEEV DKVELLYTKF VSLVKSEPVI HTLLPLSPKG
251 EICDINGNCV DAANDEFFRL TTKEGKLTVE RDIIRTKTTD FSPILQFEQD
301 PVQILDALLP LYLNSQILRA LQESLASELA ARMSAMSSAT DNATELKKNL
351 SRVYNRQRQA KITGEILEIV AGADALV
```

Unformatted sequence string: 377 residues (for pasting into other applications).

Sort by ◉ residue number ○ increasing mass ○ decreasing mass
Show ◉ matched peptides only ○ predicted peptides also

Start – End	Observed	Mr(expt)	Mr(calc)	Delta	M	Peptide
88 – 124	4115.1182	4114.1109	4114.1110	-0.0001	0	R.AQEAVVGARPFSETLVEVLYNINEQLQTDDIDVPLTK.V
158 – 159	288.0000	286.9927	287.1957	-0.2030	0	R.IR.D
203 – 222	2198.0499	2197.0426	2197.0427	-0.0000	0	K.DAQAIADDVFSLFVSEEVDK.V
250 – 269	2201.9226	2200.9153	2200.9154	-0.0000	0	K.GEICDINGNCVDAANDEFFR.L
274 – 276	333.0000	331.9927	332.1696	-0.1768	0	K.EGK.L
288 – 319	3700.9836	3699.9763	3699.9763	-0.0000	0	K.TTDFSPILQFEQDPVQILDALLPLYLNSQILR.A
333 – 347	1556.6931	1555.6858	1555.6858	0.0000	0	R.MSAMSSATDNATELK.K
362 – 377	1583.8890	1582.8817	1582.8818	-0.0001	0	K.ITGEILEIVAGADALV.-

No match to: 203.0000, 250.0000, 362.0000

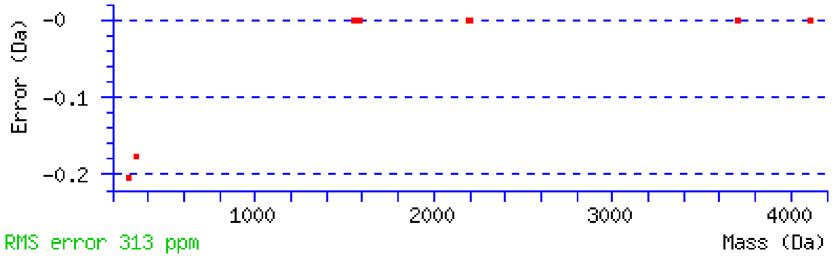

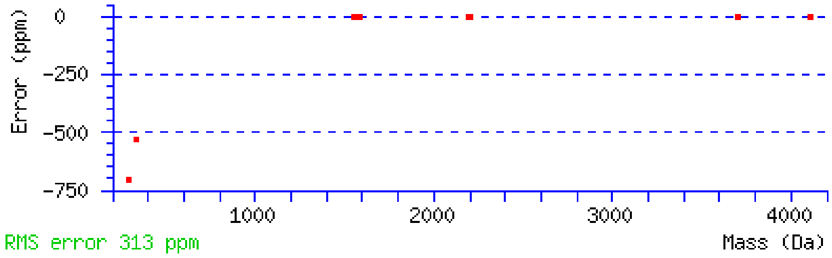

```
ID   ATPG_TOBAC              Reviewed;         377 AA.
AC   P29790;
DT   01-APR-1993, integrated into UniProtKB/Swiss-Prot.
DT   01-APR-1993, sequence version 1.
DT   31-JUL-2019, entry version 94.
DE   RecName: Full=ATP synthase gamma chain, chloroplastic;
DE   AltName: Full=F-ATPase gamma subunit;
DE   Flags: Precursor;
GN   Name=ATPC;
OS   Nicotiana tabacum (Common tobacco).
OC   Eukaryota; Viridiplantae; Streptophyta; Embryophyta; Tracheophyta;
OC   Spermatophyta; Magnoliopsida; eudicotyledons; Gunneridae;
OC   Pentapetalae; asterids; lamiids; Solanales; Solanaceae;
OC   Nicotianoideae; Nicotianeae; Nicotiana.
OX   NCBI_TaxID=4097;
RN   [1]
RP   NUCLEOTIDE SEQUENCE [MRNA], AND PROTEIN SEQUENCE OF 56-60.
RC   TISSUE=Leaf;
RX   PubMed=1535803; DOI=10.1007/BF00027359;
RA   Larsson K.H., Napier J.A., Gray J.C.;
RT   "Import and processing of the precursor form of the gamma subunit of
RT   the chloroplast ATP synthase from tobacco.";
RL   Plant Mol. Biol. 19:343-349(1992).
CC   -!- FUNCTION: Produces ATP from ADP in the presence of a proton
CC       gradient across the membrane. The gamma chain is believed to be
CC       important in regulating ATPase activity and the flow of protons
CC       through the CF(0) complex.
CC   -!- SUBUNIT: F-type ATPases have 2 components, CF(1) - the catalytic
CC       core - and CF(0) - the membrane proton channel. CF(1) has five
CC       subunits: alpha(3), beta(3), gamma(1), delta(1), epsilon(1). CF(0)
CC       has four main subunits: a, b, b' and c (By similarity).
CC       {ECO:0000250}.
CC   -!- SUBCELLULAR LOCATION: Plastid, chloroplast thylakoid membrane
CC       {ECO:0000250}; Peripheral membrane protein {ECO:0000250}.
CC   -!- SIMILARITY: Belongs to the ATPase gamma chain family.
CC       {ECO:0000305}.
DR   EMBL; X63606; CAA45152.1; -; mRNA.
DR   PIR; S22486; PWNTG.
DR   RefSeq; NP_001312843.1; NM_001325914.1.
DR   SMR; P29790; -.
DR   PRIDE; P29790; -.
DR   ProMEX; P29790; -.
DR   GeneID; 107813955; -.
DR   KEGG; nta:107813955; -.
DR   KO; K02115; -.
DR   Proteomes; UP000084051; Genome assembly.
DR   GO; GO:0009535; C:chloroplast thylakoid membrane; IEA:UniProtKB-SubCell.
DR   GO; GO:0045261; C:proton-transporting ATP synthase complex, catalytic core F(1); IEA:UniProtKB-KW.
DR   GO; GO:0046933; F:proton-transporting ATP synthase activity, rotational mechanism; IEA:InterPro.
DR   GO; GO:0015986; P:ATP synthesis coupled proton transport; IEA:InterPro.
DR   CDD; cd12151; F1-ATPase_gamma; 1.
DR   HAMAP; MF_00815; ATP_synth_gamma_bact; 1.
DR   InterPro; IPR035968; ATP_synth_F1_ATPase_gsu.
DR   InterPro; IPR000131; ATP_synth_F1_gsu.
DR   InterPro; IPR023632; ATP_synth_F1_gsu_CS.
DR   PANTHER; PTHR11693; PTHR11693; 1.
```

```
DR   Pfam; PF00231; ATP-synt; 1.
DR   PRINTS; PR00126; ATPASEGAMMA.
DR   SUPFAM; SSF52943; SSF52943; 1.
DR   TIGRFAMs; TIGR01146; ATPsyn_F1gamma; 1.
DR   PROSITE; PS00153; ATPASE_GAMMA; 1.
PE   1: Evidence at protein level;
KW   ATP synthesis; CF(1); Chloroplast; Complete proteome;
KW   Direct protein sequencing; Disulfide bond; Hydrogen ion transport;
KW   Ion transport; Membrane; Plastid; Reference proteome; Thylakoid;
KW   Transit peptide; Transport.
FT   TRANSIT       1     55       Chloroplast.
FT                                {ECO:0000269|PubMed:1535803}.
FT   CHAIN        56    377       ATP synthase gamma chain, chloroplastic.
FT                                /FTId=PRO_0000002681.
FT   ACT_SITE    143    143       {ECO:0000250}.
FT   DISULFID    253    259       {ECO:0000250}.
SQ   SEQUENCE   377 AA;  41447 MW;  60A262F08013F3E0 CRC64;
     MSCSNLTMLV SSKPSLSDSS ALSFRSSVSP FQLPNHNTSG PSNPSRSSSV TPVHCGLRDL
     RDRIESVKNT QKITEAMKLV AAAKVRRAQE AVVGARPFSE TLVEVLYNIN EQLQTDDIDV
     PLTKVRPVKK VALVVVTGDR GLCGGFNNYL IKKAEARIRD LKALGIDYTI ISVGKKGNSY
     FIRRPYIPVD KFLEGSNLPT AKDAQAIADD VFSLFVSEEV DKVELLYTKF VSLVKSEPVI
     HTLLPLSPKG EICDINGNCV DAANDEFFRL TTKEGKLTVE RDIIRTKTTD FSPILQFEQD
     PVQILDALLP LYLNSQILRA LQESLASELA ARMSAMSSAT DNATELKKNL SRVYNRQRQA
     KITGEILEIV AGADALV
```

Mascot: http://www.matrixscience.com/

MATRIX SCIENCE MASCOT Search Results

Protein View: PSBO_SPIOL

Oxygen-evolving enhancer protein 1, chloroplastic OS=Spinacia oleracea OX=3562 GN=PSBO PE=1 SV=1

**Database:**	SwissProt
**Score:**	50
**Expect:**	0.38
**Monoisotopic mass (M_r):**	35149
**Calculated pI:**	5.58
**Taxonomy:**	Spinacia oleracea

Sequence similarity is available as an NCBI BLAST search of PSBO_SPIOL against nr.

Search parameters

**Enzyme:**	Trypsin: cuts C-term side of KR unless next residue is P.
**Mass values searched:**	8
**Mass values matched:**	4

Protein sequence coverage: 30%

Matched peptides shown in ***bold red***.

```
  1 MAASLQASTT FLQPTKVASR NTLQLRSTQN VCKAFGVESA SSGGRLSLSL
 51 QSDLKELANK CVDATKLAGL ALATSALIAS GANAEGGKRL TYDEIQSKTY
101 LEVKGTGTAN QCPTVEGGVD SFAFKPGKYT AKKFCLEPTK FAVKAEGISK
151 NSGPDFQNTK LMTRLTYTLD EIEGPFEVSS DGTVKFEEKD GIDYAAVTVQ
201 LPGGERVPFL FTIKQLVASG KPESFSGDFL VPSYRGSSFL DPKGRGGSTG
251 YDNAVALPAG GRGDEEELQK ENNKNVASSK GTITLSVTSS KPETGEVIGV
301 FQSLQPSDTD LGAKVPKDVK IEGVWYAQLE QQ
```

Unformatted sequence string: 332 residues (for pasting into other applications).

Sort by ◉ residue number ○ increasing mass ○ decreasing mass
Show ◉ matched peptides only ○ predicted peptides also

Start - End	Observed	Mr(expt)	Mr(calc)	Delta	M	Peptide
105 - 128	2368.1237	2367.1164	2367.1165	-0.0001	0	K.GTGTANQCPTVEGGVDSFAFKPGK.Y
165 - 185	2300.1180	2299.1107	2299.1107	-0.0000	0	R.LTYTLDEIEGPFEVSSDGTVK.F
215 - 235	2284.1608	2283.1535	2283.1535	-0.0000	0	K.QLVASGKPESFSGDFLVPSYR.G
281 - 314	3462.7849	3461.7776	3461.7778	-0.0001	0	K.GTITLSVTSSKPETGEVIGVFQSLQPSDTDLGAK.V

No match to: 105.0000, 165.0000, 215.0000, 281.0000

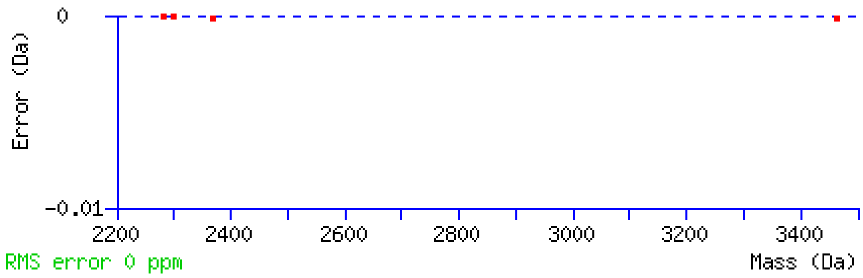

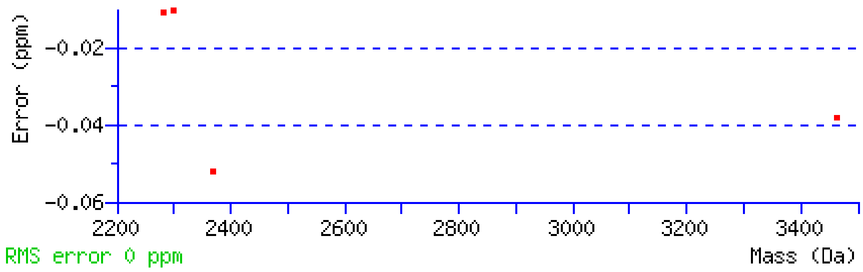

```
ID   PSBO_SPIOL              Reviewed;         332 AA.
AC   P12359;
DT   01-OCT-1989, integrated into UniProtKB/Swiss-Prot.
DT   01-OCT-1989, sequence version 1.
DT   31-JUL-2019, entry version 82.
DE   RecName: Full=Oxygen-evolving enhancer protein 1, chloroplastic;
DE            Short=OEE1;
DE   AltName: Full=33 kDa subunit of oxygen evolving system of photosystem II;
DE   AltName: Full=33 kDa thylakoid membrane protein;
DE   AltName: Full=OEC 33 kDa subunit;
DE   Flags: Precursor;
GN   Name=PSBO;
OS   Spinacia oleracea (Spinach).
OC   Eukaryota; Viridiplantae; Streptophyta; Embryophyta; Tracheophyta;
OC   Spermatophyta; Magnoliopsida; eudicotyledons; Gunneridae;
OC   Pentapetalae; Caryophyllales; Chenopodiaceae; Chenopodioideae;
OC   Anserineae; Spinacia.
OX   NCBI_TaxID=3562;
RN   [1]
RP   NUCLEOTIDE SEQUENCE [MRNA].
RA   Tyagi A., Hermans J., Steppuhn R.C.J., Jansson C., Vater F.,
RA   Herrmann R.G.;
RT   "Nucleotide sequence of cDNA clones encoding the complete '33 kDa'
RT   precursor protein associated with the photosynthetic oxygen-evolving
RT   complex from spinach.";
RL   Mol. Gen. Genet. 207:288-293(1987).
RN   [2]
RP   PROTEIN SEQUENCE OF 85-332.
RA   Oh-Oka H., Tanaka S., Wada K., Kuwabara T., Murata N.;
RT   "Complete amino acid sequence of 33 kDa protein isolated from spinach
RT   photosystem II particles.";
RL   FEBS Lett. 197:63-66(1986).
RN   [3]
RP   PROTEIN SEQUENCE OF 85-121.
RA   Yamamoto Y., Hermodson M.A., Krogmann D.W.;
RT   "Improved purification and N-terminal sequence of the 33-kDa protein
RT   in spinach PS II.";
RL   FEBS Lett. 195:155-158(1986).
RN   [4]
RP   PROTEIN SEQUENCE OF 85-91, SUBCELLULAR LOCATION, AND CHARACTERIZATION.
RX   PubMed=14736920; DOI=10.1073/pnas.0308164100;
RA   Spetea C., Hundal T., Lundin B., Heddad M., Adamska I., Andersson B.;
RT   "Multiple evidence for nucleotide metabolism in the chloroplast
RT   thylakoid lumen.";
RL   Proc. Natl. Acad. Sci. U.S.A. 101:1409-1414(2004).
CC   -!- FUNCTION: Stabilizes the manganese cluster which is the primary
CC       site of water splitting (By similarity). Binds GTP after
CC       preillumination of photosystem II core complex. This binding is
CC       inhibited by DCMU. {ECO:0000250}.
CC   -!- SUBCELLULAR LOCATION: Plastid, chloroplast thylakoid membrane
CC       {ECO:0000269|PubMed:14736920}. Note=Associated with the
CC       photosystem II complex.
CC   -!- SIMILARITY: Belongs to the PsbO family. {ECO:0000305}.
```

```
DR   EMBL; X05548; CAA29062.1; -; mRNA.
DR   PIR; A23613; A23613.
DR   PIR; S00415; S00415.
DR   PDB; 3JCU; EM; 3.20 A; O/o=1-332.
DR   PDBsum; 3JCU; -.
DR   SMR; P12359; -.
DR   DIP; DIP-62020N; -.
DR   IntAct; P12359; 1.
DR   PRIDE; P12359; -.
DR   GO; GO:0030095; C:chloroplast photosystem II; IDA:CAFA.
DR   GO; GO:0009654; C:photosystem II oxygen evolving complex; IMP:CAFA.
DR   GO; GO:0010242; F:oxygen evolving activity; IMP:CAFA.
DR   GO; GO:0055114; P:oxidation-reduction process; IMP:CAFA.
DR   GO; GO:0019684; P:photosynthesis, light reaction; IMP:CAFA.
DR   GO; GO:0010207; P:photosystem II assembly; IMP:CAFA.
DR   GO; GO:0042549; P:photosystem II stabilization; IEA:InterPro.
DR   DisProt; DP00188; -.
DR   InterPro; IPR011250; OMP/PagP_b-brl.
DR   InterPro; IPR002628; PSII_MSP.
DR   PANTHER; PTHR34058; PTHR34058; 1.
DR   Pfam; PF01716; MSP; 1.
DR   SUPFAM; SSF56925; SSF56925; 1.
PE   1: Evidence at protein level;
KW   3D-structure; Chloroplast; Direct protein sequencing; Manganese;
KW   Membrane; Photosynthesis; Photosystem II; Plastid; Thylakoid;
KW   Transit peptide.
FT   TRANSIT       1      ?       Chloroplast.
FT   TRANSIT       ?     84       Thylakoid.
FT   CHAIN        85    332       Oxygen-evolving enhancer protein 1,
FT                                chloroplastic.
FT                                /FTId=PRO_0000029561.
FT   CONFLICT     96     96       Q -> N (in Ref. 3; AA sequence).
FT                                {ECO:0000305}.
FT   CONFLICT    111    111       Q -> E (in Ref. 3; AA sequence).
FT                                {ECO:0000305}.
FT   CONFLICT    120    120       D -> K (in Ref. 3; AA sequence).
FT                                {ECO:0000305}.
FT   HELIX        92     97       {ECO:0000244|PDB:3JCU}.
FT   HELIX       100    103       {ECO:0000244|PDB:3JCU}.
FT   TURN        104    106       {ECO:0000244|PDB:3JCU}.
FT   HELIX       108    110       {ECO:0000244|PDB:3JCU}.
FT   STRAND      136    144       {ECO:0000244|PDB:3JCU}.
FT   STRAND      161    163       {ECO:0000244|PDB:3JCU}.
FT   STRAND      194    200       {ECO:0000244|PDB:3JCU}.
FT   STRAND      206    212       {ECO:0000244|PDB:3JCU}.
FT   STRAND      217    220       {ECO:0000244|PDB:3JCU}.
FT   HELIX       222    224       {ECO:0000244|PDB:3JCU}.
FT   STRAND      225    231       {ECO:0000244|PDB:3JCU}.
FT   STRAND      245    253       {ECO:0000244|PDB:3JCU}.
FT   HELIX       258    263       {ECO:0000244|PDB:3JCU}.
FT   TURN        266    268       {ECO:0000244|PDB:3JCU}.
FT   HELIX       269    272       {ECO:0000244|PDB:3JCU}.
FT   STRAND      279    290       {ECO:0000244|PDB:3JCU}.
FT   TURN        292    294       {ECO:0000244|PDB:3JCU}.
FT   STRAND      296    302       {ECO:0000244|PDB:3JCU}.
FT   STRAND      318    328       {ECO:0000244|PDB:3JCU}.
SQ   SEQUENCE   332 AA;  35171 MW;  B15507F5835117FF CRC64;
     MAASLQASTT FLQPTKVASR NTLQLRSTQN VCKAFGVESA SSGGRLSLSL QSDLKELANK
     CVDATKLAGL ALATSALIAS GANAEGGKRL TYDEIQSKTY LEVKGTGTAN QCPTVEGGVD
     SFAFKPGKYT AKKFCLEPTK FAVKAEGISK NSGPDFQNTK LMTRLTYTLD EIEGPFEVSS
     DGTVKFEEKD GIDYAAVTVQ LPGGERVPFL FTIKQLVASG KPESFSGDFL VPSYRGSSFL
     DPKGRGGSTG YDNAVALPAG GRGDEEELQK ENNKNVASSK GTITLSVTSS KPETGEVIGV
     FQSLQPSDTD LGAKVPKDVK IEGVWYAQLE QQ
```

MATRIX SCIENCE MASCOT Search Results

Protein View: P2SAF_ORYSJ

Photosystem II stability/assembly factor HCF136, chloroplastic OS=Oryza sativa subsp. japonica OX=39947 GN=HCF136 PE=3 SV=1

**Database:**	SwissProt
**Score:**	50
**Expect:**	0.43
**Monoisotopic mass (M_r):**	45441
**Calculated pI:**	9.02
**Taxonomy:**	Oryza sativa Japonica Group

Sequence similarity is available as an NCBI BLAST search of P2SAF_ORYSJ against nr.

Search parameters

**Enzyme:**	Trypsin: cuts C-term side of KR unless next residue is P.
**Mass values searched:**	7
**Mass values matched:**	4

Protein sequence coverage: 24%

Matched peptides shown in ***bold red***.

```
  1 MATTASLHLH LHLLLSSSRR RCRLLVPRAH TDSISTGRRR FIADTATASA
 51 AAAVGPLVLP RTPLARADQP PSLSEWERVL LPIDPGVVLL DIAFVPDDPS
101 HGFLLGTRQT ILETKNGGNT WFPRSIPSAE DEDFNYRFNS VSFMGKEGWI
151 IGKPAILLHT SDAGDSWERI PLSAQLPGNM VYIKATGEQS AEMVTDEGAI
201 YVTSNRGYNW KAAVQETVSA TLNRTVSSGI SGASYYTGTF NTVNRSPDGR
251 YVAVSSRGNF YLTWEPGQPF WQPHNRAVAR RIQNMGWRAD GGLWLLVRGG
301 GLFLSKGSGF QFFYRGLNDA HAISYLHPPN QITEDFEEAS VQSRGFGILD
351 VGYRSKDEAW AAGGSGVLLK TTNGGKTWVR DKAADNIAAN LYSVKFLGDN
401 KGYVLGNDGV LLRYVG
```

Unformatted sequence string: 416 residues (for pasting into other applications).

Sort by ◉ residue number ○ increasing mass ○ decreasing mass
Show ◉ matched peptides only ○ predicted peptides also

Start – End	Observed	Mr(expt)	Mr(calc)	Delta	M	Peptide
79 – 108	3185.7608	3184.7535	3184.7537	-0.0001	0	R.VLLPIDPGVVLLDIAFVPDDPSHGFLLGTR.Q
147 – 169	2551.2939	2550.2866	2550.2867	-0.0000	0	K.EGWIIGKPAILLHTSDAGDSWER.I
258 – 276	2374.1152	2373.1079	2373.1079	-0.0000	0	R.GNFYLTWEPGQPFWQPHNR.A
316 – 344	3238.5399	3237.5326	3237.5327	-0.0000	0	R.GLNDAHAISYLHPPNQITEDFEEASVQSR.G

No match to: 147.0000, 258.0000, 316.0000

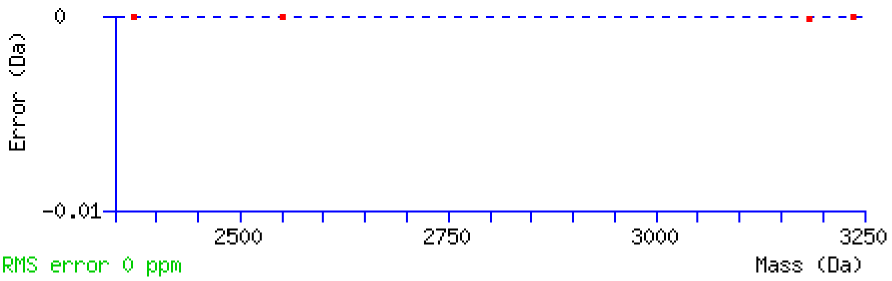

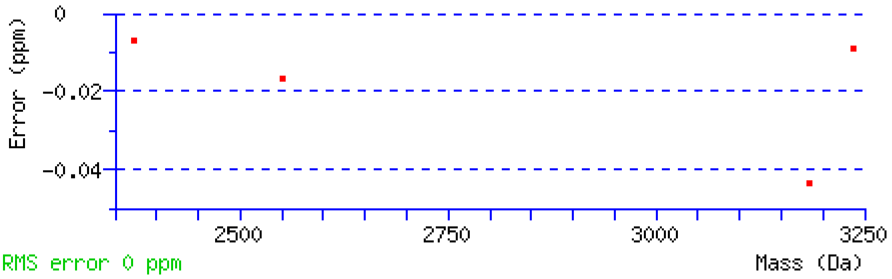

```
ID   P2SAF_ORYSJ             Reviewed;         416 AA.
AC   Q5Z5A8;
DT   13-JUN-2006, integrated into UniProtKB/Swiss-Prot.
DT   23-NOV-2004, sequence version 1.
DT   31-JUL-2019, entry version 67.
DE   RecName: Full=Photosystem II stability/assembly factor HCF136, chloroplastic;
DE   Flags: Precursor;
GN   Name=HCF136; OrderedLocusNames=Os06g0729500, LOC_Os06g51330;
GN   ORFNames=OSJNBa0069C14.15;
OS   Oryza sativa subsp. japonica (Rice).
OC   Eukaryota; Viridiplantae; Streptophyta; Embryophyta; Tracheophyta;
OC   Spermatophyta; Magnoliopsida; Liliopsida; Poales; Poaceae; BOP clade;
OC   Oryzoideae; Oryzeae; Oryzinae; Oryza; Oryza sativa.
OX   NCBI_TaxID=39947;
RN   [1]
RP   NUCLEOTIDE SEQUENCE [LARGE SCALE GENOMIC DNA].
RC   STRAIN=cv. Nipponbare;
RX   PubMed=16100779; DOI=10.1038/nature03895;
RG   International rice genome sequencing project (IRGSP);
RT   "The map-based sequence of the rice genome.";
RL   Nature 436:793-800(2005).
RN   [2]
RP   GENOME REANNOTATION.
RC   STRAIN=cv. Nipponbare;
RX   PubMed=24280374; DOI=10.1186/1939-8433-6-4;
RA   Kawahara Y., de la Bastide M., Hamilton J.P., Kanamori H.,
RA   McCombie W.R., Ouyang S., Schwartz D.C., Tanaka T., Wu J., Zhou S.,
RA   Childs K.L., Davidson R.M., Lin H., Quesada-Ocampo L.,
RA   Vaillancourt B., Sakai H., Lee S.S., Kim J., Numa H., Itoh T.,
RA   Buell C.R., Matsumoto T.;
RT   "Improvement of the Oryza sativa Nipponbare reference genome using
RT   next generation sequence and optical map data.";
RL   Rice 6:4-4(2013).
CC   -!- FUNCTION: Essential for photosystem II (PSII) biogenesis; required
CC       for assembly of an early intermediate in PSII assembly that
CC       includes D2 (psbD) and cytochrome b559. {ECO:0000250}.
CC   -!- SUBCELLULAR LOCATION: Plastid, chloroplast thylakoid membrane
CC       {ECO:0000250}; Peripheral membrane protein {ECO:0000250}; Lumenal
CC       side {ECO:0000250}. Note=Restricted to the stromal lamelae.
CC       Translocation into the thylakoid lumen occurs via the Tat pathway.
CC       {ECO:0000250}.
CC   -!- SIMILARITY: Belongs to the Ycf48 family. {ECO:0000305}.
DR   EMBL; AP005750; BAD62115.1; -; Genomic_DNA.
DR   EMBL; AP014962; -; NOT_ANNOTATED_CDS; Genomic_DNA.
DR   SMR; Q5Z5A8; -.
DR   STRING; 4530.OS06T0729650-00; -.
DR   PRIDE; Q5Z5A8; -.
DR   InParanoid; Q5Z5A8; -.
DR   Proteomes; UP000059680; Chromosome 6.
DR   GO; GO:0009535; C:chloroplast thylakoid membrane; IEA:UniProtKB-SubCell.
DR   GO; GO:0009523; C:photosystem II; IEA:UniProtKB-KW.
DR   GO; GO:0015979; P:photosynthesis; IEA:UniProtKB-KW.
```

```
DR   Gene3D; 2.130.10.10; -; 1.
DR   InterPro; IPR028203; PSII_CF48-like_dom.
DR   InterPro; IPR006311; TAT_signal.
DR   InterPro; IPR015943; WD40/YVTN_repeat-like_dom_sf.
DR   Pfam; PF14870; PSII_BNR; 1.
DR   PROSITE; PS51318; TAT; 1.
PE   3: Inferred from homology;
KW   Chloroplast; Complete proteome; Membrane; Photosynthesis;
KW   Photosystem II; Plastid; Reference proteome; Repeat; Thylakoid;
KW   Transit peptide.
FT   TRANSIT       1     36       Chloroplast. {ECO:0000255}.
FT   TRANSIT      37     67       Thylakoid. {ECO:0000250}.
FT   CHAIN        68    416       Photosystem II stability/assembly factor
FT                                HCF136, chloroplastic.
FT                                /FTId=PRO_0000239672.
FT   REPEAT      112    123       BNR 1.
FT   REPEAT      158    169       BNR 2.
FT   REPEAT      201    212       BNR 3.
FT   REPEAT      369    380       BNR 4.
SQ   SEQUENCE   416 AA;  45469 MW;  6CA9748662B0CB79 CRC64;
     MATTASLHLH LHLLLSSSRR RCRLLVPRAH TDSISTGRRR FIADTATASA AAAVGPLVLP
     RTPLARADQP PSLSEWERVL LPIDPGVVLL DIAFVPDDPS HGFLLGTRQT ILETKNGGNT
     WFPRSIPSAE DEDFNYRFNS VSFMGKEGWI IGKPAILLHT SDAGDSWERI PLSAQLPGNM
     VYIKATGEQS AEMVTDEGAI YVTSNRGYNW KAAVQETVSA TLNRTVSSGI SGASYYTGTF
     NTVNRSPDGR YVAVSSRGNF YLTWEPGQPF WQPHNRAVAR RIQNMGWRAD GGLWLLVRGG
     GLFLSKGSGF QFFYRGLNDA HAISYLHPPN QITEDFEEAS VQSRGFGILD VGYRSKDEAW
     AAGGSGVLLK TTNGGKTWVR DKAADNIAAN LYSVKFLGDN KGYVLGNDGV LLRYVG
```

Mascot: http://www.matrixscience.com/

MATRIX SCIENCE MASCOT Search Results

Protein View: HIS7A_ARATH

Imidazoleglycerol-phosphate dehydratase 1, chloroplastic OS=Arabidopsis thaliana OX=3702 GN=HISN5A PE=1 SV=1

**Database:**	SwissProt
**Score:**	74
**Expect:**	0.0015
**Monoisotopic mass (M_r):**	29207
**Calculated pI:**	7.23
**Taxonomy:**	Arabidopsis thaliana

Sequence similarity is available as an NCBI BLAST search of HIS5A_ARATH against nr.

Search parameters

**Enzyme:**	Trypsin: cuts C-term side of KR unless next residue is P.
**Mass values searched:**	9
**Mass values matched:**	5

Protein sequence coverage: 51%

Matched peptides shown in ***bold red***.

```
  1 MELSSASAIL SHSSSAAQLL RPKLGFIDLL PRRAMIVSSP SSSLPRFLRM
 51 ESQSQLRQSI SCSASSSSSM ALGRIGEVKR VTKETNVSVK INLDGTGVAD
101 SSSGIPFLDH MLDQLASHGL FDVHVRATGD VHIDDHHTNE DIALAIGTAL
151 LKALGERKGI NRFGDFTAPL DEALIHVSLD LSGRPYLGYN LEIPTQRVGT
201 YDTQLVEHFF QSLVNTSGMT LHIRQLAGEN SHHIIEATFK AFARALRQAT
251 ETDPRRGGTI PSSKGVLSRS
```

Unformatted sequence string: 270 residues (for pasting into other applications).

Sort by ◉ residue number ○ increasing mass ○ decreasing mass
Show ◉ matched peptides only ○ predicted peptides also

Start – End	Observed	Mr(expt)	Mr(calc)	Delta	M	Peptide
91 – 126	3833.8915	3832.8842	3832.8843	-0.0001	0	K.INLDGTGVADSSSGIPFLDHMLDQLASHGLFDVHVR.A
127 – 152	2740.3900	2739.3827	2739.3828	-0.0000	0	R.ATGDVHIDDHHTNEDIALAIGTALLK.A
163 – 197	3918.0071	3916.9998	3916.9999	-0.0001	0	R.FGDFTAPLDEALIHVSLDLSGRPYLGYNLEIPTQR.V
198 – 224	3093.5462	3092.5389	3092.5390	-0.0001	0	R.VGTYDTQLVEHFFQSLVNTSGMTLHIR.Q
225 – 240	1794.9133	1793.9060	1793.9060	0.0000	0	R.QLAGENSHHIIEATFK.A

No match to: 127.0000, 163.0000, 198.0000, 225.0000

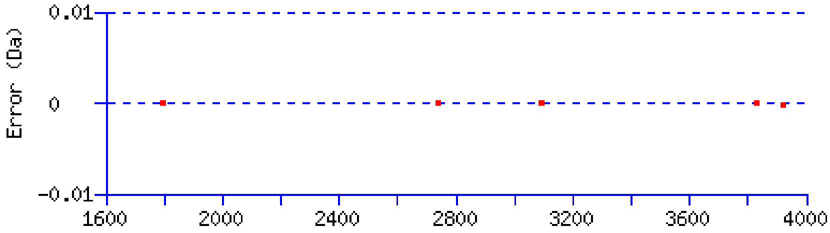

RMS error 0 ppm

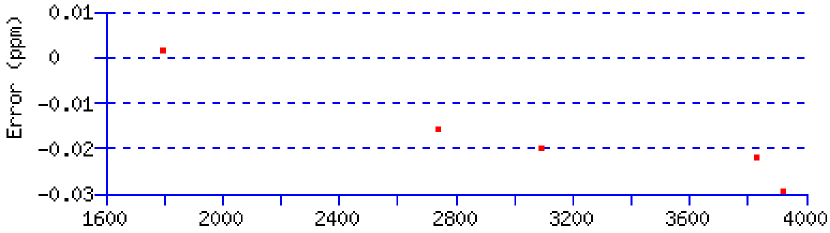

RMS error 0 ppm

```
ID   HIS5A_ARATH             Reviewed;         270 AA.
AC   P34047; Q67YN9; Q67YZ9; Q8VYM1;
DT   01-FEB-1994, integrated into UniProtKB/Swiss-Prot.
DT   01-FEB-1994, sequence version 1.
DT   31-JUL-2019, entry version 147.
DE   RecName: Full=Imidazoleglycerol-phosphate dehydratase 1, chloroplastic {ECO:0000303|PubMed:8066131};
DE            Short=IGPD 1 {ECO:0000303|PubMed:8066131};
DE            EC=4.2.1.19 {ECO:0000269|PubMed:8066131};
DE   AltName: Full=Protein HISTIDINE BIOSYNTHESIS 5A {ECO:0000303|PubMed:16547652};
DE   Flags: Precursor;
GN   Name=HISN5A {ECO:0000303|PubMed:16547652};
GN   OrderedLocusNames=At3g22425 {ECO:0000312|Araport:AT3G22425};
GN   ORFNames=MCB17.17 {ECO:0000312|EMBL:BAB01781.1};
OS   Arabidopsis thaliana (Mouse-ear cress).
OC   Eukaryota; Viridiplantae; Streptophyta; Embryophyta; Tracheophyta;
OC   Spermatophyta; Magnoliopsida; eudicotyledons; Gunneridae;
OC   Pentapetalae; rosids; malvids; Brassicales; Brassicaceae; Camelineae;
OC   Arabidopsis.
OX   NCBI_TaxID=3702;
RN   [1]
RP   NUCLEOTIDE SEQUENCE [MRNA] (ISOFORM 1), CATALYTIC ACTIVITY, AND
RP   PATHWAY.
RX   PubMed=8066131; DOI=10.1104/pp.105.2.579;
RA   Tada S., Volrath S., Guyer D., Scheidegger A., Ryals J., Ohta D.,
RA   Ward E.;
RT   "Isolation and characterization of cDNAs encoding
RT   imidazoleglycerolphosphate dehydratase from Arabidopsis thaliana.";
RL   Plant Physiol. 105:579-583(1994).
RN   [2]
RP   NUCLEOTIDE SEQUENCE [LARGE SCALE GENOMIC DNA].
RC   STRAIN=cv. Columbia;
RX   PubMed=10819329; DOI=10.1093/dnares/7.2.131;
RA   Sato S., Nakamura Y., Kaneko T., Katoh T., Asamizu E., Tabata S.;
RT   "Structural analysis of Arabidopsis thaliana chromosome 3. I. Sequence
RT   features of the regions of 4,504,864 bp covered by sixty P1 and TAC
RT   clones.";
RL   DNA Res. 7:131-135(2000).
RN   [3]
RP   GENOME REANNOTATION.
RC   STRAIN=cv. Columbia;
RX   PubMed=27862469; DOI=10.1111/tpj.13415;
RA   Cheng C.Y., Krishnakumar V., Chan A.P., Thibaud-Nissen F., Schobel S.,
RA   Town C.D.;
RT   "Araport11: a complete reannotation of the Arabidopsis thaliana
RT   reference genome.";
RL   Plant J. 89:789-804(2017).
RN   [4]
RP   NUCLEOTIDE SEQUENCE [LARGE SCALE MRNA] (ISOFORM 1).
RC   STRAIN=cv. Columbia;
RX   PubMed=11910074; DOI=10.1126/science.1071006;
RA   Seki M., Narusaka M., Kamiya A., Ishida J., Satou M., Sakurai T.,
RA   Nakajima M., Enju A., Akiyama K., Oono Y., Muramatsu M.,
RA   Hayashizaki Y., Kawai J., Carninci P., Itoh M., Ishii Y., Arakawa T.,
RA   Shibata K., Shinagawa A., Shinozaki K.;
RT   "Functional annotation of a full-length Arabidopsis cDNA collection.";
RL   Science 296:141-145(2002).
RN   [5]
RP   NUCLEOTIDE SEQUENCE [LARGE SCALE MRNA] (ISOFORM 2).
RC   STRAIN=cv. Columbia;
RX   PubMed=14593172; DOI=10.1126/science.1088305;
RA   Yamada K., Lim J., Dale J.M., Chen H., Shinn P., Palm C.J.,
RA   Southwick A.M., Wu H.C., Kim C.J., Nguyen M., Pham P.K., Cheuk R.F.,
RA   Karlin-Newmann G., Liu S.X., Lam B., Sakano H., Wu T., Yu G.,
RA   Miranda M., Quach H.L., Tripp M., Chang C.H., Lee J.M., Toriumi M.J.,
RA   Chan M.M., Tang C.C., Onodera C.S., Deng J.M., Akiyama K., Ansari Y.,
RA   Arakawa T., Banh J., Banno F., Bowser L., Brooks S.Y., Carninci P.,
RA   Chao Q., Choy N., Enju A., Goldsmith A.D., Gurjal M., Hansen N.F.,
RA   Hayashizaki Y., Johnson-Hopson C., Hsuan V.W., Iida K., Karnes M.,
RA   Khan S., Koesema E., Ishida J., Jiang P.X., Jones T., Kawai J.,
RA   Kamiya A., Meyers C., Nakajima M., Narusaka M., Seki M., Sakurai T.,
RA   Satou M., Tamse R., Vaysberg M., Wallender E.K., Wong C., Yamamura Y.,
RA   Yuan S., Shinozaki K., Davis R.W., Theologis A., Ecker J.R.;
RT   "Empirical analysis of transcriptional activity in the Arabidopsis
RT   genome.";
RL   Science 302:842-846(2003).
RN   [6]
RP   NUCLEOTIDE SEQUENCE [LARGE SCALE MRNA] (ISOFORM 1).
RC   STRAIN=cv. Columbia;
RA   Totoki Y., Seki M., Ishida J., Nakajima M., Enju A., Kamiya A.,
RA   Narusaka M., Shin-i T., Nakagawa M., Sakamoto N., Oishi K., Kohara Y.,
```

```
RA   Kobayashi M., Toyoda A., Sakaki Y., Sakurai T., Iida K., Akiyama K.,
RA   Satou M., Toyoda T., Konagaya A., Carninci P., Kawai J.,
RA   Hayashizaki Y., Shinozaki K.;
RT   "Large-scale analysis of RIKEN Arabidopsis full-length (RAFL) cDNAs.";
RL   Submitted (SEP-2004) to the EMBL/GenBank/DDBJ databases.
RN   [7]
RP   NUCLEOTIDE SEQUENCE [LARGE SCALE MRNA] (ISOFORM 1).
RA   Brover V.V., Troukhan M.E., Alexandrov N.A., Lu Y.-P., Flavell R.B.,
RA   Feldmann K.A.;
RT   "Full-length cDNA from Arabidopsis thaliana.";
RL   Submitted (MAR-2002) to the EMBL/GenBank/DDBJ databases.
RN   [8]
RP   GENE FAMILY, AND NOMENCLATURE.
RX   PubMed=16547652; DOI=10.1007/s00726-005-0247-0;
RA   Stepansky A., Leustek T.;
RT   "Histidine biosynthesis in plants.";
RL   Amino Acids 30:127-142(2006).
RN   [9]
RP   GENE FAMILY, AND NOMENCLATURE.
RX   PubMed=17434988; DOI=10.1104/pp.107.096511;
RA   Muralla R., Sweeney C., Stepansky A., Leustek T., Meinke D.;
RT   "Genetic dissection of histidine biosynthesis in Arabidopsis.";
RL   Plant Physiol. 144:890-903(2007).
RN   [10]
RP   ACETYLATION [LARGE SCALE ANALYSIS] AT SER-63, CLEAVAGE OF TRANSIT
RP   PEPTIDE [LARGE SCALE ANALYSIS] AFTER CYS-62, AND IDENTIFICATION BY
RP   MASS SPECTROMETRY [LARGE SCALE ANALYSIS].
RX   PubMed=22223895; DOI=10.1074/mcp.M111.015131;
RA   Bienvenut W.V., Sumpton D., Martinez A., Lilla S., Espagne C.,
RA   Meinnel T., Giglione C.;
RT   "Comparative large-scale characterisation of plant vs. mammal proteins
RT   reveals similar and idiosyncratic N-alpha acetylation features.";
RL   Mol. Cell. Proteomics 11:M111.015131-M111.015131(2012).
RN   [11]
RP   X-RAY CRYSTALLOGRAPHY (3.00 ANGSTROMS) OF 64-270 IN COMPLEX WITH
RP   MANGANESE IONS.
RX   PubMed=16338409; DOI=10.1016/j.str.2005.08.012;
RA   Glynn S.E., Baker P.J., Sedelnikova S.E., Davies C.L., Eadsforth T.C.,
RA   Levy C.W., Rodgers H.F., Blackburn G.M., Hawkes T.R., Viner R.,
RA   Rice D.W.;
RT   "Structure and mechanism of imidazoleglycerol-phosphate dehydratase.";
RL   Structure 13:1809-1817(2005).
CC   -!- CATALYTIC ACTIVITY:
CC       Reaction=D-erythro-1-(imidazol-4-yl)glycerol 3-phosphate = 3-
CC         (imidazol-4-yl)-2-oxopropyl phosphate + H2O;
CC         Xref=Rhea:RHEA:11040, ChEBI:CHEBI:15377, ChEBI:CHEBI:57766,
CC         ChEBI:CHEBI:58278; EC=4.2.1.19;
CC         Evidence={ECO:0000269|PubMed:8066131};
CC   -!- COFACTOR:
CC       Name=Mn(2+); Xref=ChEBI:CHEBI:29035;
CC         Evidence={ECO:0000269|PubMed:16338409};
CC       Note=Binds 2 manganese ions per subunit.
CC       {ECO:0000269|PubMed:16338409};
CC   -!- PATHWAY: Amino-acid biosynthesis; L-histidine biosynthesis; L-
CC       histidine from 5-phospho-alpha-D-ribose 1-diphosphate: step 6/9.
CC       {ECO:0000269|PubMed:8066131}.
CC   -!- SUBCELLULAR LOCATION: Plastid, chloroplast {ECO:0000305}.
CC   -!- ALTERNATIVE PRODUCTS:
CC       Event=Alternative splicing; Named isoforms=2;
CC         Comment=Experimental confirmation may be lacking for some
CC         isoforms.;
CC       Name=1;
CC         IsoId=P34047-1; Sequence=Displayed;
CC       Name=2;
CC         IsoId=P34047-2; Sequence=VSP_008895;
CC         Note=May be due to an intron retention.;
CC   -!- SIMILARITY: Belongs to the imidazoleglycerol-phosphate dehydratase
CC       family. {ECO:0000305}.
DR   EMBL; U02689; AAA93196.1; -; mRNA.
DR   EMBL; AB022215; BAB01781.1; -; Genomic_DNA.
DR   EMBL; CP002686; AEE76636.1; -; Genomic_DNA.
DR   EMBL; CP002686; AEE76637.1; -; Genomic_DNA.
DR   EMBL; AK118815; BAC43405.1; -; mRNA.
DR   EMBL; AY070442; AAL49845.1; -; mRNA.
DR   EMBL; AK176319; BAD44082.1; -; mRNA.
DR   EMBL; AK176429; BAD44192.1; -; mRNA.
DR   EMBL; AY087948; AAM65496.1; -; mRNA.
DR   RefSeq; NP_850624.1; NM_180293.1. [P34047-2]
DR   RefSeq; NP_850625.1; NM_180294.2. [P34047-1]
DR   PDB; 2F1D; X-ray; 3.00 A; A/B/C/D/E/F/G/H/I/J/K/L/M/N/O/P=64-270.
```

```
DR   PDBsum; 2F1D; -.
DR   SMR; P34047; -.
DR   BioGrid; 7144; 1.
DR   DIP; DIP-48462N; -.
DR   STRING; 3702.AT3G22425.2; -.
DR   iPTMnet; P34047; -.
DR   PaxDb; P34047; -.
DR   PRIDE; P34047; -.
DR   EnsemblPlants; AT3G22425.1; AT3G22425.1; AT3G22425. [P34047-2]
DR   EnsemblPlants; AT3G22425.2; AT3G22425.2; AT3G22425. [P34047-1]
DR   GeneID; 821812; -.
DR   Gramene; AT3G22425.1; AT3G22425.1; AT3G22425. [P34047-2]
DR   Gramene; AT3G22425.2; AT3G22425.2; AT3G22425. [P34047-1]
DR   KEGG; ath:AT3G22425; -.
DR   Araport; AT3G22425; -.
DR   TAIR; locus:1005716545; AT3G22425.
DR   eggNOG; KOG3143; Eukaryota.
DR   eggNOG; COG0131; LUCA.
DR   HOGENOM; HOG000228064; -.
DR   KO; K01693; -.
DR   OMA; HIDTHHT; -.
DR   OrthoDB; 1097523at2759; -.
DR   PhylomeDB; P34047; -.
DR   BioCyc; MetaCyc:AT3G22425-MONOMER; -.
DR   UniPathway; UPA00031; UER00011.
DR   EvolutionaryTrace; P34047; -.
DR   PRO; PR:P34047; -.
DR   Proteomes; UP000006548; Chromosome 3.
DR   ExpressionAtlas; P34047; baseline and differential.
DR   Genevisible; P34047; AT.
DR   GO; GO:0009507; C:chloroplast; IEA:UniProtKB-SubCell.
DR   GO; GO:0004424; F:imidazoleglycerol-phosphate dehydratase activity; IDA:TAIR.
DR   GO; GO:0046872; F:metal ion binding; IEA:UniProtKB-KW.
DR   GO; GO:0000105; P:histidine biosynthetic process; IDA:TAIR.
DR   CDD; cd07914; IGPD; 1.
DR   Gene3D; 3.30.230.40; -; 2.
DR   HAMAP; MF_00076; HisB; 1.
DR   InterPro; IPR038494; IGPD_sf.
DR   InterPro; IPR000807; ImidazoleglycerolP_deHydtase.
DR   InterPro; IPR020565; ImidazoleglycerP_deHydtase_CS.
DR   InterPro; IPR020568; Ribosomal_S5_D2-typ_fold.
DR   PANTHER; PTHR23133; PTHR23133; 1.
DR   Pfam; PF00475; IGPD; 1.
DR   SUPFAM; SSF54211; SSF54211; 2.
DR   PROSITE; PS00954; IGP_DEHYDRATASE_1; 1.
DR   PROSITE; PS00955; IGP_DEHYDRATASE_2; 1.
PE   1: Evidence at protein level;
KW   3D-structure; Acetylation; Alternative splicing;
KW   Amino-acid biosynthesis; Chloroplast; Complete proteome;
KW   Histidine biosynthesis; Lyase; Manganese; Metal-binding; Plastid;
KW   Reference proteome; Transit peptide.
FT   TRANSIT       1     62       Chloroplast.
FT                                {ECO:0000244|PubMed:22223895}.
FT   CHAIN        63    270       Imidazoleglycerol-phosphate dehydratase
FT                                1, chloroplastic.
FT                                /FTId=PRO_0000158253.
FT   REGION      110    118       Substrate binding.
FT                                {ECO:0000250|UniProtKB:O23346}.
FT   REGION      136    140       Substrate binding.
FT                                {ECO:0000250|UniProtKB:O23346}.
FT   REGION      232    240       Substrate binding.
FT                                {ECO:0000250|UniProtKB:O23346}.
FT   REGION      262    264       Substrate binding.
FT                                {ECO:0000250|UniProtKB:O23346}.
FT   METAL       110    110       Manganese 1; via tele nitrogen.
FT                                {ECO:0000244|PDB:2F1D,
FT                                ECO:0000269|PubMed:16338409}.
FT   METAL       136    136       Manganese 2; via tele nitrogen.
FT                                {ECO:0000244|PDB:2F1D,
FT                                ECO:0000269|PubMed:16338409}.
FT   METAL       137    137       Manganese 1; via tele nitrogen.
FT                                {ECO:0000244|PDB:2F1D,
FT                                ECO:0000269|PubMed:16338409}.
FT   METAL       140    140       Manganese 2. {ECO:0000244|PDB:2F1D,
FT                                ECO:0000269|PubMed:16338409}.
FT   METAL       208    208       Manganese 2; via tele nitrogen.
FT                                {ECO:0000244|PDB:2F1D,
FT                                ECO:0000269|PubMed:16338409}.
FT   METAL       232    232       Manganese 1; via tele nitrogen.
FT                                {ECO:0000244|PDB:2F1D,
```

```
FT                                   ECO:0000269|PubMed:16338409}.
FT   METAL       233    233       Manganese 2; via tele nitrogen.
FT                                   {ECO:0000244|PDB:2F1D,
FT                                   ECO:0000269|PubMed:16338409}.
FT   METAL       236    236       Manganese 1. {ECO:0000244|PDB:2F1D,
FT                                   ECO:0000269|PubMed:16338409}.
FT   BINDING      84     84       Substrate.
FT                                   {ECO:0000250|UniProtKB:O23346}.
FT   BINDING     162    162       Substrate.
FT                                   {ECO:0000250|UniProtKB:O23346}.
FT   BINDING     184    184       Substrate.
FT                                   {ECO:0000250|UniProtKB:O23346}.
FT   MOD_RES      63     63       N-acetylserine.
FT                                   {ECO:0000244|PubMed:22223895}.
FT   VAR_SEQ     205    270       LVEHFFQSLVNTSGMTLHIRQLAGENSHHIIEATFKAFARA
FT                                   LRQATETDPRRGGTIPSSKGVLSRS -> VLSLLLELSSFG
FT                                   FICVIRCLVIIESVAKNCLTFRFVVGGALFPVVGEYFWYDS
FT                                   SHPAARW (in isoform 2).
FT                                   {ECO:0000303|PubMed:14593172}.
FT                                   /FTId=VSP_008895.
FT   CONFLICT    201    201       Y -> C (in Ref. 6; BAD44082).
FT                                   {ECO:0000305}.
FT   STRAND       75     81       {ECO:0000244|PDB:2F1D}.
FT   STRAND       86     92       {ECO:0000244|PDB:2F1D}.
FT   STRAND       99    102       {ECO:0000244|PDB:2F1D}.
FT   HELIX       106    119       {ECO:0000244|PDB:2F1D}.
FT   STRAND      122    128       {ECO:0000244|PDB:2F1D}.
FT   TURN        131    133       {ECO:0000244|PDB:2F1D}.
FT   HELIX       136    154       {ECO:0000244|PDB:2F1D}.
FT   STRAND      166    170       {ECO:0000244|PDB:2F1D}.
FT   STRAND      173    180       {ECO:0000244|PDB:2F1D}.
FT   STRAND      186    190       {ECO:0000244|PDB:2F1D}.
FT   STRAND      194    198       {ECO:0000244|PDB:2F1D}.
FT   HELIX       205    217       {ECO:0000244|PDB:2F1D}.
FT   STRAND      220    227       {ECO:0000244|PDB:2F1D}.
FT   HELIX       231    250       {ECO:0000244|PDB:2F1D}.
SQ   SEQUENCE   270 AA;  29225 MW;  7132D80CC687E20C CRC64;
     MELSSASAIL SHSSSAAQLL RPKLGFIDLL PRRAMIVSSP SSSLPRFLRM ESQSQLRQSI
     SCSASSSSSM ALGRIGEVKR VTKETNVSVK INLDGTGVAD SSSGIPFLDH MLDQLASHGL
     FDVHVRATGD VHIDDHHTNE DIALAIGTAL LKALGERKGI NRFGDFTAPL DEALIHVSLD
     LSGRPYLGYN LEIPTQRVGT YDTQLVEHFF QSLVNTSGMT LHIRQLAGEN SHHIIEATFK
     AFARALRQAT ETDPRRGGTI PSSKGVLSRS
```

MATRIX SCIENCE

MASCOT Search Results

Protein View: MDHC1_ARATH

Malate dehydrogenase [NADP], chloroplastic OS=Arabidopsis thaliana OX=3702 GN=At5g58330 PE=2 SV=1

**Database:**	SwissProt
**Score:**	37
**Expect:**	8.4
**Monoisotopic mass (M_r):**	38286
**Calculated pI:**	6.01
**Taxonomy:**	Arabidopsis thaliana

Sequence similarity is available as **an NCBI BLAST search of MDHNP_ARATH against nr**.

Search parameters

**Enzyme:**	Trypsin: cuts C-term side of KR unless next residue is P.
**Mass values searched:**	28
**Mass values matched:**	7

Protein sequence coverage: 24%

Matched peptides shown in ***bold red***.

1 MAMAELSTPK TTSPFLNSSS RLRLSSKLHL SNHFRHLLLP PLHTTTPNSK
51 ISCSVSQNSQ APVAVQENGL VKTKK**ECYGV** **FCLTYDLK**AE EETRSWKKLI
101 NIAVSGAAGM ISNHLLFK**LA** **SGEVFGPDQP** **IALK**LLGSER SIQALEGVAM
151 ELEDSLFPLL REVDIGTDPN EVFQDVEWAI LIGAKPRGPG MER**ADLLDIN**
201 **GQIFAEQGK**A LNKAASPNVK **VLVVGNPCNT** **NALICLK**NAP NIPAKNFHAL
251 TRLDENR**AK**C QLALKAGVFY DKVSNMTIWG NHSTTQVPDF LNARINGLPV
301 KEVITDHKWL EEGFTESVQK RGGLLIQKWG RSSAASTAVS IVDAIK**SLVT**
351 **PTPEGDWFST** **GVYTDGNPYG** **IEEGLVFSMP** **CR**SK**GDGDYE** **LVK**DVEIDDY
401 LRQRIAKSEA ELLAEKRCVA HLTGEGIAYC DLGPVDTMLP GEV

Unformatted sequence string: **443 residues** (for pasting into other applications).

Sort by ◉ residue number ○ increasing mass ○ decreasing mass
Show ◉ matched peptides only ○ predicted peptides also

Start – End	Observed	Mr(expt)	Mr(calc)	Delta	M	Peptide
76 – 88	1552.7716	1551.7643	1552.6942	-0.9299	0	K.ECYGVFCLTYDLK.A
119 – 134	1641.8846	1640.8773	1640.8774	-0.0000	0	K.LASGEVFGPDQPIALK.L
194 – 209	1731.8911	1730.8838	1730.8839	-0.0001	0	R.ADLLDINGQIFAEQGK.A
221 – 237	1770.9604	1769.9531	1769.9532	-0.0001	0	K.VLVVGNPCNTNALICLK.N
258 – 259	219.0000	217.9927	217.1426	0.8501	0	R.AK.C
347 – 382	3920.8145	3919.8072	3920.7914	-0.9842	0	K.SLVTPTPEGDWFSTGVYTDGNPYGIEEGLVFSMPCR.S
385 – 393	995.4680	994.4607	994.4607	-0.0000	0	K.GDGDYELVK.D

No match to: 117.0000, 139.0000, 160.0000, 192.0000, 271.0000, 307.0000, 330.0000, 345.0000, 383.0000, 392.0000, 406.0000, 416.0000, 1047.5204, 1141.5524, 1391.7376, 2074.1187, 2367.1900, 2460.1976, 2588.1499, 2858.5086, 3693.8291

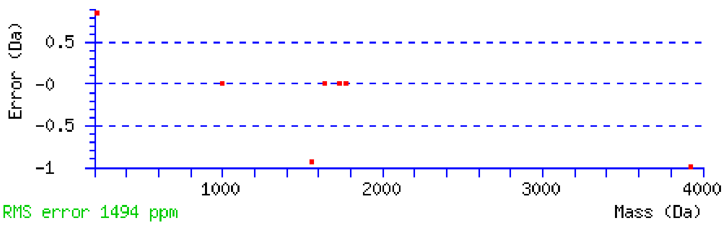

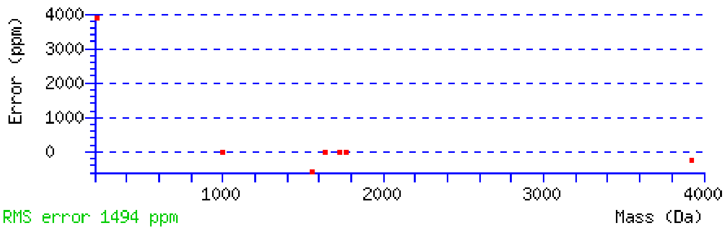


```
ID   MDHNP_ARATH             Reviewed;         443 AA.
AC   Q8H1E2; Q8LCQ9; Q8VXZ3; Q9LVL7;
DT   30-NOV-2016, integrated into UniProtKB/Swiss-Prot.
DT   01-MAR-2003, sequence version 1.
DT   31-JUL-2019, entry version 137.
DE   RecName: Full=Malate dehydrogenase [NADP], chloroplastic {ECO:0000305};
DE            EC=1.1.1.82 {ECO:0000305};
DE   AltName: Full=NADP-MDH {ECO:0000305};
DE   Flags: Precursor;
GN   OrderedLocusNames=At5g58330 {ECO:0000312|Araport:AT5G58330};
OS   Arabidopsis thaliana (Mouse-ear cress).
OC   Eukaryota; Viridiplantae; Streptophyta; Embryophyta; Tracheophyta;
OC   Spermatophyta; Magnoliopsida; eudicotyledons; Gunneridae;
OC   Pentapetalae; rosids; malvids; Brassicales; Brassicaceae; Camelineae;
OC   Arabidopsis.
OX   NCBI_TaxID=3702;
```

```
RN   [1]
RP   NUCLEOTIDE SEQUENCE [LARGE SCALE GENOMIC DNA].
RC   STRAIN=cv. Columbia;
RX   PubMed=10718197; DOI=10.1093/dnares/7.1.31;
RA   Sato S., Nakamura Y., Kaneko T., Katoh T., Asamizu E., Kotani H.,
RA   Tabata S.;
RT   "Structural analysis of Arabidopsis thaliana chromosome 5. X. Sequence
RT   features of the regions of 3,076,755 bp covered by sixty P1 and TAC
RT   clones.";
RL   DNA Res. 7:31-63(2000).
RN   [2]
RP   GENOME REANNOTATION.
RC   STRAIN=cv. Columbia;
RX   PubMed=27862469; DOI=10.1111/tpj.13415;
RA   Cheng C.Y., Krishnakumar V., Chan A.P., Thibaud-Nissen F., Schobel S.,
RA   Town C.D.;
RT   "Araport11: a complete reannotation of the Arabidopsis thaliana
RT   reference genome.";
RL   Plant J. 89:789-804(2017).
RN   [3]
RP   NUCLEOTIDE SEQUENCE [LARGE SCALE MRNA] (ISOFORM 1).
RC   STRAIN=cv. Columbia;
RX   PubMed=14593172; DOI=10.1126/science.1088305;
RA   Yamada K., Lim J., Dale J.M., Chen H., Shinn P., Palm C.J.,
RA   Southwick A.M., Wu H.C., Kim C.J., Nguyen M., Pham P.K., Cheuk R.F.,
RA   Karlin-Newmann G., Liu S.X., Lam B., Sakano H., Wu T., Yu G.,
RA   Miranda M., Quach H.L., Tripp M., Chang C.H., Lee J.M., Toriumi M.J.,
RA   Chan M.M., Tang C.C., Onodera C.S., Deng J.M., Akiyama K., Ansari Y.,
RA   Arakawa T., Banh J., Banno F., Bowser L., Brooks S.Y., Carninci P.,
RA   Chao Q., Choy N., Enju A., Goldsmith A.D., Gurjal M., Hansen N.F.,
RA   Hayashizaki Y., Johnson-Hopson C., Hsuan V.W., Iida K., Karnes M.,
RA   Khan S., Koesema E., Ishida J., Jiang P.X., Jones T., Kawai J.,
RA   Kamiya A., Meyers C., Nakajima M., Narusaka M., Seki M., Sakurai T.,
RA   Satou M., Tamse R., Vaysberg M., Wallender E.K., Wong C., Yamamura Y.,
RA   Yuan S., Shinozaki K., Davis R.W., Theologis A., Ecker J.R.;
RT   "Empirical analysis of transcriptional activity in the Arabidopsis
RT   genome.";
RL   Science 302:842-846(2003).
RN   [4]
RP   NUCLEOTIDE SEQUENCE [LARGE SCALE MRNA] (ISOFORM 1).
RC   STRAIN=cv. Columbia;
RA   Totoki Y., Seki M., Ishida J., Nakajima M., Enju A., Kamiya A.,
RA   Narusaka M., Shin-i T., Nakagawa M., Sakamoto N., Oishi K., Kohara Y.,
RA   Kobayashi M., Toyoda A., Sakaki Y., Sakurai T., Iida K., Akiyama K.,
RA   Satou M., Toyoda T., Konagaya A., Carninci P., Kawai J.,
RA   Hayashizaki Y., Shinozaki K.;
RT   "Large-scale analysis of RIKEN Arabidopsis full-length (RAFL) cDNAs.";
RL   Submitted (JUL-2006) to the EMBL/GenBank/DDBJ databases.
RN   [5]
RP   NUCLEOTIDE SEQUENCE [LARGE SCALE MRNA] (ISOFORM 1).
RA   Brover V.V., Troukhan M.E., Alexandrov N.A., Lu Y.-P., Flavell R.B.,
RA   Feldmann K.A.;
RT   "Full-length cDNA from Arabidopsis thaliana.";
RL   Submitted (MAR-2002) to the EMBL/GenBank/DDBJ databases.
RN   [6]
RP   INDUCTION.
RX   PubMed=17925997; DOI=10.1007/s00239-007-9025-9;
RA   Hameister S., Becker B., Holtgrefe S., Strodtkoetter I., Linke V.,
RA   Backhausen J.E., Scheibe R.;
RT   "Transcriptional regulation of NADP-dependent malate dehydrogenase:
RT   comparative genetics and identification of DNA-binding proteins.";
RL   J. Mol. Evol. 65:437-455(2007).
RN   [7]
RP   DISRUPTION PHENOTYPE.
RX   PubMed=22140244; DOI=10.1093/jxb/err386;
RA   Hebbelmann I., Selinski J., Wehmeyer C., Goss T., Voss I., Mulo P.,
RA   Kangasjaervi S., Aro E.M., Oelze M.L., Dietz K.J., Nunes-Nesi A.,
RA   Do P.T., Fernie A.R., Talla S.K., Raghavendra A.S., Linke V.,
RA   Scheibe R.;
RT   "Multiple strategies to prevent oxidative stress in Arabidopsis plants
RT   lacking the malate valve enzyme NADP-malate dehydrogenase.";
RL   J. Exp. Bot. 63:1445-1459(2012).
RN   [8]
RP   FUNCTION.
RX   PubMed=24591715; DOI=10.1098/rstb.2013.0228;
RA   Heyno E., Innocenti G., Lemaire S.D., Issakidis-Bourguet E.,
RA   Krieger-Liszkay A.;
RT   "Putative role of the malate valve enzyme NADP-malate dehydrogenase in
RT   H2O2 signalling in Arabidopsis.";
RL   Philos. Trans. R. Soc. Lond., B, Biol. Sci.
RL   369:20130228-20130228(2014).
CC   -!- FUNCTION: The chloroplastic, NADP-dependent form is essential for
CC       the photosynthesis C4 cycle, which allows plants to circumvent the
CC       problem of photorespiration. In C4 plants, NADP-MDH activity acts
CC       to convert oxaloacetate to malate in chloroplasts of mesophyll
CC       cells for transport to the bundle sheath cells (Probable). Plays
CC       an essential role in the regulation of catalase activity and the
CC       accumulation of a hydrogen peroxide-dependent signal by
CC       transmitting the redox state of the chloroplast to other cell
CC       compartments (PubMed:24591715). {ECO:0000269|PubMed:24591715,
CC       ECO:0000305}.
CC   -!- CATALYTIC ACTIVITY:
```

```
CC       Reaction=(S)-malate + NADP(+) = H(+) + NADPH + oxaloacetate;
CC         Xref=Rhea:RHEA:10824, ChEBI:CHEBI:15378, ChEBI:CHEBI:15589,
CC         ChEBI:CHEBI:16452, ChEBI:CHEBI:57783, ChEBI:CHEBI:58349;
CC         EC=1.1.1.82; Evidence={ECO:0000305};
CC   -!- ACTIVITY REGULATION: Chloroplast NADP-MDH is activated upon
CC       illumination. In order to be enzymatically active, disulfide
CC       bridges on the protein must be reduced by thioredoxin which
CC       receives electrons from ferredoxin and the electron transport
CC       system of photosynthesis.
CC   -!- SUBUNIT: Homodimer. {ECO:0000250|UniProtKB:P21528}.
CC   -!- SUBCELLULAR LOCATION: Plastid, chloroplast {ECO:0000255}.
CC   -!- ALTERNATIVE PRODUCTS:
CC       Event=Alternative splicing; Named isoforms=2;
CC       Name=1;
CC         IsoId=Q8H1E2-1; Sequence=Displayed;
CC       Name=2;
CC         IsoId=Q8H1E2-2; Sequence=VSP_058651;
CC         Note=May be due to a competing acceptor splice site. No
CC         experimental confirmation available. {ECO:0000305};
CC   -!- INDUCTION: By low temperature and high light.
CC       {ECO:0000269|PubMed:17925997}.
CC   -!- DISRUPTION PHENOTYPE: No visible phenotype under normal growth
CC       conditions and high-light conditions in short days.
CC       {ECO:0000269|PubMed:22140244}.
CC   -!- SIMILARITY: Belongs to the LDH/MDH superfamily. MDH type 2 family.
CC       {ECO:0000305}.
CC   -!- SEQUENCE CAUTION:
CC       Sequence=AAM63456.1; Type=Erroneous initiation; Note=Translation N-terminally extended.; Evidence={ECO:0000305};
DR   EMBL; AB019228; BAA96924.1; -; Genomic_DNA.
DR   EMBL; CP002688; AED97037.1; -; Genomic_DNA.
DR   EMBL; CP002688; AED97038.1; -; Genomic_DNA.
DR   EMBL; AY074329; AAL67025.1; -; mRNA.
DR   EMBL; AY150479; AAN13004.1; -; mRNA.
DR   EMBL; AK226364; BAE98512.1; -; mRNA.
DR   EMBL; AY086453; AAM63456.1; ALT_INIT; mRNA.
DR   RefSeq; NP_568875.2; NM_125218.4. [Q8H1E2-2]
DR   RefSeq; NP_851214.1; NM_180883.3. [Q8H1E2-1]
DR   SMR; Q8H1E2; -.
DR   IntAct; Q8H1E2; 1.
DR   STRING; 3702.AT5G58330.1; -.
DR   PaxDb; Q8H1E2; -.
DR   PRIDE; Q8H1E2; -.
DR   EnsemblPlants; AT5G58330.1; AT5G58330.1; AT5G58330. [Q8H1E2-1]
DR   EnsemblPlants; AT5G58330.2; AT5G58330.2; AT5G58330. [Q8H1E2-2]
DR   GeneID; 835945; -.
DR   Gramene; AT5G58330.1; AT5G58330.1; AT5G58330. [Q8H1E2-1]
DR   Gramene; AT5G58330.2; AT5G58330.2; AT5G58330. [Q8H1E2-2]
DR   KEGG; ath:AT5G58330; -.
DR   Araport; AT5G58330; -.
DR   TAIR; locus:2161188; AT5G58330.
DR   eggNOG; KOG1496; Eukaryota.
DR   eggNOG; COG0039; LUCA.
DR   HOGENOM; HOG000220953; -.
DR   InParanoid; Q8H1E2; -.
DR   KO; K00051; -.
DR   OrthoDB; 1118998at2759; -.
DR   PhylomeDB; Q8H1E2; -.
DR   BioCyc; ARA:AT5G58330-MONOMER; -.
DR   BRENDA; 1.1.1.40; 399.
DR   PRO; PR:Q8H1E2; -.
DR   Proteomes; UP000006548; Chromosome 5.
DR   ExpressionAtlas; Q8H1E2; baseline and differential.
DR   GO; GO:0048046; C:apoplast; IDA:TAIR.
DR   GO; GO:0009507; C:chloroplast; IDA:TAIR.
DR   GO; GO:0009941; C:chloroplast envelope; IDA:TAIR.
DR   GO; GO:0009570; C:chloroplast stroma; IDA:TAIR.
DR   GO; GO:0005739; C:mitochondrion; IDA:TAIR.
DR   GO; GO:0009579; C:thylakoid; IDA:TAIR.
DR   GO; GO:0030060; F:L-malate dehydrogenase activity; IBA:GO_Central.
DR   GO; GO:0046554; F:malate dehydrogenase (NADP+) activity; IEA:UniProtKB-EC.
DR   GO; GO:0008746; F:NAD(P)+ transhydrogenase activity; IMP:TAIR.
DR   GO; GO:0005975; P:carbohydrate metabolic process; IEA:InterPro.
DR   GO; GO:0006108; P:malate metabolic process; IBA:GO_Central.
DR   GO; GO:0006734; P:NADH metabolic process; IBA:GO_Central.
DR   GO; GO:0006107; P:oxaloacetate metabolic process; IBA:GO_Central.
DR   GO; GO:0051775; P:response to redox state; IMP:TAIR.
DR   GO; GO:0006099; P:tricarboxylic acid cycle; IBA:GO_Central.
DR   Gene3D; 3.90.110.10; -; 1.
DR   HAMAP; MF_01517; Malate_dehydrog_2; 1.
DR   InterPro; IPR022383; Lactate/malate_DH_C.
DR   InterPro; IPR001236; Lactate/malate_DH_N.
DR   InterPro; IPR015955; Lactate_DH/Glyco_Ohase_4_C.
DR   InterPro; IPR001252; Malate_DH_AS.
DR   InterPro; IPR011273; Malate_DH_NADP-dep_pln.
DR   InterPro; IPR010945; Malate_DH_type2.
DR   InterPro; IPR036291; NAD(P)-bd_dom_sf.
DR   PANTHER; PTHR23382; PTHR23382; 1.
DR   Pfam; PF02866; Ldh_1_C; 1.
DR   Pfam; PF00056; Ldh_1_N; 1.
DR   SUPFAM; SSF51735; SSF51735; 1.
DR   SUPFAM; SSF56327; SSF56327; 1.
DR   TIGRFAMs; TIGR01757; Malate-DH_plant; 1.
```

```
DR   TIGRFAMs; TIGR01759; MalateDH-SF1; 1.
DR   PROSITE; PS00068; MDH; 1.
PE   2: Evidence at transcript level;
KW   Alternative splicing; Chloroplast; Complete proteome; Disulfide bond;
KW   Isopeptide bond; NADP; Oxidoreductase; Plastid; Reference proteome;
KW   Transit peptide; Ubl conjugation.
FT   TRANSIT       1     52       Chloroplast. {ECO:0000255}.
FT   CHAIN        53    443       Malate dehydrogenase [NADP],
FT                                chloroplastic.
FT                                /FTId=PRO_0000438325.
FT   NP_BIND     106    112       NADP. {ECO:0000250|UniProtKB:P11708}.
FT   NP_BIND     224    226       NADP. {ECO:0000250|UniProtKB:P11708}.
FT   ACT_SITE    282    282       Proton acceptor.
FT                                {ECO:0000250|UniProtKB:P11708}.
FT   BINDING     187    187       Substrate.
FT                                {ECO:0000250|UniProtKB:P11708}.
FT   BINDING     193    193       Substrate.
FT                                {ECO:0000250|UniProtKB:P11708}.
FT   BINDING     200    200       NADP. {ECO:0000250|UniProtKB:P11708}.
FT   BINDING     207    207       NADP. {ECO:0000250|UniProtKB:P11708}.
FT   BINDING     226    226       Substrate.
FT                                {ECO:0000250|UniProtKB:P11708}.
FT   BINDING     257    257       Substrate.
FT                                {ECO:0000250|UniProtKB:P11708}.
FT   SITE         77     77       Activation of NADP-MDH.
FT                                {ECO:0000250|UniProtKB:P21528}.
FT   SITE         82     82       Activation of NADP-MDH.
FT                                {ECO:0000250|UniProtKB:P21528}.
FT   DISULFID     77     82       In oxidized inactive NADP-MDH.
FT                                {ECO:0000250|UniProtKB:P21528}.
FT   DISULFID    418    430       In oxidized inactive NADP-MDH.
FT                                {ECO:0000250}.
FT   CROSSLNK    213    213       Glycyl lysine isopeptide (Lys-Gly)
FT                                (interchain with G-Cter in ubiquitin).
FT                                {ECO:0000250|UniProtKB:P93819}.
FT   VAR_SEQ      59     59       Missing (in isoform 2).
FT                                /FTId=VSP_058651.
FT   CONFLICT    380    380       P -> L (in Ref. 5; AAM63456).
FT                                {ECO:0000305}.
FT   CONFLICT    414    414       A -> P (in Ref. 3; AAL67025).
FT                                {ECO:0000305}.
SQ   SEQUENCE   443 AA;  48316 MW;  29BE4C23B9E94F41 CRC64;
     MAMAELSTPK TTSPFLNSSS RLRLSSKLHL SNHFRHLLLP PLHTTTPNSK ISCSVSQNSQ
     APVAVQENGL VKTKKECYGV FCLTYDLKAE EETRSWKKLI NIAVSGAAGM ISNHLLFKLA
     SGEVFGPDQP IALKLLGSER SIQALEGVAM ELEDSLFPLL REVDIGTDPN EVFQDVEWAI
     LIGAKPRGPG MERADLLDIN GQIFAEQGKA LNKAASPNVK VLVVGNPCNT NALICLKNAP
     NIPAKNFHAL TRLDENRAKC QLALKAGVFY DKVSNMTIWG NHSTTQVPDF LNARINGLPV
     KEVITDHKWL EEGFTESVQK RGGLLIQKWG RSSAASTAVS IVDAIKSLVT PTPEGDWFST
     GVYTDGNPYG IEEGLVFSMP CRSKGDGDYE LVKDVEIDDY LRQRIAKSEA ELLAEKRCVA
     HLTGEGIAYC DLGPVDTMLP GEV
```

Mascot: http://www.matrixscience.com/

MASCOT Search Results

Protein View: SPSA1_CRAPL

Probable sucrose-phosphate synthase 1 OS=Craterostigma plantagineum OX=4153 GN=SPS1 PE=2 SV=1

**Database:**	SwissProt
**Score:**	312
**Expect:**	2.5e-27
**Monoisotopic mass (M_r):**	118945
**Calculated pI:**	6.08
**Taxonomy:**	Craterostigma plantagineum

Sequence similarity is available as an NCBI BLAST search of SPSA1_CRAPL against nr.

Search parameters

**Enzyme:**	Trypsin: cuts C-term side of KR unless next residue is P.
**Mass values searched:**	128
**Mass values matched:**	81

Protein sequence coverage: 87%

Matched peptides shown in ***bold red***.

```
   1 MAGNDWINSY LEAILDVGPG IDEAKGSLLL RERGRFSPTR YFVEEVVSGF
  51 DETDLHRSWI RAQATRSPQE RNTRLENMCW RIWNLARQKK QLENEEAQRM
 101 AKRRLERERG RREAVADMSE DLSEGEKGDI VVDHSHHGES NRGRLPRINS
 151 VDTMEAWMNQ QKGKKLYIVL ISLHGLIRGE NMELGRDSDT GGQVKYVVEL
 201 ARALGSMPGV YRVDLLTRQV SSPEVDWSYG EPTEMLPPRN SENMMDEMGE
 251 SSGSYIVRIP FGPKDKYVAK ELLWPHIPEF VDGALGHIIQ MSKVLGEQIG
 301 NGHPIWPAAI HGHYADAGDS AALLSGALNV PMLFTGHSLG RDKLEQLLRQ
 351 GRLSRDEINS TYKIMRRIEA EELSLDASEM VITSTRQEIE EQWRLYDGFD
 401 PILERKLRAR IKRNVSCYGR FMPRMMVIPP GMEFHHIVPH DGDLDAEPEF
 451 NEDSKSPDPH IWTEIMRFFS NPRKPMILAL ARPDPKKNLT TLVKAFGECK
 501 PLRELANLTL IMGNRDNIDE MSGTNASVLL SILKMIDKYD LYGLVAYPKH
 551 HKQSDVPDIY RLAAKTKGVF INPAFIEPFG LTLIEAAAHG LPIVATKNGG
 601 PVDIHRVLDN GILVDPHNQE SIADALLKLV AEKHLWAKCR ANGLKNIHLF
 651 SWPEHCKSYL SKLASCKPRQ PRWLRNEEDD DENSESDSPS DSLRDIQDIS
 701 LNLKFSFDGD KNESREKGGG SHPDDRASKI ENAVLEWSKG VAKGPQRSMS
 751 IEKGEHNSNA GKFPALRRRK IMFVIAVDCK PSAGLSESVR KVFAAVENER
 801 AEGSVGFILA TSFNISEIRH FLVSEKLNPT DFDAFICNSG GDLYYSSHHS
 851 EDNPFVVDLY YHSQIEYRWG GEGLRKTLVR WAASITDKKG EKEEHVIIED
 901 EETSADYCYS FKVQKPNVVP PVKEARKVMR IQALRCHVVY CQNGNKINVI
 951 PVLASRAQAL RYLYLRWGME LSKTVVVVGE SGDTDYEEML GGVHKTVVLS
1001 GVCTTATNLL HANRSYPLAD VVCFDDLNIF KTHNEECSST DLRALLEEHG
1051 AFKA
```

Unformatted sequence string: 1054 residues (for pasting into other applications).

Sort by (•) residue number ( ) increasing mass ( ) decreasing mass
Show (•) matched peptides only ( ) predicted peptides also

Start – End	Observed	Mr(expt)	Mr(calc)	Delta	M	Peptide
1 – 25	2691.2970	2690.2897	2690.2897	-0.0000	0	-.MAGNDWINSYLEAILDVGPGIDEAK.G
26 – 31	658.4246	657.4173	657.4173	-0.0000	0	K.GSLLLR.E
34 – 40	820.0000	818.9927	819.4351	-0.4424	1	R.GRFSPTR.Y
36 – 40	607.0000	605.9927	606.3126	-0.3198	0	R.FSPTR.Y
41 – 57	2041.9501	2040.9428	2040.9429	-0.0001	0	R.YFVEEVVSGFDETDLHR.S
75 – 81	951.4175	950.4102	950.4102	0.0000	0	R.LENMCWR.I
82 – 87	772.4464	771.4391	771.4391	-0.0000	0	R.IWNLAR.Q
91 – 99	1116.5280	1115.5207	1115.5207	0.0000	0	K.QLENEEAQR.M
113 – 127	1609.6897	1608.6824	1608.6825	-0.0000	0	R.EAVADMSEDLSEGEK.G
128 – 142	1658.7629	1657.7556	1657.7557	-0.0001	0	K.GDIVVDHSHHGESNR.G
143 – 147	598.0000	596.9927	597.3711	-0.3783	1	R.GRLPR.I
148 – 162	1794.8149	1793.8076	1793.8076	-0.0000	0	R.INSVDTMEAWMNQQK.G
163 – 164	203.0000	201.9927	203.1270	-1.1343	0	K.GK.K

Start – End	Observed	Mr(expt)	Mr(calc)	Delta	M	Peptide
166 – 178	1509.9515	1508.9442	1508.9442	-0.0000	0	K.LYIVLISLHGLIR.G
179 – 186	905.4145	904.4072	904.4072	-0.0000	0	R.GENMELGR.D
187 – 195	906.4163	905.4090	905.4091	-0.0000	0	R.DSDTGGQVK.Y
196 – 202	849.4828	848.4755	848.4756	-0.0001	0	K.YVVELAR.A
203 – 212	1050.5400	1049.5327	1049.5328	-0.0001	0	R.ALGSMPGVYR.V
213 – 218	716.4301	715.4228	715.4228	-0.0000	0	R.VDLLTR.Q
219 – 239	2404.1125	2403.1052	2403.1053	-0.0000	0	R.QVSSPEVDWSYGEPTEMLPPR.N
240 – 258	2135.8678	2134.8605	2134.8605	0.0000	0	R.NSENMMDEMGESSGSYIVR.I
259 – 264	658.3922	657.3849	657.3850	-0.0001	0	R.IPFGPK.D
271 – 293	2630.3799	2629.3726	2629.3726	-0.0000	0	K.ELLWPHIPEFVDGALGHIIQMSK.V
294 – 341	4888.4739	4887.4666	4887.4667	-0.0001	0	K.VLGEQIGNGHPIWPAAIHGHYADAGDSAALLSGALNVPMLFTGHSLGR.D
342 – 349	1015.0000	1013.9927	1013.5869	0.4058	1	R.DKLEQLLR.Q
344 – 349	771.0000	769.9927	770.4650	-0.4723	0	K.LEQLLR.Q
344 – 349	771.4723	770.4650	770.4650	0.0000	0	K.LEQLLR.Q
356 – 363	969.4523	968.4450	968.4451	-0.0000	0	R.DEINSTYK.I
368 – 386	2094.0270	2093.0197	2093.0198	-0.0001	0	R.IEAEELSLDASEMVITSTR.Q
387 – 394	1117.5272	1116.5199	1116.5200	-0.0000	0	R.QEIEEQWR.L
395 – 405	1337.6735	1336.6662	1336.6663	-0.0001	0	R.LYDGFDPILER.K
407 – 410	516.0000	514.9927	514.3339	0.6588	1	K.LRAR.I
411 – 412	259.0000	257.9927	259.1896	-1.1969	0	R.IK.R
414 – 420	798.3563	797.3490	797.3490	0.0000	0	R.NVSCYGR.F
425 – 455	3533.5810	3532.5737	3532.5738	-0.0000	0	R.MMVIPPGMEFHHIVPHDGDLDAEPEFNEDSK.S
456 – 467	1481.7205	1480.7132	1480.7133	-0.0000	0	K.SPDPHIWTEIMR.F
468 – 473	767.3835	766.3762	766.3762	0.0000	0	R.FFSNPR.K
474 – 486	1449.8610	1448.8537	1448.8537	0.0000	0	R.KPMILALARPDPK.K
488 – 494	788.4876	787.4803	787.4803	-0.0000	0	K.NLTTLVK.A
495 – 503	1020.5295	1019.5222	1019.5222	0.0000	0	K.AFGECKPLR.E
504 – 515	1344.7304	1343.7231	1343.7231	0.0000	0	R.ELANLTLIMGNR.D
516 – 534	2020.0267	2019.0194	2019.0194	0.0000	0	R.DNIDEMSGTNASVLLSILK.M
539 – 549	1301.6776	1300.6703	1300.6703	0.0000	0	K.YDLYGLVAYPK.H
553 – 561	1092.5320	1091.5247	1091.5247	-0.0000	0	K.QSDVPDIYR.L
568 – 597	3106.7338	3105.7265	3105.7267	-0.0001	0	K.GVFINPAFIEPFGLTLIEAAAHGLPIVATK.N
598 – 606	964.4959	963.4886	963.4886	-0.0000	0	K.NGGPVDIHR.V
607 – 628	2374.2612	2373.2539	2373.2540	-0.0000	0	R.VLDNGILVDPHNQESIADALLK.L
634 – 638	654.3722	653.3649	653.3649	0.0000	0	K.HLWAK.C
646 – 657	1510.7259	1509.7186	1509.7187	-0.0001	0	K.NIHLFSWPEHCK.S
663 – 669	774.4290	773.4217	773.4218	-0.0000	0	K.LASCKPR.Q
673 – 675	474.0000	472.9927	473.2750	-0.2823	0	R.WLR.N
676 – 694	2139.8068	2138.7995	2138.7996	-0.0000	0	R.NEEDDDENSESDSPSDSLR.D
695 – 704	1158.6364	1157.6291	1157.6292	-0.0001	0	R.DIQDISLNLK.F
705 – 711	815.3570	814.3497	814.3497	-0.0000	0	K.FSFDGDK.N
718 – 726	897.3809	896.3736	896.3737	-0.0000	0	K.GGGSHPDDR.A
730 – 739	1188.6259	1187.6186	1187.6186	0.0000	0	K.IENAVLEWSK.G
748 – 753	694.3440	693.3367	693.3367	0.0000	0	R.SMSIEK.G
748 – 753	695.0000	693.9927	693.3367	0.6560	0	R.SMSIEK.G
754 – 762	913.0000	911.9927	912.4049	-0.4122	0	K.GEHNSNAGK.F
754 – 762	913.4122	912.4049	912.4049	-0.0000	0	K.GEHNSNAGK.F
771 – 790	2122.1035	2121.0962	2121.0962	0.0000	0	K.IMFVIAVDCKPSAGLSESVR.K
792 – 800	1034.5265	1033.5192	1033.5192	-0.0000	0	K.VFAAVENER.A
801 – 819	2011.0494	2010.0421	2010.0422	-0.0001	0	R.AEGSVGFILATSFNISEIR.H
820 – 826	859.4672	858.4599	858.4599	-0.0000	0	R.HFLVSEK.L
827 – 868	4928.1845	4927.1772	4927.1773	-0.0001	0	K.LNPTDFDAFICNSGGDLYYSSHHSEDNPFVVDLYYHSQIEYR.W
869 – 875	774.3893	773.3820	773.3820	-0.0000	0	R.WGGEGLR.K
877 – 880	488.0000	486.9927	487.3118	-0.3191	0	K.TLVR.W
881 – 888	891.4570	890.4497	890.4498	-0.0000	0	R.WAASITDK.K
893 – 912	2407.0282	2406.0209	2406.0209	0.0000	0	K.EEHVIIEDEETSADYCYSFK.V
913 – 923	1204.7412	1203.7339	1203.7339	-0.0000	0	K.VQKPNVVPPVK.E
924 – 927	504.0000	502.9927	502.2863	0.7064	1	K.EARK.V
927 – 927	148.0000	146.9927	146.1055	0.8872	0	R.K.V
936 – 946	1264.5561	1263.5488	1263.5489	-0.0000	0	R.CHVVYCQNGNK.I
947 – 956	1081.6728	1080.6655	1080.6655	0.0000	0	K.INVIPVLASR.A
962 – 966	727.4137	726.4064	726.4064	-0.0000	0	R.YLYLR.W
967 – 973	850.4127	849.4054	849.4055	-0.0000	0	R.WGMELSK.T
974 – 995	2321.0965	2320.0892	2320.0893	-0.0001	0	K.TVVVVGESGDTDYEEMLGGVHK.T
996 – 1014	1970.0487	1969.0414	1969.0415	-0.0001	0	K.TVVLSGVCTTATNLLHANR.S
1015 – 1031	1958.9568	1957.9495	1957.9496	-0.0000	0	R.SYPLADVVCFDDLNIFK.T
1032 – 1043	1391.5856	1390.5783	1390.5783	0.0000	0	K.THNEECSSTDLR.A
1044 – 1053	1114.5891	1113.5818	1113.5818	-0.0000	0	R.ALLEEHGAFK.A

No match to: 113.0000, 128.0000, 166.0000, 179.0000, 187.0000, 196.0000, 213.0000, 219.0000, 240.0000, 271.0000, 294.0000, 344.0000, 356.0000, 368.0000, 387.0000, 395.0000, 414.0000, 425.0000, 456.0000, 468.0000, 495.0000, 539.0000, 553.0000, 568.0000, 634.0000, 646.0000, 663.0000, 676.0000, 705.0000, 718.0000, 730.0000, 748.0000, 754.0000, 792.0000, 801.0000, 827.0000, 869.0000, 881.0000, 893.0000, 936.0000, 947.0000, 962.0000, 967.0000, 974.0000, 996.0000, 1032.0000, 1044.0000

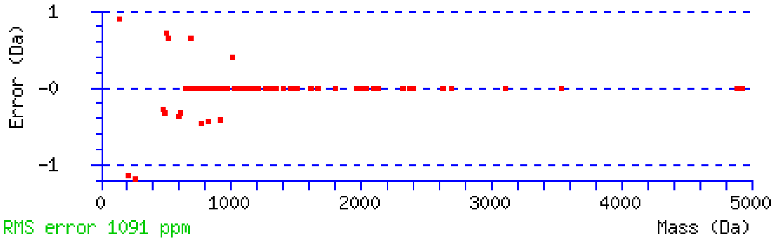

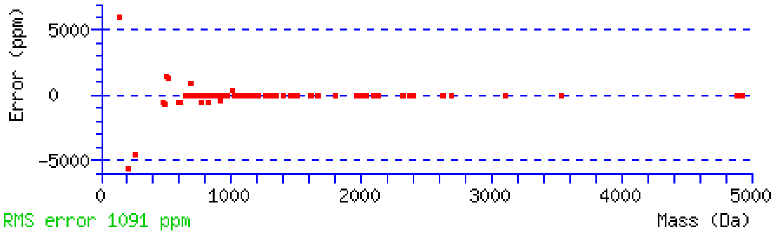


```
ID   SPSA1_CRAPL             Reviewed;        1054 AA.
AC   O04932;
DT   15-DEC-1998, integrated into UniProtKB/Swiss-Prot.
DT   01-JUL-1997, sequence version 1.
DT   31-JUL-2019, entry version 79.
DE   RecName: Full=Probable sucrose-phosphate synthase 1;
DE            EC=2.4.1.14;
DE   AltName: Full=UDP-glucose-fructose-phosphate glucosyltransferase 1;
GN   Name=SPS1;
OS   Craterostigma plantagineum (Blue gem) (Torenia plantagineum).
OC   Eukaryota; Viridiplantae; Streptophyta; Embryophyta; Tracheophyta;
OC   Spermatophyta; Magnoliopsida; eudicotyledons; Gunneridae;
OC   Pentapetalae; asterids; lamiids; Lamiales; Linderniaceae;
OC   Craterostigma.
OX   NCBI_TaxID=4153;
RN   [1]
RP   NUCLEOTIDE SEQUENCE [MRNA].
RX   PubMed=9306694; DOI=10.1104/pp.115.1.113;
RA   Ingram J., Chandler J.W., Gallagher L., Salamini F., Bartels D.;
RT   "Analysis of cDNA clones encoding sucrose-phosphate synthase in
RT   relation to sugar interconversions associated with dehydration in the
RT   resurrection plant Craterostigma plantagineum Hochst.";
RL   Plant Physiol. 115:113-121(1997).
CC   -!- FUNCTION: Plays a role in photosynthetic sucrose synthesis by
CC       catalyzing the rate-limiting step of sucrose biosynthesis from
CC       UDP-glucose and fructose- 6-phosphate. Involved in the regulation
CC       of carbon partitioning in the leaves of plants. May regulate the
CC       synthesis of sucrose and therefore play a major role as a limiting
CC       factor in the export of photoassimilates out of the leaf. Plays a
CC       role for sucrose availability that is essential for plant growth
CC       and fiber elongation (By similarity). {ECO:0000250}.
CC   -!- CATALYTIC ACTIVITY:
CC       Reaction=beta-D-fructose 6-phosphate + UDP-alpha-D-glucose = H(+)
CC         + sucrose 6(F)-phosphate + UDP; Xref=Rhea:RHEA:22172,
CC         ChEBI:CHEBI:15378, ChEBI:CHEBI:57634, ChEBI:CHEBI:57723,
CC         ChEBI:CHEBI:58223, ChEBI:CHEBI:58885; EC=2.4.1.14;
CC   -!- ACTIVITY REGULATION: Activity is regulated by phosphorylation and
CC       moderated by concentration of metabolites and light.
CC       {ECO:0000250}.
CC   -!- PATHWAY: Glycan biosynthesis; sucrose biosynthesis; sucrose from
CC       D-fructose 6-phosphate and UDP-alpha-D-glucose: step 1/2.
CC   -!- SUBUNIT: Homodimer or homotetramer. {ECO:0000250}.
CC   -!- SIMILARITY: Belongs to the glycosyltransferase 1 family.
CC       {ECO:0000305}.
DR   EMBL; Y11821; CAA72506.1; -; mRNA.
DR   PIR; T09833; T09833.
DR   CAZy; GT4; Glycosyltransferase Family 4.
DR   PRIDE; O04932; -.
DR   UniPathway; UPA00371; UER00545.
DR   GO; GO:0016157; F:sucrose synthase activity; IEA:InterPro.
DR   GO; GO:0046524; F:sucrose-phosphate synthase activity; IEA:UniProtKB-EC.
DR   GO; GO:0005986; P:sucrose biosynthetic process; IEA:UniProtKB-UniPathway.
DR   CDD; cd16419; HAD_SPS; 1.
DR   Gene3D; 3.40.50.1000; -; 1.
DR   InterPro; IPR001296; Glyco_trans_1.
DR   InterPro; IPR023214; HAD_sf.
DR   InterPro; IPR006380; SPP_N.
DR   InterPro; IPR035659; SPS_C.
DR   InterPro; IPR000368; Sucrose_synth.
DR   InterPro; IPR012819; SucrsPsyn_pln.
DR   Pfam; PF00534; Glycos_transf_1; 1.
DR   Pfam; PF05116; S6PP; 1.
DR   Pfam; PF00862; Sucrose_synth; 1.
```

```
DR   TIGRFAMs; TIGR02468; sucrsPsyn_pln; 1.
PE   2: Evidence at transcript level;
KW   Glycosyltransferase; Transferase.
FT   CHAIN         1   1054       Probable sucrose-phosphate synthase 1.
FT                                /FTId=PRO_0000204669.
SQ   SEQUENCE   1054 AA;  119020 MW;  58558A387AE78BC4 CRC64;
     MAGNDWINSY LEAILDVGPG IDEAKGSLLL RERGRFSPTR YFVEEVVSGF DETDLHRSWI
     RAQATRSPQE RNTRLENMCW RIWNLARQKK QLENEEAQRM AKRRLERERG RREAVADMSE
     DLSEGEKGDI VVDHSHHGES NRGRLPRINS VDTMEAWMNQ QKGKKLYIVL ISLHGLIRGE
     NMELGRDSDT GGQVKYVVEL ARALGSMPGV YRVDLLTRQV SSPEVDWSYG EPTEMLPPRN
     SENMMDEMGE SSGSYIVRIP FGPKDKYVAK ELLWPHIPEF VDGALGHIIQ MSKVLGEQIG
     NGHPIWPAAI HGHYADAGDS AALLSGALNV PMLFTGHSLG RDKLEQLLRQ GRLSRDEINS
     TYKIMRRIEA EELSLDASEM VITSTRQEIE EQWRLYDGFD PILERKLRAR IKRNVSCYGR
     FMPRMMVIPP GMEFHHIVPH DGDLDAEPEF NEDSKSPDPH IWTEIMRFFS NPRKPMILAL
     ARPDPKKNLT TLVKAFGECK PLRELANLTL IMGNRDNIDE MSGTNASVLL SILKMIDKYD
     LYGLVAYPKH HKQSDVPDIY RLAAKTKGVF INPAFIEPFG LTLIEAAAHG LPIVATKNGG
     PVDIHRVLDN GILVDPHNQE SIADALLKLV AEKHLWAKCR ANGLKNIHLF SWPEHCKSYL
     SKLASCKPRQ PRWLRNEEDD DENSESDSPS DSLRDIQDIS LNLKFSFDGD KNESREKGGG
     SHPDDRASKI ENAVLEWSKG VAKGPQRSMS IEKGEHNSNA GKFPALRRRK IMFVIAVDCK
     PSAGLSESVR KVFAAVENER AEGSVGFILA TSFNISEIRH FLVSEKLNPT DFDAFICNSG
     GDLYYSSHHS EDNPFVVDLY YHSQIEYRWG GEGLRKTLVR WAASITDKKG EKEEHVIIED
     EETSADYCYS FKVQKPNVVP PVKEARKVMR IQALRCHVVY CQNGNKINVI PVLASRAQAL
     RYLYLRWGME LSKTVVVVGE SGDTDYEEML GGVHKTVVLS GVCTTATNLL HANRSYPLAD
     VVCFDDLNIF KTHNEECSST DLRALLEEHG AFKA
```

Mascot: http://www.matrixscience.com/

MATRIX SCIENCE MASCOT Search Results

Protein View: RK23_CHLVU

50S ribosomal protein L23, chloroplastic OS=Chlorella vulgaris OX=3077 GN=rpl23 PE=3 SV=1

**Database:**	SwissProt
**Score:**	82
**Expect:**	0.00028
**Monoisotopic mass (M_r):**	10046
**Calculated pI:**	10.22
**Taxonomy:**	Chlorella vulgaris

Sequence similarity is available as an NCBI BLAST search of RK23_CHLVU against nr.

Search parameters

**Enzyme:**	Trypsin: cuts C-term side of KR unless next residue is P.
**Mass values searched:**	5
**Mass values matched:**	5

Protein sequence coverage: 63%

Matched peptides shown in ***bold red***.

```
 1 MMLDLVKYPV IRTEKTTRLV ENNQLSFDVD VRITKPQIRK IIEEFFNVKV
51 LAVNTHRPPR KTNRLGSKPS YKRVIVTVDS DVTLLK
```

Unformatted sequence string: 86 residues (for pasting into other applications).

Sort by ◉ residue number ○ increasing mass ○ decreasing mass
Show ◉ matched peptides only ○ predicted peptides also

Start – End	Observed	Mr(expt)	Mr(calc)	Delta	M	Peptide
19 – 32	1647.8336	1646.8263	1646.8264	-0.0001	0	R.LVENNQLSFDVDVR.I
41 – 49	1138.6142	1137.6069	1137.6070	-0.0001	0	K.IIEEFFNVK.V
50 – 60	1259.7331	1258.7258	1258.7258	-0.0000	0	K.VLAVNTHRPPR.K
65 – 72	879.4934	878.4861	878.4861	-0.0000	0	R.LGSKPSYK.R
74 – 86	1401.8199	1400.8126	1400.8127	-0.0000	0	R.VIVTVDSDVTLLK.-

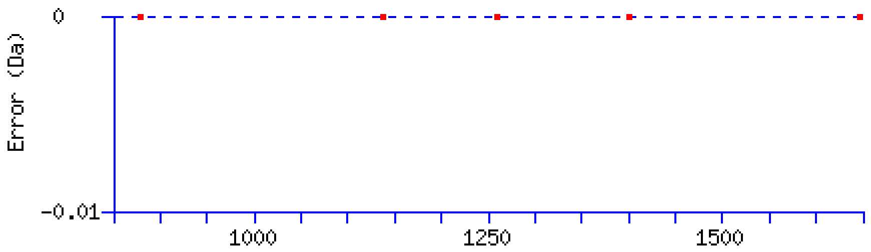

RMS error 0 ppm

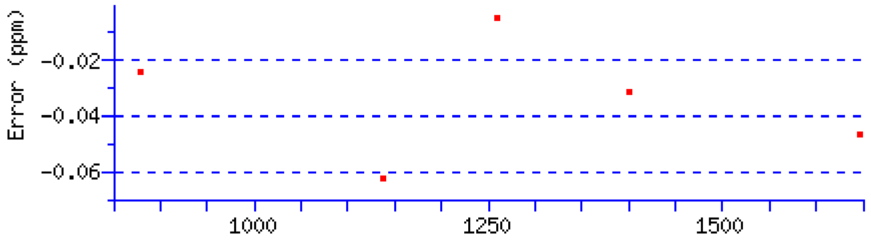

RMS error 0 ppm

```
ID   RK23_CHLVU              Reviewed;         86 AA.
AC   P56368;
```

```
DT   15-JUL-1998, integrated into UniProtKB/Swiss-Prot.
DT   15-JUL-1998, sequence version 1.
DT   08-MAY-2019, entry version 66.
DE   RecName: Full=50S ribosomal protein L23, chloroplastic;
GN   Name=rpl23;
OS   Chlorella vulgaris (Green alga).
OG   Plastid; Chloroplast.
OC   Eukaryota; Viridiplantae; Chlorophyta; Trebouxiophyceae; Chlorellales;
OC   Chlorellaceae; Chlorella clade; Chlorella.
OX   NCBI_TaxID=3077;
RN   [1]
RP   NUCLEOTIDE SEQUENCE [LARGE SCALE GENOMIC DNA].
RC   STRAIN=IAM C-27 / Tamiya;
RX   PubMed=9159184; DOI=10.1073/pnas.94.11.5967;
RA   Wakasugi T., Nagai T., Kapoor M., Sugita M., Ito M., Ito S.,
RA   Tsudzuki J., Nakashima K., Tsudzuki T., Suzuki Y., Hamada A., Ohta T.,
RA   Inamura A., Yoshinaga K., Sugiura M.;
RT   "Complete nucleotide sequence of the chloroplast genome from the green
RT   alga Chlorella vulgaris: the existence of genes possibly involved in
RT   chloroplast division.";
RL   Proc. Natl. Acad. Sci. U.S.A. 94:5967-5972(1997).
CC   -!- FUNCTION: Binds to 23S rRNA. {ECO:0000250}.
CC   -!- SUBUNIT: Part of the 50S ribosomal subunit. {ECO:0000250}.
CC   -!- SUBCELLULAR LOCATION: Plastid, chloroplast.
CC   -!- SIMILARITY: Belongs to the universal ribosomal protein uL23
CC       family. {ECO:0000305}.
DR   EMBL; AB001684; BAA58010.1; -; Genomic_DNA.
DR   PIR; T07362; T07362.
DR   RefSeq; NP_045934.1; NC_001865.1.
DR   SMR; P56368; -.
DR   PRIDE; P56368; -.
DR   GeneID; 809147; -.
DR   GO; GO:0009507; C:chloroplast; IEA:UniProtKB-SubCell.
DR   GO; GO:0005840; C:ribosome; IEA:UniProtKB-KW.
DR   GO; GO:0019843; F:rRNA binding; IEA:UniProtKB-UniRule.
DR   GO; GO:0003735; F:structural constituent of ribosome; IEA:InterPro.
DR   GO; GO:0006412; P:translation; IEA:UniProtKB-UniRule.
DR   Gene3D; 3.30.70.330; -; 1.
DR   HAMAP; MF_01369_B; Ribosomal_L23_B; 1.
DR   InterPro; IPR012677; Nucleotide-bd_a/b_plait_sf.
DR   InterPro; IPR012678; Ribosomal_L23/L15e_core_dom_sf.
DR   InterPro; IPR013025; Ribosomal_L25/23.
DR   Pfam; PF00276; Ribosomal_L23; 1.
DR   SUPFAM; SSF54189; SSF54189; 1.
PE   3: Inferred from homology;
KW   Chloroplast; Plastid; Ribonucleoprotein; Ribosomal protein;
KW   RNA-binding; rRNA-binding.
FT   CHAIN         1     86       50S ribosomal protein L23, chloroplastic.
FT                                /FTId=PRO_0000129447.
SQ   SEQUENCE   86 AA;  10052 MW;  3F42BDAEF7A9D8F8 CRC64;
     MMLDLVKYPV IRTEKTTRLV ENNQLSFDVD VRITKPQIRK IIEEFFNVKV LAVNTHRPPR
     KTNRLGSKPS YKRVIVTVDS DVTLLK
```

Mascot: http://www.matrixscience.com/

MASCOT Search Results

Protein View: 12KD_FRAAN

Auxin-repressed 12.5 kDa protein OS=Fragaria ananassa OX=3747 PE=2 SV=1

**Database:**	SwissProt
**Score:**	77
**Expect:**	0.00086
**Monoisotopic mass (M_r):**	12408
**Calculated pI:**	9.10
**Taxonomy:**	Fragaria x ananassa

Sequence similarity is available as an NCBI BLAST search of 12KD_FRAAN against nr.

Search parameters

**Enzyme:**	Trypsin: cuts C-term side of KR unless next residue is P.
**Mass values searched:**	4
**Mass values matched:**	4

Protein sequence coverage: 64%

Matched peptides shown in ***bold red***.

```
  1 MVLLDKLWDD IVAGPQPERG LGMLRKVPQP LNLKDEGESS KITMPTTPTT
 51 PVTPTTPISA RKDNVWRSVF HPGSNLSSKT MGNQVFDSPQ PNSPTVYDWM
101 YSGETRSKHH R
```

Unformatted sequence string: 111 residues (for pasting into other applications).

Sort by ◉ residue number ○ increasing mass ○ decreasing mass
Show ◉ matched peptides only ○ predicted peptides also

Start – End	Observed	Mr(expt)	Mr(calc)	Delta	M	Peptide
7 – 19	1495.7539	1494.7466	1494.7467	-0.0001	0	K.LWDDIVAGPQPER.G
42 – 61	2083.1103	2082.1030	2082.1031	-0.0001	0	K.ITMPTTPTTPVTPTTPISAR.K
68 – 79	1259.6378	1258.6305	1258.6306	-0.0001	0	R.SVFHPGSNLSSK.T
80 – 106	3107.3509	3106.3436	3106.3437	-0.0001	0	K.TMGNQVFDSPQPNSPTVYDWMYSGETR.S

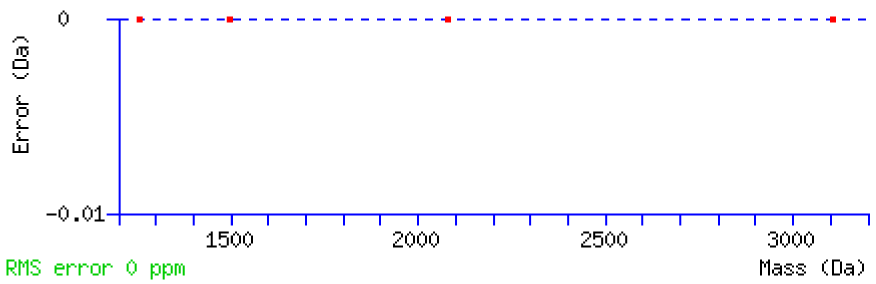

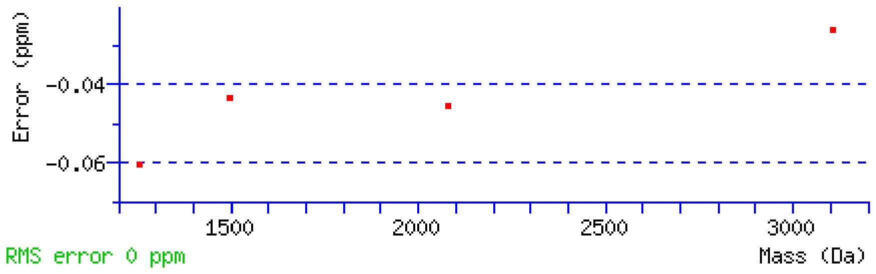


```
ID   12KD_FRAAN              Reviewed;         111 AA.
AC   Q05349;
DT   01-OCT-1996, integrated into UniProtKB/Swiss-Prot.
```

```
DT   01-OCT-1996, sequence version 1.
DT   31-JUL-2019, entry version 43.
DE   RecName: Full=Auxin-repressed 12.5 kDa protein;
OS   Fragaria ananassa (Strawberry) (Fragaria chiloensis x Fragaria
OS   virginiana).
OC   Eukaryota; Viridiplantae; Streptophyta; Embryophyta; Tracheophyta;
OC   Spermatophyta; Magnoliopsida; eudicotyledons; Gunneridae;
OC   Pentapetalae; rosids; fabids; Rosales; Rosaceae; Rosoideae;
OC   Potentilleae; Fragariinae; Fragaria.
OX   NCBI_TaxID=3747;
RN   [1]
RP   NUCLEOTIDE SEQUENCE [MRNA].
RC   STRAIN=cv. Ozark Beauty; TISSUE=Flower;
RX   PubMed=2101687; DOI=10.1007/BF00018554;
RA   Reddy A.S.N., Poovaiah B.W.;
RT   "Molecular cloning and sequencing of a cDNA for an auxin-repressed
RT   mRNA: correlation between fruit growth and repression of the auxin-
RT   regulated gene.";
RL   Plant Mol. Biol. 14:127-136(1990).
CC   -!- INDUCTION: Repressed by exogenous auxin.
CC   -!- SIMILARITY: Belongs to the DRM1/ARP family. {ECO:0000305}.
DR   EMBL; X52429; CAA36676.1; -; mRNA.
DR   EMBL; L44142; AAA73872.1; -; mRNA.
DR   PIR; S11850; S11850.
DR   GO; GO:0009734; P:auxin-activated signaling pathway; IEA:UniProtKB-KW.
DR   InterPro; IPR008406; DRM/ARP.
DR   PANTHER; PTHR33565; PTHR33565; 1.
DR   Pfam; PF05564; Auxin_repressed; 1.
PE   2: Evidence at transcript level;
KW   Auxin signaling pathway.
FT   CHAIN         1    111       Auxin-repressed 12.5 kDa protein.
FT                                /FTId=PRO_0000064348.
FT   COMPBIAS     43     57       Pro/Thr-rich.
SQ   SEQUENCE   111 AA;  12416 MW;  E44CACBADE6F3C51 CRC64;
     MVLLDKLWDD IVAGPQPERG LGMLRKVPQP LNLKDEGESS KITMPTTPTT PVTPTTPISA
     RKDNVWRSVF HPGSNLSSKT MGNQVFDSPQ PNSPTVYDWM YSGETRSKHH R
```

Mascot: http://www.matrixscience.com/

MATRIX SCIENCE MASCOT Search Results

Protein View: ARP_ARATH

DNA-(apurinic or apyrimidinic site) lyase, chloroplastic OS=Arabidopsis thaliana OX=3702 GN=ARP PE=1 SV=2

**Database:**	SwissProt
**Score:**	92
**Expect:**	2.8e-05
**Monoisotopic mass (M_r):**	60223
**Calculated pI:**	9.11
**Taxonomy:**	Arabidopsis thaliana

Sequence similarity is available as an NCBI BLAST search of ARP_ARATH against nr.

Search parameters

**Enzyme:**	Trypsin: cuts C-term side of KR unless next residue is P.
**Mass values searched:**	11
**Mass values matched:**	9

Protein sequence coverage: 28%

Matched peptides shown in ***bold red***.

```
  1 MNNVLQFGLQ SSAIYVAKFL VVPLRSLRVG SSFVGVGVGT RSFNKRLMSN
 51 ATAFSINNSK RKELKIPGAA IDQNCHQMGS DTDRDEMGTL QDDRKEIEAM
101 TVQELRSTLR KLGVPVKGRK QELISTLRLH MDSNLPDQKE TSSSTRSDSV
151 TIKRKISNRE EPTEDECTNS EAYDIEHGEK RVKQSTEKNL KAKVSAKAIA
201 KEQKSLMRTG KQQIQSKEET SSTISSELLK TEEIISSPSQ SEPWTVLAHK
251 KPQKDWKAYN PKTMRPPPLP EGTKCVKVMT WNVNGLRGLL KFESFSALQL
301 AQRENFDILC LQETKLQVKD VEEIKKTLID GYDHSFWSCS VSKLGYSGTA
351 IISRIKPLSV RYGTGLSGHD TEGRIVTAEF DSFYLINTYV PNSGDGLKRL
401 SYRIEEWDRT LSNHIKELEK SKPVVLTGDL NCAHEEIDIF NPAGNKRSAG
451 FTIEERQSFG ANLLDKGFVD TFRKQHPGVV GYTYWGYRHG GRKTNKGWRL
501 DYFLVSQSIA ANVHDSYILP DINGSDHCPI GLILKL
```

Unformatted sequence string: 536 residues (for pasting into other applications).

Sort by ◉ residue number ○ increasing mass ○ decreasing mass
Show ◉ matched peptides only ○ predicted peptides also

Start – End	Observed	Mr(expt)	Mr(calc)	Delta	M	Peptide
26 – 28	375.0000	373.9927	374.2278	-0.2350	0	R.SLR.V
66 – 84	2028.8862	2027.8789	2027.8789	0.0000	0	K.IPGAAIDQNCHQMGSDTDR.D
118 – 119	231.0000	229.9927	231.1331	-1.1404	0	K.GR.K
160 – 180	2424.9619	2423.9546	2423.9547	-0.0000	0	R.EEPTEDECTNSEAYDIEHGEK.R
231 – 250	2239.1241	2238.1168	2238.1168	0.0000	0	K.TEEIISSPSQSEPWTVLAHK.K
251 – 254	500.0000	498.9927	499.3118	-0.3191	0	K.KPQK.D
375 – 398	2663.3239	2662.3166	2662.3167	-0.0000	0	R.IVTAEFDSFYLINTYVPNSGDGLK.R
421 – 446	2781.3875	2780.3802	2780.3803	-0.0001	0	K.SKPVVLTGDLNCAHEEIDIFNPAGNK.R
500 – 535	3941.0153	3940.0080	3940.0081	-0.0000	0	R.LDYFLVSQSIAANVHDSYILPDINGSDHCPIGLILK.L

No match to: 160.0000, 421.0000

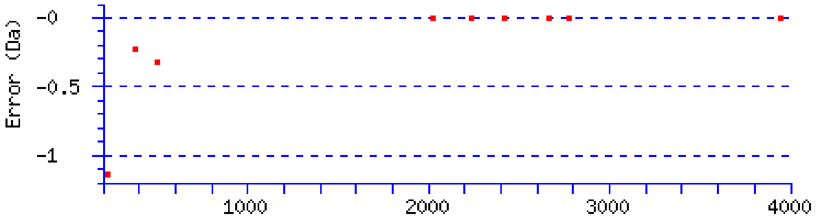

RMS error 1679 ppm

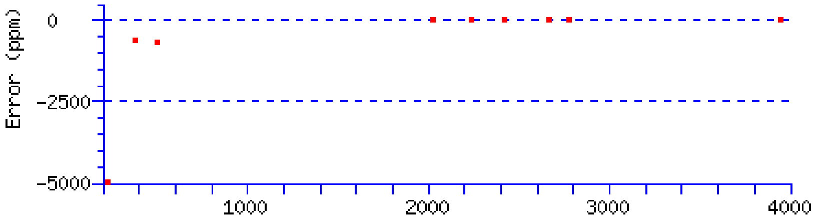

RMS error 1679 ppm

```
ID   ARP_ARATH                Reviewed;         536 AA.
AC   P45951;
DT   01-NOV-1995, integrated into UniProtKB/Swiss-Prot.
DT   30-MAY-2000, sequence version 2.
DT   31-JUL-2019, entry version 159.
DE   RecName: Full=DNA-(apurinic or apyrimidinic site) lyase, chloroplastic {ECO:0000303|PubMed:7512729};
DE            EC=4.2.99.18 {ECO:0000255|PROSITE-ProRule:PRU00764};
DE   AltName: Full=Apurinic endonuclease-redox protein;
GN   Name=ARP {ECO:0000303|PubMed:7512729}; Synonyms=REF;
GN   OrderedLocusNames=At2g41460 {ECO:0000312|Araport:AT2G41460};
GN   ORFNames=T26J13.5 {ECO:0000312|EMBL:AAC23731.1};
OS   Arabidopsis thaliana (Mouse-ear cress).
OC   Eukaryota; Viridiplantae; Streptophyta; Embryophyta; Tracheophyta;
OC   Spermatophyta; Magnoliopsida; eudicotyledons; Gunneridae;
OC   Pentapetalae; rosids; malvids; Brassicales; Brassicaceae; Camelineae;
OC   Arabidopsis.
OX   NCBI_TaxID=3702;
RN   [1]
RP   NUCLEOTIDE SEQUENCE [LARGE SCALE GENOMIC DNA].
RC   STRAIN=cv. Columbia;
RX   PubMed=10617197; DOI=10.1038/45471;
RA   Lin X., Kaul S., Rounsley S.D., Shea T.P., Benito M.-I., Town C.D.,
RA   Fujii C.Y., Mason T.M., Bowman C.L., Barnstead M.E., Feldblyum T.V.,
RA   Buell C.R., Ketchum K.A., Lee J.J., Ronning C.M., Koo H.L.,
RA   Moffat K.S., Cronin L.A., Shen M., Pai G., Van Aken S., Umayam L.,
RA   Tallon L.J., Gill J.E., Adams M.D., Carrera A.J., Creasy T.H.,
RA   Goodman H.M., Somerville C.R., Copenhaver G.P., Preuss D.,
RA   Nierman W.C., White O., Eisen J.A., Salzberg S.L., Fraser C.M.,
RA   Venter J.C.;
RT   "Sequence and analysis of chromosome 2 of the plant Arabidopsis
RT   thaliana.";
RL   Nature 402:761-768(1999).
RN   [2]
RP   GENOME REANNOTATION.
RC   STRAIN=cv. Columbia;
RX   PubMed=27862469; DOI=10.1111/tpj.13415;
RA   Cheng C.Y., Krishnakumar V., Chan A.P., Thibaud-Nissen F., Schobel S.,
RA   Town C.D.;
RT   "Araport11: a complete reannotation of the Arabidopsis thaliana
RT   reference genome.";
RL   Plant J. 89:789-804(2017).
RN   [3]
RP   NUCLEOTIDE SEQUENCE [MRNA] OF 10-536, FUNCTION, AND TISSUE
RP   SPECIFICITY.
RC   STRAIN=cv. Columbia; TISSUE=Callus;
RX   PubMed=7512729; DOI=10.1073/pnas.91.8.3299;
RA   Babiychuk E., Kushnir S., van Montagu M., Inze D.;
RT   "The Arabidopsis thaliana apurinic endonuclease Arp reduces human
RT   transcription factors Fos and Jun.";
RL   Proc. Natl. Acad. Sci. U.S.A. 91:3299-3303(1994).
RN   [4]
RP   FUNCTION, SUBCELLULAR LOCATION, AND DISRUPTION PHENOTYPE.
RX   PubMed=19372224; DOI=10.1074/jbc.M109.008342;
RA   Gutman B.L., Niyogi K.K.;
RT   "Evidence for base excision repair of oxidative DNA damage in
RT   chloroplasts of Arabidopsis thaliana.";
RL   J. Biol. Chem. 284:17006-17012(2009).
RN   [5]
RP   FUNCTION, MUTAGENESIS OF ARG-354, AND DISRUPTION PHENOTYPE.
```

```
RX   PubMed=19172180; DOI=10.1371/journal.pone.0004297;
RA   Murphy T.M., Belmonte M., Shu S., Britt A.B., Hatteroth J.;
RT   "Requirement for abasic endonuclease gene homologues in Arabidopsis
RT   seed development.";
RL   PLoS ONE 4:E4297-E4297(2009).
RN   [6]
RP   FUNCTION, AND DISRUPTION PHENOTYPE.
RX   PubMed=21781197; DOI=10.1111/j.1365-313X.2011.04720.x;
RA   Cordoba-Canero D., Roldan-Arjona T., Ariza R.R.;
RT   "Arabidopsis ARP endonuclease functions in a branched base excision
RT   DNA repair pathway completed by LIG1.";
RL   Plant J. 68:693-702(2011).
RN   [7]
RP   FUNCTION, TISSUE SPECIFICITY, CATALYTIC ACTIVITY, COFACTOR, AND
RP   DNA-BINDING.
RX   PubMed=25228464; DOI=10.1093/nar/gku834;
RA   Lee J., Jang H., Shin H., Choi W.L., Mok Y.G., Huh J.H.;
RT   "AP endonucleases process 5-methylcytosine excision intermediates
RT   during active DNA demethylation in Arabidopsis.";
RL   Nucleic Acids Res. 42:11408-11418(2014).
RN   [8]
RP   FUNCTION, CATALYTIC ACTIVITY, AND DISRUPTION PHENOTYPE.
RX   PubMed=25569774; DOI=10.1371/journal.pgen.1004905;
RA   Li Y., Cordoba-Canero D., Qian W., Zhu X., Tang K., Zhang H.,
RA   Ariza R.R., Roldan-Arjona T., Zhu J.K.;
RT   "An AP endonuclease functions in active DNA dimethylation and gene
RT   imprinting in Arabidopsis.";
RL   PLoS Genet. 11:E1004905-E1004905(2015).
CC   -!- FUNCTION: Repairs oxidative DNA damages, seems also to act as a
CC       redox factor (PubMed:7512729). Is multifunctional and may be
CC       involved both in DNA repair and in the regulation of transcription
CC       (PubMed:7512729). Exhibits apurinic/apyrimidinic (AP) endonuclease
CC       activity (PubMed:25569774, PubMed:21781197, PubMed:25228464).
CC       Catalyzes the conversion of 3'-phosphor-alpha,beta-unsaturated
CC       aldehyde (3'-PUA) to 3'-OH (PubMed:25228464). May be involved in
CC       base excision repair in chloroplasts (PubMed:19372224). According
CC       to a report, has a significant in vitro 3'-phosphatase activity
CC       (PubMed:25228464). According to another report, has no in vitro
CC       3'-phosphatase activity (PubMed:25569774). Has a strong non-
CC       specific affinity to DNA (PubMed:25228464).
CC       {ECO:0000269|PubMed:19372224, ECO:0000269|PubMed:21781197,
CC       ECO:0000269|PubMed:25228464, ECO:0000269|PubMed:25569774,
CC       ECO:0000269|PubMed:7512729, ECO:0000305|PubMed:19172180}.
CC   -!- CATALYTIC ACTIVITY:
CC       Reaction=The C-O-P bond 3' to the apurinic or apyrimidinic site in
CC         DNA is broken by a beta-elimination reaction, leaving a 3'-
CC         terminal unsaturated sugar and a product with a terminal 5'-
CC         phosphate.; EC=4.2.99.18; Evidence={ECO:0000269|PubMed:25228464,
CC         ECO:0000269|PubMed:25569774};
CC   -!- COFACTOR:
CC       Name=Mg(2+); Xref=ChEBI:CHEBI:18420;
CC         Evidence={ECO:0000269|PubMed:25228464};
CC       Name=Mn(2+); Xref=ChEBI:CHEBI:29035;
CC         Evidence={ECO:0000250|UniProtKB:P27695};
CC       Note=Probably binds two magnesium or manganese ions per subunit.
CC       {ECO:0000250|UniProtKB:P27695};
CC   -!- SUBCELLULAR LOCATION: Plastid, chloroplast stroma, chloroplast
CC       nucleoid {ECO:0000269|PubMed:19372224}.
CC   -!- ALTERNATIVE PRODUCTS:
CC       Event=Alternative splicing; Named isoforms=1;
CC         Comment=A number of isoforms are produced. According to EST
CC         sequences.;
CC       Name=1;
CC         IsoId=P45951-1; Sequence=Displayed;
CC   -!- TISSUE SPECIFICITY: Expressed in the siliques, flowers, and stems
CC       (PubMed:7512729). A high level expression is seen in the leaves
CC       (PubMed:7512729). Expressed in both vegetative and reproductive
CC       organs (PubMed:25228464). {ECO:0000269|PubMed:25228464,
CC       ECO:0000269|PubMed:7512729}.
CC   -!- DISRUPTION PHENOTYPE: No visible phenotype (PubMed:19372224,
CC       PubMed:19172180, PubMed:21781197, PubMed:25569774). Loss of
CC       chloroplastic glycosylase-lyase/endonuclease activity
CC       (PubMed:19372224). Hypersensitivity to 5-fluorouracil
CC       (PubMed:21781197). Ape1l arp double mutants have no visible
CC       phenotype (PubMed:19172180). Ape2 arp double mutants have no
CC       visible phenotype (PubMed:19172180). {ECO:0000269|PubMed:19172180,
CC       ECO:0000269|PubMed:19372224, ECO:0000269|PubMed:21781197,
CC       ECO:0000269|PubMed:25569774}.
CC   -!- SIMILARITY: Belongs to the DNA repair enzymes AP/ExoA family.
CC       {ECO:0000305}.
DR   EMBL; AC004625; AAC23731.1; -; Genomic_DNA.
```

```
DR   EMBL; CP002685; AEC09984.1; -; Genomic_DNA.
DR   EMBL; X76912; CAA54234.1; -; mRNA.
DR   PIR; T02441; T02441.
DR   RefSeq; NP_181677.1; NM_129709.5. [P45951-1]
DR   SMR; P45951; -.
DR   STRING; 3702.AT2G41460.1; -.
DR   iPTMnet; P45951; -.
DR   PaxDb; P45951; -.
DR   PRIDE; P45951; -.
DR   EnsemblPlants; AT2G41460.1; AT2G41460.1; AT2G41460. [P45951-1]
DR   GeneID; 818744; -.
DR   Gramene; AT2G41460.1; AT2G41460.1; AT2G41460. [P45951-1]
DR   KEGG; ath:AT2G41460; -.
DR   Araport; AT2G41460; -.
DR   TAIR; locus:2060540; AT2G41460.
DR   eggNOG; KOG1294; Eukaryota.
DR   eggNOG; COG0708; LUCA.
DR   InParanoid; P45951; -.
DR   KO; K01142; -.
DR   OrthoDB; 1105625at2759; -.
DR   PhylomeDB; P45951; -.
DR   PRO; PR:P45951; -.
DR   Proteomes; UP000006548; Chromosome 2.
DR   ExpressionAtlas; P45951; baseline and differential.
DR   Genevisible; P45951; AT.
DR   GO; GO:0042644; C:chloroplast nucleoid; IDA:TAIR.
DR   GO; GO:0005634; C:nucleus; IBA:GO_Central.
DR   GO; GO:0008408; F:3'-5' exonuclease activity; IDA:TAIR.
DR   GO; GO:0140078; F:class I DNA-(apurinic or apyrimidinic site) endonuclease activity; IEA:UniProtKB-EC.
DR   GO; GO:0003677; F:DNA binding; IEA:InterPro.
DR   GO; GO:0003906; F:DNA-(apurinic or apyrimidinic site) endonuclease activity; IDA:TAIR.
DR   GO; GO:0008311; F:double-stranded DNA 3'-5' exodeoxyribonuclease activity; IBA:GO_Central.
DR   GO; GO:0004519; F:endonuclease activity; IEA:InterPro.
DR   GO; GO:0046872; F:metal ion binding; IEA:UniProtKB-KW.
DR   GO; GO:0016791; F:phosphatase activity; IDA:TAIR.
DR   GO; GO:0004528; F:phosphodiesterase I activity; IDA:TAIR.
DR   GO; GO:0006284; P:base-excision repair; IBA:GO_Central.
DR   GO; GO:0033683; P:nucleotide-excision repair, DNA incision; IDA:TAIR.
DR   GO; GO:0045893; P:positive regulation of transcription, DNA-templated; TAS:TAIR.
DR   Gene3D; 1.10.720.30; -; 1.
DR   Gene3D; 3.60.10.10; -; 1.
DR   InterPro; IPR004808; AP_endonuc_1.
DR   InterPro; IPR020847; AP_endonuclease_F1_BS.
DR   InterPro; IPR020848; AP_endonuclease_F1_CS.
DR   InterPro; IPR036691; Endo/exonu/phosph_ase_sf.
DR   InterPro; IPR005135; Endo/exonuclease/phosphatase.
DR   InterPro; IPR003034; SAP_dom.
DR   InterPro; IPR036361; SAP_dom_sf.
DR   PANTHER; PTHR22748; PTHR22748; 1.
DR   Pfam; PF03372; Exo_endo_phos; 1.
DR   Pfam; PF02037; SAP; 1.
DR   SMART; SM00513; SAP; 1.
DR   SUPFAM; SSF56219; SSF56219; 1.
DR   SUPFAM; SSF68906; SSF68906; 1.
DR   TIGRFAMs; TIGR00633; xth; 1.
DR   PROSITE; PS00726; AP_NUCLEASE_F1_1; 1.
DR   PROSITE; PS00727; AP_NUCLEASE_F1_2; 1.
DR   PROSITE; PS00728; AP_NUCLEASE_F1_3; 1.
DR   PROSITE; PS51435; AP_NUCLEASE_F1_4; 1.
DR   PROSITE; PS50800; SAP; 1.
PE   1: Evidence at protein level;
KW   Alternative splicing; Chloroplast; Complete proteome; DNA damage;
KW   DNA repair; Lyase; Magnesium; Metal-binding; Plastid;
KW   Reference proteome.
FT   CHAIN         1    536       DNA-(apurinic or apyrimidinic site)
FT                                lyase, chloroplastic.
FT                                /FTId=PRO_0000200018.
FT   DOMAIN       97    131       SAP. {ECO:0000255|PROSITE-
FT                                ProRule:PRU00186}.
FT   REGION        1    278       Highly charged; increases the affinity of
FT                                ARP for DNA.
FT   REGION      279    536       AP endonuclease.
FT   ACT_SITE    389    389       {ECO:0000250|UniProtKB:P27695}.
FT   ACT_SITE    527    527       Proton acceptor. {ECO:0000255|PROSITE-
FT                                ProRule:PRU00764}.
FT   METAL       282    282       Magnesium or manganese.
FT                                {ECO:0000255|PROSITE-ProRule:PRU00764}.
FT   METAL       313    313       Magnesium or manganese.
FT                                {ECO:0000255|PROSITE-ProRule:PRU00764}.
FT   METAL       429    429       Magnesium or manganese.
FT                                {ECO:0000255|PROSITE-ProRule:PRU00764}.
```

```
FT   METAL       431    431       Magnesium or manganese.
FT                                {ECO:0000255|PROSITE-ProRule:PRU00764}.
FT   METAL       526    526       Magnesium or manganese.
FT                                {ECO:0000255|PROSITE-ProRule:PRU00764}.
FT   METAL       527    527       Magnesium or manganese.
FT                                {ECO:0000255|PROSITE-ProRule:PRU00764}.
FT   SITE        431    431       Transition state stabilizer.
FT                                {ECO:0000250|UniProtKB:P27695}.
FT   SITE        501    501       Important for catalytic activity.
FT                                {ECO:0000250|UniProtKB:P27695}.
FT   SITE        527    527       Interaction with DNA substrate.
FT                                {ECO:0000250|UniProtKB:P27695}.
FT   MUTAGEN     354    354       R->W: In arp-1; loss of activity.
FT                                {ECO:0000269|PubMed:19172180}.
SQ   SEQUENCE   536 AA;  60260 MW;  5C1FC17EA991D27B CRC64;
     MNNVLQFGLQ SSAIYVAKFL VVPLRSLRVG SSFVGVGVGT RSFNKRLMSN ATAFSINNSK
     RKELKIPGAA IDQNCHQMGS DTDRDEMGTL QDDRKEIEAM TVQELRSTLR KLGVPVKGRK
     QELISTLRLH MDSNLPDQKE TSSSTRSDSV TIKRKISNRE EPTEDECTNS EAYDIEHGEK
     RVKQSTEKNL KAKVSAKAIA KEQKSLMRTG KQQIQSKEET SSTISSELLK TEEIISSPSQ
     SEPWTVLAHK KPQKDWKAYN PKTMRPPPLP EGTKCVKVMT WNVNGLRGLL KFESFSALQL
     AQRENFDILC LQETKLQVKD VEEIKKTLID GYDHSFWSCS VSKLGYSGTA IISRIKPLSV
     RYGTGLSGHD TEGRIVTAEF DSFYLINTYV PNSGDGLKRL SYRIEEWDRT LSNHIKELEK
     SKPVVLTGDL NCAHEEIDIF NPAGNKRSAG FTIEERQSFG ANLLDKGFVD TFRKQHPGVV
     GYTYWGYRHG GRKTNKGWRL DYFLVSQSIA ANVHDSYILP DINGSDHCPI GLILKL
```

MATRIX SCIENCE MASCOT Search Results

Protein View: ACCO2_ARATH

1-aminocyclopropane-1-carboxylate oxidase 2 OS=Arabidopsis thaliana OX=3702 GN=ACO2 PE=1 SV=2

**Database:**	SwissProt
**Score:**	89
**Expect:**	4.8e-05
**Monoisotopic mass (M_r):**	36160
**Calculated pI:**	4.98
**Taxonomy:**	Arabidopsis thaliana

Sequence similarity is available as an NCBI BLAST search of ACCO2_ARATH against nr.

Search parameters

**Enzyme:**	Trypsin: cuts C-term side of KR unless next residue is P.
**Mass values searched:**	11
**Mass values matched:**	7

Protein sequence coverage: 49%

Matched peptides shown in ***bold red***.

```
  1 MEKNMKFPVV DLSKLNGEER DQTMALINEA CENWGFFEIV NHGLPHDLMD
 51 KIEKMTKDHY KTCQEQKFND MLKSKGLDNL ETEVEDVDWE STFYVRHLPQ
101 SNLNDISDVS DEYRTAMKDF GKRLENLAED LLDLLCENLG LEKGYLKKVF
151 HGTKGPTFGT KVSNYPPCPK PEMIKGLRAH TDAGGIILLF QDDKVSGLQL
201 LKDGDWIDVP PLNHSIVINL GDQLEVITNG KYKSVLHRVV TQQEGNRMSV
251 ASFYNPGSDA EISPATSLVE KDSEYPSFVF DDYMKLYAGV KFQPKEPRFA
301 AMKNASAVTE LNPTAAVETF
```

Unformatted sequence string: 320 residues (for pasting into other applications).

Sort by ◉ residue number ○ increasing mass ○ decreasing mass
Show ◉ matched peptides only ○ predicted peptides also

Start – End	Observed	Mr(expt)	Mr(calc)	Delta	M	Peptide
21 – 51	3587.6392	3586.6319	3586.6319	-0.0000	0	R.DQTMALINEACENWGFFEIVNHGLPHDLMDK.I
76 – 96	2516.1463	2515.1390	2515.1391	-0.0001	0	K.GLDNLETEVEDVDWESTFYVR.H
97 – 114	2101.9785	2100.9712	2100.9712	0.0000	0	R.HLPQSNLNDISDVSDEYR.T
124 – 143	2257.1631	2256.1558	2256.1559	-0.0000	0	R.LENLAEDLLDLLCENLGLEK.G
203 – 231	3171.6320	3170.6247	3170.6248	-0.0001	0	K.DGDWIDVPPLNHSIVINLGDQLEVITNGK.Y
248 – 271	2500.1911	2499.1838	2499.1839	-0.0001	0	R.MSVASFYNPGSDAEISPATSLVEK.D
272 – 285	1742.7254	1741.7181	1741.7182	-0.0000	0	K.DSEYPSFVFDDYMK.L

No match to: 124.0000, 203.0000, 248.0000, 272.0000

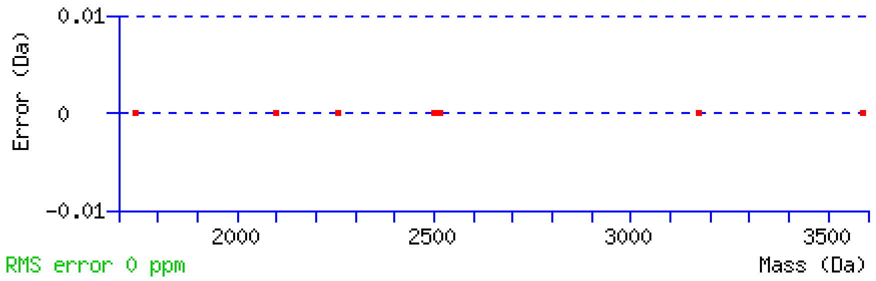

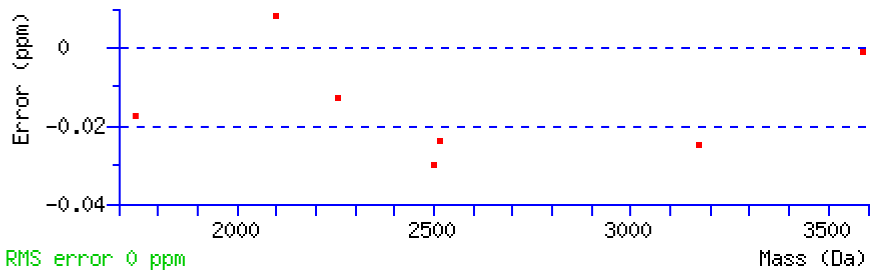

```
ID   ACCO2_ARATH              Reviewed;         320 AA.
AC   Q41931; O81093; Q7DLM8;
DT   03-MAY-2011, integrated into UniProtKB/Swiss-Prot.
DT   01-NOV-1998, sequence version 2.
DT   31-JUL-2019, entry version 131.
DE   RecName: Full=1-aminocyclopropane-1-carboxylate oxidase 2;
DE            Short=ACC oxidase 2;
DE            Short=AtACO2;
DE            EC=1.14.17.4;
GN   Name=ACO2; Synonyms=EI305; OrderedLocusNames=At1g62380;
GN   ORFNames=F24O1.10;
OS   Arabidopsis thaliana (Mouse-ear cress).
OC   Eukaryota; Viridiplantae; Streptophyta; Embryophyta; Tracheophyta;
OC   Spermatophyta; Magnoliopsida; eudicotyledons; Gunneridae;
OC   Pentapetalae; rosids; malvids; Brassicales; Brassicaceae; Camelineae;
OC   Arabidopsis.
OX   NCBI_TaxID=3702;
RN   [1]
RP   NUCLEOTIDE SEQUENCE [MRNA], TISSUE SPECIFICITY, AND INDUCTION BY
RP   ETHYLENE.
RC   STRAIN=cv. Columbia;
RX   PubMed=10409511;
RA   Raz V., Ecker J.R.;
RT   "Regulation of differential growth in the apical hook of
RT   Arabidopsis.";
RL   Development 126:3661-3668(1999).
RN   [2]
RP   NUCLEOTIDE SEQUENCE [LARGE SCALE GENOMIC DNA].
RC   STRAIN=cv. Columbia;
RX   PubMed=11130712; DOI=10.1038/35048500;
RA   Theologis A., Ecker J.R., Palm C.J., Federspiel N.A., Kaul S.,
RA   White O., Alonso J., Altafi H., Araujo R., Bowman C.L., Brooks S.Y.,
RA   Buehler E., Chan A., Chao Q., Chen H., Cheuk R.F., Chin C.W.,
RA   Chung M.K., Conn L., Conway A.B., Conway A.R., Creasy T.H., Dewar K.,
RA   Dunn P., Etgu P., Feldblyum T.V., Feng J.-D., Fong B., Fujii C.Y.,
RA   Gill J.E., Goldsmith A.D., Haas B., Hansen N.F., Hughes B., Huizar L.,
RA   Hunter J.L., Jenkins J., Johnson-Hopson C., Khan S., Khaykin E.,
RA   Kim C.J., Koo H.L., Kremenetskaia I., Kurtz D.B., Kwan A., Lam B.,
RA   Langin-Hooper S., Lee A., Lee J.M., Lenz C.A., Li J.H., Li Y.-P.,
RA   Lin X., Liu S.X., Liu Z.A., Luros J.S., Maiti R., Marziali A.,
RA   Militscher J., Miranda M., Nguyen M., Nierman W.C., Osborne B.I.,
RA   Pai G., Peterson J., Pham P.K., Rizzo M., Rooney T., Rowley D.,
RA   Sakano H., Salzberg S.L., Schwartz J.R., Shinn P., Southwick A.M.,
RA   Sun H., Tallon L.J., Tambunga G., Toriumi M.J., Town C.D.,
RA   Utterback T., Van Aken S., Vaysberg M., Vysotskaia V.S., Walker M.,
RA   Wu D., Yu G., Fraser C.M., Venter J.C., Davis R.W.;
RT   "Sequence and analysis of chromosome 1 of the plant Arabidopsis
RT   thaliana.";
RL   Nature 408:816-820(2000).
RN   [3]
RP   GENOME REANNOTATION.
RC   STRAIN=cv. Columbia;
```

RX   PubMed=27862469; DOI=10.1111/tpj.13415;
RA   Cheng C.Y., Krishnakumar V., Chan A.P., Thibaud-Nissen F., Schobel S.,
RA   Town C.D.;
RT   "Araport11: a complete reannotation of the Arabidopsis thaliana
RT   reference genome.";
RL   Plant J. 89:789-804(2017).
RN   [4]
RP   NUCLEOTIDE SEQUENCE [LARGE SCALE MRNA].
RC   STRAIN=cv. Columbia;
RX   PubMed=14593172; DOI=10.1126/science.1088305;
RA   Yamada K., Lim J., Dale J.M., Chen H., Shinn P., Palm C.J.,
RA   Southwick A.M., Wu H.C., Kim C.J., Nguyen M., Pham P.K., Cheuk R.F.,
RA   Karlin-Newmann G., Liu S.X., Lam B., Sakano H., Wu T., Yu G.,
RA   Miranda M., Quach H.L., Tripp M., Chang C.H., Lee J.M., Toriumi M.J.,
RA   Chan M.M., Tang C.C., Onodera C.S., Deng J.M., Akiyama K., Ansari Y.,
RA   Arakawa T., Banh J., Banno F., Bowser L., Brooks S.Y., Carninci P.,
RA   Chao Q., Choy N., Enju A., Goldsmith A.D., Gurjal M., Hansen N.F.,
RA   Hayashizaki Y., Johnson-Hopson C., Hsuan V.W., Iida K., Karnes M.,
RA   Khan S., Koesema E., Ishida J., Jiang P.X., Jones T., Kawai J.,
RA   Kamiya A., Meyers C., Nakajima M., Narusaka M., Seki M., Sakurai T.,
RA   Satou M., Tamse R., Vaysberg M., Wallender E.K., Wong C., Yamamura Y.,
RA   Yuan S., Shinozaki K., Davis R.W., Theologis A., Ecker J.R.;
RT   "Empirical analysis of transcriptional activity in the Arabidopsis
RT   genome.";
RL   Science 302:842-846(2003).
RN   [5]
RP   NUCLEOTIDE SEQUENCE [LARGE SCALE MRNA].
RC   STRAIN=cv. Columbia;
RA   Totoki Y., Seki M., Ishida J., Nakajima M., Enju A., Kamiya A.,
RA   Narusaka M., Shin-i T., Nakagawa M., Sakamoto N., Oishi K., Kohara Y.,
RA   Kobayashi M., Toyoda A., Sakaki Y., Sakurai T., Iida K., Akiyama K.,
RA   Satou M., Toyoda T., Konagaya A., Carninci P., Kawai J.,
RA   Hayashizaki Y., Shinozaki K.;
RT   "Large-scale analysis of RIKEN Arabidopsis full-length (RAFL) cDNAs.";
RL   Submitted (JUL-2006) to the EMBL/GenBank/DDBJ databases.
RN   [6]
RP   NUCLEOTIDE SEQUENCE [LARGE SCALE MRNA] OF 230-320.
RC   STRAIN=cv. Columbia; TISSUE=Green siliques;
RA   Raynal M., Grellet F., Laudie M., Meyer Y., Cooke R., Delseny M.;
RT   "The Arabidopsis thaliana transcribed genome: the GDR cDNA program.";
RL   Submitted (NOV-1992) to the EMBL/GenBank/DDBJ databases.
RN   [7]
RP   TISSUE SPECIFICITY.
RX   PubMed=12972669; DOI=10.1104/pp.103.022665;
RA   Vandenbussche F., Vriezen W.H., Smalle J., Laarhoven L.J.J.,
RA   Harren F.J.M., Van Der Straeten D.;
RT   "Ethylene and auxin control the Arabidopsis response to decreased
RT   light intensity.";
RL   Plant Physiol. 133:517-527(2003).
RN   [8]
RP   INDUCTION BY ETHYLENE.
RC   STRAIN=cv. Columbia;
RX   PubMed=15272873; DOI=10.1111/j.1365-313X.2004.02156.x;
RA   De Paepe A., Vuylsteke M., Van Hummelen P., Zabeau M.,
RA   Van Der Straeten D.;
RT   "Transcriptional profiling by cDNA-AFLP and microarray analysis
RT   reveals novel insights into the early response to ethylene in
RT   Arabidopsis.";
RL   Plant J. 39:537-559(2004).
RN   [9]
RP   INDUCTION BY OZONE.
RC   STRAIN=cv. Columbia;
RX   PubMed=15728341; DOI=10.1104/pp.104.055681;
RA   Overmyer K., Brosche M., Pellinen R., Kuittinen T., Tuominen H.,
RA   Ahlfors R., Keinaenen M., Saarma M., Scheel D., Kangasjaervi J.;
RT   "Ozone-induced programmed cell death in the Arabidopsis radical-
RT   induced cell death1 mutant.";
RL   Plant Physiol. 137:1092-1104(2005).
RN   [10]
RP   INDUCTION BY ETHYLENE.
RC   STRAIN=cv. Columbia;
RX   PubMed=16920797; DOI=10.1073/pnas.0605528103;
RA   Olmedo G., Guo H., Gregory B.D., Nourizadeh S.D., Aguilar-Henonin L.,
RA   Li H., An F., Guzman P., Ecker J.R.;

```
RT   "ETHYLENE-INSENSITIVE5 encodes a 5'-->3' exoribonuclease required for
RT   regulation of the EIN3-targeting F-box proteins EBF1/2.";
RL   Proc. Natl. Acad. Sci. U.S.A. 103:13286-13293(2006).
RN   [11]
RP   FUNCTION, INDUCTION BY VERY-LONG-CHAIN FATTY ACIDS, AND TISSUE
RP   SPECIFICITY.
RC   STRAIN=cv. Columbia;
RX   PubMed=17993622; DOI=10.1105/tpc.107.054437;
RA   Qin Y.-M., Hu C.-Y., Pang Y., Kastaniotis A.J., Hiltunen J.K.,
RA   Zhu Y.-X.;
RT   "Saturated very-long-chain fatty acids promote cotton fiber and
RT   Arabidopsis cell elongation by activating ethylene biosynthesis.";
RL   Plant Cell 19:3692-3704(2007).
RN   [12]
RP   FUNCTION, AND DISRUPTION PHENOTYPE.
RX   PubMed=20023197; DOI=10.1105/tpc.109.070201;
RA   Linkies A., Mueller K., Morris K., Tureckova V., Wenk M.,
RA   Cadman C.S.C., Corbineau F., Strnad M., Lynn J.R., Finch-Savage W.E.,
RA   Leubner-Metzger G.;
RT   "Ethylene interacts with abscisic acid to regulate endosperm rupture
RT   during germination: a comparative approach using Lepidium sativum and
RT   Arabidopsis thaliana.";
RL   Plant Cell 21:3803-3822(2009).
RN   [13]
RP   INDUCTION BY IRON DEFICIENCY.
RX   PubMed=20627899; DOI=10.1093/jxb/erq203;
RA   Garcia M.J., Lucena C., Romera F.J., Alcantara E., Perez-Vicente R.;
RT   "Ethylene and nitric oxide involvement in the up-regulation of key
RT   genes related to iron acquisition and homeostasis in Arabidopsis.";
RL   J. Exp. Bot. 61:3885-3899(2010).
RN   [14]
RP   COFACTOR.
RX   PubMed=20018591; DOI=10.1104/pp.109.147942;
RA   Tan Y.-F., O'Toole N., Taylor N.L., Millar A.H.;
RT   "Divalent metal ions in plant mitochondria and their role in
RT   interactions with proteins and oxidative stress-induced damage to
RT   respiratory function.";
RL   Plant Physiol. 152:747-761(2010).
RN   [15]
RP   INDUCTION BY NITRIC OXIDE.
RX   PubMed=21316254; DOI=10.1016/j.plaphy.2011.01.019;
RA   Garcia M.J., Suarez V., Romera F.J., Alcantara E., Perez-Vicente R.;
RT   "A new model involving ethylene, nitric oxide and Fe to explain the
RT   regulation of Fe-acquisition genes in Strategy I plants.";
RL   Plant Physiol. Biochem. 49:537-544(2011).
CC   -!- FUNCTION: Enzyme involved in the ethylene biosynthesis. Required
CC       to mediate the 1-aminocyclopropane-1-carboxylic acid (ACC)-
CC       mediated reversion of the ABA-induced inhibition of seed
CC       germination via endosperm rupture. May promote stem elongation by
CC       maximizing the extensibility cells, possibly by activating
CC       ethylene biosynthesis, in response to very-long-chain fatty acids
CC       (VLCFAs C20:0 to C30:0). {ECO:0000269|PubMed:17993622,
CC       ECO:0000269|PubMed:20023197}.
CC   -!- CATALYTIC ACTIVITY:
CC       Reaction=1-aminocyclopropane-1-carboxylate + L-ascorbate + O2 =
CC         CO2 + ethene + 2 H2O + hydrogen cyanide + L-dehydroascorbate;
CC         Xref=Rhea:RHEA:23640, ChEBI:CHEBI:15377, ChEBI:CHEBI:15379,
CC         ChEBI:CHEBI:16526, ChEBI:CHEBI:18153, ChEBI:CHEBI:18407,
CC         ChEBI:CHEBI:38290, ChEBI:CHEBI:58360, ChEBI:CHEBI:58539;
CC         EC=1.14.17.4;
CC   -!- COFACTOR:
CC       Name=Fe(2+); Xref=ChEBI:CHEBI:29033;
CC         Evidence={ECO:0000255|PROSITE-ProRule:PRU00805,
CC         ECO:0000269|PubMed:20018591};
CC       Name=Cu(2+); Xref=ChEBI:CHEBI:29036;
CC         Evidence={ECO:0000255|PROSITE-ProRule:PRU00805,
CC         ECO:0000269|PubMed:20018591};
CC       Note=Binds 1 Fe(2+) ion per subunit. Can also bind Cu(2+) ions.
CC       {ECO:0000255|PROSITE-ProRule:PRU00805,
CC       ECO:0000269|PubMed:20018591};
CC   -!- PATHWAY: Alkene biosynthesis; ethylene biosynthesis via S-
CC       adenosyl-L-methionine; ethylene from S-adenosyl-L-methionine: step
CC       2/2.
CC   -!- TISSUE SPECIFICITY: Expressed in vegetative tissues.
```

```
CC       Constitutively expressed in leaves and blades. In ethylene exposed
CC       etiolated seedlings, localized in cells at the outer side of the
CC       exaggerated hook in an ethylene-dependent manner and following an
CC       ethylene sensitive pattern. Also detected in the root tip when
CC       treated by ethylene. {ECO:0000269|PubMed:10409511,
CC       ECO:0000269|PubMed:12972669, ECO:0000269|PubMed:17993622}.
CC   -!- INDUCTION: Upon iron deprivation. Induced by ethylene,
CC       particularly in root tips and hooks of ethiolated seedlings.
CC       Promoted by ozone O(3). Accumulates in response to very-long-chain
CC       fatty acids (VLCFAs C20:0 to C30:0). Induced in roots by nitric
CC       oxide (NO). {ECO:0000269|PubMed:10409511,
CC       ECO:0000269|PubMed:15272873, ECO:0000269|PubMed:15728341,
CC       ECO:0000269|PubMed:16920797, ECO:0000269|PubMed:17993622,
CC       ECO:0000269|PubMed:20627899, ECO:0000269|PubMed:21316254}.
CC   -!- DISRUPTION PHENOTYPE: Impaired in the 1-aminocyclopropane-1-
CC       carboxylic acid (ACC)-mediated reversion of the ABA-induced
CC       inhibition of seed germination. {ECO:0000269|PubMed:20023197}.
CC   -!- SIMILARITY: Belongs to the iron/ascorbate-dependent oxidoreductase
CC       family. {ECO:0000305}.
DR   EMBL; AF016100; AAC27484.1; -; mRNA.
DR   EMBL; AC003113; AAF70838.1; -; Genomic_DNA.
DR   EMBL; CP002684; AEE33960.1; -; Genomic_DNA.
DR   EMBL; AY045876; AAK76550.1; -; mRNA.
DR   EMBL; AY062685; AAL32763.1; -; mRNA.
DR   EMBL; AY093381; AAM13380.1; -; mRNA.
DR   EMBL; AY133851; AAM91785.1; -; mRNA.
DR   EMBL; AK230351; BAF02150.1; -; mRNA.
DR   EMBL; Z17775; CAA79062.1; -; mRNA.
DR   PIR; T01448; T01448.
DR   PIR; T52267; T52267.
DR   RefSeq; NP_176428.1; NM_104918.5.
DR   PDB; 5GJ9; X-ray; 2.10 A; A/B=1-303.
DR   PDB; 5GJA; X-ray; 2.10 A; A/B/C/D/E/F/G/H=1-303.
DR   PDBsum; 5GJ9; -.
DR   PDBsum; 5GJA; -.
DR   SMR; Q41931; -.
DR   BioGrid; 27757; 2.
DR   IntAct; Q41931; 1.
DR   STRING; 3702.AT1G62380.1; -.
DR   iPTMnet; Q41931; -.
DR   SwissPalm; Q41931; -.
DR   PaxDb; Q41931; -.
DR   PRIDE; Q41931; -.
DR   EnsemblPlants; AT1G62380.1; AT1G62380.1; AT1G62380.
DR   GeneID; 842536; -.
DR   Gramene; AT1G62380.1; AT1G62380.1; AT1G62380.
DR   KEGG; ath:AT1G62380; -.
DR   Araport; AT1G62380; -.
DR   TAIR; locus:2027099; AT1G62380.
DR   eggNOG; KOG0143; Eukaryota.
DR   eggNOG; COG3491; LUCA.
DR   HOGENOM; HOG000276735; -.
DR   KO; K05933; -.
DR   OMA; DEYRNVM; -.
DR   OrthoDB; 755371at2759; -.
DR   PhylomeDB; Q41931; -.
DR   UniPathway; UPA00384; UER00563.
DR   PRO; PR:Q41931; -.
DR   Proteomes; UP000006548; Chromosome 1.
DR   ExpressionAtlas; Q41931; baseline and differential.
DR   Genevisible; Q41931; AT.
DR   GO; GO:0005618; C:cell wall; IDA:TAIR.
DR   GO; GO:0005829; C:cytosol; IDA:TAIR.
DR   GO; GO:0005783; C:endoplasmic reticulum; IDA:TAIR.
DR   GO; GO:0005794; C:Golgi apparatus; IDA:TAIR.
DR   GO; GO:0005886; C:plasma membrane; IDA:TAIR.
DR   GO; GO:0009506; C:plasmodesma; IDA:TAIR.
DR   GO; GO:0009815; F:1-aminocyclopropane-1-carboxylate oxidase activity; ISS:TAIR.
DR   GO; GO:0005507; F:copper ion binding; IDA:TAIR.
DR   GO; GO:0051213; F:dioxygenase activity; IBA:GO_Central.
DR   GO; GO:0031418; F:L-ascorbic acid binding; IEA:UniProtKB-KW.
DR   GO; GO:0071398; P:cellular response to fatty acid; IEP:UniProtKB.
DR   GO; GO:0071281; P:cellular response to iron ion; IEP:TAIR.
DR   GO; GO:0071732; P:cellular response to nitric oxide; IEP:UniProtKB.
```

```
DR   GO; GO:0006952; P:defense response; IEA:UniProtKB-KW.
DR   GO; GO:0009727; P:detection of ethylene stimulus; IDA:TAIR.
DR   GO; GO:0009693; P:ethylene biosynthetic process; TAS:TAIR.
DR   GO; GO:0010030; P:positive regulation of seed germination; IMP:UniProtKB.
DR   GO; GO:0009735; P:response to cytokinin; IDA:TAIR.
DR   GO; GO:0009651; P:response to salt stress; IEP:TAIR.
DR   Gene3D; 2.60.120.330; -; 1.
DR   InterPro; IPR026992; DIOX_N.
DR   InterPro; IPR027443; IPNS-like.
DR   InterPro; IPR005123; Oxoglu/Fe-dep_dioxygenase.
DR   Pfam; PF03171; 2OG-FeII_Oxy; 1.
DR   Pfam; PF14226; DIOX_N; 1.
DR   PROSITE; PS51471; FE2OG_OXY; 1.
PE   1: Evidence at protein level;
KW   3D-structure; Coiled coil; Complete proteome; Copper;
KW   Ethylene biosynthesis; Iron; Metal-binding; Oxidoreductase;
KW   Plant defense; Reference proteome; Vitamin C.
FT   CHAIN         1    320       1-aminocyclopropane-1-carboxylate oxidase
FT                                2.
FT                                /FTId=PRO_0000408298.
FT   DOMAIN      156    256       Fe2OG dioxygenase. {ECO:0000255|PROSITE-
FT                                ProRule:PRU00805}.
FT   COILED      111    143       {ECO:0000255}.
FT   METAL       180    180       Iron. {ECO:0000255|PROSITE-
FT                                ProRule:PRU00805}.
FT   METAL       182    182       Iron. {ECO:0000255|PROSITE-
FT                                ProRule:PRU00805}.
FT   METAL       237    237       Iron. {ECO:0000255|PROSITE-
FT                                ProRule:PRU00805}.
FT   BINDING     247    247       2-oxoglutarate. {ECO:0000255|PROSITE-
FT                                ProRule:PRU00805}.
FT   CONFLICT    261    261       E -> D (in Ref. 1; AAC27484).
FT                                {ECO:0000305}.
FT   STRAND        9     11       {ECO:0000244|PDB:5GJ9}.
FT   HELIX        12     15       {ECO:0000244|PDB:5GJ9}.
FT   HELIX        20     33       {ECO:0000244|PDB:5GJ9}.
FT   STRAND       35     41       {ECO:0000244|PDB:5GJ9}.
FT   HELIX        46     62       {ECO:0000244|PDB:5GJ9}.
FT   HELIX        64     74       {ECO:0000244|PDB:5GJ9}.
FT   TURN         75     78       {ECO:0000244|PDB:5GJ9}.
FT   STRAND       84     86       {ECO:0000244|PDB:5GJA}.
FT   STRAND       91    100       {ECO:0000244|PDB:5GJ9}.
FT   HELIX       103    105       {ECO:0000244|PDB:5GJ9}.
FT   HELIX       111    139       {ECO:0000244|PDB:5GJ9}.
FT   HELIX       145    151       {ECO:0000244|PDB:5GJ9}.
FT   TURN        152    154       {ECO:0000244|PDB:5GJ9}.
FT   STRAND      157    165       {ECO:0000244|PDB:5GJ9}.
FT   HELIX       171    173       {ECO:0000244|PDB:5GJ9}.
FT   STRAND      176    180       {ECO:0000244|PDB:5GJ9}.
FT   STRAND      183    191       {ECO:0000244|PDB:5GJ9}.
FT   STRAND      198    202       {ECO:0000244|PDB:5GJ9}.
FT   STRAND      205    208       {ECO:0000244|PDB:5GJ9}.
FT   STRAND      216    220       {ECO:0000244|PDB:5GJ9}.
FT   HELIX       222    227       {ECO:0000244|PDB:5GJ9}.
FT   TURN        228    230       {ECO:0000244|PDB:5GJ9}.
FT   STRAND      237    239       {ECO:0000244|PDB:5GJ9}.
FT   STRAND      247    255       {ECO:0000244|PDB:5GJ9}.
FT   HELIX       266    268       {ECO:0000244|PDB:5GJ9}.
FT   HELIX       280    290       {ECO:0000244|PDB:5GJ9}.
FT   HELIX       296    301       {ECO:0000244|PDB:5GJ9}.
SQ   SEQUENCE   320 AA;  36183 MW;  E9B22DF21FDE35A4 CRC64;
     MEKNMKFPVV DLSKLNGEER DQTMALINEA CENWGFFEIV NHGLPHDLMD KIEKMTKDHY
     KTCQEQKFND MLKSKGLDNL ETEVEDVDWE STFYVRHLPQ SNLNDISDVS DEYRTAMKDF
     GKRLENLAED LLDLLCENLG LEKGYLKKVF HGTKGPTFGT KVSNYPPCPK PEMIKGLRAH
     TDAGGIILLF QDDKVSGLQL LKDGDWIDVP PLNHSIVINL GDQLEVITNG KYKSVLHRVV
     TQQEGNRMSV ASFYNPGSDA EISPATSLVE KDSEYPSFVF DDYMKLYAGV KFQPKEPRFA
     AMKNASAVTE LNPTAAVETF
```

MATRIX SCIENCE MASCOT Search Results

Protein View: APX1_ARATH

L-ascorbate peroxidase 1, cytosolic OS=Arabidopsis thaliana OX=3702 GN=APX1 PE=1 SV=2

**Database:**	SwissProt
**Score:**	123
**Expect:**	2e-08
**Monoisotopic mass (M_r):**	27544
**Calculated pI:**	5.72
**Taxonomy:**	Arabidopsis thaliana

Sequence similarity is available as an NCBI BLAST search of APX1_ARATH against nr.

Search parameters

**Enzyme:**	Trypsin: cuts C-term side of KR unless next residue is P.
**Mass values searched:**	17
**Mass values matched:**	11

Protein sequence coverage: 68%

Matched peptides shown in ***bold red***.

```
  1 MTKNYPTVSE DYKKAVEKCR RKLRGLIAEK NCAPIMVRLA WHSAGTFDCQ
 51 SRTGGPFGTM RFDAEQAHGA NSGIHIALRL LDPIREQFPT ISFADFHQLA
101 GVVAVEVTGG PDIPFHPGRE DKPQPPPEGR LPDATKGCDH LRDVFAKQMG
151 LSDKDIVALS GAHTLGRCHK DRSGFEGAWT SNPLIFDNSY FKELLSGEKE
201 GLLQLVSDKA LLDDPVFRPL VEKYAADEDA FFADYAEAHM KLSELGFADA
```

Unformatted sequence string: 250 residues (for pasting into other applications).

Sort by ◉ residue number ○ increasing mass ○ decreasing mass
Show ◉ matched peptides only ○ predicted peptides also

Start – End	Observed	Mr(expt)	Mr(calc)	Delta	M	Peptide
4 – 13	1215.5528	1214.5455	1214.5455	0.0000	0	K.NYPTVSEDYK.K
39 – 52	1578.7118	1577.7045	1577.7045	0.0000	0	R.LAWHSAGTFDCQSR.T
53 – 61	923.4403	922.4330	922.4331	-0.0001	0	R.TGGPFGTMR.F
62 – 79	1906.9518	1905.9445	1905.9445	-0.0000	0	R.FDAEQAHGANSGIHIALR.L
86 – 119	3635.8280	3634.8207	3634.8209	-0.0002	0	R.EQFPTISFADFHQLAGVVAVEVTGGPDIPFHPGR.E
120 – 130	1249.6171	1248.6098	1248.6098	-0.0000	0	R.EDKPQPPPEGR.L
155 – 167	1309.7222	1308.7149	1308.7150	-0.0001	0	K.DIVALSGAHTLGR.C
173 – 192	2280.0607	2279.0534	2279.0535	-0.0001	0	R.SGFEGAWTSNPLIFDNSYFK.E
200 – 209	1101.6150	1100.6077	1100.6077	-0.0000	0	K.EGLLQLVSDK.A
210 – 223	1611.9104	1610.9031	1610.9032	-0.0001	0	K.ALLDDPVFRPLVEK.Y
224 – 241	2064.8643	2063.8570	2063.8571	-0.0000	0	K.YAADEDAFFADYAEAHMK.L

No match to: 120.0000, 155.0000, 173.0000, 200.0000, 210.0000, 224.0000

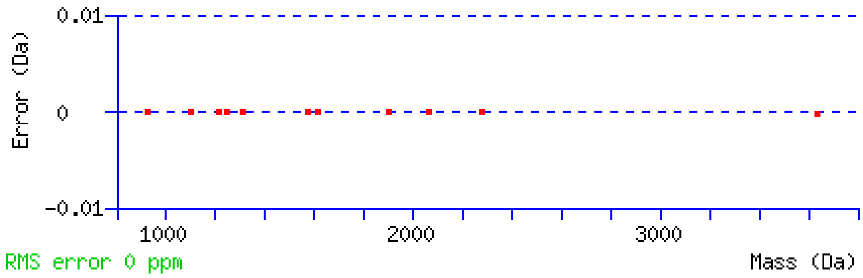

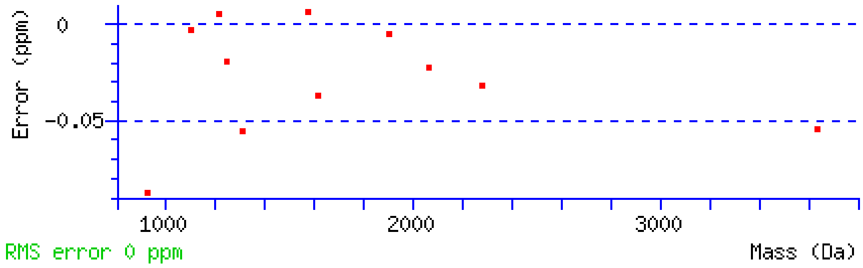

```
ID   APX1_ARATH              Reviewed;         250 AA.
AC   Q05431; Q0WLU2; Q2V4P8; Q2V4P9;
DT   01-FEB-1995, integrated into UniProtKB/Swiss-Prot.
DT   23-JAN-2007, sequence version 2.
DT   31-JUL-2019, entry version 163.
DE   RecName: Full=L-ascorbate peroxidase 1, cytosolic;
DE            Short=AP;
DE            Short=AtAPx01;
DE            EC=1.11.1.11;
GN   Name=APX1; OrderedLocusNames=At1g07890; ORFNames=F24B9.2;
OS   Arabidopsis thaliana (Mouse-ear cress).
OC   Eukaryota; Viridiplantae; Streptophyta; Embryophyta; Tracheophyta;
OC   Spermatophyta; Magnoliopsida; eudicotyledons; Gunneridae;
OC   Pentapetalae; rosids; malvids; Brassicales; Brassicaceae; Camelineae;
OC   Arabidopsis.
OX   NCBI_TaxID=3702;
RN   [1]
RP   NUCLEOTIDE SEQUENCE [MRNA], AND PROTEIN SEQUENCE OF 2-17.
RC   STRAIN=cv. Columbia; TISSUE=Leaf;
RX   PubMed=1558944; DOI=10.1007/BF00020011;
RA   Kubo A., Saji H., Tanaka K., Tanaka K., Kondo N.;
RT   "Cloning and sequencing of a cDNA encoding ascorbate peroxidase from
RT   Arabidopsis thaliana.";
RL   Plant Mol. Biol. 18:691-701(1992).
RN   [2]
RP   NUCLEOTIDE SEQUENCE [GENOMIC DNA].
RC   STRAIN=cv. Columbia;
RX   PubMed=8422923; DOI=10.1016/0014-5793(93)81185-3;
RA   Kubo A., Saji H., Tanaka K., Kondo N.;
RT   "Genomic DNA structure of a gene encoding cytosolic ascorbate
RT   peroxidase from Arabidopsis thaliana.";
RL   FEBS Lett. 315:313-317(1993).
RN   [3]
RP   NUCLEOTIDE SEQUENCE [GENOMIC DNA].
RC   STRAIN=cv. Columbia;
RA   Tremousaygue D., Bardet C., Dabos P., Regad F., Pelese F., Lescure B.;
RL   Submitted (JUL-1996) to the EMBL/GenBank/DDBJ databases.
RN   [4]
RP   NUCLEOTIDE SEQUENCE [LARGE SCALE GENOMIC DNA].
RC   STRAIN=cv. Columbia;
RX   PubMed=11130712; DOI=10.1038/35048500;
RA   Theologis A., Ecker J.R., Palm C.J., Federspiel N.A., Kaul S.,
RA   White O., Alonso J., Altafi H., Araujo R., Bowman C.L., Brooks S.Y.,
RA   Buehler E., Chan A., Chao Q., Chen H., Cheuk R.F., Chin C.W.,
RA   Chung M.K., Conn L., Conway A.B., Conway A.R., Creasy T.H., Dewar K.,
RA   Dunn P., Etgu P., Feldblyum T.V., Feng J.-D., Fong B., Fujii C.Y.,
RA   Gill J.E., Goldsmith A.D., Haas B., Hansen N.F., Hughes B., Huizar L.,
RA   Hunter J.L., Jenkins J., Johnson-Hopson C., Khan S., Khaykin E.,
RA   Kim C.J., Koo H.L., Kremenetskaia I., Kurtz D.B., Kwan A., Lam B.,
RA   Langin-Hooper S., Lee A., Lee J.M., Lenz C.A., Li J.H., Li Y.-P.,
RA   Lin X., Liu S.X., Liu Z.A., Luros J.S., Maiti R., Marziali A.,
RA   Militscher J., Miranda M., Nguyen M., Nierman W.C., Osborne B.I.,
RA   Pai G., Peterson J., Pham P.K., Rizzo M., Rooney T., Rowley D.,
```

```
RA   Sakano H., Salzberg S.L., Schwartz J.R., Shinn P., Southwick A.M.,
RA   Sun H., Tallon L.J., Tambunga G., Toriumi M.J., Town C.D.,
RA   Utterback T., Van Aken S., Vaysberg M., Vysotskaia V.S., Walker M.,
RA   Wu D., Yu G., Fraser C.M., Venter J.C., Davis R.W.;
RT   "Sequence and analysis of chromosome 1 of the plant Arabidopsis
RT   thaliana.";
RL   Nature 408:816-820(2000).
RN   [5]
RP   GENOME REANNOTATION.
RC   STRAIN=cv. Columbia;
RX   PubMed=27862469; DOI=10.1111/tpj.13415;
RA   Cheng C.Y., Krishnakumar V., Chan A.P., Thibaud-Nissen F., Schobel S.,
RA   Town C.D.;
RT   "Araport11: a complete reannotation of the Arabidopsis thaliana
RT   reference genome.";
RL   Plant J. 89:789-804(2017).
RN   [6]
RP   NUCLEOTIDE SEQUENCE [LARGE SCALE MRNA].
RC   STRAIN=cv. Columbia;
RX   PubMed=14593172; DOI=10.1126/science.1088305;
RA   Yamada K., Lim J., Dale J.M., Chen H., Shinn P., Palm C.J.,
RA   Southwick A.M., Wu H.C., Kim C.J., Nguyen M., Pham P.K., Cheuk R.F.,
RA   Karlin-Newmann G., Liu S.X., Lam B., Sakano H., Wu T., Yu G.,
RA   Miranda M., Quach H.L., Tripp M., Chang C.H., Lee J.M., Toriumi M.J.,
RA   Chan M.M., Tang C.C., Onodera C.S., Deng J.M., Akiyama K., Ansari Y.,
RA   Arakawa T., Banh J., Banno F., Bowser L., Brooks S.Y., Carninci P.,
RA   Chao Q., Choy N., Enju A., Goldsmith A.D., Gurjal M., Hansen N.F.,
RA   Hayashizaki Y., Johnson-Hopson C., Hsuan V.W., Iida K., Karnes M.,
RA   Khan S., Koesema E., Ishida J., Jiang P.X., Jones T., Kawai J.,
RA   Kamiya A., Meyers C., Nakajima M., Narusaka M., Seki M., Sakurai T.,
RA   Satou M., Tamse R., Vaysberg M., Wallender E.K., Wong C., Yamamura Y.,
RA   Yuan S., Shinozaki K., Davis R.W., Theologis A., Ecker J.R.;
RT   "Empirical analysis of transcriptional activity in the Arabidopsis
RT   genome.";
RL   Science 302:842-846(2003).
RN   [7]
RP   NUCLEOTIDE SEQUENCE [LARGE SCALE MRNA].
RC   STRAIN=cv. Columbia;
RA   Totoki Y., Seki M., Ishida J., Nakajima M., Enju A., Kamiya A.,
RA   Narusaka M., Shin-i T., Nakagawa M., Sakamoto N., Oishi K., Kohara Y.,
RA   Kobayashi M., Toyoda A., Sakaki Y., Sakurai T., Iida K., Akiyama K.,
RA   Satou M., Toyoda T., Konagaya A., Carninci P., Kawai J.,
RA   Hayashizaki Y., Shinozaki K.;
RT   "Large-scale analysis of RIKEN Arabidopsis full-length (RAFL) cDNAs.";
RL   Submitted (JUL-2006) to the EMBL/GenBank/DDBJ databases.
RN   [8]
RP   NUCLEOTIDE SEQUENCE [LARGE SCALE MRNA].
RA   Brover V.V., Troukhan M.E., Alexandrov N.A., Lu Y.-P., Flavell R.B.,
RA   Feldmann K.A.;
RT   "Full-length cDNA from Arabidopsis thaliana.";
RL   Submitted (MAR-2002) to the EMBL/GenBank/DDBJ databases.
RN   [9]
RP   INDUCTION.
RX   PubMed=8534847; DOI=10.1007/BF00020979;
RA   Kubo A., Saji H., Tanaka K., Kondo N.;
RT   "Expression of Arabidopsis cytosolic ascorbate peroxidase gene in
RT   response to ozone or sulfur dioxide.";
RL   Plant Mol. Biol. 29:479-489(1995).
RN   [10]
RP   INDUCTION.
RX   PubMed=9144965; DOI=10.1105/tpc.9.4.627;
RA   Karpinski S., Escobar C., Karpinski B., Creissen G.P.,
RA   Mullineaux P.M.;
RT   "Photosynthetic electron transport regulates the expression of
RT   cytosolic ascorbate peroxidase genes in Arabidopsis during excess
RT   light stress.";
RL   Plant Cell 9:627-640(1997).
RN   [11]
RP   TISSUE SPECIFICITY, AND INDUCTION.
RX   PubMed=9808745; DOI=10.1104/pp.118.3.1005;
RA   Storozhenko S., De Pauw P., Van Montagu M., Inze D., Kushnir S.;
RT   "The heat-shock element is a functional component of the Arabidopsis
RT   APX1 gene promoter.";
RL   Plant Physiol. 118:1005-1014(1998).
RN   [12]
```

```
RP   INDUCTION.
RX   PubMed=14739345; DOI=10.1104/pp.103.029876;
RA   Fourcroy P., Vansuyt G., Kushnir S., Inze D., Briat J.-F.;
RT   "Iron-regulated expression of a cytosolic ascorbate peroxidase encoded
RT   by the APX1 gene in Arabidopsis seedlings.";
RL   Plant Physiol. 134:605-613(2004).
RN   [13]
RP   FUNCTION.
RX   PubMed=15608336; DOI=10.1105/tpc.104.026971;
RA   Davletova S., Rizhsky L., Liang H., Shengqiang Z., Oliver D.J.,
RA   Coutu J., Shulaev V., Schlauch K., Mittler R.;
RT   "Cytosolic ascorbate peroxidase 1 is a central component of the
RT   reactive oxygen gene network of Arabidopsis.";
RL   Plant Cell 17:268-281(2005).
RN   [14]
RP   INDUCTION BY CADMIUM.
RC   STRAIN=cv. Columbia;
RX   PubMed=16502469; DOI=10.1002/pmic.200500543;
RA   Sarry J.-E., Kuhn L., Ducruix C., Lafaye A., Junot C., Hugouvieux V.,
RA   Jourdain A., Bastien O., Fievet J.B., Vailhen D., Amekraz B.,
RA   Moulin C., Ezan E., Garin J., Bourguignon J.;
RT   "The early responses of Arabidopsis thaliana cells to cadmium exposure
RT   explored by protein and metabolite profiling analyses.";
RL   Proteomics 6:2180-2198(2006).
RN   [15]
RP   IDENTIFICATION BY MASS SPECTROMETRY [LARGE SCALE ANALYSIS].
RC   STRAIN=cv. Columbia;
RX   PubMed=19245862; DOI=10.1016/j.jprot.2009.02.004;
RA   Jones A.M.E., MacLean D., Studholme D.J., Serna-Sanz A.,
RA   Andreasson E., Rathjen J.P., Peck S.C.;
RT   "Phosphoproteomic analysis of nuclei-enriched fractions from
RT   Arabidopsis thaliana.";
RL   J. Proteomics 72:439-451(2009).
RN   [16]
RP   PHOSPHORYLATION [LARGE SCALE ANALYSIS] AT SER-196, AND IDENTIFICATION
RP   BY MASS SPECTROMETRY [LARGE SCALE ANALYSIS].
RX   PubMed=22092075; DOI=10.1021/pr200917t;
RA   Aryal U.K., Krochko J.E., Ross A.R.;
RT   "Identification of phosphoproteins in Arabidopsis thaliana leaves
RT   using polyethylene glycol fractionation, immobilized metal-ion
RT   affinity chromatography, two-dimensional gel electrophoresis and mass
RT   spectrometry.";
RL   J. Proteome Res. 11:425-437(2012).
CC   -!- FUNCTION: Plays a key role in hydrogen peroxide removal.
CC       Constitutes a central component of the reactive oxygen gene
CC       network. {ECO:0000269|PubMed:15608336}.
CC   -!- CATALYTIC ACTIVITY:
CC       Reaction=H2O2 + L-ascorbate = 2 H2O + L-dehydroascorbate;
CC         Xref=Rhea:RHEA:22996, ChEBI:CHEBI:15377, ChEBI:CHEBI:16240,
CC         ChEBI:CHEBI:38290, ChEBI:CHEBI:58539; EC=1.11.1.11;
CC   -!- COFACTOR:
CC       Name=heme b; Xref=ChEBI:CHEBI:60344;
CC       Note=Binds 1 heme b (iron(II)-protoporphyrin IX) group per
CC       subunit.;
CC   -!- INTERACTION:
CC       Q42403:TRX3; NbExp=2; IntAct=EBI-449365, EBI-449157;
CC   -!- SUBCELLULAR LOCATION: Cytoplasm.
CC   -!- ALTERNATIVE PRODUCTS:
CC       Event=Alternative splicing; Named isoforms=1;
CC         Comment=A number of isoforms are produced. According to EST
CC         sequences.;
CC       Name=1;
CC         IsoId=Q05431-1; Sequence=Displayed;
CC   -!- TISSUE SPECIFICITY: Predominantly expressed in flowers.
CC       {ECO:0000269|PubMed:9808745}.
CC   -!- INDUCTION: By ethylene, ozone, sulfur dioxide, Fe exposure,
CC       oxidative and heat-shock stresses, and by excess light treatment.
CC       Induced by cadmium (PubMed:16502469).
CC       {ECO:0000269|PubMed:14739345, ECO:0000269|PubMed:16502469,
CC       ECO:0000269|PubMed:8534847, ECO:0000269|PubMed:9144965,
CC       ECO:0000269|PubMed:9808745}.
CC   -!- MISCELLANEOUS: Binds one cation per subunit; probably K(+), but
CC       might also be Ca(2+). {ECO:0000250}.
CC   -!- SIMILARITY: Belongs to the peroxidase family. Ascorbate peroxidase
CC       subfamily. {ECO:0000305}.
```

```
DR   EMBL; X59600; CAA42168.1; -; mRNA.
DR   EMBL; D14442; BAA03334.1; -; Genomic_DNA.
DR   EMBL; U63815; AAB07880.1; -; Genomic_DNA.
DR   EMBL; AC007583; AAF75066.1; -; Genomic_DNA.
DR   EMBL; CP002684; AEE28200.1; -; Genomic_DNA.
DR   EMBL; CP002684; AEE28201.1; -; Genomic_DNA.
DR   EMBL; CP002684; AEE28202.1; -; Genomic_DNA.
DR   EMBL; CP002684; AEE28203.1; -; Genomic_DNA.
DR   EMBL; CP002684; AEE28204.1; -; Genomic_DNA.
DR   EMBL; CP002684; AEE28206.1; -; Genomic_DNA.
DR   EMBL; CP002684; AEE28207.1; -; Genomic_DNA.
DR   EMBL; AY039879; AAK63983.1; -; mRNA.
DR   EMBL; AY056395; AAL08251.1; -; mRNA.
DR   EMBL; AY094002; AAM16263.1; -; mRNA.
DR   EMBL; AK230096; BAF01915.1; -; mRNA.
DR   EMBL; AY086425; AAM63427.1; -; mRNA.
DR   PIR; D86214; D86214.
DR   PIR; S20866; S20866.
DR   RefSeq; NP_001030991.2; NM_001035914.2. [Q05431-1]
DR   RefSeq; NP_001077482.1; NM_001084013.1. [Q05431-1]
DR   RefSeq; NP_001117244.1; NM_001123772.2. [Q05431-1]
DR   RefSeq; NP_001318949.1; NM_001331739.1. [Q05431-1]
DR   RefSeq; NP_172267.1; NM_100663.4. [Q05431-1]
DR   RefSeq; NP_849607.1; NM_179276.2. [Q05431-1]
DR   RefSeq; NP_973786.1; NM_202057.2. [Q05431-1]
DR   SMR; Q05431; -.
DR   BioGrid; 22545; 4.
DR   IntAct; Q05431; 4.
DR   MINT; Q05431; -.
DR   STRING; 3702.AT1G07890.8; -.
DR   PeroxiBase; 1890; AtAPx01.
DR   iPTMnet; Q05431; -.
DR   SWISS-2DPAGE; Q05431; -.
DR   PaxDb; Q05431; -.
DR   PRIDE; Q05431; -.
DR   EnsemblPlants; AT1G07890.1; AT1G07890.1; AT1G07890. [Q05431-1]
DR   EnsemblPlants; AT1G07890.2; AT1G07890.2; AT1G07890. [Q05431-1]
DR   EnsemblPlants; AT1G07890.3; AT1G07890.3; AT1G07890. [Q05431-1]
DR   EnsemblPlants; AT1G07890.4; AT1G07890.4; AT1G07890. [Q05431-1]
DR   EnsemblPlants; AT1G07890.5; AT1G07890.5; AT1G07890. [Q05431-1]
DR   EnsemblPlants; AT1G07890.7; AT1G07890.7; AT1G07890. [Q05431-1]
DR   EnsemblPlants; AT1G07890.8; AT1G07890.8; AT1G07890. [Q05431-1]
DR   GeneID; 837304; -.
DR   Gramene; AT1G07890.1; AT1G07890.1; AT1G07890. [Q05431-1]
DR   Gramene; AT1G07890.2; AT1G07890.2; AT1G07890. [Q05431-1]
DR   Gramene; AT1G07890.3; AT1G07890.3; AT1G07890. [Q05431-1]
DR   Gramene; AT1G07890.4; AT1G07890.4; AT1G07890. [Q05431-1]
DR   Gramene; AT1G07890.5; AT1G07890.5; AT1G07890. [Q05431-1]
DR   Gramene; AT1G07890.7; AT1G07890.7; AT1G07890. [Q05431-1]
DR   Gramene; AT1G07890.8; AT1G07890.8; AT1G07890. [Q05431-1]
DR   KEGG; ath:AT1G07890; -.
DR   Araport; AT1G07890; -.
DR   TAIR; locus:2026616; AT1G07890.
DR   eggNOG; ENOG410IF5T; Eukaryota.
DR   eggNOG; COG0376; LUCA.
DR   HOGENOM; HOG000189824; -.
DR   InParanoid; Q05431; -.
DR   KO; K00434; -.
DR   OrthoDB; 1228462at2759; -.
DR   PhylomeDB; Q05431; -.
DR   BioCyc; ARA:GQT-2090-MONOMER; -.
DR   BRENDA; 1.11.1.11; 399.
DR   PRO; PR:Q05431; -.
DR   Proteomes; UP000006548; Chromosome 1.
DR   ExpressionAtlas; Q05431; baseline and differential.
DR   Genevisible; Q05431; AT.
DR   GO; GO:0005618; C:cell wall; IDA:TAIR.
DR   GO; GO:0009507; C:chloroplast; IDA:TAIR.
DR   GO; GO:0009570; C:chloroplast stroma; IDA:TAIR.
DR   GO; GO:0005829; C:cytosol; IDA:TAIR.
DR   GO; GO:0005794; C:Golgi apparatus; IDA:TAIR.
DR   GO; GO:0005886; C:plasma membrane; IDA:TAIR.
DR   GO; GO:0009506; C:plasmodesma; IDA:TAIR.
DR   GO; GO:0020037; F:heme binding; IEA:InterPro.
DR   GO; GO:0016688; F:L-ascorbate peroxidase activity; IMP:TAIR.
```

```
DR   GO; GO:0046872; F:metal ion binding; IEA:UniProtKB-KW.
DR   GO; GO:0004601; F:peroxidase activity; IBA:GO_Central.
DR   GO; GO:0034599; P:cellular response to oxidative stress; IBA:GO_Central.
DR   GO; GO:0009793; P:embryo development ending in seed dormancy; IMP:TAIR.
DR   GO; GO:0042744; P:hydrogen peroxide catabolic process; IMP:TAIR.
DR   GO; GO:0046686; P:response to cadmium ion; IEP:TAIR.
DR   GO; GO:0009735; P:response to cytokinin; IDA:TAIR.
DR   GO; GO:0009408; P:response to heat; IEP:TAIR.
DR   GO; GO:0000302; P:response to reactive oxygen species; IMP:TAIR.
DR   GO; GO:0009651; P:response to salt stress; IEP:TAIR.
DR   InterPro; IPR002016; Haem_peroxidase.
DR   InterPro; IPR010255; Haem_peroxidase_sf.
DR   InterPro; IPR002207; Peroxidase_I.
DR   InterPro; IPR019794; Peroxidases_AS.
DR   InterPro; IPR019793; Peroxidases_heam-ligand_BS.
DR   Pfam; PF00141; peroxidase; 1.
DR   PRINTS; PR00459; ASPEROXIDASE.
DR   PRINTS; PR00458; PEROXIDASE.
DR   SUPFAM; SSF48113; SSF48113; 1.
DR   PROSITE; PS00435; PEROXIDASE_1; 1.
DR   PROSITE; PS00436; PEROXIDASE_2; 1.
DR   PROSITE; PS50873; PEROXIDASE_4; 1.
PE   1: Evidence at protein level;
KW   Alternative splicing; Calcium; Complete proteome; Cytoplasm;
KW   Direct protein sequencing; Heme; Hydrogen peroxide; Iron;
KW   Metal-binding; Oxidoreductase; Peroxidase; Phosphoprotein; Potassium;
KW   Reference proteome.
FT   INIT_MET      1      1       Removed. {ECO:0000269|PubMed:1558944}.
FT   CHAIN         2    250       L-ascorbate peroxidase 1, cytosolic.
FT                                /FTId=PRO_0000055592.
FT   ACT_SITE     42     42       Proton acceptor. {ECO:0000255|PROSITE-
FT                                ProRule:PRU00297, ECO:0000255|PROSITE-
FT                                ProRule:PRU10012}.
FT   METAL       163    163       Iron (heme axial ligand).
FT                                {ECO:0000255|PROSITE-ProRule:PRU00297}.
FT   METAL       164    164       Potassium or calcium. {ECO:0000250}.
FT   METAL       180    180       Potassium or calcium. {ECO:0000250}.
FT   METAL       182    182       Potassium or calcium. {ECO:0000250}.
FT   METAL       185    185       Potassium or calcium; via carbonyl
FT                                oxygen. {ECO:0000250}.
FT   METAL       187    187       Potassium or calcium. {ECO:0000250}.
FT   SITE         38     38       Transition state stabilizer.
FT                                {ECO:0000255|PROSITE-ProRule:PRU00297}.
FT   MOD_RES     196    196       Phosphoserine.
FT                                {ECO:0000244|PubMed:22092075}.
SQ   SEQUENCE   250 AA;  27561 MW;  33A536D85B2CAA6C CRC64;
     MTKNYPTVSE DYKKAVEKCR RKLRGLIAEK NCAPIMVRLA WHSAGTFDCQ SRTGGPFGTM
     RFDAEQAHGA NSGIHIALRL LDPIREQFPT ISFADFHQLA GVVAVEVTGG PDIPFHPGRE
     DKPQPPPEGR LPDATKGCDH LRDVFAKQMG LSDKDIVALS GAHTLGRCHK DRSGFEGAWT
     SNPLIFDNSY FKELLSGEKE GLLQLVSDKA LLDDPVFRPL VEKYAADEDA FFADYAEAHM
     KLSELGFADA
```

MATRIX SCIENCE MASCOT Search Results

Protein View: SODM1_ARATH

Superoxide dismutase [Mn] 1, mitochondrial OS=Arabidopsis thaliana OX=3702 GN=MSD1 PE=1 SV=2

**Database:**	SwissProt
**Score:**	89
**Expect:**	5.4e-05
**Monoisotopic mass (M_r):**	25428
**Calculated pI:**	8.47
**Taxonomy:**	Arabidopsis thaliana

Sequence similarity is available as an NCBI BLAST search of SODM1_ARATH against nr.

Search parameters

**Enzyme:**	Trypsin: cuts C-term side of KR unless next residue is P.
**Mass values searched:**	15
**Mass values matched:**	8

Protein sequence coverage: 58%

Matched peptides shown in ***bold red***.

```
  1 MAIRCVASRK TLAGLKETSS RLLRIRGIQT FTLPDLPYDY GALEPAISGE
 51 IMQIHHQKHH QAYVTNYNNA LEQLDQAVNK GDASTVVKLQ SAIKFNGGGH
101 VNHSIFWKNL APSSEGGGEP PKGSLGSAID AHFGSLEGLV KKMSAEGAAV
151 QGSGWVWLGL DKELKKLVVD TTANQDPLVT KGGSLVPLVG IDVWEHAYYL
201 QYKNVRPEYL KNVWKVINWK YASEVYEKEN N
```

Unformatted sequence string: 231 residues (for pasting into other applications).

Sort by ◉ residue number ○ increasing mass ○ decreasing mass
Show ◉ matched peptides only ○ predicted peptides also

Start – End	Observed	Mr(expt)	Mr(calc)	Delta	M	Peptide
59 – 80	2570.2382	2569.2309	2569.2309	-0.0000	0	K.HHQAYVTNYNNALEQLDQAVNK.G
95 – 108	1599.7815	1598.7742	1598.7743	-0.0000	0	K.FNGGGHVNHSIFWK.N
109 – 122	1339.6488	1338.6415	1338.6415	-0.0000	0	K.NLAPSSEGGGEPPK.G
123 – 141	1857.9704	1856.9631	1856.9632	-0.0001	0	K.GSLGSAIDAHFGSLEGLVK.K
143 – 162	2062.0062	2060.9989	2060.9990	-0.0000	0	K.MSAEGAAVQGSGWVWLGLDK.E
167 – 181	1613.8744	1612.8671	1612.8672	-0.0001	0	K.LVVDTTANQDPLVTK.G
182 – 203	2507.2969	2506.2896	2506.2896	-0.0000	0	K.GGSLVPLVGIDVWEHAYYLQYK.N
204 – 211	1018.5680	1017.5607	1017.5607	0.0000	0	K.NVRPEYLK.N

No match to: 109.0000, 123.0000, 143.0000, 167.0000, 182.0000, 204.0000, 3284.6296

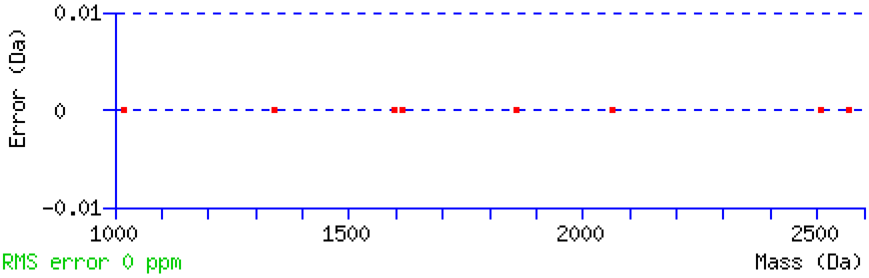

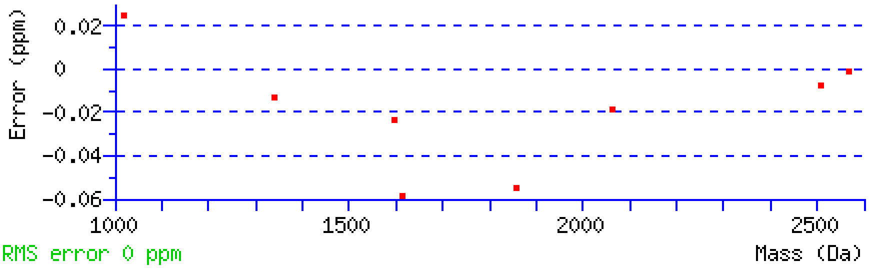

```
ID   SODM1_ARATH             Reviewed;         231 AA.
AC   O81235; Q8LEP0; Q9SRK3;
DT   11-JAN-2001, integrated into UniProtKB/Swiss-Prot.
DT   20-JUN-2002, sequence version 2.
DT   31-JUL-2019, entry version 152.
DE   RecName: Full=Superoxide dismutase [Mn] 1, mitochondrial;
DE            EC=1.15.1.1;
DE   AltName: Full=Protein MANGANESE SUPEROXIDE DISMUTASE 1;
DE            Short=AtMSD1;
DE   AltName: Full=Protein MATERNAL EFFECT EMBRYO ARREST 33;
DE   Flags: Precursor;
GN   Name=MSD1; Synonyms=MEE3, SODA; OrderedLocusNames=At3g10920;
GN   ORFNames=F9F8.26;
OS   Arabidopsis thaliana (Mouse-ear cress).
OC   Eukaryota; Viridiplantae; Streptophyta; Embryophyta; Tracheophyta;
OC   Spermatophyta; Magnoliopsida; eudicotyledons; Gunneridae;
OC   Pentapetalae; rosids; malvids; Brassicales; Brassicaceae; Camelineae;
OC   Arabidopsis.
OX   NCBI_TaxID=3702;
RN   [1]
RP   NUCLEOTIDE SEQUENCE [MRNA], AND GENE FAMILY.
RC   STRAIN=cv. Columbia;
RX   PubMed=9765550; DOI=10.1104/pp.118.2.637;
RA   Kliebenstein D.J., Monde R.A., Last R.L.;
RT   "Superoxide dismutase in Arabidopsis: an eclectic enzyme family with
RT   disparate regulation and protein localization.";
RL   Plant Physiol. 118:637-650(1998).
RN   [2]
RP   NUCLEOTIDE SEQUENCE [LARGE SCALE GENOMIC DNA].
RC   STRAIN=cv. Columbia;
RX   PubMed=11130713; DOI=10.1038/35048706;
RA   Salanoubat M., Lemcke K., Rieger M., Ansorge W., Unseld M.,
RA   Fartmann B., Valle G., Bloecker H., Perez-Alonso M., Obermaier B.,
RA   Delseny M., Boutry M., Grivell L.A., Mache R., Puigdomenech P.,
RA   De Simone V., Choisne N., Artiguenave F., Robert C., Brottier P.,
RA   Wincker P., Cattolico L., Weissenbach J., Saurin W., Quetier F.,
RA   Schaefer M., Mueller-Auer S., Gabel C., Fuchs M., Benes V.,
RA   Wurmbach E., Drzonek H., Erfle H., Jordan N., Bangert S.,
RA   Wiedelmann R., Kranz H., Voss H., Holland R., Brandt P., Nyakatura G.,
RA   Vezzi A., D'Angelo M., Pallavicini A., Toppo S., Simionati B.,
RA   Conrad A., Hornischer K., Kauer G., Loehnert T.-H., Nordsiek G.,
RA   Reichelt J., Scharfe M., Schoen O., Bargues M., Terol J., Climent J.,
RA   Navarro P., Collado C., Perez-Perez A., Ottenwaelder B., Duchemin D.,
RA   Cooke R., Laudie M., Berger-Llauro C., Purnelle B., Masuy D.,
RA   de Haan M., Maarse A.C., Alcaraz J.-P., Cottet A., Casacuberta E.,
RA   Monfort A., Argiriou A., Flores M., Liguori R., Vitale D.,
RA   Mannhaupt G., Haase D., Schoof H., Rudd S., Zaccaria P., Mewes H.-W.,
RA   Mayer K.F.X., Kaul S., Town C.D., Koo H.L., Tallon L.J., Jenkins J.,
RA   Rooney T., Rizzo M., Walts A., Utterback T., Fujii C.Y., Shea T.P.,
RA   Creasy T.H., Haas B., Maiti R., Wu D., Peterson J., Van Aken S.,
RA   Pai G., Militscher J., Sellers P., Gill J.E., Feldblyum T.V.,
RA   Preuss D., Lin X., Nierman W.C., Salzberg S.L., White O., Venter J.C.,
```

RA   Fraser C.M., Kaneko T., Nakamura Y., Sato S., Kato T., Asamizu E.,
RA   Sasamoto S., Kimura T., Idesawa K., Kawashima K., Kishida Y.,
RA   Kiyokawa C., Kohara M., Matsumoto M., Matsuno A., Muraki A.,
RA   Nakayama S., Nakazaki N., Shinpo S., Takeuchi C., Wada T.,
RA   Watanabe A., Yamada M., Yasuda M., Tabata S.;
RT   "Sequence and analysis of chromosome 3 of the plant Arabidopsis
RT   thaliana.";
RL   Nature 408:820-822(2000).
RN   [3]
RP   GENOME REANNOTATION.
RC   STRAIN=cv. Columbia;
RX   PubMed=27862469; DOI=10.1111/tpj.13415;
RA   Cheng C.Y., Krishnakumar V., Chan A.P., Thibaud-Nissen F., Schobel S.,
RA   Town C.D.;
RT   "Araport11: a complete reannotation of the Arabidopsis thaliana
RT   reference genome.";
RL   Plant J. 89:789-804(2017).
RN   [4]
RP   NUCLEOTIDE SEQUENCE [LARGE SCALE MRNA].
RC   STRAIN=cv. Columbia;
RX   PubMed=14593172; DOI=10.1126/science.1088305;
RA   Yamada K., Lim J., Dale J.M., Chen H., Shinn P., Palm C.J.,
RA   Southwick A.M., Wu H.C., Kim C.J., Nguyen M., Pham P.K., Cheuk R.F.,
RA   Karlin-Newmann G., Liu S.X., Lam B., Sakano H., Wu T., Yu G.,
RA   Miranda M., Quach H.L., Tripp M., Chang C.H., Lee J.M., Toriumi M.J.,
RA   Chan M.M., Tang C.C., Onodera C.S., Deng J.M., Akiyama K., Ansari Y.,
RA   Arakawa T., Banh J., Banno F., Bowser L., Brooks S.Y., Carninci P.,
RA   Chao Q., Choy N., Enju A., Goldsmith A.D., Gurjal M., Hansen N.F.,
RA   Hayashizaki Y., Johnson-Hopson C., Hsuan V.W., Iida K., Karnes M.,
RA   Khan S., Koesema E., Ishida J., Jiang P.X., Jones T., Kawai J.,
RA   Kamiya A., Meyers C., Nakajima M., Narusaka M., Seki M., Sakurai T.,
RA   Satou M., Tamse R., Vaysberg M., Wallender E.K., Wong C., Yamamura Y.,
RA   Yuan S., Shinozaki K., Davis R.W., Theologis A., Ecker J.R.;
RT   "Empirical analysis of transcriptional activity in the Arabidopsis
RT   genome.";
RL   Science 302:842-846(2003).
RN   [5]
RP   NUCLEOTIDE SEQUENCE [LARGE SCALE MRNA].
RA   Brover V.V., Troukhan M.E., Alexandrov N.A., Lu Y.-P., Flavell R.B.,
RA   Feldmann K.A.;
RT   "Full-length cDNA from Arabidopsis thaliana.";
RL   Submitted (MAR-2002) to the EMBL/GenBank/DDBJ databases.
RN   [6]
RP   IDENTIFICATION BY MASS SPECTROMETRY, AND SUBCELLULAR LOCATION [LARGE
RP   SCALE ANALYSIS].
RC   STRAIN=cv. Landsberg erecta;
RX   PubMed=14671022; DOI=10.1105/tpc.016055;
RA   Heazlewood J.L., Tonti-Filippini J.S., Gout A.M., Day D.A., Whelan J.,
RA   Millar A.H.;
RT   "Experimental analysis of the Arabidopsis mitochondrial proteome
RT   highlights signaling and regulatory components, provides assessment of
RT   targeting prediction programs, and indicates plant-specific
RT   mitochondrial proteins.";
RL   Plant Cell 16:241-256(2004).
RN   [7]
RP   ACTIVITY REGULATION.
RX   PubMed=17522887; DOI=10.1007/s00425-007-0547-6;
RA   Su Z., Chai M.F., Lu P.L., An R., Chen J., Wang X.C.;
RT   "AtMTM1, a novel mitochondrial protein, may be involved in activation
RT   of the manganese-containing superoxide dismutase in Arabidopsis.";
RL   Planta 226:1031-1039(2007).
RN   [8]
RP   INDUCTION BY SALT.
RX   PubMed=18275461; DOI=10.1111/j.1399-3054.2007.01009.x;
RA   Attia H., Arnaud N., Karray N., Lachaal M.;
RT   "Long-term effects of mild salt stress on growth, ion accumulation and
RT   superoxide dismutase expression of Arabidopsis rosette leaves.";
RL   Physiol. Plantarum 132:293-305(2008).
RN   [9]
RP   PHOSPHORYLATION [LARGE SCALE ANALYSIS] AT SER-124, AND IDENTIFICATION
RP   BY MASS SPECTROMETRY [LARGE SCALE ANALYSIS].
RX   PubMed=22092075; DOI=10.1021/pr200917t;
RA   Aryal U.K., Krochko J.E., Ross A.R.;
RT   "Identification of phosphoproteins in Arabidopsis thaliana leaves

```
RT   using polyethylene glycol fractionation, immobilized metal-ion
RT   affinity chromatography, two-dimensional gel electrophoresis and mass
RT   spectrometry.";
RL   J. Proteome Res. 11:425-437(2012).
CC   -!- FUNCTION: Destroys superoxide anion radicals which are normally
CC       produced within the cells and which are toxic to biological
CC       systems.
CC   -!- CATALYTIC ACTIVITY:
CC       Reaction=2 H(+) + 2 superoxide = H2O2 + O2; Xref=Rhea:RHEA:20696,
CC         ChEBI:CHEBI:15378, ChEBI:CHEBI:15379, ChEBI:CHEBI:16240,
CC         ChEBI:CHEBI:18421; EC=1.15.1.1;
CC   -!- COFACTOR:
CC       Name=Mn(2+); Xref=ChEBI:CHEBI:29035; Evidence={ECO:0000250};
CC       Note=Binds 1 Mn(2+) ion per subunit. {ECO:0000250};
CC   -!- ACTIVITY REGULATION: Activated by MTM1.
CC       {ECO:0000269|PubMed:17522887}.
CC   -!- SUBUNIT: Homotetramer. {ECO:0000250}.
CC   -!- SUBCELLULAR LOCATION: Mitochondrion matrix
CC       {ECO:0000269|PubMed:14671022}.
CC   -!- ALTERNATIVE PRODUCTS:
CC       Event=Alternative splicing; Named isoforms=1;
CC         Comment=A number of isoforms are produced. According to EST
CC         sequences.;
CC       Name=1;
CC         IsoId=O81235-1; Sequence=Displayed;
CC   -!- INDUCTION: Induced by salt stress. {ECO:0000269|PubMed:18275461}.
CC   -!- SIMILARITY: Belongs to the iron/manganese superoxide dismutase
CC       family. {ECO:0000305}.
DR   EMBL; AF061518; AAC24832.1; -; mRNA.
DR   EMBL; AC009991; AAF01529.1; -; Genomic_DNA.
DR   EMBL; CP002686; AEE74977.1; -; Genomic_DNA.
DR   EMBL; AY072495; AAL66910.1; -; mRNA.
DR   EMBL; AY059807; AAL24289.1; -; mRNA.
DR   EMBL; AY085319; AAM62550.1; -; mRNA.
DR   PIR; PA0012; PA0012.
DR   PIR; T50827; T50827.
DR   RefSeq; NP_187703.1; NM_111929.4. [O81235-1]
DR   PDB; 4C7U; X-ray; 1.95 A; A/B/C/D/E/F/G/H=30-231.
DR   PDBsum; 4C7U; -.
DR   SMR; O81235; -.
DR   STRING; 3702.AT3G10920.1; -.
DR   iPTMnet; O81235; -.
DR   PaxDb; O81235; -.
DR   PRIDE; O81235; -.
DR   EnsemblPlants; AT3G10920.1; AT3G10920.1; AT3G10920. [O81235-1]
DR   GeneID; 820263; -.
DR   Gramene; AT3G10920.1; AT3G10920.1; AT3G10920. [O81235-1]
DR   KEGG; ath:AT3G10920; -.
DR   Araport; AT3G10920; -.
DR   TAIR; locus:2085552; AT3G10920.
DR   eggNOG; KOG0876; Eukaryota.
DR   eggNOG; COG0605; LUCA.
DR   HOGENOM; HOG000013583; -.
DR   InParanoid; O81235; -.
DR   KO; K04564; -.
DR   OMA; KWGSFDK; -.
DR   OrthoDB; 1353361at2759; -.
DR   PhylomeDB; O81235; -.
DR   PRO; PR:O81235; -.
DR   Proteomes; UP000006548; Chromosome 3.
DR   ExpressionAtlas; O81235; baseline and differential.
DR   Genevisible; O81235; AT.
DR   GO; GO:0005759; C:mitochondrial matrix; IEA:UniProtKB-SubCell.
DR   GO; GO:0005739; C:mitochondrion; IDA:TAIR.
DR   GO; GO:0005507; F:copper ion binding; IDA:TAIR.
DR   GO; GO:0030145; F:manganese ion binding; IBA:GO_Central.
DR   GO; GO:0046872; F:metal ion binding; IDA:TAIR.
DR   GO; GO:0004784; F:superoxide dismutase activity; IBA:GO_Central.
DR   GO; GO:0042742; P:defense response to bacterium; IEP:TAIR.
DR   GO; GO:0009793; P:embryo development ending in seed dormancy; IMP:TAIR.
DR   GO; GO:0009651; P:response to salt stress; IEP:TAIR.
DR   GO; GO:0010043; P:response to zinc ion; IEP:TAIR.
DR   Gene3D; 1.10.287.990; -; 1.
DR   Gene3D; 2.40.500.20; -; 1.
```

```
DR   InterPro; IPR001189; Mn/Fe_SOD.
DR   InterPro; IPR019833; Mn/Fe_SOD_BS.
DR   InterPro; IPR019832; Mn/Fe_SOD_C.
DR   InterPro; IPR019831; Mn/Fe_SOD_N.
DR   InterPro; IPR036324; Mn/Fe_SOD_N_sf.
DR   InterPro; IPR036314; SOD_C_sf.
DR   Pfam; PF02777; Sod_Fe_C; 1.
DR   Pfam; PF00081; Sod_Fe_N; 1.
DR   PIRSF; PIRSF000349; SODismutase; 1.
DR   PRINTS; PR01703; MNSODISMTASE.
DR   SUPFAM; SSF46609; SSF46609; 1.
DR   SUPFAM; SSF54719; SSF54719; 1.
DR   PROSITE; PS00088; SOD_MN; 1.
PE   1: Evidence at protein level;
KW   3D-structure; Alternative splicing; Complete proteome; Manganese;
KW   Metal-binding; Mitochondrion; Oxidoreductase; Phosphoprotein;
KW   Reference proteome; Transit peptide.
FT   TRANSIT       1     29       Mitochondrion. {ECO:0000250}.
FT   CHAIN        30    231       Superoxide dismutase [Mn] 1,
FT                                mitochondrial.
FT                                /FTId=PRO_0000032891.
FT   METAL        55     55       Manganese. {ECO:0000250}.
FT   METAL       103    103       Manganese. {ECO:0000250}.
FT   METAL       192    192       Manganese. {ECO:0000250}.
FT   METAL       196    196       Manganese. {ECO:0000250}.
FT   MOD_RES     124    124       Phosphoserine.
FT                                {ECO:0000244|PubMed:22092075}.
FT   CONFLICT    169    169       V -> F (in Ref. 1; AAC24832).
FT                                {ECO:0000305}.
FT   CONFLICT    230    230       N -> S (in Ref. 5; AAM62550).
FT                                {ECO:0000305}.
FT   HELIX        40     43       {ECO:0000244|PDB:4C7U}.
FT   TURN         44     46       {ECO:0000244|PDB:4C7U}.
FT   HELIX        49     57       {ECO:0000244|PDB:4C7U}.
FT   HELIX        59     80       {ECO:0000244|PDB:4C7U}.
FT   HELIX        83     88       {ECO:0000244|PDB:4C7U}.
FT   HELIX        90    108       {ECO:0000244|PDB:4C7U}.
FT   HELIX       113    115       {ECO:0000244|PDB:4C7U}.
FT   TURN        116    118       {ECO:0000244|PDB:4C7U}.
FT   HELIX       123    133       {ECO:0000244|PDB:4C7U}.
FT   HELIX       136    149       {ECO:0000244|PDB:4C7U}.
FT   STRAND      152    161       {ECO:0000244|PDB:4C7U}.
FT   TURN        162    165       {ECO:0000244|PDB:4C7U}.
FT   STRAND      166    173       {ECO:0000244|PDB:4C7U}.
FT   HELIX       178    181       {ECO:0000244|PDB:4C7U}.
FT   STRAND      185    192       {ECO:0000244|PDB:4C7U}.
FT   HELIX       195    197       {ECO:0000244|PDB:4C7U}.
FT   HELIX       199    202       {ECO:0000244|PDB:4C7U}.
FT   HELIX       206    212       {ECO:0000244|PDB:4C7U}.
FT   HELIX       213    216       {ECO:0000244|PDB:4C7U}.
FT   HELIX       219    228       {ECO:0000244|PDB:4C7U}.
SQ   SEQUENCE   231 AA;  25444 MW;  2DBD5560A9E8AD7D CRC64;
     MAIRCVASRK TLAGLKETSS RLLRIRGIQT FTLPDLPYDY GALEPAISGE IMQIHHQKHH
     QAYVTNYNNA LEQLDQAVNK GDASTVVKLQ SAIKFNGGGH VNHSIFWKNL APSSEGGGEP
     PKGSLGSAID AHFGSLEGLV KKMSAEGAAV QGSGWVWLGL DKELKKLVVD TTANQDPLVT
     KGGSLVPLVG IDVWEHAYYL QYKNVRPEYL KNVWKVINWK YASEVYEKEN N
```

Mascot: http://www.matrixscience.com/

MATRIX SCIENCE MASCOT Search Results

Protein View: G3PC1_ARATH

Glyceraldehyde-3-phosphate dehydrogenase GAPC1, cytosolic OS=Arabidopsis thaliana OX=3702 GN=GAPC1 PE=1 SV=2

**Database:**	SwissProt
**Score:**	100
**Expect:**	4e-06
**Monoisotopic mass (M_r):**	36891
**Calculated pI:**	6.62
**Taxonomy:**	Arabidopsis thaliana

Sequence similarity is available as an NCBI BLAST search of G3PC1_ARATH against nr.

Search parameters

**Enzyme:**	Trypsin: cuts C-term side of KR unless next residue is P.
**Mass values searched:**	13
**Mass values matched:**	9

Protein sequence coverage: 45%

Matched peptides shown in ***bold red***.

```
  1 MADKKIRIGI NGFGRIGRLV ARVVLQRDDV ELVAVNDPFI TTEYMTYMFK
 51 YDSVHGQWKH NELKIKDEKT LLFGEKPVTV FGIRNPEDIP WAEAGADYVV
101 ESTGVFTDKD KAAAHLKGGA KKVVISAPSK DAPMFVVGVN EHEYKSDLDI
151 VSNASCTTNC LAPLAKVIND RFGIVEGLMT TVHSITATQK TVDGPSMKDW
201 RGGRAASFNI IPSSTGAAKA VGKVLPALNG KLTGMSFRVP TVDVSVVDLT
251 VRLEKAATYD EIKKAIKEES EGKLKGILGY TEDDVVSTDF VGDNRSSIFD
301 AKAGIALSDK FVKLVSWYDN EWGYSSRVVD LIVHMSKA
```

Unformatted sequence string: 338 residues (for pasting into other applications).

Sort by ◉ residue number ○ increasing mass ○ decreasing mass
Show ◉ matched peptides only ○ predicted peptides also

Start – End	Observed	Mr(expt)	Mr(calc)	Delta	M	Peptide
28 – 50	2741.2724	2740.2651	2740.2652	-0.0001	0	R.DDVELVAVNDPFITTEYMTYMFK.Y
70 – 84	1676.9733	1675.9660	1675.9661	-0.0001	0	K.TLLFGEKPVTVFGIR.N
85 – 109	2710.2518	2709.2445	2709.2446	-0.0001	0	R.NPEDIPWAEAGADYVVESTGVFTDK.D
131 – 145	1734.8155	1733.8082	1733.8083	-0.0001	0	K.DAPMFVVGVNEHEYK.S
146 – 166	2136.0311	2135.0238	2135.0238	-0.0000	0	K.SDLDIVSNASCTTNCLAPLAK.V
172 – 190	2033.0735	2032.0662	2032.0663	-0.0001	0	R.FGIVEGLMTTVHSITATQK.T
264 – 264	146.0000	144.9927	146.1055	-1.1128	0	K.K.A
276 – 295	2172.0091	2171.0018	2171.0019	-0.0001	0	K.GILGYTEDDVVSTDFVGDNR.S
314 – 327	1761.7867	1760.7794	1760.7794	-0.0000	0	K.LVSWYDNEWGYSSR.V

No match to: 131.0000, 172.0000, 276.0000, 314.0000

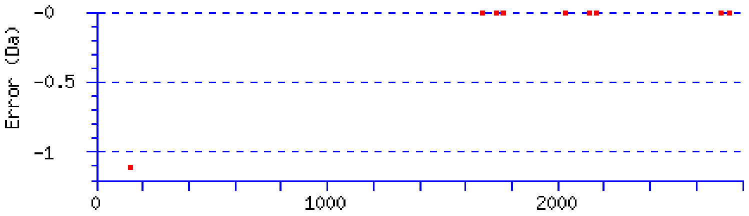

RMS error 2558 ppm

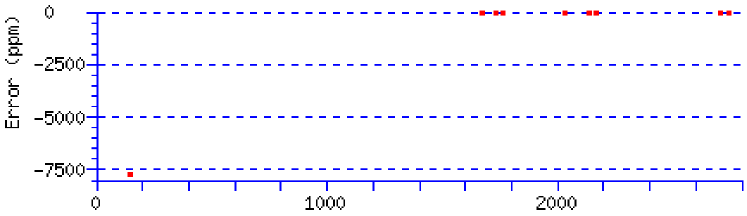

RMS error 2558 ppm

```
ID   G3PC1_ARATH             Reviewed;         338 AA.
AC   P25858; Q0WVE7; Q42352; Q8LAS0; Q9M8W8;
DT   01-MAY-1992, integrated into UniProtKB/Swiss-Prot.
DT   02-MAY-2002, sequence version 2.
DT   31-JUL-2019, entry version 169.
DE   RecName: Full=Glyceraldehyde-3-phosphate dehydrogenase GAPC1, cytosolic;
DE            EC=1.2.1.12;
DE   AltName: Full=NAD-dependent glyceraldehydephosphate dehydrogenase C subunit 1;
GN   Name=GAPC1; Synonyms=GAPC, GAPDH; OrderedLocusNames=At3g04120;
GN   ORFNames=T6K12.26;
OS   Arabidopsis thaliana (Mouse-ear cress).
```

```
OC   Eukaryota; Viridiplantae; Streptophyta; Embryophyta; Tracheophyta;
OC   Spermatophyta; Magnoliopsida; eudicotyledons; Gunneridae;
OC   Pentapetalae; rosids; malvids; Brassicales; Brassicaceae; Camelineae;
OC   Arabidopsis.
OX   NCBI_TaxID=3702;
RN   [1]
RP   NUCLEOTIDE SEQUENCE [GENOMIC DNA / MRNA].
RX   PubMed=1916285; DOI=10.1016/0378-1119(91)90242-4;
RA   Shih M.-C., Heinrich P., Goodman H.M.;
RT   "Cloning and chromosomal mapping of nuclear genes encoding chloroplast
RT   and cytosolic glyceraldehyde-3-phosphate-dehydrogenase from
RT   Arabidopsis thaliana.";
RL   Gene 104:133-138(1991).
RN   [2]
RP   ERRATUM.
RX   PubMed=1398114; DOI=10.1016/0378-1119(92)90290-6;
RA   Shih M.-C., Heinrich P., Goodman H.M.;
RL   Gene 119:317-319(1992).
RN   [3]
RP   NUCLEOTIDE SEQUENCE [LARGE SCALE GENOMIC DNA].
RC   STRAIN=cv. Columbia;
RX   PubMed=11130713; DOI=10.1038/35048706;
RA   Salanoubat M., Lemcke K., Rieger M., Ansorge W., Unseld M.,
RA   Fartmann B., Valle G., Bloecker H., Perez-Alonso M., Obermaier B.,
RA   Delseny M., Boutry M., Grivell L.A., Mache R., Puigdomenech P.,
RA   De Simone V., Choisne N., Artiguenave F., Robert C., Brottier P.,
RA   Wincker P., Cattolico L., Weissenbach J., Saurin W., Quetier F.,
RA   Schaefer M., Mueller-Auer S., Gabel C., Fuchs M., Benes V.,
RA   Wurmbach E., Drzonek H., Erfle H., Jordan N., Bangert S.,
RA   Wiedelmann R., Kranz H., Voss H., Holland R., Brandt P., Nyakatura G.,
RA   Vezzi A., D'Angelo M., Pallavicini A., Toppo S., Simionati B.,
RA   Conrad A., Hornischer K., Kauer G., Loehnert T.-H., Nordsiek G.,
RA   Reichelt J., Scharfe M., Schoen O., Bargues M., Terol J., Climent J.,
RA   Navarro P., Collado C., Perez-Perez A., Ottenwaelder B., Duchemin D.,
RA   Cooke R., Laudie M., Berger-Llauro C., Purnelle B., Masuy D.,
RA   de Haan M., Maarse A.C., Alcaraz J.-P., Cottet A., Casacuberta E.,
RA   Monfort A., Argiriou A., Flores M., Liguori R., Vitale D.,
RA   Mannhaupt G., Haase D., Schoof H., Rudd S., Zaccaria P., Mewes H.-W.,
RA   Mayer K.F.X., Kaul S., Town C.D., Koo H.L., Tallon L.J., Jenkins J.,
RA   Rooney T., Rizzo M., Walts A., Utterback T., Fujii C.Y., Shea T.P.,
RA   Creasy T.H., Haas B., Maiti R., Wu D., Peterson J., Van Aken S.,
RA   Pai G., Militscher J., Sellers P., Gill J.E., Feldblyum T.V.,
RA   Preuss D., Lin X., Nierman W.C., Salzberg S.L., White O., Venter J.C.,
RA   Fraser C.M., Kaneko T., Nakamura Y., Sato S., Kato T., Asamizu E.,
RA   Sasamoto S., Kimura T., Idesawa K., Kawashima K., Kishida Y.,
RA   Kiyokawa C., Kohara M., Matsumoto M., Matsuno A., Muraki A.,
RA   Nakayama S., Nakazaki N., Shinpo S., Takeuchi C., Wada T.,
RA   Watanabe A., Yamada M., Yasuda M., Tabata S.;
RT   "Sequence and analysis of chromosome 3 of the plant Arabidopsis
RT   thaliana.";
RL   Nature 408:820-822(2000).
RN   [4]
RP   GENOME REANNOTATION.
RC   STRAIN=cv. Columbia;
RX   PubMed=27862469; DOI=10.1111/tpj.13415;
RA   Cheng C.Y., Krishnakumar V., Chan A.P., Thibaud-Nissen F., Schobel S.,
RA   Town C.D.;
RT   "Araport11: a complete reannotation of the Arabidopsis thaliana
RT   reference genome.";
RL   Plant J. 89:789-804(2017).
RN   [5]
RP   NUCLEOTIDE SEQUENCE [LARGE SCALE MRNA].
RC   STRAIN=cv. Columbia;
RX   PubMed=14593172; DOI=10.1126/science.1088305;
RA   Yamada K., Lim J., Dale J.M., Chen H., Shinn P., Palm C.J.,
RA   Southwick A.M., Wu H.C., Kim C.J., Nguyen M., Pham P.K., Cheuk R.F.,
RA   Karlin-Newmann G., Liu S.X., Lam B., Sakano H., Wu T., Yu G.,
RA   Miranda M., Quach H.L., Tripp M., Chang C.H., Lee J.M., Toriumi M.J.,
RA   Chan M.M., Tang C.C., Onodera C.S., Deng J.M., Akiyama K., Ansari Y.,
RA   Arakawa T., Banh J., Banno F., Bowser L., Brooks S.Y., Carninci P.,
RA   Chao Q., Choy N., Enju A., Goldsmith A.D., Gurjal M., Hansen N.F.,
RA   Hayashizaki Y., Johnson-Hopson C., Hsuan V.W., Iida K., Karnes M.,
RA   Khan S., Koesema E., Ishida J., Jiang P.X., Jones T., Kawai J.,
RA   Kamiya A., Meyers C., Nakajima M., Narusaka M., Seki M., Sakurai T.,
RA   Satou M., Tamse R., Vaysberg M., Wallender E.K., Wong C., Yamamura Y.,
RA   Yuan S., Shinozaki K., Davis R.W., Theologis A., Ecker J.R.;
RT   "Empirical analysis of transcriptional activity in the Arabidopsis
RT   genome.";
RL   Science 302:842-846(2003).
RN   [6]
RP   NUCLEOTIDE SEQUENCE [LARGE SCALE MRNA].
RC   STRAIN=cv. Columbia;
RA   Totoki Y., Seki M., Ishida J., Nakajima M., Enju A., Kamiya A.,
RA   Narusaka M., Shin-i T., Nakagawa M., Sakamoto N., Oishi K., Kohara Y.,
RA   Kobayashi M., Toyoda A., Sakaki Y., Sakurai T., Iida K., Akiyama K.,
RA   Satou M., Toyoda T., Konagaya A., Carninci P., Kawai J.,
```

RA   Hayashizaki Y., Shinozaki K.;
RT   "Large-scale analysis of RIKEN Arabidopsis full-length (RAFL) cDNAs.";
RL   Submitted (JUL-2006) to the EMBL/GenBank/DDBJ databases.
RN   [7]
RP   NUCLEOTIDE SEQUENCE [LARGE SCALE MRNA].
RA   Brover V.V., Troukhan M.E., Alexandrov N.A., Lu Y.-P., Flavell R.B.,
RA   Feldmann K.A.;
RT   "Full-length cDNA from Arabidopsis thaliana.";
RL   Submitted (MAR-2002) to the EMBL/GenBank/DDBJ databases.
RN   [8]
RP   NUCLEOTIDE SEQUENCE [LARGE SCALE MRNA] OF 181-321.
RC   STRAIN=cv. Columbia;
RX   PubMed=8580968; DOI=10.1046/j.1365-313X.1996.09010101.x;
RA   Cooke R., Raynal M., Laudie M., Grellet F., Delseny M., Morris P.-C.,
RA   Guerrier D., Giraudat J., Quigley F., Clabault G., Li Y.-F., Mache R.,
RA   Krivitzky M., Gy I.J.-J., Kreis M., Lecharny A., Parmentier Y.,
RA   Marbach J., Fleck J., Clement B., Philipps G., Herve C., Bardet C.,
RA   Tremousaygue D., Lescure B., Lacomme C., Roby D., Jourjon M.-F.,
RA   Chabrier P., Charpenteau J.-L., Desprez T., Amselem J., Chiapello H.,
RA   Hoefte H.;
RT   "Further progress towards a catalogue of all Arabidopsis genes:
RT   analysis of a set of 5000 non-redundant ESTs.";
RL   Plant J. 9:101-124(1996).
RN   [9]
RP   TISSUE SPECIFICITY, AND INDUCTION.
RX   PubMed=15533878; DOI=10.1093/jxb/eri020;
RA   Marri L., Sparla F., Pupillo P., Trost P.;
RT   "Co-ordinated gene expression of photosynthetic glyceraldehyde-3-
RT   phosphate dehydrogenase, phosphoribulokinase, and CP12 in Arabidopsis
RT   thaliana.";
RL   J. Exp. Bot. 56:73-80(2005).
RN   [10]
RP   ACTIVITY REGULATION, AND IDENTIFICATION BY MASS SPECTROMETRY.
RX   PubMed=16289945; DOI=10.1016/j.plaphy.2005.07.012;
RA   Hancock J.T., Henson D., Nyirenda M., Desikan R., Harrison J.,
RA   Lewis M., Hughes J., Neill S.J.;
RT   "Proteomic identification of glyceraldehyde 3-phosphate dehydrogenase
RT   as an inhibitory target of hydrogen peroxide in Arabidopsis.";
RL   Plant Physiol. Biochem. 43:828-835(2005).
RN   [11]
RP   CATALYTIC ACTIVITY, ACTIVITY REGULATION, AND SUBCELLULAR LOCATION.
RX   PubMed=18298409; DOI=10.1111/j.1399-3054.2008.01066.x;
RA   Holtgrefe S., Gohlke J., Starmann J., Druce S., Klocke S., Altmann B.,
RA   Wojtera J., Lindermayr C., Scheibe R.;
RT   "Regulation of plant cytosolic glyceraldehyde 3-phosphate
RT   dehydrogenase isoforms by thiol modifications.";
RL   Physiol. Plantarum 133:211-228(2008).
RN   [12]
RP   FUNCTION, AND DISRUPTION PHENOTYPE.
RX   PubMed=18820081; DOI=10.1104/pp.108.128769;
RA   Rius S.P., Casati P., Iglesias A.A., Gomez-Casati D.F.;
RT   "Characterization of Arabidopsis lines deficient in GAPC-1, a
RT   cytosolic NAD-dependent glyceraldehyde-3-phosphate dehydrogenase.";
RL   Plant Physiol. 148:1655-1667(2008).
RN   [13]
RP   IDENTIFICATION BY MASS SPECTROMETRY [LARGE SCALE ANALYSIS].
RC   STRAIN=cv. Columbia;
RX   PubMed=19245862; DOI=10.1016/j.jprot.2009.02.004;
RA   Jones A.M.E., MacLean D., Studholme D.J., Serna-Sanz A.,
RA   Andreasson E., Rathjen J.P., Peck S.C.;
RT   "Phosphoproteomic analysis of nuclei-enriched fractions from
RT   Arabidopsis thaliana.";
RL   J. Proteomics 72:439-451(2009).
RN   [14]
RP   SUBCELLULAR LOCATION.
RX   PubMed=19675149; DOI=10.1104/pp.109.143701;
RA   Munoz-Bertomeu J., Cascales-Minana B., Mulet J.M.,
RA   Baroja-Fernandez E., Pozueta-Romero J., Kuhn J.M., Segura J., Ros R.;
RT   "Plastidial glyceraldehyde-3-phosphate dehydrogenase deficiency leads
RT   to altered root development and affects the sugar and amino acid
RT   balance in Arabidopsis.";
RL   Plant Physiol. 151:541-558(2009).
RN   [15]
RP   ACTIVITY REGULATION, GLUTATHIONYLATION AT CYS-156, AND MUTAGENESIS OF
RP   CYS-156 AND CYS-160.
RX   PubMed=22607208; DOI=10.1042/BJ20120505;
RA   Bedhomme M., Adamo M., Marchand C.H., Couturier J., Rouhier N.,
RA   Lemaire S.D., Zaffagnini M., Trost P.;
RT   "Glutathionylation of cytosolic glyceraldehyde-3-phosphate
RT   dehydrogenase from the model plant Arabidopsis thaliana is reversed by
RT   both glutaredoxins and thioredoxins in vitro.";
RL   Biochem. J. 445:337-347(2012).
RN   [16]
RP   FUNCTION, AND INTERACTION WITH FBA6 AND VDAC3.
RX   PubMed=23316205; DOI=10.3389/fpls.2012.00284;
RA   Wojtera-Kwiczor J., Gross F., Leffers H.M., Kang M., Schneider M.,

```
RA   Scheibe R.;
RT   "Transfer of a redox-signal through the cytosol by redox-dependent
RT   microcompartmentation of glycolytic enzymes at mitochondria and actin
RT   cytoskeleton.";
RL   Front. Plant Sci. 3:284-284(2012).
RN   [17]
RP   FUNCTION, ACTIVITY REGULATION, AND INTERACTION WITH PLDDELTA.
RX   PubMed=22589465; DOI=10.1105/tpc.111.094946;
RA   Guo L., Devaiah S.P., Narasimhan R., Pan X., Zhang Y., Zhang W.,
RA   Wang X.;
RT   "Cytosolic glyceraldehyde-3-phosphate dehydrogenases interact with
RT   phospholipase Ddelta to transduce hydrogen peroxide signals in the
RT   Arabidopsis response to stress.";
RL   Plant Cell 24:2200-2212(2012).
RN   [18]
RP   X-RAY CRYSTALLOGRAPHY (2.30 ANGSTROMS) OF 5-338, AND ACTIVE SITE.
RA   Zaffagnini M., Fermani S., Calvaresi M., Marchand C., Orru R.,
RA   Iommarini L., Sparla F., Bottoni F., Falini G., Lemaire S.D.,
RA   Trost P.;
RT   "Structural determinants of sulfenic acid reactivity in the active
RT   site of cytoplasmic and photosynthetic Arabidopsis GAPDHs from
RT   Arabidopsis thaliana.";
RL   Submitted (MAR-2015) to the PDB data bank.
CC   -!- FUNCTION: Key enzyme in glycolysis that catalyzes the first step
CC       of the pathway by converting D-glyceraldehyde 3-phosphate (G3P)
CC       into 3-phospho-D-glyceroyl phosphate. Essential for the
CC       maintenance of cellular ATP levels and carbohydrate metabolism.
CC       Required for full fertility (PubMed:18820081). Involved in
CC       response to oxidative stress by mediating plant responses to
CC       abscisic acid (ABA) and water deficits through the activation of
CC       PLDDELTA and production of phosphatidic acid (PA), a
CC       multifunctional stress signaling lipid in plants
CC       (PubMed:22589465). Associates with FBA6 to the outer mitochondrial
CC       membrane, in a redox-dependent manner, leading to binding and
CC       bundling of actin. Actin binding and bundling occurs under
CC       oxidizing conditions and is reversible under reducing conditions.
CC       May be part of a redox-dependent retrograde signal transduction
CC       network for adaptation upon oxidative stress (PubMed:23316205).
CC       Binds DNA in vitro. {ECO:0000269|PubMed:18820081,
CC       ECO:0000269|PubMed:22589465, ECO:0000269|PubMed:23316205}.
CC   -!- CATALYTIC ACTIVITY:
CC       Reaction=D-glyceraldehyde 3-phosphate + NAD(+) + phosphate = 3-
CC         phospho-D-glyceroyl phosphate + H(+) + NADH;
CC         Xref=Rhea:RHEA:10300, ChEBI:CHEBI:15378, ChEBI:CHEBI:43474,
CC         ChEBI:CHEBI:57540, ChEBI:CHEBI:57604, ChEBI:CHEBI:57945,
CC         ChEBI:CHEBI:59776; EC=1.2.1.12; Evidence={ECO:0000255|PROSITE-
CC         ProRule:PRU10009, ECO:0000269|PubMed:18298409};
CC   -!- ACTIVITY REGULATION: Inhibition by oxidized glutathione (GSSG), S-
CC       nitrosoglutathione (GSNO), hydrogen peroxide and sodium
CC       nitroprusside (SNP). {ECO:0000269|PubMed:16289945,
CC       ECO:0000269|PubMed:18298409, ECO:0000269|PubMed:22589465,
CC       ECO:0000269|PubMed:22607208}.
CC   -!- PATHWAY: Carbohydrate degradation; glycolysis; pyruvate from D-
CC       glyceraldehyde 3-phosphate: step 1/5.
CC   -!- SUBUNIT: Homotetramer (By similarity). Interacts with PLDDELTA
CC       (PubMed:22589465). Interacts with FBA6 and VDAC3
CC       (PubMed:23316205). {ECO:0000250, ECO:0000269|PubMed:22589465,
CC       ECO:0000269|PubMed:23316205}.
CC   -!- SUBCELLULAR LOCATION: Cytoplasm. Nucleus.
CC   -!- TISSUE SPECIFICITY: Expressed in leaves, stems and siliques and at
CC       lower levels in roots and flowers. {ECO:0000269|PubMed:15533878}.
CC   -!- INDUCTION: Not repressed by darkness or sucrose.
CC       {ECO:0000269|PubMed:15533878}.
CC   -!- PTM: S-glutathionylation at Cys-156 in the presence of oxidized
CC       glutathione (GSSG). S-nitrosylation in the presence of S-
CC       nitrosoglutathione (GSNO) or sodium nitroprusside (SNP). These
CC       reactions may be both a protective mechanism against irreversible
CC       oxidation and a mean to store inhibited enzyme in a recoverable
CC       form. Glutathionylation is reversed by both glutaredoxins and
CC       thioredoxins in vitro. {ECO:0000269|PubMed:22607208}.
CC   -!- DISRUPTION PHENOTYPE: Delayed growth, small siliques with defects
CC       in fertility, and alterations of seed and fruit development.
CC       Reduced respiratory rates, pyruvate levels and Krebs cycle
CC       intermediates. Increased reactive oxygen species levels.
CC       {ECO:0000269|PubMed:18820081}.
CC   -!- MISCELLANEOUS: Plants contain three types of GAPDH: NAD-dependent
CC       cytosolic forms which participate in glycolysis, NAD-dependent
CC       chloroplastic forms which participate in plastidic glycolysis and
CC       NADP-dependent chloroplastic forms which participate in the
CC       photosynthetic reductive pentose phosphate pathway (Calvin-Benson
CC       cycle). All the forms are encoded by distinct genes.
CC   -!- SIMILARITY: Belongs to the glyceraldehyde-3-phosphate
CC       dehydrogenase family. {ECO:0000305}.
DR   EMBL; M64116; AAA32794.1; -; mRNA.
DR   EMBL; M64119; AAA32796.1; -; Genomic_DNA.
DR   EMBL; AC016829; AAF26801.1; -; Genomic_DNA.
```

```
DR   EMBL; CP002686; AEE74039.1; -; Genomic_DNA.
DR   EMBL; AY052267; AAK97737.1; -; mRNA.
DR   EMBL; AY060521; AAL31134.1; -; mRNA.
DR   EMBL; AY140084; AAM98225.1; -; mRNA.
DR   EMBL; AK226804; BAE98901.1; -; mRNA.
DR   EMBL; AY087651; AAM65189.1; -; mRNA.
DR   EMBL; F20074; CAA23391.1; -; mRNA.
DR   PIR; JQ1287; JQ1287.
DR   RefSeq; NP_187062.1; NM_111283.4.
DR   PDB; 4Z0H; X-ray; 2.30 A; O/R=5-338.
DR   PDBsum; 4Z0H; -.
DR   SMR; P25858; -.
DR   BioGrid; 4902; 11.
DR   IntAct; P25858; 2.
DR   MINT; P25858; -.
DR   STRING; 3702.AT3G04120.1; -.
DR   iPTMnet; P25858; -.
DR   PaxDb; P25858; -.
DR   PRIDE; P25858; -.
DR   EnsemblPlants; AT3G04120.1; AT3G04120.1; AT3G04120.
DR   GeneID; 819567; -.
DR   Gramene; AT3G04120.1; AT3G04120.1; AT3G04120.
DR   KEGG; ath:AT3G04120; -.
DR   Araport; AT3G04120; -.
DR   TAIR; locus:2103085; AT3G04120.
DR   eggNOG; KOG0657; Eukaryota.
DR   eggNOG; COG0057; LUCA.
DR   HOGENOM; HOG000071678; -.
DR   InParanoid; P25858; -.
DR   KO; K00134; -.
DR   OMA; KYDPSSM; -.
DR   OrthoDB; 945145at2759; -.
DR   PhylomeDB; P25858; -.
DR   BRENDA; 1.2.1.12; 399.
DR   UniPathway; UPA00109; UER00184.
DR   PRO; PR:P25858; -.
DR   Proteomes; UP000006548; Chromosome 3.
DR   ExpressionAtlas; P25858; baseline and differential.
DR   Genevisible; P25858; AT.
DR   GO; GO:0048046; C:apoplast; IDA:TAIR.
DR   GO; GO:0009507; C:chloroplast; IDA:TAIR.
DR   GO; GO:0005829; C:cytosol; IDA:TAIR.
DR   GO; GO:0016020; C:membrane; IDA:TAIR.
DR   GO; GO:0005740; C:mitochondrial envelope; IDA:TAIR.
DR   GO; GO:0005739; C:mitochondrion; IDA:TAIR.
DR   GO; GO:0005634; C:nucleus; IDA:TAIR.
DR   GO; GO:0005886; C:plasma membrane; IDA:TAIR.
DR   GO; GO:0005774; C:vacuolar membrane; IDA:TAIR.
DR   GO; GO:0005507; F:copper ion binding; IDA:TAIR.
DR   GO; GO:0003677; F:DNA binding; IEA:UniProtKB-KW.
DR   GO; GO:0004365; F:glyceraldehyde-3-phosphate dehydrogenase (NAD+) (phosphorylating) activity; IBA:GO_Central.
DR   GO; GO:0008886; F:glyceraldehyde-3-phosphate dehydrogenase (NADP+) (non-phosphorylating) activity; IDA:TAIR.
DR   GO; GO:0051287; F:NAD binding; IEA:InterPro.
DR   GO; GO:0050661; F:NADP binding; IEA:InterPro.
DR   GO; GO:0010154; P:fruit development; IMP:TAIR.
DR   GO; GO:0006094; P:gluconeogenesis; TAS:TAIR.
DR   GO; GO:0006096; P:glycolytic process; IDA:TAIR.
DR   GO; GO:0046686; P:response to cadmium ion; IEP:TAIR.
DR   GO; GO:0009408; P:response to heat; IEP:TAIR.
DR   GO; GO:0042542; P:response to hydrogen peroxide; IDA:TAIR.
DR   GO; GO:0006979; P:response to oxidative stress; IDA:TAIR.
DR   GO; GO:0051775; P:response to redox state; IDA:TAIR.
DR   GO; GO:0009651; P:response to salt stress; IEP:TAIR.
DR   GO; GO:0009744; P:response to sucrose; IEP:TAIR.
DR   GO; GO:0048316; P:seed development; IMP:TAIR.
DR   InterPro; IPR020831; GlycerAld/Erythrose_P_DH.
DR   InterPro; IPR020830; GlycerAld_3-P_DH_AS.
DR   InterPro; IPR020829; GlycerAld_3-P_DH_cat.
DR   InterPro; IPR020828; GlycerAld_3-P_DH_NAD(P)-bd.
DR   InterPro; IPR006424; Glyceraldehyde-3-P_DH_1.
DR   InterPro; IPR036291; NAD(P)-bd_dom_sf.
DR   PANTHER; PTHR10836; PTHR10836; 1.
DR   Pfam; PF02800; Gp_dh_C; 1.
DR   Pfam; PF00044; Gp_dh_N; 1.
DR   PIRSF; PIRSF000149; GAP_DH; 1.
DR   PRINTS; PR00078; G3PDHDRGNASE.
DR   SMART; SM00846; Gp_dh_N; 1.
DR   SUPFAM; SSF51735; SSF51735; 1.
DR   TIGRFAMs; TIGR01534; GAPDH-I; 1.
DR   PROSITE; PS00071; GAPDH; 1.
PE   1: Evidence at protein level;
KW   3D-structure; Complete proteome; Cytoplasm; DNA-binding;
KW   Glutathionylation; Glycolysis; NAD; Nucleus; Oxidoreductase;
KW   Reference proteome; S-nitrosylation; Stress response.
FT   CHAIN         1    338       Glyceraldehyde-3-phosphate dehydrogenase
FT                                GAPC1, cytosolic.
```

```
FT                                   /FTId=PRO_0000145594.
FT   NP_BIND      15     16       NAD. {ECO:0000250}.
FT   REGION      155    157       Glyceraldehyde 3-phosphate binding.
FT                                {ECO:0000250}.
FT   REGION      215    216       Glyceraldehyde 3-phosphate binding.
FT                                {ECO:0000250}.
FT   ACT_SITE    156    156       Nucleophile. {ECO:0000269|Ref.18}.
FT   BINDING      37     37       NAD. {ECO:0000250}.
FT   BINDING      84     84       NAD; via carbonyl oxygen. {ECO:0000250}.
FT   BINDING     186    186       Glyceraldehyde 3-phosphate.
FT                                {ECO:0000250}.
FT   BINDING     238    238       Glyceraldehyde 3-phosphate.
FT                                {ECO:0000250}.
FT   BINDING     320    320       NAD. {ECO:0000250}.
FT   SITE        183    183       Activates thiol group during catalysis.
FT                                {ECO:0000250}.
FT   MOD_RES     156    156       S-glutathionyl cysteine; transient;
FT                                alternate. {ECO:0000269|PubMed:22607208}.
FT   MOD_RES     156    156       S-nitrosocysteine; transient; alternate.
FT                                {ECO:0000250|UniProtKB:Q9FX54}.
FT   MOD_RES     160    160       S-nitrosocysteine; transient.
FT                                {ECO:0000250|UniProtKB:Q9FX54}.
FT   MUTAGEN     156    156       C->S: Loss of activity. Loss of
FT                                glutathionylation.
FT                                {ECO:0000269|PubMed:22607208}.
FT   MUTAGEN     160    160       C->S: No effect on the activity.
FT                                {ECO:0000269|PubMed:22607208}.
FT   CONFLICT    127    127       A -> E (in Ref. 1; AAA32794/AAA32796).
FT                                {ECO:0000305}.
FT   CONFLICT    136    136       V -> F (in Ref. 7; AAM65189).
FT                                {ECO:0000305}.
FT   CONFLICT    260    260       D -> E (in Ref. 1; AAA32794/AAA32796).
FT                                {ECO:0000305}.
FT   CONFLICT    325    325       S -> N (in Ref. 7; AAM65189).
FT                                {ECO:0000305}.
FT   STRAND        7     12       {ECO:0000244|PDB:4Z0H}.
FT   HELIX        15     26       {ECO:0000244|PDB:4Z0H}.
FT   STRAND       31     36       {ECO:0000244|PDB:4Z0H}.
FT   HELIX        42     50       {ECO:0000244|PDB:4Z0H}.
FT   TURN         53     55       {ECO:0000244|PDB:4Z0H}.
FT   STRAND       63     66       {ECO:0000244|PDB:4Z0H}.
FT   TURN         67     69       {ECO:0000244|PDB:4Z0H}.
FT   STRAND       70     73       {ECO:0000244|PDB:4Z0H}.
FT   STRAND       76     81       {ECO:0000244|PDB:4Z0H}.
FT   HELIX        86     88       {ECO:0000244|PDB:4Z0H}.
FT   HELIX        91     94       {ECO:0000244|PDB:4Z0H}.
FT   STRAND       97    101       {ECO:0000244|PDB:4Z0H}.
FT   STRAND      103    105       {ECO:0000244|PDB:4Z0H}.
FT   HELIX       109    112       {ECO:0000244|PDB:4Z0H}.
FT   HELIX       114    117       {ECO:0000244|PDB:4Z0H}.
FT   STRAND      121    127       {ECO:0000244|PDB:4Z0H}.
FT   STRAND      130    132       {ECO:0000244|PDB:4Z0H}.
FT   TURN        137    139       {ECO:0000244|PDB:4Z0H}.
FT   HELIX       141    143       {ECO:0000244|PDB:4Z0H}.
FT   STRAND      150    152       {ECO:0000244|PDB:4Z0H}.
FT   HELIX       156    171       {ECO:0000244|PDB:4Z0H}.
FT   STRAND      174    183       {ECO:0000244|PDB:4Z0H}.
FT   STRAND      191    193       {ECO:0000244|PDB:4Z0H}.
FT   HELIX       200    203       {ECO:0000244|PDB:4Z0H}.
FT   HELIX       206    208       {ECO:0000244|PDB:4Z0H}.
FT   STRAND      211    214       {ECO:0000244|PDB:4Z0H}.
FT   HELIX       217    221       {ECO:0000244|PDB:4Z0H}.
FT   TURN        222    224       {ECO:0000244|PDB:4Z0H}.
FT   HELIX       226    228       {ECO:0000244|PDB:4Z0H}.
FT   STRAND      231    238       {ECO:0000244|PDB:4Z0H}.
FT   STRAND      245    255       {ECO:0000244|PDB:4Z0H}.
FT   HELIX       259    271       {ECO:0000244|PDB:4Z0H}.
FT   TURN        272    277       {ECO:0000244|PDB:4Z0H}.
FT   STRAND      278    281       {ECO:0000244|PDB:4Z0H}.
FT   HELIX       287    290       {ECO:0000244|PDB:4Z0H}.
FT   STRAND      295    300       {ECO:0000244|PDB:4Z0H}.
FT   TURN        301    303       {ECO:0000244|PDB:4Z0H}.
FT   STRAND      305    308       {ECO:0000244|PDB:4Z0H}.
FT   STRAND      311    318       {ECO:0000244|PDB:4Z0H}.
FT   HELIX       322    335       {ECO:0000244|PDB:4Z0H}.
SQ   SEQUENCE   338 AA;  36914 MW;  4186F65E1F1EE96F CRC64;
     MADKKIRIGI NGFGRIGRLV ARVVLQRDDV ELVAVNDPFI TTEYMTYMFK YDSVHGQWKH
     NELKIKDEKT LLFGEKPVTV FGIRNPEDIP WAEAGADYVV ESTGVFTDKD KAAAHLKGGA
     KKVVISAPSK DAPMFVVGVN EHEYKSDLDI VSNASCTTNC LAPLAKVIND RFGIVEGLMT
     TVHSITATQK TVDGPSMKDW RGGRAASFNI IPSSTGAAKA VGKVLPALNG KLTGMSFRVP
     TVDVSVVDLT VRLEKAATYD EIKKAIKEES EGKLKGILGY TEDDVVSTDF VGDNRSSIFD
     AKAGIALSDK FVKLVSWYDN EWGYSSRVVD LIVHMSKA
```

MASCOT Search Results

Protein View: TBA6_ARATH

Tubulin alpha-6 chain OS=Arabidopsis thaliana OX=3702 GN=TUBA4 PE=2 SV=2

**Database:**	SwissProt
**Score:**	115
**Expect:**	1.3e-07
**Monoisotopic mass (M_r):**	49509
**Calculated pI:**	4.93
**Taxonomy:**	Arabidopsis thaliana

Sequence similarity is available as an NCBI BLAST search of TBA4_ARATH against nr.

Search parameters

**Enzyme:**	Trypsin: cuts C-term side of KR unless next residue is P.
**Mass values searched:**	23
**Mass values matched:**	12

Protein sequence coverage: 56%

Matched peptides shown in ***bold red***.

```
  1 MRECISIHIG QAGIQVGNAC WELYCLEHGI QPDGQMPSDK TVGGGDDAFN
 51 TFFSETGAGK HVPRAVFVDL EPTVIDEVRT GTYRQLFHPE QLISGKEDAA
101 NNFARGHYTI GKEIVDLCLD RIRKLADNCT GLQGFLVFNA VGGGTGSGLG
151 SLLLERLSVD YGKKSKLGFT VYPSPQVSTS VVEPYNSVLS THSLLEHTDV
201 SILLDNEAIY DICRRSLSIE RPTYTNLNRL VSQVISSLTA SLRFDGALNV
251 DVTEFQTNLV PYPRIHFMLS SYAPVISAEK AFHEQLSVAE ITNSAFEPAS
301 MMAKCDPRHG KYMACCLMYR GDVVPKDVNA AVGTIKTKRT IQFVDWCPTG
351 FKCGINYQPP TVVPGGDLAK VQRAVCMISN STSVAEVFSR IDHKFDLMYA
401 KRAFVHWYVG EGMEEGEFSE AREDLAALEK DYEEVGAEGG DDEDDEGEEY
```

Unformatted sequence string: 450 residues (for pasting into other applications).

Sort by ◉ residue number ○ increasing mass ○ decreasing mass
Show ◉ matched peptides only ○ predicted peptides also

Start – End	Observed	Mr(expt)	Mr(calc)	Delta	M	Peptide
41 – 60	1977.8824	1976.8751	1976.8752	-0.0001	0	K.TVGGGDDAFNTFFSETGAGK.H
65 – 79	1701.9057	1700.8984	1700.8985	-0.0001	0	R.AVFVDLEPTVIDEVR.T
125 – 156	3136.6095	3135.6022	3135.6023	-0.0001	0	K.LADNCTGLQGFLVFNAVGGGTGSGLGSLLLER.L
167 – 214	5321.6562	5320.6489	5320.6490	-0.0000	0	K.LGFTVYPSPQVSTSVVEPYNSVLSTHSLLEHTDVSILLDNEAIYDICR.R
244 – 264	2395.1928	2394.1855	2394.1856	-0.0001	0	R.FDGALNVDVTEFQTNLVPYPR.I
265 – 280	1792.9302	1791.9229	1791.9229	0.0000	0	R.IHFMLSSYAPVISAEK.A
281 – 304	2609.2374	2608.2301	2608.2301	0.0000	0	K.AFHEQLSVAEITNSAFEPASMMAK.C
353 – 370	1828.9261	1827.9188	1827.9189	-0.0001	0	K.CGINYQPPTVVPGGDLAK.V
371 – 373	403.0000	401.9927	401.2387	0.7541	0	K.VQR.A
374 – 390	1800.8619	1799.8546	1799.8546	0.0000	0	R.AVCMISNSTSVAEVFSR.I
403 – 422	2330.0182	2329.0109	2329.0110	-0.0000	0	R.AFVHWYVGEGMEEGEFSEAR.E
431 – 450	2221.7687	2220.7614	2220.7615	-0.0000	0	K.DYEEVGAEGGDDEDDEGEEY.-

No match to: 125.0000, 167.0000, 216.0000, 244.0000, 265.0000, 281.0000, 353.0000, 374.0000, 431.0000, 1690.8871, 4109.8976

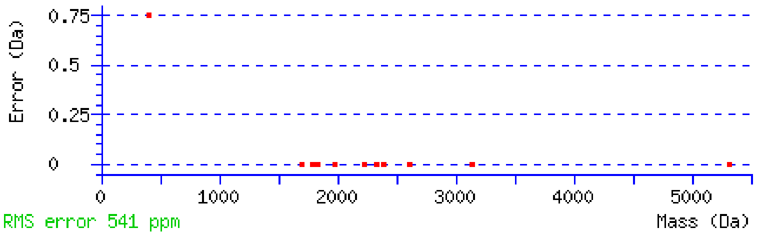

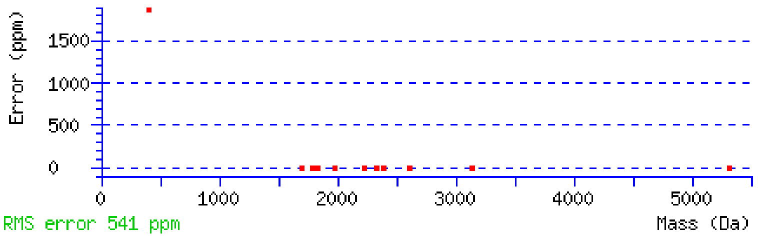

```
ID   TBA4_ARATH              Reviewed;         450 AA.
AC   Q0WV25; P29510;
DT   03-OCT-2012, integrated into UniProtKB/Swiss-Prot.
DT   03-OCT-2012, sequence version 2.
DT   31-JUL-2019, entry version 83.
DE   RecName: Full=Tubulin alpha-4 chain;
GN   Name=TUBA4; Synonyms=TUA4; OrderedLocusNames=At1g04820;
GN   ORFNames=F13M7.19;
OS   Arabidopsis thaliana (Mouse-ear cress).
OC   Eukaryota; Viridiplantae; Streptophyta; Embryophyta; Tracheophyta;
OC   Spermatophyta; Magnoliopsida; eudicotyledons; Gunneridae;
OC   Pentapetalae; rosids; malvids; Brassicales; Brassicaceae; Camelineae;
OC   Arabidopsis.
OX   NCBI_TaxID=3702;
RN   [1]
RP   NUCLEOTIDE SEQUENCE [GENOMIC DNA].
RC   STRAIN=cv. Columbia;
RX   PubMed=1498608; DOI=10.1105/tpc.4.5.539;
RA   Kopczak S.D., Haas N.A., Hussey P.J., Silflow C.D., Snustad D.P.;
RT   "The small genome of Arabidopsis contains at least six expressed
RT   alpha-tubulin genes.";
RL   Plant Cell 4:539-547(1992).
RN   [2]
RP   NUCLEOTIDE SEQUENCE [LARGE SCALE GENOMIC DNA].
RC   STRAIN=cv. Columbia;
RX   PubMed=11130712; DOI=10.1038/35048500;
RA   Theologis A., Ecker J.R., Palm C.J., Federspiel N.A., Kaul S.,
RA   White O., Alonso J., Altafi H., Araujo R., Bowman C.L., Brooks S.Y.,
RA   Buehler E., Chan A., Chao Q., Chen H., Cheuk R.F., Chin C.W.,
RA   Chung M.K., Conn L., Conway A.B., Conway A.R., Creasy T.H., Dewar K.,
RA   Dunn P., Etgu P., Feldblyum T.V., Feng J.-D., Fong B., Fujii C.Y.,
RA   Gill J.E., Goldsmith A.D., Haas B., Hansen N.F., Hughes B., Huizar L.,
RA   Hunter J.L., Jenkins J., Johnson-Hopson C., Khan S., Khaykin E.,
RA   Kim C.J., Koo H.L., Kremenetskaia I., Kurtz D.B., Kwan A., Lam B.,
RA   Langin-Hooper S., Lee A., Lee J.M., Lenz C.A., Li J.H., Li Y.-P.,
RA   Lin X., Liu S.X., Liu Z.A., Luros J.S., Maiti R., Marziali A.,
RA   Militscher J., Miranda M., Nguyen M., Nierman W.C., Osborne B.I.,
RA   Pai G., Peterson J., Pham P.K., Rizzo M., Rooney T., Rowley D.,
RA   Sakano H., Salzberg S.L., Schwartz J.R., Shinn P., Southwick A.M.,
RA   Sun H., Tallon L.J., Tambunga G., Toriumi M.J., Town C.D.,
RA   Utterback T., Van Aken S., Vaysberg M., Vysotskaia V.S., Walker M.,
RA   Wu D., Yu G., Fraser C.M., Venter J.C., Davis R.W.;
RT   "Sequence and analysis of chromosome 1 of the plant Arabidopsis
RT   thaliana.";
RL   Nature 408:816-820(2000).
RN   [3]
RP   GENOME REANNOTATION.
RC   STRAIN=cv. Columbia;
RX   PubMed=27862469; DOI=10.1111/tpj.13415;
RA   Cheng C.Y., Krishnakumar V., Chan A.P., Thibaud-Nissen F., Schobel S.,
RA   Town C.D.;
RT   "Araport11: a complete reannotation of the Arabidopsis thaliana
RT   reference genome.";
RL   Plant J. 89:789-804(2017).
RN   [4]
RP   NUCLEOTIDE SEQUENCE [LARGE SCALE MRNA].
RC   STRAIN=cv. Columbia;
RX   PubMed=14593172; DOI=10.1126/science.1088305;
RA   Yamada K., Lim J., Dale J.M., Chen H., Shinn P., Palm C.J.,
RA   Southwick A.M., Wu H.C., Kim C.J., Nguyen M., Pham P.K., Cheuk R.F.,
RA   Karlin-Newmann G., Liu S.X., Lam B., Sakano H., Wu T., Yu G.,
RA   Miranda M., Quach H.L., Tripp M., Chang C.H., Lee J.M., Toriumi M.J.,
RA   Chan M.M., Tang C.C., Onodera C.S., Deng J.M., Akiyama K., Ansari Y.,
```

```
RA   Arakawa T., Banh J., Banno F., Bowser L., Brooks S.Y., Carninci P.,
RA   Chao Q., Choy N., Enju A., Goldsmith A.D., Gurjal M., Hansen N.F.,
RA   Hayashizaki Y., Johnson-Hopson C., Hsuan V.W., Iida K., Karnes M.,
RA   Khan S., Koesema E., Ishida J., Jiang P.X., Jones T., Kawai J.,
RA   Kamiya A., Meyers C., Nakajima M., Narusaka M., Seki M., Sakurai T.,
RA   Satou M., Tamse R., Vaysberg M., Wallender E.K., Wong C., Yamamura Y.,
RA   Yuan S., Shinozaki K., Davis R.W., Theologis A., Ecker J.R.;
RT   "Empirical analysis of transcriptional activity in the Arabidopsis
RT   genome.";
RL   Science 302:842-846(2003).
RN   [5]
RP   NUCLEOTIDE SEQUENCE [LARGE SCALE MRNA].
RC   STRAIN=cv. Columbia;
RA   Totoki Y., Seki M., Ishida J., Nakajima M., Enju A., Kamiya A.,
RA   Narusaka M., Shin-i T., Nakagawa M., Sakamoto N., Oishi K., Kohara Y.,
RA   Kobayashi M., Toyoda A., Sakaki Y., Sakurai T., Iida K., Akiyama K.,
RA   Satou M., Toyoda T., Konagaya A., Carninci P., Kawai J.,
RA   Hayashizaki Y., Shinozaki K.;
RT   "Large-scale analysis of RIKEN Arabidopsis full-length (RAFL) cDNAs.";
RL   Submitted (JUL-2006) to the EMBL/GenBank/DDBJ databases.
CC   -!- FUNCTION: Tubulin is the major constituent of microtubules. It
CC       binds two moles of GTP, one at an exchangeable site on the beta
CC       chain and one at a non-exchangeable site on the alpha chain.
CC   -!- SUBUNIT: Dimer of alpha and beta chains. A typical microtubule is
CC       a hollow water-filled tube with an outer diameter of 25 nm and an
CC       inner diameter of 15 nM. Alpha-beta heterodimers associate head-
CC       to-tail to form protofilaments running lengthwise along the
CC       microtubule wall with the beta-tubulin subunit facing the
CC       microtubule plus end conferring a structural polarity.
CC       Microtubules usually have 13 protofilaments but different
CC       protofilament numbers can be found in some organisms and
CC       specialized cells.
CC   -!- SUBCELLULAR LOCATION: Cytoplasm, cytoskeleton.
CC   -!- PTM: Undergoes a tyrosination/detyrosination cycle, the cyclic
CC       removal and re-addition of a C-terminal tyrosine residue by the
CC       enzymes tubulin tyrosine carboxypeptidase (TTCP) and tubulin
CC       tyrosine ligase (TTL), respectively. {ECO:0000250}.
CC   -!- PTM: Acetylation of alpha chains at Lys-40 stabilizes microtubules
CC       and affects affinity and processivity of microtubule motors. This
CC       modification has a role in multiple cellular functions, ranging
CC       from cell motility, cell cycle progression or cell differentiation
CC       to intracellular trafficking and signaling (By similarity).
CC       {ECO:0000250}.
CC   -!- MISCELLANEOUS: There are six genes coding for alpha-tubulin. The
CC       sequences coded by genes 2 and 4 are identical.
CC   -!- SIMILARITY: Belongs to the tubulin family. {ECO:0000305}.
DR   EMBL; M84697; AAA32890.1; -; Genomic_DNA.
DR   EMBL; AC004809; AAF40454.1; -; Genomic_DNA.
DR   EMBL; CP002684; AEE27747.1; -; Genomic_DNA.
DR   EMBL; AY058199; AAL25612.1; -; mRNA.
DR   EMBL; AY142005; AAM98269.1; -; mRNA.
DR   EMBL; AK226954; BAE99023.1; -; mRNA.
DR   PIR; JQ1594; JQ1594.
DR   RefSeq; NP_171974.1; NM_100360.4.
DR   RefSeq; NP_175423.1; NM_103889.3.
DR   SMR; Q0WV25; -.
DR   BioGrid; 24640; 8.
DR   BioGrid; 26650; 3.
DR   IntAct; Q0WV25; 2.
DR   PRIDE; Q0WV25; -.
DR   EnsemblPlants; AT1G04820.1; AT1G04820.1; AT1G04820.
DR   EnsemblPlants; AT1G50010.1; AT1G50010.1; AT1G50010.
DR   GeneID; 839405; -.
DR   GeneID; 841425; -.
DR   Gramene; AT1G04820.1; AT1G04820.1; AT1G04820.
DR   Gramene; AT1G50010.1; AT1G50010.1; AT1G50010.
DR   KEGG; ath:AT1G04820; -.
DR   KEGG; ath:AT1G50010; -.
DR   Araport; AT1G04820; -.
DR   eggNOG; KOG1376; Eukaryota.
DR   eggNOG; COG5023; LUCA.
DR   InParanoid; Q0WV25; -.
DR   KO; K07374; -.
DR   OrthoDB; 514396at2759; -.
DR   PhylomeDB; Q0WV25; -.
DR   PRO; PR:Q0WV25; -.
DR   Proteomes; UP000006548; Chromosome 1.
DR   ExpressionAtlas; Q0WV25; baseline and differential.
DR   Genevisible; Q0WV25; AT.
DR   GO; GO:0005737; C:cytoplasm; IEA:UniProtKB-KW.
DR   GO; GO:0005874; C:microtubule; IEA:UniProtKB-KW.
DR   GO; GO:0005525; F:GTP binding; IEA:UniProtKB-KW.
DR   GO; GO:0003924; F:GTPase activity; IEA:InterPro.
DR   GO; GO:0005200; F:structural constituent of cytoskeleton; IEA:InterPro.
DR   GO; GO:0007017; P:microtubule-based process; IEA:InterPro.
DR   Gene3D; 1.10.287.600; -; 1.
```

```
DR   Gene3D; 3.30.1330.20; -; 1.
DR   Gene3D; 3.40.50.1440; -; 1.
DR   InterPro; IPR002452; Alpha_tubulin.
DR   InterPro; IPR008280; Tub_FtsZ_C.
DR   InterPro; IPR000217; Tubulin.
DR   InterPro; IPR018316; Tubulin/FtsZ_2-layer-sand-dom.
DR   InterPro; IPR037103; Tubulin/FtsZ_C_sf.
DR   InterPro; IPR036525; Tubulin/FtsZ_GTPase_sf.
DR   InterPro; IPR023123; Tubulin_C.
DR   InterPro; IPR017975; Tubulin_CS.
DR   InterPro; IPR003008; Tubulin_FtsZ_GTPase.
DR   PANTHER; PTHR11588; PTHR11588; 1.
DR   Pfam; PF00091; Tubulin; 1.
DR   Pfam; PF03953; Tubulin_C; 1.
DR   PRINTS; PR01162; ALPHATUBULIN.
DR   PRINTS; PR01161; TUBULIN.
DR   SMART; SM00864; Tubulin; 1.
DR   SMART; SM00865; Tubulin_C; 1.
DR   SUPFAM; SSF52490; SSF52490; 1.
DR   SUPFAM; SSF55307; SSF55307; 1.
DR   PROSITE; PS00227; TUBULIN; 1.
PE   2: Evidence at transcript level;
KW   Acetylation; Complete proteome; Cytoplasm; Cytoskeleton; GTP-binding;
KW   Microtubule; Nucleotide-binding; Phosphoprotein; Reference proteome.
FT   CHAIN         1    450       Tubulin alpha-4 chain.
FT                                /FTId=PRO_0000419521.
FT   NP_BIND     142    148       GTP. {ECO:0000255}.
FT   SITE        450    450       Involved in polymerization.
FT   MOD_RES     349    349       Phosphothreonine.
FT                                {ECO:0000250|UniProtKB:Q56WH1}.
FT   CONFLICT    433    433       E -> G (in Ref. 5; BAE99023).
FT                                {ECO:0000305}.
SQ   SEQUENCE   450 AA;  49541 MW;  0E319AD3CC0E5523 CRC64;
     MRECISIHIG QAGIQVGNAC WELYCLEHGI QPDGQMPSDK TVGGGDDAFN TFFSETGAGK
     HVPRAVFVDL EPTVIDEVRT GTYRQLFHPE QLISGKEDAA NNFARGHYTI GKEIVDLCLD
     RIRKLADNCT GLQGFLVFNA VGGGTGSGLG SLLLERLSVD YGKKSKLGFT VYPSPQVSTS
     VVEPYNSVLS THSLLEHTDV SILLDNEAIY DICRRSLSIE RPTYTNLNRL VSQVISSLTA
     SLRFDGALNV DVTEFQTNLV PYPRIHFMLS SYAPVISAEK AFHEQLSVAE ITNSAFEPAS
     MMAKCDPRHG KYMACCLMYR GDVVPKDVNA AVGTIKTKRT IQFVDWCPTG FKCGINYQPP
     TVVPGGDLAK VQRAVCMISN STSVAEVFSR IDHKFDLMYA KRAFVHWYVG EGMEEGEFSE
     AREDLAALEK DYEEVGAEGG DDEDDEGEEY
```

Mascot: http://www.matrixscience.com/

MATRIX SCIENCE MASCOT Search Results

Protein View: EIF3I_ARATH

Eukaryotic translation initiation factor 3 subunit I OS=Arabidopsis thaliana OX=3702 GN=TIF3I1 PE=2 SV=2

**Database:**	SwissProt
**Score:**	125
**Expect:**	1.3e-08
**Monoisotopic mass (M_r):**	36365
**Calculated pI:**	6.50
**Taxonomy:**	Arabidopsis thaliana

Sequence similarity is available as an NCBI BLAST search of EIF3I_ARATH against nr.

Search parameters

**Enzyme:**	Trypsin: cuts C-term side of KR unless next residue is P.
**Mass values searched:**	20
**Mass values matched:**	13

Protein sequence coverage: 58%

Matched peptides shown in ***bold red***.

```
  1 MRPILMKGHE RPLTFLRYNR EGDLLFSCAK DHTPTLWFAD NGERLGTYRG
 51 HNGAVWCCDV SRDSSRLITG SADQTAKLWD VKSGKELFTF KFNAPTRSVD
101 FAVGDRLAVI TTDHFVDRTA AIHVKRIAED PEEQDAESVL VLHCPDGKKR
151 INRAVWGPLN QTIVSGGEDK VIRIWDAETG KLLKQSDEEV GHKKDITSLC
201 KAADDSHFLT GSLDKTAKLW DMRTLTLLKT YTTVVPVNAV SLSPLLNHVV
251 LGGGQDASAV TTTDHRAGKF EAKFYDKILQ EEIGGVKGHF GPINALAFNP
301 DGKSFSSGGE DGYVRLHHFD SDYFNIKI
```

Unformatted sequence string: 328 residues (for pasting into other applications).

Sort by ◉ residue number ○ increasing mass ○ decreasing mass
Show ◉ matched peptides only ○ predicted peptides also

Start – End	Observed	Mr(expt)	Mr(calc)	Delta	M	Peptide
8 – 17	1225.6800	1224.6727	1224.6727	-0.0000	0	K.GHERPLTFLR.Y
31 – 44	1658.7557	1657.7484	1657.7485	-0.0001	0	K.DHTPTLWFADNGER.L
50 – 62	1403.5943	1402.5870	1402.5871	-0.0000	0	R.GHNGAVWCCDVSR.D
67 – 77	1104.5895	1103.5822	1103.5822	-0.0000	0	R.LITGSADQTAK.L
107 – 118	1386.7375	1385.7302	1385.7303	-0.0001	0	R.LAVITTDHFVDR.T
127 – 148	2394.1129	2393.1056	2393.1057	-0.0000	0	R.IAEDPEEQDAESVLVLHCPDGK.K
149 – 150	304.0000	302.9927	302.2066	0.7861	1	K.KR.I
154 – 170	1770.9020	1769.8947	1769.8948	-0.0001	0	R.AVWGPLNQTIVSGGEDK.V
202 – 215	1476.6965	1475.6892	1475.6892	-0.0000	0	K.AADDSHFLTGSLDK.T
230 – 266	3789.9769	3788.9696	3788.9698	-0.0001	0	K.TYTTVVPVNAVSLSPLLNHVVLGGGQDASAVTTTDHR.A
288 – 303	1654.8336	1653.8263	1653.8263	-0.0000	0	K.GHFGPINALAFNPDGK.S
304 – 315	1260.5491	1259.5418	1259.5419	-0.0000	0	K.SFSSGGEDGYVR.L
316 – 327	1535.7277	1534.7204	1534.7205	-0.0001	0	R.LHHFDSDYFNIK.I

No match to: 107.0000, 127.0000, 154.0000, 202.0000, 230.0000, 288.0000, 316.0000

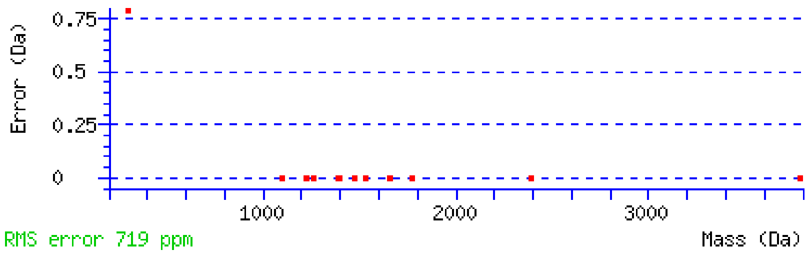

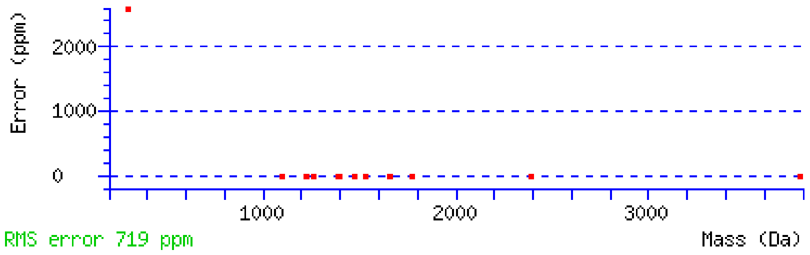

ID   EIF3I_ARATH   Reviewed;   328 AA.
AC   Q38884; O82342; Q94K09; Q9C5Z0;
DT   01-NOV-1997, integrated into UniProtKB/Swiss-Prot.
DT   19-SEP-2002, sequence version 2.
DT   31-JUL-2019, entry version 139.
DE   RecName: Full=Eukaryotic translation initiation factor 3 subunit I {ECO:0000255|HAMAP-Rule:MF_03008};
DE   Short=eIF3i {ECO:0000255|HAMAP-Rule:MF_03008};
DE   AltName: Full=Eukaryotic translation initiation factor 3 subunit 2 {ECO:0000255|HAMAP-Rule:MF_03008};
DE   AltName: Full=TGF-beta receptor-interacting protein 1;
DE   Short=TRIP-1;
DE   AltName: Full=eIF-3-beta {ECO:0000255|HAMAP-Rule:MF_03008};
DE   AltName: Full=eIF3 p36 {ECO:0000255|HAMAP-Rule:MF_03008};
GN   Name=TIF3I1; OrderedLocusNames=At2g46280; ORFNames=T3F17.7;
OS   Arabidopsis thaliana (Mouse-ear cress).
OC   Eukaryota; Viridiplantae; Streptophyta; Embryophyta; Tracheophyta;
OC   Spermatophyta; Magnoliopsida; eudicotyledons; Gunneridae;
OC   Pentapetalae; rosids; malvids; Brassicales; Brassicaceae; Camelineae;
OC   Arabidopsis.
OX   NCBI_TaxID=3702;
RN   [1]
RP   NUCLEOTIDE SEQUENCE [MRNA].
RX   PubMed=7566156; DOI=10.1038/377548a0;
RA   Chen R.H., Miettinen P.J., Maruka E.M., Choy L., Derynck R.;
RT   "A WD-domain protein that is associated with and phosphorylated by the
RT   type II TGF-beta receptor.";
RL   Nature 377:548-552(1995).
RN   [2]
RP   NUCLEOTIDE SEQUENCE [MRNA].
RX   PubMed=11042177; DOI=10.1074/jbc.M007236200;
RA   Burks E.A., Bezerra P.P., Le H., Gallie D.R., Browning K.S.;
RT   "Plant initiation factor 3 subunit composition resembles mammalian
RT   initiation factor 3 and has a novel subunit.";
RL   J. Biol. Chem. 276:2122-2131(2001).
RN   [3]
RP   NUCLEOTIDE SEQUENCE [LARGE SCALE GENOMIC DNA].
RC   STRAIN=cv. Columbia;
RX   PubMed=10617197; DOI=10.1038/45471;
RA   Lin X., Kaul S., Rounsley S.D., Shea T.P., Benito M.-I., Town C.D.,
RA   Fujii C.Y., Mason T.M., Bowman C.L., Barnstead M.E., Feldblyum T.V.,
RA   Buell C.R., Ketchum K.A., Lee J.J., Ronning C.M., Koo H.L.,
RA   Moffat K.S., Cronin L.A., Shen M., Pai G., Van Aken S., Umayam L.,
RA   Tallon L.J., Gill J.E., Adams M.D., Carrera A.J., Creasy T.H.,
RA   Goodman H.M., Somerville C.R., Copenhaver G.P., Preuss D.,
RA   Nierman W.C., White O., Eisen J.A., Salzberg S.L., Fraser C.M.,
RA   Venter J.C.;
RT   "Sequence and analysis of chromosome 2 of the plant Arabidopsis
RT   thaliana.";
RL   Nature 402:761-768(1999).
RN   [4]
RP   GENOME REANNOTATION.
RC   STRAIN=cv. Columbia;
RX   PubMed=27862469; DOI=10.1111/tpj.13415;
RA   Cheng C.Y., Krishnakumar V., Chan A.P., Thibaud-Nissen F., Schobel S.,
RA   Town C.D.;
RT   "Araport11: a complete reannotation of the Arabidopsis thaliana
RT   reference genome.";
RL   Plant J. 89:789-804(2017).
RN   [5]

```
RP   NUCLEOTIDE SEQUENCE [LARGE SCALE MRNA].
RC   STRAIN=cv. Columbia;
RX   PubMed=14593172; DOI=10.1126/science.1088305;
RA   Yamada K., Lim J., Dale J.M., Chen H., Shinn P., Palm C.J.,
RA   Southwick A.M., Wu H.C., Kim C.J., Nguyen M., Pham P.K., Cheuk R.F.,
RA   Karlin-Newmann G., Liu S.X., Lam B., Sakano H., Wu T., Yu G.,
RA   Miranda M., Quach H.L., Tripp M., Chang C.H., Lee J.M., Toriumi M.J.,
RA   Chan M.M., Tang C.C., Onodera C.S., Deng J.M., Akiyama K., Ansari Y.,
RA   Arakawa T., Banh J., Banno F., Bowser L., Brooks S.Y., Carninci P.,
RA   Chao Q., Choy N., Enju A., Goldsmith A.D., Gurjal M., Hansen N.F.,
RA   Hayashizaki Y., Johnson-Hopson C., Hsuan V.W., Iida K., Karnes M.,
RA   Khan S., Koesema E., Ishida J., Jiang P.X., Jones T., Kawai J.,
RA   Kamiya A., Meyers C., Nakajima M., Narusaka M., Seki M., Sakurai T.,
RA   Satou M., Tamse R., Vaysberg M., Wallender E.K., Wong C., Yamamura Y.,
RA   Yuan S., Shinozaki K., Davis R.W., Theologis A., Ecker J.R.;
RT   "Empirical analysis of transcriptional activity in the Arabidopsis
RT   genome.";
RL   Science 302:842-846(2003).
CC   -!- FUNCTION: Component of the eukaryotic translation initiation
CC       factor 3 (eIF-3) complex, which is involved in protein synthesis
CC       of a specialized repertoire of mRNAs and, together with other
CC       initiation factors, stimulates binding of mRNA and methionyl-tRNAi
CC       to the 40S ribosome. The eIF-3 complex specifically targets and
CC       initiates translation of a subset of mRNAs involved in cell
CC       proliferation. {ECO:0000255|HAMAP-Rule:MF_03008}.
CC   -!- SUBUNIT: Component of the eukaryotic translation initiation factor
CC       3 (eIF-3) complex. {ECO:0000255|HAMAP-Rule:MF_03008}.
CC   -!- SUBCELLULAR LOCATION: Cytoplasm {ECO:0000255|HAMAP-Rule:MF_03008}.
CC   -!- ALTERNATIVE PRODUCTS:
CC       Event=Alternative splicing; Named isoforms=1;
CC         Comment=A number of isoforms are produced. According to EST
CC         sequences.;
CC       Name=1;
CC         IsoId=Q38884-1; Sequence=Displayed;
CC   -!- SIMILARITY: Belongs to the eIF-3 subunit I family.
CC       {ECO:0000255|HAMAP-Rule:MF_03008}.
DR   EMBL; U36765; AAC49079.1; -; mRNA.
DR   EMBL; AF285835; AAG53616.1; -; mRNA.
DR   EMBL; AC005397; AAC62878.1; -; Genomic_DNA.
DR   EMBL; CP002685; AEC10670.1; -; Genomic_DNA.
DR   EMBL; CP002685; AEC10671.1; -; Genomic_DNA.
DR   EMBL; AF370485; AAK43862.1; -; mRNA.
DR   EMBL; AY064633; AAL47346.1; -; mRNA.
DR   PIR; H84900; H84900.
DR   PIR; S60256; S60256.
DR   RefSeq; NP_182151.1; NM_130191.6. [Q38884-1]
DR   RefSeq; NP_850450.1; NM_180119.3. [Q38884-1]
DR   BioGrid; 4571; 5.
DR   IntAct; Q38884; 2.
DR   STRING; 3702.AT2G46280.1; -.
DR   iPTMnet; Q38884; -.
DR   PaxDb; Q38884; -.
DR   PRIDE; Q38884; -.
DR   EnsemblPlants; AT2G46280.1; AT2G46280.1; AT2G46280. [Q38884-1]
DR   EnsemblPlants; AT2G46280.2; AT2G46280.2; AT2G46280. [Q38884-1]
DR   GeneID; 819236; -.
DR   Gramene; AT2G46280.1; AT2G46280.1; AT2G46280. [Q38884-1]
DR   Gramene; AT2G46280.2; AT2G46280.2; AT2G46280. [Q38884-1]
DR   KEGG; ath:AT2G46280; -.
DR   Araport; AT2G46280; -.
DR   TAIR; locus:2063009; AT2G46280.
DR   eggNOG; KOG0643; Eukaryota.
DR   eggNOG; ENOG410XQ3E; LUCA.
DR   HOGENOM; HOG000231322; -.
DR   InParanoid; Q38884; -.
DR   KO; K03246; -.
DR   OMA; FETRFWH; -.
DR   OrthoDB; 866359at2759; -.
DR   PhylomeDB; Q38884; -.
DR   PRO; PR:Q38884; -.
DR   Proteomes; UP000006548; Chromosome 2.
DR   ExpressionAtlas; Q38884; baseline and differential.
DR   Genevisible; Q38884; AT.
DR   GO; GO:0080008; C:Cul4-RING E3 ubiquitin ligase complex; IPI:TAIR.
DR   GO; GO:0005829; C:cytosol; IDA:TAIR.
DR   GO; GO:0016282; C:eukaryotic 43S preinitiation complex; IEA:UniProtKB-UniRule.
DR   GO; GO:0033290; C:eukaryotic 48S preinitiation complex; IEA:UniProtKB-UniRule.
DR   GO; GO:0005852; C:eukaryotic translation initiation factor 3 complex; IEA:UniProtKB-UniRule.
DR   GO; GO:0003743; F:translation initiation factor activity; IEA:UniProtKB-UniRule.
DR   GO; GO:0001732; P:formation of cytoplasmic translation initiation complex; IEA:UniProtKB-UniRule.
```

```
DR   GO; GO:0046686; P:response to cadmium ion; IEP:TAIR.
DR   GO; GO:0009651; P:response to salt stress; IEP:TAIR.
DR   Gene3D; 2.130.10.10; -; 1.
DR   HAMAP; MF_03008; eIF3i; 1.
DR   InterPro; IPR027525; eIF3i.
DR   InterPro; IPR020472; G-protein_beta_WD-40_rep.
DR   InterPro; IPR015943; WD40/YVTN_repeat-like_dom_sf.
DR   InterPro; IPR001680; WD40_repeat.
DR   InterPro; IPR019775; WD40_repeat_CS.
DR   InterPro; IPR017986; WD40_repeat_dom.
DR   InterPro; IPR036322; WD40_repeat_dom_sf.
DR   Pfam; PF00400; WD40; 4.
DR   PRINTS; PR00320; GPROTEINBRPT.
DR   SMART; SM00320; WD40; 5.
DR   SUPFAM; SSF50978; SSF50978; 1.
DR   PROSITE; PS00678; WD_REPEATS_1; 2.
DR   PROSITE; PS50082; WD_REPEATS_2; 5.
DR   PROSITE; PS50294; WD_REPEATS_REGION; 2.
PE   2: Evidence at transcript level;
KW   Alternative splicing; Complete proteome; Cytoplasm; Initiation factor;
KW   Protein biosynthesis; Reference proteome; Repeat; WD repeat.
FT   CHAIN         1    328       Eukaryotic translation initiation factor
FT                                3 subunit I.
FT                                /FTId=PRO_0000051039.
FT   REPEAT        8     49       WD 1.
FT   REPEAT       50     89       WD 2.
FT   REPEAT      146    185       WD 3.
FT   REPEAT      191    230       WD 4.
FT   REPEAT      288    327       WD 5.
FT   CONFLICT     21     23       EGD -> QPH (in Ref. 2; AAG53616).
FT                                {ECO:0000305}.
FT   CONFLICT    107    107       L -> F (in Ref. 2; AAG53616).
FT                                {ECO:0000305}.
FT   CONFLICT    126    128       RIA -> AYC (in Ref. 1; AAC49079).
FT                                {ECO:0000305}.
FT   CONFLICT    166    166       G -> C (in Ref. 1; AAC49079).
FT                                {ECO:0000305}.
FT   CONFLICT    255    255       Q -> E (in Ref. 1; AAC49079).
FT                                {ECO:0000305}.
FT   CONFLICT    275    275       Y -> C (in Ref. 1; AAC49079).
FT                                {ECO:0000305}.
FT   CONFLICT    304    304       S -> G (in Ref. 5; AAK43862/AAL47346).
FT                                {ECO:0000305}.
FT   CONFLICT    311    311       D -> G (in Ref. 1; AAC49079).
FT                                {ECO:0000305}.
SQ   SEQUENCE   328 AA;  36388 MW;  4ABFE9866A09CFAF CRC64;
     MRPILMKGHE RPLTFLRYNR EGDLLFSCAK DHTPTLWFAD NGERLGTYRG HNGAVWCCDV
     SRDSSRLITG SADQTAKLWD VKSGKELFTF KFNAPTRSVD FAVGDRLAVI TTDHFVDRTA
     AIHVKRIAED PEEQDAESVL VLHCPDGKKR INRAVWGPLN QTIVSGGEDK VIRIWDAETG
     KLLKQSDEEV GHKKDITSLC KAADDSHFLT GSLDKTAKLW DMRTLTLLKT YTTVVPVNAV
     SLSPLLNHVV LGGGQDASAV TTTDHRAGKF EAKFYDKILQ EEIGGVKGHF GPINALAFNP
     DGKSFSSGGE DGYVRLHHFD SDYFNIKI
```

MATRIX SCIENCE MASCOT Search Results

Protein View: AOC3_ARATH

Allene oxide cyclase 3, chloroplastic OS=Arabidopsis thaliana OX=3702 GN=AOC3 PE=2 SV=1

**Database:**	SwissProt
**Score:**	66
**Expect:**	0.011
**Monoisotopic mass (M_r):**	28380
**Calculated pI:**	9.19
**Taxonomy:**	Arabidopsis thaliana

Sequence similarity is available as an NCBI BLAST search of AOC3_ARATH against nr.

Search parameters

**Enzyme:**	Trypsin: cuts C-term side of KR unless next residue is P.
**Mass values searched:**	9
**Mass values matched:**	5

Protein sequence coverage: 43%

Matched peptides shown in ***bold red***.

1 MASSSAAMSL ESISMTTLNN LSRNHQSHRS SLLGFSRSFQ NLGISSNGPD
51 FSSRSRSTTS KNLNVTRAFF WNWGKKTENS RPSK**IQELNV** **YELNEGDR**NS
101 PAVLKLGK**KP** **TELCLGDLVP** **FTNK**LYTGDL KKR**VGITAGL** **CVLIQHVPEK**
151 SGDR**FEASYS** **FYFGDYGHLS** **VQGQYLTYED** **TFLAVTGGSG** **IFEGAYGQVK**
201 LRQLVYPTKL FYTFYLK**GLA** **NDLPLELTGT** **AVTPSK**DVKP APEAKAMEPS
251 GVISNFTN

Unformatted sequence string: 258 residues (for pasting into other applications).

Sort by ◉ residue number ○ increasing mass ○ decreasing mass
Show ◉ matched peptides only ○ predicted peptides also

Start – End	Observed	Mr(expt)	Mr(calc)	Delta	M	Peptide
85 – 98	1691.8235	1690.8162	1690.8162	0.0000	0	K.IQELNVYELNEGDR.N
109 – 124	1774.9407	1773.9334	1773.9335	-0.0001	0	K.KPTELCLGDLVPFTNK.L
134 – 150	1777.0040	1775.9967	1775.9968	-0.0001	0	R.VGITAGLCVLIQHVPEK.S
155 – 200	5069.3468	5068.3395	5068.3396	-0.0001	0	R.FEASYSFYFGDYGHLSVQGQYLTYEDTFLAVTGGSGIFEGAYGQVK.L
218 – 236	1897.0276	1896.0203	1896.0204	-0.0001	0	K.GLANDLPLELTGTAVTPSK.D

No match to: 109.0000, 134.0000, 155.0000, 218.0000

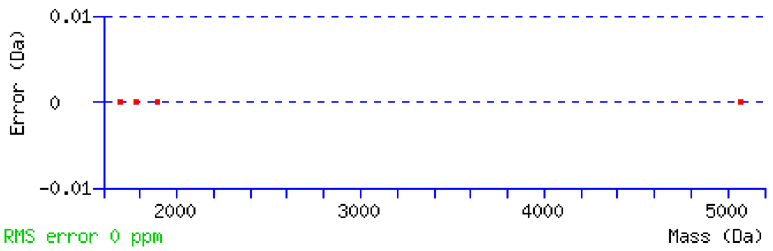

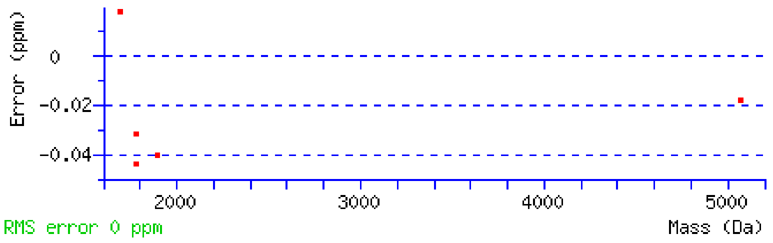


```
ID   AOC3_ARATH              Reviewed;         258 AA.
AC   Q9LS01;
DT   30-AUG-2005, integrated into UniProtKB/Swiss-Prot.
DT   01-OCT-2000, sequence version 1.
DT   31-JUL-2019, entry version 104.
DE   RecName: Full=Allene oxide cyclase 3, chloroplastic;
```

```
DE            EC=5.3.99.6;
DE   Flags: Precursor;
GN   Name=AOC3; OrderedLocusNames=At3g25780; ORFNames=K13N2.12, K13N2_10;
OS   Arabidopsis thaliana (Mouse-ear cress).
OC   Eukaryota; Viridiplantae; Streptophyta; Embryophyta; Tracheophyta;
OC   Spermatophyta; Magnoliopsida; eudicotyledons; Gunneridae;
OC   Pentapetalae; rosids; malvids; Brassicales; Brassicaceae; Camelineae;
OC   Arabidopsis.
OX   NCBI_TaxID=3702;
RN   [1]
RP   NUCLEOTIDE SEQUENCE [MRNA], TISSUE SPECIFICITY, SUBCELLULAR LOCATION,
RP   AND INDUCTION.
RC   STRAIN=cv. Columbia; TISSUE=Leaf;
RX   PubMed=12777050; DOI=10.1023/A:1023049319723;
RA   Stenzel I., Hause B., Miersch O., Kurz T., Maucher H., Weichart H.,
RA   Ziegler J., Feussner I., Wasternack C.;
RT   "Jasmonate biosynthesis and the allene oxide cyclase family of
RT   Arabidopsis thaliana.";
RL   Plant Mol. Biol. 51:895-911(2003).
RN   [2]
RP   NUCLEOTIDE SEQUENCE [LARGE SCALE GENOMIC DNA].
RC   STRAIN=cv. Columbia;
RX   PubMed=10819329; DOI=10.1093/dnares/7.2.131;
RA   Sato S., Nakamura Y., Kaneko T., Katoh T., Asamizu E., Tabata S.;
RT   "Structural analysis of Arabidopsis thaliana chromosome 3. I. Sequence
RT   features of the regions of 4,504,864 bp covered by sixty P1 and TAC
RT   clones.";
RL   DNA Res. 7:131-135(2000).
RN   [3]
RP   GENOME REANNOTATION.
RC   STRAIN=cv. Columbia;
RX   PubMed=27862469; DOI=10.1111/tpj.13415;
RA   Cheng C.Y., Krishnakumar V., Chan A.P., Thibaud-Nissen F., Schobel S.,
RA   Town C.D.;
RT   "Araport11: a complete reannotation of the Arabidopsis thaliana
RT   reference genome.";
RL   Plant J. 89:789-804(2017).
RN   [4]
RP   NUCLEOTIDE SEQUENCE [LARGE SCALE MRNA].
RA   Brover V.V., Troukhan M.E., Alexandrov N.A., Lu Y.-P., Flavell R.B.,
RA   Feldmann K.A.;
RT   "Full-length cDNA from Arabidopsis thaliana.";
RL   Submitted (MAR-2002) to the EMBL/GenBank/DDBJ databases.
CC   -!- FUNCTION: Involved in the production of 12-oxo-phytodienoic acid
CC       (OPDA), a precursor of jasmonic acid.
CC   -!- CATALYTIC ACTIVITY:
CC       Reaction=(9Z,13S,15Z)-12,13-epoxyoctadeca-9,11,15-trienoate =
CC         (10Z,15Z)-12-oxophytodienoate; Xref=Rhea:RHEA:22592,
CC         ChEBI:CHEBI:36438, ChEBI:CHEBI:57411; EC=5.3.99.6;
CC   -!- SUBCELLULAR LOCATION: Plastid, chloroplast
CC       {ECO:0000269|PubMed:12777050}.
CC   -!- TISSUE SPECIFICITY: Highly expressed in fully developed leaves.
CC       {ECO:0000269|PubMed:12777050}.
CC   -!- INDUCTION: Low local and systemic induction by wounding.
CC       {ECO:0000269|PubMed:12777050}.
CC   -!- MISCELLANEOUS: The four allene oxide cyclase proteins (AOC1, AOC2,
CC       AOC3 and AOC4) are encoded by duplicated genes. They are very
CC       similar, and most experiments involving antibodies do not
CC       discriminate between the different members.
CC   -!- SIMILARITY: Belongs to the allene oxide cyclase family.
CC       {ECO:0000305}.
DR   EMBL; AJ308485; CAC83763.1; -; mRNA.
DR   EMBL; AB028607; BAA95765.1; -; Genomic_DNA.
DR   EMBL; CP002686; AEE77067.1; -; Genomic_DNA.
DR   EMBL; AY087359; AAM64909.1; -; mRNA.
DR   RefSeq; NP_566777.1; NM_113477.5.
DR   SMR; Q9LS01; -.
DR   STRING; 3702.AT3G25780.1; -.
DR   SwissPalm; Q9LS01; -.
DR   PaxDb; Q9LS01; -.
DR   PRIDE; Q9LS01; -.
DR   EnsemblPlants; AT3G25780.1; AT3G25780.1; AT3G25780.
DR   GeneID; 822169; -.
DR   Gramene; AT3G25780.1; AT3G25780.1; AT3G25780.
DR   KEGG; ath:AT3G25780; -.
DR   Araport; AT3G25780; -.
DR   TAIR; locus:2085974; AT3G25780.
DR   eggNOG; ENOG410IHTA; Eukaryota.
DR   eggNOG; ENOG4111X07; LUCA.
DR   HOGENOM; HOG000240167; -.
DR   InParanoid; Q9LS01; -.
DR   KO; K10525; -.
DR   OMA; KPTELCL; -.
DR   OrthoDB; 1284108at2759; -.
```

```
DR   PhylomeDB; Q9LS01; -.
DR   BioCyc; ARA:AT3G25780-MONOMER; -.
DR   BRENDA; 5.3.99.6; 399.
DR   PRO; PR:Q9LS01; -.
DR   Proteomes; UP000006548; Chromosome 3.
DR   ExpressionAtlas; Q9LS01; baseline and differential.
DR   Genevisible; Q9LS01; AT.
DR   GO; GO:0009507; C:chloroplast; IEA:UniProtKB-SubCell.
DR   GO; GO:0016020; C:membrane; IDA:TAIR.
DR   GO; GO:0005886; C:plasma membrane; IDA:TAIR.
DR   GO; GO:0005774; C:vacuolar membrane; IDA:TAIR.
DR   GO; GO:0046423; F:allene-oxide cyclase activity; ISS:TAIR.
DR   GO; GO:0009695; P:jasmonic acid biosynthetic process; TAS:TAIR.
DR   GO; GO:0009620; P:response to fungus; IEP:TAIR.
DR   GO; GO:0009651; P:response to salt stress; IEP:TAIR.
DR   Gene3D; 2.40.480.10; -; 1.
DR   InterPro; IPR009410; Allene_ox_cyc.
DR   InterPro; IPR034871; Allene_oxi_cyc_sf.
DR   PANTHER; PTHR31843; PTHR31843; 1.
DR   Pfam; PF06351; Allene_ox_cyc; 1.
DR   SUPFAM; SSF141493; SSF141493; 1.
PE   2: Evidence at transcript level;
KW   Chloroplast; Complete proteome; Isomerase; Plastid;
KW   Reference proteome; Transit peptide.
FT   TRANSIT       1     56       Chloroplast. {ECO:0000255}.
FT   CHAIN        57    258       Allene oxide cyclase 3, chloroplastic.
FT                                /FTId=PRO_0000001704.
SQ   SEQUENCE   258 AA;  28398 MW;  42AF942E0E9AAB87 CRC64;
     MASSSAAMSL ESISMTTLNN LSRNHQSHRS SLLGFSRSFQ NLGISSNGPD FSSRSRSTTS
     KNLNVTRAFF WNWGKKTENS RPSKIQELNV YELNEGDRNS PAVLKLGKKP TELCLGDLVP
     FTNKLYTGDL KKRVGITAGL CVLIQHVPEK SGDRFEASYS FYFGDYGHLS VQGQYLTYED
     TFLAVTGGSG IFEGAYGQVK LRQLVYPTKL FYTFYLKGLA NDLPLELTGT AVTPSKDVKP
     APEAKAMEPS GVISNFTN
```

Mascot: http://www.matrixscience.com/

MATRIX SCIENCE **MASCOT Search Results**

Protein View: DUT_ARATH

Deoxyuridine 5'-triphosphate nucleotidohydrolase OS=Arabidopsis thaliana OX=3702 GN=DUT PE=1 SV=1

**Database:**	SwissProt
**Score:**	70
**Expect:**	0.0036
**Monoisotopic mass (M_r):**	17546
**Calculated pI:**	5.34
**Taxonomy:**	Arabidopsis thaliana

Sequence similarity is available as an NCBI BLAST search of DUT_ARATH against nr.

Search parameters

**Enzyme:**	Trypsin: cuts C-term side of KR unless next residue is P.
**Mass values searched:**	7
**Mass values matched:**	5

Protein sequence coverage: 51%

Matched peptides shown in ***bold red***.

```
  1 MACVNEPSPK LQKLDRNGIH GDSSPSPFFK VKKLSEKAVI PTRGSPLSAG
 51 YDLSSAVDSK VPARGKALIP TDLSIAVPEG TYARIAPRSG LAWKHSIDVG
101 AGVIDADYRG PVGVILFNHS DADFEVKFGD RIAQLIIEKI VTPDVVEVDD
151 LDETVRGDGG FGSTGV
```

Unformatted sequence string: 166 residues (for pasting into other applications).

Sort by ◉ residue number ○ increasing mass ○ decreasing mass
Show ◉ matched peptides only ○ predicted peptides also

Start – End	Observed	Mr(expt)	Mr(calc)	Delta	M	Peptide
44 – 60	1653.7966	1652.7893	1652.7893	-0.0000	0	R.GSPLSAGYDLSSAVDSK.V
67 – 84	1887.0221	1886.0148	1886.0149	-0.0001	0	K.ALIPTDLSIAVPEGTYAR.I
95 – 109	1587.7761	1586.7688	1586.7689	-0.0001	0	K.HSIDVGAGVIDADYR.G
110 – 127	1943.9861	1942.9788	1942.9789	-0.0001	0	R.GPVGVILFNHSDADFEVK.F
140 – 156	1913.9702	1912.9629	1912.9630	-0.0001	0	K.IVTPDVVEVDDLDETVR.G

No match to: 110.0000, 140.0000

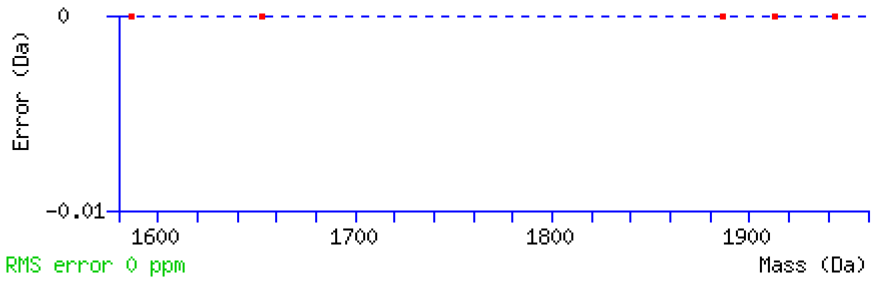

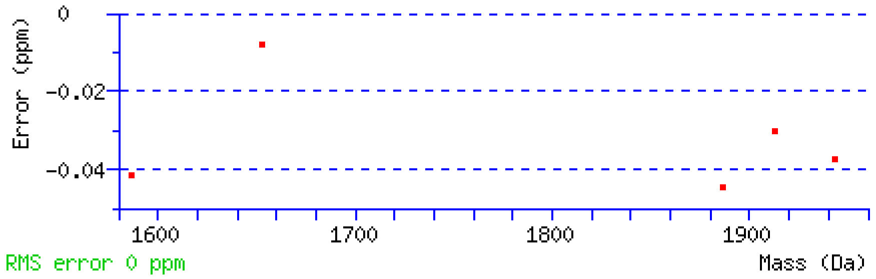

```
ID   DUT_ARATH               Reviewed;         166 AA.
AC   Q9STG6;
DT   30-NOV-2010, integrated into UniProtKB/Swiss-Prot.
DT   01-MAY-2000, sequence version 1.
DT   31-JUL-2019, entry version 123.
DE   RecName: Full=Deoxyuridine 5'-triphosphate nucleotidohydrolase;
DE            Short=dUTPase;
DE            EC=3.6.1.23;
DE   AltName: Full=dUTP pyrophosphatase;
DE   AltName: Full=dUTP-pyrophosphatase-like 1;
DE            Short=AtDUT1;
GN   Name=DUT; Synonyms=DUT1; OrderedLocusNames=At3g46940;
GN   ORFNames=T6H20.30;
OS   Arabidopsis thaliana (Mouse-ear cress).
OC   Eukaryota; Viridiplantae; Streptophyta; Embryophyta; Tracheophyta;
OC   Spermatophyta; Magnoliopsida; eudicotyledons; Gunneridae;
OC   Pentapetalae; rosids; malvids; Brassicales; Brassicaceae; Camelineae;
OC   Arabidopsis.
OX   NCBI_TaxID=3702;
RN   [1]
RP   NUCLEOTIDE SEQUENCE [LARGE SCALE GENOMIC DNA].
RC   STRAIN=cv. Columbia;
RX   PubMed=11130713; DOI=10.1038/35048706;
RA   Salanoubat M., Lemcke K., Rieger M., Ansorge W., Unseld M.,
RA   Fartmann B., Valle G., Bloecker H., Perez-Alonso M., Obermaier B.,
RA   Delseny M., Boutry M., Grivell L.A., Mache R., Puigdomenech P.,
RA   De Simone V., Choisne N., Artiguenave F., Robert C., Brottier P.,
RA   Wincker P., Cattolico L., Weissenbach J., Saurin W., Quetier F.,
RA   Schaefer M., Mueller-Auer S., Gabel C., Fuchs M., Benes V.,
RA   Wurmbach E., Drzonek H., Erfle H., Jordan N., Bangert S.,
RA   Wiedelmann R., Kranz H., Voss H., Holland R., Brandt P., Nyakatura G.,
RA   Vezzi A., D'Angelo M., Pallavicini A., Toppo S., Simionati B.,
RA   Conrad A., Hornischer K., Kauer G., Loehnert T.-H., Nordsiek G.,
RA   Reichelt J., Scharfe M., Schoen O., Bargues M., Terol J., Climent J.,
RA   Navarro P., Collado C., Perez-Perez A., Ottenwaelder B., Duchemin D.,
RA   Cooke R., Laudie M., Berger-Llauro C., Purnelle B., Masuy D.,
RA   de Haan M., Maarse A.C., Alcaraz J.-P., Cottet A., Casacuberta E.,
RA   Monfort A., Argiriou A., Flores M., Liguori R., Vitale D.,
RA   Mannhaupt G., Haase D., Schoof H., Rudd S., Zaccaria P., Mewes H.-W.,
RA   Mayer K.F.X., Kaul S., Town C.D., Koo H.L., Tallon L.J., Jenkins J.,
RA   Rooney T., Rizzo M., Walts A., Utterback T., Fujii C.Y., Shea T.P.,
RA   Creasy T.H., Haas B., Maiti R., Wu D., Peterson J., Van Aken S.,
RA   Pai G., Militscher J., Sellers P., Gill J.E., Feldblyum T.V.,
RA   Preuss D., Lin X., Nierman W.C., Salzberg S.L., White O., Venter J.C.,
RA   Fraser C.M., Kaneko T., Nakamura Y., Sato S., Kato T., Asamizu E.,
RA   Sasamoto S., Kimura T., Idesawa K., Kawashima K., Kishida Y.,
RA   Kiyokawa C., Kohara M., Matsumoto M., Matsuno A., Muraki A.,
RA   Nakayama S., Nakazaki N., Shinpo S., Takeuchi C., Wada T.,
RA   Watanabe A., Yamada M., Yasuda M., Tabata S.;
RT   "Sequence and analysis of chromosome 3 of the plant Arabidopsis
RT   thaliana.";
RL   Nature 408:820-822(2000).
```

```
RN   [2]
RP   GENOME REANNOTATION.
RC   STRAIN=cv. Columbia;
RX   PubMed=27862469; DOI=10.1111/tpj.13415;
RA   Cheng C.Y., Krishnakumar V., Chan A.P., Thibaud-Nissen F., Schobel S.,
RA   Town C.D.;
RT   "Araport11: a complete reannotation of the Arabidopsis thaliana
RT   reference genome.";
RL   Plant J. 89:789-804(2017).
RN   [3]
RP   NUCLEOTIDE SEQUENCE [LARGE SCALE MRNA].
RC   STRAIN=cv. Columbia;
RX   PubMed=14593172; DOI=10.1126/science.1088305;
RA   Yamada K., Lim J., Dale J.M., Chen H., Shinn P., Palm C.J.,
RA   Southwick A.M., Wu H.C., Kim C.J., Nguyen M., Pham P.K., Cheuk R.F.,
RA   Karlin-Newmann G., Liu S.X., Lam B., Sakano H., Wu T., Yu G.,
RA   Miranda M., Quach H.L., Tripp M., Chang C.H., Lee J.M., Toriumi M.J.,
RA   Chan M.M., Tang C.C., Onodera C.S., Deng J.M., Akiyama K., Ansari Y.,
RA   Arakawa T., Banh J., Banno F., Bowser L., Brooks S.Y., Carninci P.,
RA   Chao Q., Choy N., Enju A., Goldsmith A.D., Gurjal M., Hansen N.F.,
RA   Hayashizaki Y., Johnson-Hopson C., Hsuan V.W., Iida K., Karnes M.,
RA   Khan S., Koesema E., Ishida J., Jiang P.X., Jones T., Kawai J.,
RA   Kamiya A., Meyers C., Nakajima M., Narusaka M., Seki M., Sakurai T.,
RA   Satou M., Tamse R., Vaysberg M., Wallender E.K., Wong C., Yamamura Y.,
RA   Yuan S., Shinozaki K., Davis R.W., Theologis A., Ecker J.R.;
RT   "Empirical analysis of transcriptional activity in the Arabidopsis
RT   genome.";
RL   Science 302:842-846(2003).
RN   [4]
RP   FUNCTION.
RX   PubMed=20227352; DOI=10.1016/j.dnarep.2010.02.009;
RA   Siaud N., Dubois E., Massot S., Richaud A., Dray E., Collier J.,
RA   Doutriaux M.P.;
RT   "The SOS screen in Arabidopsis: a search for functions involved in DNA
RT   metabolism.";
RL   DNA Repair 9:567-578(2010).
RN   [5]
RP   X-RAY CRYSTALLOGRAPHY (2.2 ANGSTROMS) IN COMPLEX WITH MAGNESIUM, AND
RP   SUBUNIT.
RX   PubMed=17565183; DOI=10.1107/S1744309107016004;
RA   Bajaj M., Moriyama H.;
RT   "Purification, crystallization and preliminary crystallographic
RT   analysis of deoxyuridine triphosphate nucleotidohydrolase from
RT   Arabidopsis thaliana.";
RL   Acta Crystallogr. F 63:409-411(2007).
RN   [6]
RP   X-RAY CRYSTALLOGRAPHY (2.00 ANGSTROMS).
RA   Bajaj M., Moriyama H.;
RT   "Structure of dutpase from Arabidopsis thaliana.";
RL   Submitted (FEB-2009) to the PDB data bank.
CC   -!- FUNCTION: This enzyme is involved in nucleotide metabolism: it
CC       produces dUMP, the immediate precursor of thymidine nucleotides
CC       and it decreases the intracellular concentration of dUTP,
CC       preventing uracil incorporation into DNA.
CC       {ECO:0000269|PubMed:20227352}.
CC   -!- CATALYTIC ACTIVITY:
CC       Reaction=dUTP + H2O = diphosphate + dUMP + H(+);
CC         Xref=Rhea:RHEA:10248, ChEBI:CHEBI:15377, ChEBI:CHEBI:15378,
CC         ChEBI:CHEBI:33019, ChEBI:CHEBI:61555, ChEBI:CHEBI:246422;
CC         EC=3.6.1.23;
CC   -!- COFACTOR:
CC       Name=Mg(2+); Xref=ChEBI:CHEBI:18420;
CC       Note=Binds 1 Mg(2+) per trimer.;
CC   -!- PATHWAY: Pyrimidine metabolism; dUMP biosynthesis; dUMP from dCTP
CC       (dUTP route): step 2/2.
CC   -!- SUBUNIT: Homotrimer. {ECO:0000269|PubMed:17565183}.
CC   -!- MISCELLANEOUS: Silencing of DUT leads to high seedling mortality
CC       and affects plant growth and flower organ morphology in surviving
CC       plants.
CC   -!- SIMILARITY: Belongs to the dUTPase family. {ECO:0000305}.
DR   EMBL; AL096859; CAB51171.1; -; Genomic_DNA.
DR   EMBL; CP002686; AEE78222.1; -; Genomic_DNA.
DR   EMBL; AF370334; AAK44149.1; -; mRNA.
DR   EMBL; AY062989; AAL34163.1; -; mRNA.
```

```
DR   PIR; T12954; T12954.
DR   RefSeq; NP_190278.1; NM_114561.4.
DR   PDB; 2PC5; X-ray; 2.20 A; A/B/C=1-166.
DR   PDB; 4OOP; X-ray; 1.50 A; A/B/C=1-166.
DR   PDB; 4OOQ; X-ray; 2.00 A; A/B/C=1-166.
DR   PDBsum; 2PC5; -.
DR   PDBsum; 4OOP; -.
DR   PDBsum; 4OOQ; -.
DR   SMR; Q9STG6; -.
DR   BioGrid; 9167; 1.
DR   STRING; 3702.AT3G46940.1; -.
DR   iPTMnet; Q9STG6; -.
DR   PaxDb; Q9STG6; -.
DR   PRIDE; Q9STG6; -.
DR   EnsemblPlants; AT3G46940.1; AT3G46940.1; AT3G46940.
DR   GeneID; 823847; -.
DR   Gramene; AT3G46940.1; AT3G46940.1; AT3G46940.
DR   KEGG; ath:AT3G46940; -.
DR   Araport; AT3G46940; -.
DR   TAIR; locus:2102817; AT3G46940.
DR   eggNOG; KOG3370; Eukaryota.
DR   eggNOG; COG0756; LUCA.
DR   HOGENOM; HOG000028966; -.
DR   KO; K01520; -.
DR   OrthoDB; 1336556at2759; -.
DR   PhylomeDB; Q9STG6; -.
DR   BioCyc; ARA:AT3G46940-MONOMER; -.
DR   BioCyc; MetaCyc:AT3G46940-MONOMER; -.
DR   BRENDA; 3.6.1.23; 399.
DR   UniPathway; UPA00610; UER00666.
DR   EvolutionaryTrace; Q9STG6; -.
DR   PRO; PR:Q9STG6; -.
DR   Proteomes; UP000006548; Chromosome 3.
DR   ExpressionAtlas; Q9STG6; baseline and differential.
DR   Genevisible; Q9STG6; AT.
DR   GO; GO:0005829; C:cytosol; IDA:TAIR.
DR   GO; GO:0004170; F:dUTP diphosphatase activity; IBA:GO_Central.
DR   GO; GO:0042802; F:identical protein binding; IPI:UniProtKB.
DR   GO; GO:0000287; F:magnesium ion binding; IDA:UniProtKB.
DR   GO; GO:0006281; P:DNA repair; IMP:TAIR.
DR   GO; GO:0006226; P:dUMP biosynthetic process; IBA:GO_Central.
DR   GO; GO:0046081; P:dUTP catabolic process; IBA:GO_Central.
DR   CDD; cd07557; trimeric_dUTPase; 1.
DR   Gene3D; 2.70.40.10; -; 1.
DR   InterPro; IPR029054; dUTPase-like.
DR   InterPro; IPR036157; dUTPase-like_sf.
DR   InterPro; IPR008181; dUTPase_1.
DR   InterPro; IPR033704; dUTPase_trimeric.
DR   PANTHER; PTHR11241; PTHR11241; 1.
DR   Pfam; PF00692; dUTPase; 1.
DR   SUPFAM; SSF51283; SSF51283; 1.
DR   TIGRFAMs; TIGR00576; dut; 1.
PE   1: Evidence at protein level;
KW   3D-structure; Complete proteome; Hydrolase; Magnesium; Metal-binding;
KW   Nucleotide metabolism; Reference proteome.
FT   CHAIN         1    166       Deoxyuridine 5'-triphosphate
FT                                nucleotidohydrolase.
FT                                /FTId=PRO_0000401366.
FT   METAL       138    138       Magnesium; shared with trimeric partners.
FT                                {ECO:0000269|PubMed:17565183}.
FT   STRAND       30     35       {ECO:0000244|PDB:4OOP}.
FT   STRAND       42     45       {ECO:0000244|PDB:4OOP}.
FT   STRAND       51     54       {ECO:0000244|PDB:4OOP}.
FT   STRAND       59     61       {ECO:0000244|PDB:4OOP}.
FT   STRAND       66     70       {ECO:0000244|PDB:4OOP}.
FT   STRAND       73     76       {ECO:0000244|PDB:4OOP}.
FT   STRAND       81     86       {ECO:0000244|PDB:4OOP}.
FT   HELIX        89     95       {ECO:0000244|PDB:4OOP}.
FT   STRAND       97    100       {ECO:0000244|PDB:4OOP}.
FT   STRAND      103    105       {ECO:0000244|PDB:2PC5}.
FT   STRAND      113    118       {ECO:0000244|PDB:4OOP}.
FT   STRAND      120    122       {ECO:0000244|PDB:4OOP}.
FT   STRAND      124    126       {ECO:0000244|PDB:4OOP}.
FT   STRAND      131    141       {ECO:0000244|PDB:4OOP}.
```

```
FT   STRAND       145    147       {ECO:0000244|PDB:4OOP}.
SQ   SEQUENCE   166 AA;  17557 MW;  5487738DF4A55BFF CRC64;
     MACVNEPSPK LQKLDRNGIH GDSSPSPFFK VKKLSEKAVI PTRGSPLSAG YDLSSAVDSK
     VPARGKALIP TDLSIAVPEG TYARIAPRSG LAWKHSIDVG AGVIDADYRG PVGVILFNHS
     DADFEVKFGD RIAQLIIEKI VTPDVVEVDD LDETVRGDGG FGSTGV
```

Mascot: http://www.matrixscience.com/

MATRIX SCIENCE MASCOT Search Results

Protein View: RAN1_ORYSI

GTP-binding nuclear protein Ran-1 OS=Oryza sativa subsp. indica OX=39946 GN=RAN1 PE=2 SV=2

**Database:**	SwissProt
**Score:**	120
**Expect:**	4e-08
**Monoisotopic mass (M_r):**	24987
**Calculated pI:**	6.38
**Taxonomy:**	Oryza sativa Indica Group

Sequence similarity is available as an NCBI BLAST search of RAN1_ORYSI against nr.

Search parameters

**Enzyme:**	Trypsin: cuts C-term side of KR unless next residue is P.
**Mass values searched:**	25
**Mass values matched:**	14

Protein sequence coverage: 56%

Matched peptides shown in ***bold red***.

```
  1 MALPNQQTVD YPSFKLVIVG DGGTGKTTFV KRHLTGEFEK KYEPTIGVEV
 51 HPLDFFTNCG KIRFYCWDTA GQEKFGGLRD GYYIHGQCAI IMFDVTSRLT
101 YKNVPTWHRD LCRVCENIPI VLCGNKVDVK NRQVKAKQVT FHRKKNLQYY
151 EVSAKSNYNF EKPFLYLARK LAGDGNLHFV ETPALAPPDV TIDLAAQQQH
201 EAELAAAAAQ PLPDDDDDLI E
```

Unformatted sequence string: 221 residues (for pasting into other applications).

Sort by ◉ residue number ○ increasing mass ○ decreasing mass
Show ◉ matched peptides only ○ predicted peptides also

Start - End	Observed	Mr(expt)	Mr(calc)	Delta	M	Peptide
1 - 15	1738.8469	1737.8396	1737.8396	0.0000	0	-.MALPNQQTVDYPSFK.L
16 - 26	1015.5782	1014.5709	1014.5710	-0.0001	0	K.LVIVGDGGTGK.T
27 - 31	595.3450	594.3377	594.3377	0.0000	0	K.TTFVK.R
33 - 40	960.4785	959.4712	959.4712	-0.0000	0	R.HLTGEFEK.K
42 - 61	2266.0848	2265.0775	2265.0776	-0.0001	0	K.YEPTIGVEVHPLDFFTNCGK.I
64 - 74	1347.5674	1346.5601	1346.5601	-0.0000	0	R.FYCWDTAGQEK.F
75 - 79	549.3143	548.3070	548.3071	-0.0001	0	K.FGGLR.D
99 - 102	524.3079	523.3006	523.3006	0.0000	0	R.LTYK.N
103 - 109	909.4689	908.4616	908.4617	-0.0001	0	K.NVPTWHR.D
110 - 113	506.2391	505.2318	505.2319	-0.0000	0	R.DLCR.V
114 - 126	1401.7228	1400.7155	1400.7156	-0.0001	0	R.VCENIPIVLCGNK.V
138 - 143	787.4209	786.4136	786.4137	-0.0001	0	K.QVTFHR.K
156 - 169	1761.8958	1760.8885	1760.8886	-0.0000	0	K.SNYNFEKPFLYLAR.K
170 - 170	146.0000	144.9927	146.1055	-1.1128	0	R.K.L

No match to: 103.0000, 110.0000, 114.0000, 138.0000, 156.0000, 171.0000, 200.0000, 1228.6208, 2173.0205, 2429.0990, 3166.6167

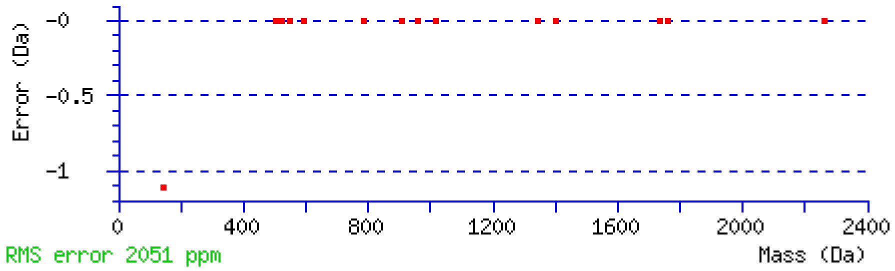

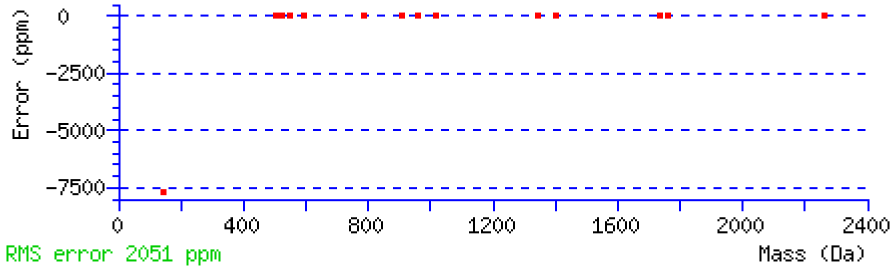

```
ID   RAN1_ORYSI              Reviewed;         221 AA.
AC   A2WSI7;
DT   02-SEP-2008, integrated into UniProtKB/Swiss-Prot.
DT   02-SEP-2008, sequence version 2.
DT   31-JUL-2019, entry version 71.
DE   RecName: Full=GTP-binding nuclear protein Ran-1;
DE            Short=OsRan1;
DE   AltName: Full=Ras-related nuclear protein 1;
GN   Name=RAN1; ORFNames=OsI_002780;
OS   Oryza sativa subsp. indica (Rice).
OC   Eukaryota; Viridiplantae; Streptophyta; Embryophyta; Tracheophyta;
OC   Spermatophyta; Magnoliopsida; Liliopsida; Poales; Poaceae; BOP clade;
OC   Oryzoideae; Oryzeae; Oryzinae; Oryza; Oryza sativa.
OX   NCBI_TaxID=39946;
RN   [1]
RP   NUCLEOTIDE SEQUENCE [LARGE SCALE GENOMIC DNA].
RC   STRAIN=cv. 93-11;
RX   PubMed=15685292; DOI=10.1371/journal.pbio.0030038;
RA   Yu J., Wang J., Lin W., Li S., Li H., Zhou J., Ni P., Dong W., Hu S.,
RA   Zeng C., Zhang J., Zhang Y., Li R., Xu Z., Li S., Li X., Zheng H.,
RA   Cong L., Lin L., Yin J., Geng J., Li G., Shi J., Liu J., Lv H., Li J.,
RA   Wang J., Deng Y., Ran L., Shi X., Wang X., Wu Q., Li C., Ren X.,
RA   Wang J., Wang X., Li D., Liu D., Zhang X., Ji Z., Zhao W., Sun Y.,
RA   Zhang Z., Bao J., Han Y., Dong L., Ji J., Chen P., Wu S., Liu J.,
RA   Xiao Y., Bu D., Tan J., Yang L., Ye C., Zhang J., Xu J., Zhou Y.,
RA   Yu Y., Zhang B., Zhuang S., Wei H., Liu B., Lei M., Yu H., Li Y.,
RA   Xu H., Wei S., He X., Fang L., Zhang Z., Zhang Y., Huang X., Su Z.,
RA   Tong W., Li J., Tong Z., Li S., Ye J., Wang L., Fang L., Lei T.,
RA   Chen C.-S., Chen H.-C., Xu Z., Li H., Huang H., Zhang F., Xu H.,
RA   Li N., Zhao C., Li S., Dong L., Huang Y., Li L., Xi Y., Qi Q., Li W.,
RA   Zhang B., Hu W., Zhang Y., Tian X., Jiao Y., Liang X., Jin J., Gao L.,
RA   Zheng W., Hao B., Liu S.-M., Wang W., Yuan L., Cao M., McDermott J.,
RA   Samudrala R., Wang J., Wong G.K.-S., Yang H.;
RT   "The genomes of Oryza sativa: a history of duplications.";
RL   PLoS Biol. 3:266-281(2005).
RN   [2]
RP   NUCLEOTIDE SEQUENCE [LARGE SCALE MRNA].
RC   STRAIN=cv. Guang-Lu-Ai No.4;
RX   PubMed=17522955; DOI=10.1007/s11103-007-9174-7;
RA   Liu X., Lu T., Yu S., Li Y., Huang Y., Huang T., Zhang L., Zhu J.,
RA   Zhao Q., Fan D., Mu J., Shangguan Y., Feng Q., Guan J., Ying K.,
RA   Zhang Y., Lin Z., Sun Z., Qian Q., Lu Y., Han B.;
RT   "A collection of 10,096 indica rice full-length cDNAs reveals highly
RT   expressed sequence divergence between Oryza sativa indica and japonica
RT   subspecies.";
RL   Plant Mol. Biol. 65:403-415(2007).
CC   -!- FUNCTION: GTP-binding protein involved in nucleocytoplasmic
CC       transport. Required for the import of protein into the nucleus and
CC       also for RNA export. Involved in chromatin condensation and
CC       control of cell cycle (By similarity). {ECO:0000250}.
CC   -!- SUBUNIT: Found in a nuclear export complex with RanGTP, exportin
CC       and pre-miRNA (By similarity). {ECO:0000250|UniProtKB:P62825}.
```

```
CC   -!- SUBCELLULAR LOCATION: Nucleus {ECO:0000250}.
CC   -!- SIMILARITY: Belongs to the small GTPase superfamily. Ran family.
CC       {ECO:0000305}.
CC   -!- SEQUENCE CAUTION:
CC       Sequence=EAY74933.1; Type=Erroneous gene model prediction; Evidence={ECO:0000305};
DR   EMBL; CM000126; EAY74933.1; ALT_SEQ; Genomic_DNA.
DR   EMBL; CT855777; -; NOT_ANNOTATED_CDS; mRNA.
DR   SMR; A2WSI7; -.
DR   PRIDE; A2WSI7; -.
DR   eggNOG; KOG0096; Eukaryota.
DR   eggNOG; ENOG410XNRS; LUCA.
DR   HOGENOM; HOG000216664; -.
DR   Proteomes; UP000007015; Chromosome 1.
DR   GO; GO:0005634; C:nucleus; IEA:UniProtKB-SubCell.
DR   GO; GO:0005525; F:GTP binding; IEA:UniProtKB-KW.
DR   GO; GO:0003924; F:GTPase activity; IEA:InterPro.
DR   GO; GO:0006913; P:nucleocytoplasmic transport; IEA:InterPro.
DR   GO; GO:0015031; P:protein transport; IEA:UniProtKB-KW.
DR   InterPro; IPR027417; P-loop_NTPase.
DR   InterPro; IPR002041; Ran_GTPase.
DR   InterPro; IPR005225; Small_GTP-bd_dom.
DR   InterPro; IPR001806; Small_GTPase.
DR   PANTHER; PTHR24071; PTHR24071; 1.
DR   Pfam; PF00071; Ras; 1.
DR   PRINTS; PR00627; GTPRANTC4.
DR   SUPFAM; SSF52540; SSF52540; 1.
DR   TIGRFAMs; TIGR00231; small_GTP; 1.
DR   PROSITE; PS51418; RAN; 1.
PE   2: Evidence at transcript level;
KW   Complete proteome; GTP-binding; Nucleotide-binding; Nucleus;
KW   Protein transport; Reference proteome; Transport.
FT   CHAIN         1    221       GTP-binding nuclear protein Ran-1.
FT                                /FTId=PRO_0000347209.
FT   NP_BIND      21     28       GTP. {ECO:0000250|UniProtKB:P62825}.
FT   NP_BIND     125    128       GTP. {ECO:0000250|UniProtKB:P62825}.
FT   NP_BIND     153    155       GTP. {ECO:0000250|UniProtKB:P62825}.
FT   BINDING      71     71       GTP; via amide nitrogen.
FT                                {ECO:0000250|UniProtKB:P62825}.
SQ   SEQUENCE   221 AA;  25002 MW;  42BFE1527CC64E28 CRC64;
     MALPNQQTVD YPSFKLVIVG DGGTGKTTFV KRHLTGEFEK KYEPTIGVEV HPLDFFTNCG
     KIRFYCWDTA GQEKFGGLRD GYYIHGQCAI IMFDVTSRLT YKNVPTWHRD LCRVCENIPI
     VLCGNKVDVK NRQVKAKQVT FHRKKNLQYY EVSAKSNYNF EKPFLYLARK LAGDGNLHFV
     ETPALAPPDV TIDLAAQQQH EAELAAAAAQ PLPDDDDDLI E
```

MATRIX SCIENCE MASCOT Search Results

Protein View: PRE1_ARATH

Transcription factor PRE1 OS=Arabidopsis thaliana OX=3702 GN=PRE1 PE=1 SV=1

**Database:**	SwissProt
**Score:**	120
**Expect:**	4e-08
**Monoisotopic mass (M_r):**	10508
**Calculated pI:**	9.09
**Taxonomy:**	Arabidopsis thaliana

Sequence similarity is available as an NCBI BLAST search of PRE1_ARATH against nr.

Search parameters

**Enzyme:**	Trypsin: cuts C-term side of KR unless next residue is P.
**Mass values searched:**	8
**Mass values matched:**	8

Protein sequence coverage: 79%

Matched peptides shown in ***bold red***.

```
 1 MSNRRSRQSS SAPRISDNQM IDLVSKLRQI LPEIGQRRRS DKASASKVLQ
51 ETCNYIRNLN REVDNLSERL SQLLESVDED SPEAAVIRSL LM
```

Unformatted sequence string: 92 residues (for pasting into other applications).

Sort by ◉ residue number ○ increasing mass ○ decreasing mass
Show ◉ matched peptides only ○ predicted peptides also

Start – End	Observed	Mr(expt)	Mr(calc)	Delta	M	Peptide
1 – 4	507.2344	506.2271	506.2271	0.0000	0	-.MSNR.R
8 – 14	732.3635	731.3562	731.3562	0.0000	0	R.QSSSAPR.I
15 – 26	1362.6933	1361.6860	1361.6861	-0.0000	0	R.ISDNQMIDLVSK.L
29 – 37	1053.6051	1052.5978	1052.5978		0	R.QILPEIGQR.R
48 – 57	1238.6198	1237.6125	1237.6125	0.0000	0	K.VLQETCNYIR.N
58 – 61	516.2889	515.2816	515.2816	0.0001	0	R.NLNR.E
62 – 69	961.4585	960.4512	960.4512	0.0000	0	R.EVDNLSER.L
70 – 88	2071.0553	2070.0480	2070.0480	-0.0000	0	R.LSQLLESVDEDSPEAAVIR.S

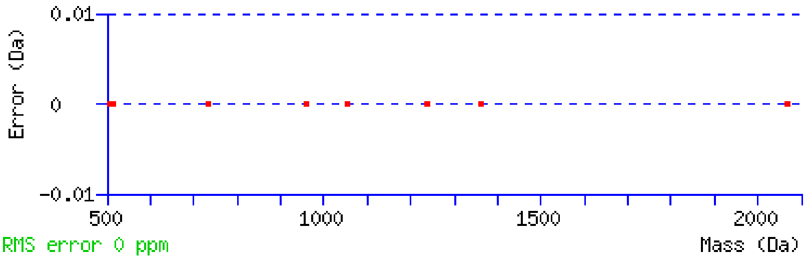

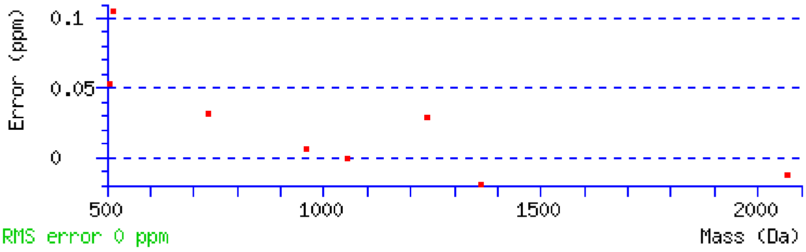

```
ID   PRE1_ARATH              Reviewed;          92 AA.
AC   Q9FLE9;
DT   14-MAY-2014, integrated into UniProtKB/Swiss-Prot.
DT   01-MAR-2001, sequence version 1.
DT   31-JUL-2019, entry version 109.
DE   RecName: Full=Transcription factor PRE1;
```

```
DE   AltName: Full=Basic helix-loop-helix protein 136;
DE            Short=AtbHLH136;
DE            Short=bHLH 136;
DE   AltName: Full=Protein BANQUO 1;
DE   AltName: Full=Protein PACLOBUTRAZOL RESISTANCE 1;
DE   AltName: Full=bHLH transcription factor bHLH136;
GN   Name=PRE1; Synonyms=BHLH136, BNQ1; OrderedLocusNames=At5g39860;
GN   ORFNames=MYH19.1;
OS   Arabidopsis thaliana (Mouse-ear cress).
OC   Eukaryota; Viridiplantae; Streptophyta; Embryophyta; Tracheophyta;
OC   Spermatophyta; Magnoliopsida; eudicotyledons; Gunneridae;
OC   Pentapetalae; rosids; malvids; Brassicales; Brassicaceae; Camelineae;
OC   Arabidopsis.
OX   NCBI_TaxID=3702;
RN   [1]
RP   NUCLEOTIDE SEQUENCE [LARGE SCALE GENOMIC DNA].
RC   STRAIN=cv. Columbia;
RX   PubMed=9628582; DOI=10.1093/dnares/5.1.41;
RA   Sato S., Kaneko T., Kotani H., Nakamura Y., Asamizu E., Miyajima N.,
RA   Tabata S.;
RT   "Structural analysis of Arabidopsis thaliana chromosome 5. IV.
RT   Sequence features of the regions of 1,456,315 bp covered by nineteen
RT   physically assigned P1 and TAC clones.";
RL   DNA Res. 5:41-54(1998).
RN   [2]
RP   GENOME REANNOTATION.
RC   STRAIN=cv. Columbia;
RX   PubMed=27862469; DOI=10.1111/tpj.13415;
RA   Cheng C.Y., Krishnakumar V., Chan A.P., Thibaud-Nissen F., Schobel S.,
RA   Town C.D.;
RT   "Araport11: a complete reannotation of the Arabidopsis thaliana
RT   reference genome.";
RL   Plant J. 89:789-804(2017).
RN   [3]
RP   NUCLEOTIDE SEQUENCE [LARGE SCALE MRNA].
RA   Brover V.V., Troukhan M.E., Alexandrov N.A., Lu Y.-P., Flavell R.B.,
RA   Feldmann K.A.;
RT   "Full-length cDNA from Arabidopsis thaliana.";
RL   Submitted (MAR-2002) to the EMBL/GenBank/DDBJ databases.
RN   [4]
RP   GENE FAMILY, AND NOMENCLATURE.
RX   PubMed=14600211; DOI=10.1105/tpc.151140;
RA   Bailey P.C., Martin C., Toledo-Ortiz G., Quail P.H., Huq E.,
RA   Heim M.A., Jakoby M., Werber M., Weisshaar B.;
RT   "Update on the basic helix-loop-helix transcription factor gene family
RT   in Arabidopsis thaliana.";
RL   Plant Cell 15:2497-2502(2003).
RN   [5]
RP   FUNCTION, TISSUE SPECIFICITY, AND INDUCTION BY GIBBERELLIN.
RX   PubMed=16527868; DOI=10.1093/pcp/pcj026;
RA   Lee S., Lee S., Yang K.Y., Kim Y.M., Park S.Y., Kim S.Y., Soh M.S.;
RT   "Overexpression of PRE1 and its homologous genes activates
RT   gibberellin-dependent responses in Arabidopsis thaliana.";
RL   Plant Cell Physiol. 47:591-600(2006).
RN   [6]
RP   FUNCTION, INTERACTION WITH IBH1, AND INDUCTION BY EPIBRASSINOLIDE.
RX   PubMed=20009022; DOI=10.1105/tpc.109.070441;
RA   Zhang L.Y., Bai M.Y., Wu J., Zhu J.Y., Wang H., Zhang Z., Wang W.,
RA   Sun Y., Zhao J., Sun X., Yang H., Xu Y., Kim S.H., Fujioka S.,
RA   Lin W.H., Chong K., Lu T., Wang Z.Y.;
RT   "Antagonistic HLH/bHLH transcription factors mediate brassinosteroid
RT   regulation of cell elongation and plant development in rice and
RT   Arabidopsis.";
RL   Plant Cell 21:3767-3780(2009).
RN   [7]
RP   FUNCTION, AND INTERACTION WITH HFR1.
RX   PubMed=20305124; DOI=10.1105/tpc.109.065946;
RA   Mara C.D., Huang T., Irish V.F.;
RT   "The Arabidopsis floral homeotic proteins APETALA3 and PISTILLATA
RT   negatively regulate the BANQUO genes implicated in light signaling.";
RL   Plant Cell 22:690-702(2010).
RN   [8]
RP   FUNCTION, AND INTERACTION WITH IBH1.
RX   PubMed=23161888; DOI=10.1105/tpc.112.105023;
RA   Ikeda M., Fujiwara S., Mitsuda N., Ohme-Takagi M.;
RT   "A triantagonistic basic helix-loop-helix system regulates cell
RT   elongation in Arabidopsis.";
RL   Plant Cell 24:4483-4497(2012).
RN   [9]
RP   FUNCTION, AND INTERACTION WITH IBH1.
```

```
RX   PubMed=23221598; DOI=10.1105/tpc.112.105163;
RA   Bai M.Y., Fan M., Oh E., Wang Z.Y.;
RT   "A triple helix-loop-helix/basic helix-loop-helix cascade controls
RT   cell elongation downstream of multiple hormonal and environmental
RT   signaling pathways in Arabidopsis.";
RL   Plant Cell 24:4917-4929(2012).
CC   -!- FUNCTION: Atypical and probable non DNA-binding bHLH transcription
CC       factor that integrates multiple signaling pathways to regulate
CC       cell elongation and plant development. Binds IBH1, forming a pair
CC       of antagonistic bHLH transcription factors that function
CC       downstream of BZR1 to mediate brassinosteroid regulation of cell
CC       elongation. Regulates light responses by binding and inhibiting
CC       the activity of the bHLH transcription factor HFR1, a critical
CC       regulator of light signaling and shade avoidance. May have a
CC       regulatory role in various aspects of gibberellin-dependent growth
CC       and development. {ECO:0000269|PubMed:16527868,
CC       ECO:0000269|PubMed:20009022, ECO:0000269|PubMed:20305124,
CC       ECO:0000269|PubMed:23161888, ECO:0000269|PubMed:23221598}.
CC   -!- SUBUNIT: Interacts with IBH1 and HFR1.
CC       {ECO:0000269|PubMed:20009022, ECO:0000269|PubMed:20305124,
CC       ECO:0000269|PubMed:23161888, ECO:0000269|PubMed:23221598}.
CC   -!- SUBCELLULAR LOCATION: Nucleus {ECO:0000305}.
CC   -!- TISSUE SPECIFICITY: Expressed in roots, leaves, stems and flowers.
CC       {ECO:0000269|PubMed:16527868}.
CC   -!- INDUCTION: By gibberellin and epibrassinolide.
CC       {ECO:0000269|PubMed:16527868, ECO:0000269|PubMed:20009022}.
CC   -!- MISCELLANEOUS: Gain-of-function mutants (T-DNA tagging) show long
CC       hypocotyls, pale green and slightly narrow leaves, elongated
CC       petioles and early flowering. They are not sensitive to the
CC       gibberellin inhibitor paclobutrazol during seed germination
CC       (PubMed:16527868, PubMed:20009022, PubMed:23221598).
CC       {ECO:0000305|PubMed:16527868, ECO:0000305|PubMed:20009022,
CC       ECO:0000305|PubMed:23221598}.
DR   EMBL; AB010077; BAB10210.1; -; Genomic_DNA.
DR   EMBL; CP002688; AED94484.1; -; Genomic_DNA.
DR   EMBL; AY088246; AAM65786.1; -; mRNA.
DR   RefSeq; NP_198802.1; NM_123349.3.
DR   BioGrid; 19233; 9.
DR   IntAct; Q9FLE9; 7.
DR   STRING; 3702.AT5G39860.1; -.
DR   iPTMnet; Q9FLE9; -.
DR   PaxDb; Q9FLE9; -.
DR   EnsemblPlants; AT5G39860.1; AT5G39860.1; AT5G39860.
DR   GeneID; 833982; -.
DR   Gramene; AT5G39860.1; AT5G39860.1; AT5G39860.
DR   KEGG; ath:AT5G39860; -.
DR   Araport; AT5G39860; -.
DR   TAIR; locus:2178002; AT5G39860.
DR   eggNOG; ENOG410JGIP; Eukaryota.
DR   eggNOG; ENOG41118WW; LUCA.
DR   HOGENOM; HOG000239487; -.
DR   InParanoid; Q9FLE9; -.
DR   OMA; PESQTRN; -.
DR   OrthoDB; 1589357at2759; -.
DR   PhylomeDB; Q9FLE9; -.
DR   PRO; PR:Q9FLE9; -.
DR   Proteomes; UP000006548; Chromosome 5.
DR   ExpressionAtlas; Q9FLE9; baseline and differential.
DR   Genevisible; Q9FLE9; AT.
DR   GO; GO:0005634; C:nucleus; IEA:UniProtKB-SubCell.
DR   GO; GO:0003700; F:DNA-binding transcription factor activity; ISS:TAIR.
DR   GO; GO:0046983; F:protein dimerization activity; IEA:InterPro.
DR   GO; GO:0009742; P:brassinosteroid mediated signaling pathway; IEA:UniProtKB-KW.
DR   GO; GO:0009740; P:gibberellic acid mediated signaling pathway; IEA:UniProtKB-KW.
DR   GO; GO:0009640; P:photomorphogenesis; IMP:TAIR.
DR   GO; GO:0040008; P:regulation of growth; IEA:UniProtKB-KW.
DR   GO; GO:0048510; P:regulation of timing of transition from vegetative to reproductive phase; IMP:TAIR.
DR   GO; GO:0009741; P:response to brassinosteroid; IEP:TAIR.
DR   GO; GO:0009826; P:unidimensional cell growth; IMP:TAIR.
DR   Gene3D; 4.10.280.10; -; 1.
DR   InterPro; IPR011598; bHLH_dom.
DR   InterPro; IPR036638; HLH_DNA-bd_sf.
DR   Pfam; PF00010; HLH; 1.
DR   SUPFAM; SSF47459; SSF47459; 1.
DR   PROSITE; PS50888; BHLH; 1.
PE   1: Evidence at protein level;
KW   Brassinosteroid signaling pathway; Complete proteome;
KW   Gibberellin signaling pathway; Growth regulation; Nucleus;
KW   Reference proteome; Transcription; Transcription regulation.
FT   CHAIN         1     92       Transcription factor PRE1.
```

```
FT                                /FTId=PRO_0000429083.
FT   DOMAIN        4     59       bHLH. {ECO:0000255|PROSITE-
FT                                ProRule:PRU00981}.
SQ   SEQUENCE   92 AA;  10515 MW;  172F1DA7A18857C2 CRC64;
     MSNRRSRQSS SAPRISDNQM IDLVSKLRQI LPEIGQRRRS DKASASKVLQ ETCNYIRNLN
     REVDNLSERL SQLLESVDED SPEAAVIRSL LM
```

Mascot: http://www.matrixscience.com/

MASCOT Search Results

Protein View: FIE1_ORYSJ

Polycomb group protein FIE1 OS=Oryza sativa subsp. japonica OX=39947 GN=FIE1 PE=1 SV=1

**Database:**	SwissProt
**Score:**	99
**Expect:**	4.6e-06
**Monoisotopic mass (M_r):**	51816
**Calculated pI:**	7.55
**Taxonomy:**	Oryza sativa Japonica Group

Sequence similarity is available as an NCBI BLAST search of FIE1_ORYSJ against nr.

Search parameters

**Enzyme:**	Trypsin: cuts C-term side of KR unless next residue is P.
**Mass values searched:**	17
**Mass values matched:**	10

Protein sequence coverage: 44%

Matched peptides shown in ***bold red***.

```
  1 MGPTSRNHKS SQKDVAPNEA KPPRYPQRNR SITASASASA FASPAVANSR
 51 VAKERPSSST AGEGEPQETV LKLPSIPTLP ARMAKLVPLE GLGCEAAVGS
101 LTPSREREYK VTNKHTEGRR PVYAIVFNFL DVRYYDIFAT ACGPRLSTYR
151 CLMNGKFALL QSYLDDDMNE SFFTVSWACD IDGNPLLVAA GSTGIIRVIN
201 CATEKIYKSL VGHGGSVNEI KSQPSNPSLI ISASKDESIK LWNVQTGILI
251 LVFGGVGGHR HEVLGVDFHT SDIYRFLSCG MDNTVRIWSM KEFWEYVEKS
301 YSWTDATSKF PTKFVQFPVL CAEIHSNYVD CTKWLGDFVL SKSVENEILL
351 WESITKEENP GEGHIDVLQK YPVPECNIWF MKFSCDFHHN QLAIGNRDGK
401 VYVWKVQTSP PVLIARLNNP QVKSAIRQTA VSFDGSTILA CTEDGNIWRW
451 DEVDHPTAPV PSKKQK
```

Unformatted sequence string: 466 residues (for pasting into other applications).

Sort by ◉ residue number ○ increasing mass ○ decreasing mass
Show ◉ matched peptides only ○ predicted peptides also

Start - End	Observed	Mr(expt)	Mr(calc)	Delta	M	Peptide
31 - 50	1865.9351	1864.9278	1864.9279	-0.0000	0	R.SITASASASAFASPAVANSR.V
54 - 72	2001.9723	2000.9650	2000.9651	-0.0000	0	K.ERPSSSTAGEGEPQETVLK.L
86 - 105	1969.0422	1968.0349	1968.0350	-0.0001	0	K.LVPLEGLGCEAAVGSLTPSR.E
120 - 133	1708.9533	1707.9460	1707.9461	-0.0000	0	R.RPVYAIVFNFLDVR.Y
157 - 197	4465.1366	4464.1293	4464.1294	-0.0001	0	K.FALLQSYLDDDMNESFFTVSWACDIDGNPLLVAAGSTGIIR.V
241 - 260	2136.2076	2135.2003	2135.2004	-0.0001	0	K.LWNVQTGILILVFGGVGGHR.H
261 - 275	1787.8711	1786.8638	1786.8639	-0.0000	0	R.HEVLGVDFHTSDIYR.F
314 - 333	2313.1042	2312.0969	2312.0970	-0.0000	0	K.FVQFPVLCAEIHSNYVDCTK.W
383 - 397	1758.8129	1757.8056	1757.8056	0.0000	0	K.FSCDFHHNQLAIGNR.D
428 - 449	2384.1187	2383.1114	2383.1114	-0.0000	0	R.QTAVSFDGSTILACTEDGNIWR.W

No match to: 120.0000, 157.0000, 241.0000, 261.0000, 314.0000, 383.0000, 428.0000

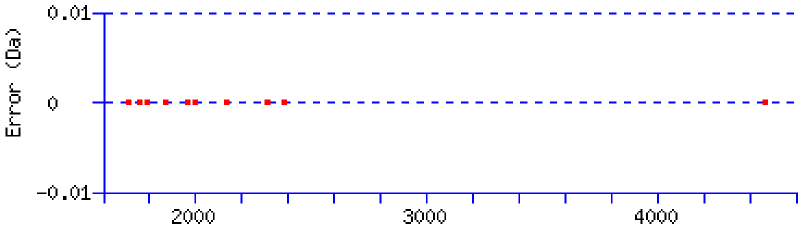

RMS error 0 ppm

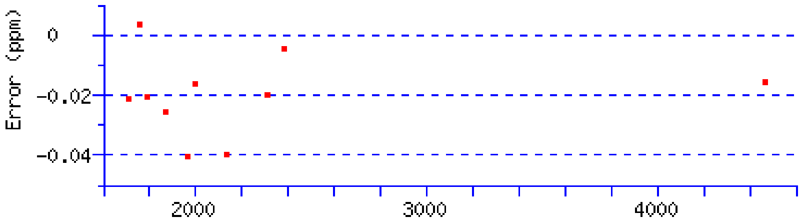

RMS error 0 ppm

```
ID   FIE1_ORYSJ              Reviewed;         466 AA.
AC   Q6ZJW8; C7J630;
DT   20-JUN-2018, integrated into UniProtKB/Swiss-Prot.
DT   05-JUL-2004, sequence version 1.
DT   31-JUL-2019, entry version 110.
DE   RecName: Full=Polycomb group protein FIE1 {ECO:0000305};
DE   AltName: Full=Protein FERTILIZATION-INDEPENDENT ENDOSPERM 1 {ECO:0000303|PubMed:19825651};
DE            Short=OsFIE1 {ECO:0000303|PubMed:19825651};
DE   AltName: Full=WD40 repeat-containing protein 154 {ECO:0000303|PubMed:22429805};
DE            Short=OsWD40-154 {ECO:0000303|PubMed:22429805};
GN   Name=FIE1 {ECO:0000303|PubMed:19825651};
GN   OrderedLocusNames=Os08g0137250 {ECO:0000312|EMBL:BAT03753.1},
GN   LOC_Os08g04290 {ECO:0000305};
GN   ORFNames=OJ1613_G04.22 {ECO:0000312|EMBL:BAD03075.1},
GN   OsJ_25973 {ECO:0000312|EMBL:EAZ41450.1};
OS   Oryza sativa subsp. japonica (Rice).
OC   Eukaryota; Viridiplantae; Streptophyta; Embryophyta; Tracheophyta;
OC   Spermatophyta; Magnoliopsida; Liliopsida; Poales; Poaceae; BOP clade;
OC   Oryzoideae; Oryzeae; Oryzinae; Oryza; Oryza sativa.
OX   NCBI_TaxID=39947;
RN   [1]
RP   NUCLEOTIDE SEQUENCE [LARGE SCALE GENOMIC DNA].
RC   STRAIN=cv. Nipponbare;
RX   PubMed=16100779; DOI=10.1038/nature03895;
RG   International rice genome sequencing project (IRGSP);
RT   "The map-based sequence of the rice genome.";
RL   Nature 436:793-800(2005).
RN   [2]
RP   GENOME REANNOTATION.
RC   STRAIN=cv. Nipponbare;
RX   PubMed=18089549; DOI=10.1093/nar/gkm978;
RG   The rice annotation project (RAP);
RT   "The rice annotation project database (RAP-DB): 2008 update.";
RL   Nucleic Acids Res. 36:D1028-D1033(2008).
RN   [3]
RP   GENOME REANNOTATION.
RC   STRAIN=cv. Nipponbare;
RX   PubMed=24280374; DOI=10.1186/1939-8433-6-4;
RA   Kawahara Y., de la Bastide M., Hamilton J.P., Kanamori H.,
RA   McCombie W.R., Ouyang S., Schwartz D.C., Tanaka T., Wu J., Zhou S.,
RA   Childs K.L., Davidson R.M., Lin H., Quesada-Ocampo L.,
RA   Vaillancourt B., Sakai H., Lee S.S., Kim J., Numa H., Itoh T.,
RA   Buell C.R., Matsumoto T.;
RT   "Improvement of the Oryza sativa Nipponbare reference genome using
RT   next generation sequence and optical map data.";
RL   Rice 6:4-4(2013).
RN   [4]
RP   NUCLEOTIDE SEQUENCE [LARGE SCALE GENOMIC DNA].
RC   STRAIN=cv. Nipponbare;
RX   PubMed=15685292; DOI=10.1371/journal.pbio.0030038;
RA   Yu J., Wang J., Lin W., Li S., Li H., Zhou J., Ni P., Dong W., Hu S.,
RA   Zeng C., Zhang J., Zhang Y., Li R., Xu Z., Li S., Li X., Zheng H.,
RA   Cong L., Lin L., Yin J., Geng J., Li G., Shi J., Liu J., Lv H., Li J.,
RA   Wang J., Deng Y., Ran L., Shi X., Wang X., Wu Q., Li C., Ren X.,
RA   Wang J., Wang X., Li D., Liu D., Zhang X., Ji Z., Zhao W., Sun Y.,
RA   Zhang Z., Bao J., Han Y., Dong L., Ji J., Chen P., Wu S., Liu J.,
RA   Xiao Y., Bu D., Tan J., Yang L., Ye C., Zhang J., Xu J., Zhou Y.,
RA   Yu Y., Zhang B., Zhuang S., Wei H., Liu B., Lei M., Yu H., Li Y.,
```

```
RA   Xu H., Wei S., He X., Fang L., Zhang Z., Zhang Y., Huang X., Su Z.,
RA   Tong W., Li J., Tong Z., Li S., Ye J., Wang L., Fang L., Lei T.,
RA   Chen C.-S., Chen H.-C., Xu Z., Li H., Huang H., Zhang F., Xu H.,
RA   Li N., Zhao C., Li S., Dong L., Huang Y., Li L., Xi Y., Qi Q., Li W.,
RA   Zhang B., Hu W., Zhang Y., Tian X., Jiao Y., Liang X., Jin J., Gao L.,
RA   Zheng W., Hao B., Liu S.-M., Wang W., Yuan L., Cao M., McDermott J.,
RA   Samudrala R., Wang J., Wong G.K.-S., Yang H.;
RT   "The genomes of Oryza sativa: a history of duplications.";
RL   PLoS Biol. 3:266-281(2005).
RN   [5]
RP   NUCLEOTIDE SEQUENCE [LARGE SCALE MRNA].
RC   STRAIN=cv. Nipponbare;
RG   The rice full-length cDNA consortium;
RT   "Oryza sativa full length cDNA.";
RL   Submitted (OCT-2006) to the EMBL/GenBank/DDBJ databases.
RN   [6]
RP   TISSUE SPECIFICITY, AND IMPRINTING.
RX   PubMed=19825651; DOI=10.1093/mp/ssp036;
RA   Luo M., Platten D., Chaudhury A., Peacock W.J., Dennis E.S.;
RT   "Expression, imprinting, and evolution of rice homologs of the
RT   polycomb group genes.";
RL   Mol. Plant 2:711-723(2009).
RN   [7]
RP   GENE FAMILY, NOMENCLATURE, AND TISSUE SPECIFICITY.
RX   PubMed=22429805; DOI=10.1186/1471-2164-13-100;
RA   Ouyang Y., Huang X., Lu Z., Yao J.;
RT   "Genomic survey, expression profile and co-expression network analysis
RT   of OsWD40 family in rice.";
RL   BMC Genomics 13:100-100(2012).
RN   [8]
RP   FUNCTION, SUBUNIT, AND INTERACTION WITH EZ1 AND CLF.
RX   PubMed=23150632; DOI=10.1105/tpc.112.102269;
RA   Zhang L., Cheng Z., Qin R., Qiu Y., Wang J.L., Cui X., Gu L.,
RA   Zhang X., Guo X., Wang D., Jiang L., Wu C.Y., Wang H., Cao X., Wan J.;
RT   "Identification and characterization of an epi-allele of FIE1 reveals
RT   a regulatory linkage between two epigenetic marks in rice.";
RL   Plant Cell 24:4407-4421(2012).
RN   [9]
RP   FUNCTION, AND TISSUE SPECIFICITY.
RX   PubMed=27133784; DOI=10.1111/tpj.13202;
RA   Huang X., Lu Z., Wang X., Ouyang Y., Chen W., Xie K., Wang D., Luo M.,
RA   Luo J., Yao J.;
RT   "Imprinted gene OsFIE1 modulates rice seed development by influencing
RT   nutrient metabolism and modifying genome H3K27me3.";
RL   Plant J. 87:305-317(2016).
CC   -!- FUNCTION: Polycomb group (PcG) protein. PcG proteins act by
CC       forming multiprotein complexes, which are required to maintain the
CC       transcriptionally repressive state of homeotic genes throughout
CC       development. PcG proteins are not required to initiate repression,
CC       but to maintain it during later stages of development. They act
CC       via the methylation of histones, rendering chromatin heritably
CC       changed in its expressibility (PubMed:23150632, PubMed:27133784).
CC       Together with EZ1 and CLF forms a complex that is involved in gene
CC       transcriptional repression by trimethylation on histone H3 'Lys-
CC       27' (H3K27me3) of target genes (PubMed:23150632). Involved in the
CC       regulation of embryo and seed endosperm development. FIE1-
CC       containing PcG complex in seed endosperm regulates the expression
CC       of various transcription factors by trimethylation on histone H3
CC       'Lys-27' (H3K27me3) of target genes. Involved in the overall
CC       expression regulation of nutrient metabolism genes, such as
CC       prolamin synthesis and seed storage protein synthesis genes. Can
CC       regulate valine, leucine and isoleucine metabolism-related genes
CC       (PubMed:27133784). {ECO:0000269|PubMed:23150632,
CC       ECO:0000269|PubMed:27133784}.
CC   -!- SUBUNIT: Interacts with EZ1 and CLF (PubMed:23150632). Component
CC       of the polycomb repressive complex 2 (PRC2), which methylates
CC       'Lys-27' residues of histone H3 (H3K27me3), leading to
CC       transcriptional repression of the affected target gene
CC       (PubMed:23150632). {ECO:0000269|PubMed:23150632}.
CC   -!- TISSUE SPECIFICITY: Expressed specifically in seed endosperm.
CC       {ECO:0000269|PubMed:19825651, ECO:0000269|PubMed:22429805,
CC       ECO:0000269|PubMed:27133784}.
CC   -!- MISCELLANEOUS: The FIE1 locus is imprinted. Maternal inherited
CC       gene is expressed in the ovule (the egg and the central cell),
CC       while the paternal inherited gene is silenced in the pollen. After
CC       fertilization, only the maternal inherited allele is expressed
CC       (PubMed:19825651). The gain-of-function epi-allele (Epi-df) plants
CC       exhibit dwarf phenotype, defect in flower development and very
CC       poor seed set (PubMed:23150632). Plants silencing FIE1 have
CC       delayed embryo development, reduced seed set, and reduced grain
```

```
CC       length, width and weight (PubMed:27133784).
CC       {ECO:0000269|PubMed:19825651, ECO:0000269|PubMed:23150632,
CC       ECO:0000269|PubMed:27133784}.
CC   -!- SIMILARITY: Belongs to the WD repeat ESC family. {ECO:0000305}.
CC   -!- SEQUENCE CAUTION:
CC       Sequence=BAH94100.1; Type=Erroneous gene model prediction; Evidence={ECO:0000305};
DR   EMBL; AP003896; BAD03075.1; -; Genomic_DNA.
DR   EMBL; AP008214; BAH94100.1; ALT_SEQ; Genomic_DNA.
DR   EMBL; AP014964; BAT03753.1; -; Genomic_DNA.
DR   EMBL; CM000145; EAZ41450.1; -; Genomic_DNA.
DR   EMBL; AK242200; BAH01221.1; -; mRNA.
DR   RefSeq; XP_015649120.1; XM_015793634.1.
DR   SMR; Q6ZJW8; -.
DR   STRING; 4530.OS08T0137250-01; -.
DR   PaxDb; Q6ZJW8; -.
DR   EnsemblPlants; Os08t0137250-01; Os08t0137250-01; Os08g0137250.
DR   GeneID; 9271694; -.
DR   Gramene; Os08t0137250-01; Os08t0137250-01; Os08g0137250.
DR   KEGG; osa:9271694; -.
DR   eggNOG; KOG1034; Eukaryota.
DR   eggNOG; ENOG410XRQI; LUCA.
DR   HOGENOM; HOG000005759; -.
DR   InParanoid; Q6ZJW8; -.
DR   OMA; HVCIAIF; -.
DR   OrthoDB; 1191277at2759; -.
DR   Proteomes; UP000059680; Chromosome 8.
DR   GO; GO:0005677; C:chromatin silencing complex; IMP:UniProtKB.
DR   GO; GO:0035098; C:ESC/E(Z) complex; IBA:GO_Central.
DR   GO; GO:0031519; C:PcG protein complex; IDA:UniProtKB.
DR   GO; GO:0030154; P:cell differentiation; IEA:UniProtKB-KW.
DR   GO; GO:0006342; P:chromatin silencing; IMP:UniProtKB.
DR   GO; GO:0009793; P:embryo development ending in seed dormancy; IMP:UniProtKB.
DR   GO; GO:0009960; P:endosperm development; IMP:UniProtKB.
DR   GO; GO:0009908; P:flower development; IMP:UniProtKB.
DR   GO; GO:0070734; P:histone H3-K27 methylation; IMP:UniProtKB.
DR   GO; GO:0000122; P:negative regulation of transcription by RNA polymerase II; IBA:GO_Central.
DR   GO; GO:0048316; P:seed development; IMP:UniProtKB.
DR   Gene3D; 2.130.10.10; -; 1.
DR   InterPro; IPR020472; G-protein_beta_WD-40_rep.
DR   InterPro; IPR015943; WD40/YVTN_repeat-like_dom_sf.
DR   InterPro; IPR001680; WD40_repeat.
DR   InterPro; IPR019775; WD40_repeat_CS.
DR   InterPro; IPR017986; WD40_repeat_dom.
DR   InterPro; IPR036322; WD40_repeat_dom_sf.
DR   Pfam; PF00400; WD40; 2.
DR   PRINTS; PR00320; GPROTEINBRPT.
DR   SMART; SM00320; WD40; 6.
DR   SUPFAM; SSF50978; SSF50978; 1.
DR   PROSITE; PS00678; WD_REPEATS_1; 1.
DR   PROSITE; PS50082; WD_REPEATS_2; 2.
DR   PROSITE; PS50294; WD_REPEATS_REGION; 1.
PE   1: Evidence at protein level;
KW   Chromatin regulator; Complete proteome; Developmental protein;
KW   Differentiation; Reference proteome; Repeat; Transcription;
KW   Transcription regulation; WD repeat.
FT   CHAIN         1    466       Polycomb group protein FIE1.
FT                                /FTId=PRO_0000444461.
FT   REPEAT      167    209       WD 1. {ECO:0000255}.
FT   REPEAT      212    252       WD 2. {ECO:0000255}.
FT   REPEAT      258    298       WD 3. {ECO:0000255}.
FT   REPEAT      324    361       WD 4. {ECO:0000255}.
FT   REPEAT      374    414       WD 5. {ECO:0000255}.
FT   REPEAT      421    460       WD 6. {ECO:0000255}.
SQ   SEQUENCE   466 AA;  51849 MW;  4F230794D8505052 CRC64;
     MGPTSRNHKS SQKDVAPNEA KPPRYPQRNR SITASASASA FASPAVANSR VAKERPSSST
     AGEGEPQETV LKLPSIPTLP ARMAKLVPLE GLGCEAAVGS LTPSREREYK VTNKHTEGRR
     PVYAIVFNFL DVRYYDIFAT ACGPRLSTYR CLMNGKFALL QSYLDDDMNE SFFTVSWACD
     IDGNPLLVAA GSTGIIRVIN CATEKIYKSL VGHGGSVNEI KSQPSNPSLI ISASKDESIK
     LWNVQTGILI LVFGGVGGHR HEVLGVDFHT SDIYRFLSCG MDNTVRIWSM KEFWEYVEKS
     YSWTDATSKF PTKFVQFPVL CAEIHSNYVD CTKWLGDFVL SKSVENEILL WESITKEENP
     GEGHIDVLQK YPVPECNIWF MKFSCDFHHN QLAIGNRDGK VYVWKVQTSP PVLIARLNNP
     QVKSAIRQTA VSFDGSTILA CTEDGNIWRW DEVDHPTAPV PSKKQK
```
